# The Discovery of Novel Ferulic Acid Derivatives Incorporating Substituted Isopropanolamine Moieties as Potential Tobacco Mosaic Virus Helicase Inhibitors

**DOI:** 10.3390/ijms232213991

**Published:** 2022-11-13

**Authors:** Zhenxing Li, Binxin Yang, Hongwu Liu, Yue Ding, Zimian Fang, Wubin Shao, Puying Qi, Xiang Zhou, Liwei Liu, Song Yang

**Affiliations:** State Key Laboratory Breeding Base of Green Pesticide and Agricultural Bioengineering, Key Laboratory of Green Pesticide and Agricultural Bioengineering, Ministry of Education, Center for R & D of Fine Chemicals, Guizhou University, Guiyang 550025, China

**Keywords:** ferulic acid, antiviral assay, molecular docking, helicase, inhibitor

## Abstract

Target-based drug design, a high-efficiency strategy used to guide the development of novel pesticide candidates, has attracted widespread attention. Herein, various natural-derived ferulic acid derivatives incorporating substituted isopropanolamine moieties were designed to target the tobacco mosaic virus (TMV) helicase. Bioassays demonstrating the optimized **A_19_**, **A_20_**, **A_29_**, and **A_31_** displayed excellent in vivo antiviral curative abilities, affording corresponding EC_50_ values of 251.1, 336.2, 347.1, and 385.5 μg/mL, which visibly surpassed those of commercial ribavirin (655.0 μg/mL). Moreover, configurational analysis shows that the *R*-forms of target compounds were more beneficial to aggrandize antiviral profiles. Mechanism studies indicate that ***R*-A_19_** had a strong affinity (*K*_d_ = 5.4 μM) to the TMV helicase and inhibited its ability to hydrolyze ATP (50.61% at 200 μM). Meanwhile, **A_19_** could down-regulate the expression of the TMV helicase *gene* in the host to attenuate viral replication. These results illustrate the excellent inhibitory activity of **A_19_** towards the TMV helicase. Additionally, docking simulations uncovered that ***R*-A_19_** formed more hydrogen bonds with the TMV helicase in the binding pocket. Recent studies have unambiguously manifested that these designed derivatives could be considered as promising potential helicase-based inhibitors for plant disease control.

## 1. Introduction

The ever-increasing malignant tobacco mosaic virus (TMV) is a cardinal agricultural and horticultural plant pathogenic virus that leads to enormous yield losses and cripples the quality of crops [1,2,3,4]. Pesticides are consistently viewed to be one of the most effective tools for preventing and controlling causative agents while reducing labor costs. Nonetheless, with the evolution and resistance development of viruses, obsolete pesticides are incapable of maintaining their efficacy sustainably [5,6]. Therefore, it is highly imperative and urgent to continuously exploit novel pesticidal candidates which empower distinct mechanisms of action to address this complex dilemma.

Helicases are present in virtually all living organisms, including eukaryotes, yeast, bacteria, and viruses. Typically, they act as molecular motors, employing the energy generated by hydrolyzing nucleoside triphosphates to break the hydrogen bonds between base pairs, thereby unwinding double-strand nucleic acid in preparation for replication [7,8,9,10]. Based on substrate specificity, helicases can be divided into two categories: DNA helicases and RNA helicases. DNA helicases are essential for DNA replication, recombination, transcription, and repair. RNA helicases regulate RNA structure and are involved in various aspects of RNA metabolism, such as transcription, translation, RNA splicing, ribosome assembly, RNA editing, and RNA degradation [11,12,13,14]. As a result, helicases-targeted drug design has attracted growing interest from researchers. Currently, the design of viral inhibitors is mainly based on RNA helicases. Frick and colleagues have screened a series of antiviral candidates with potential application prospects targeting virus helicase [15,16,17,18]. Shah et al. reviewed the nature-derived phyllaemblicin with inhibitory activity toward helicase NSp13 activity in SARS-CoV-2 [19]. Greger reported promising flavonoids that inhibit the synthesis of proteins by interacting with an ATP-dependent DEAD-box RNA helicase [20]. While the overwhelming majority of studies mainly focused on the research of animal viruses, inspired by these investigations, targeting plant viral RNA helicase may provide valuable orientations for the discovery of potential viral inhibitors.

**Figure 1:**
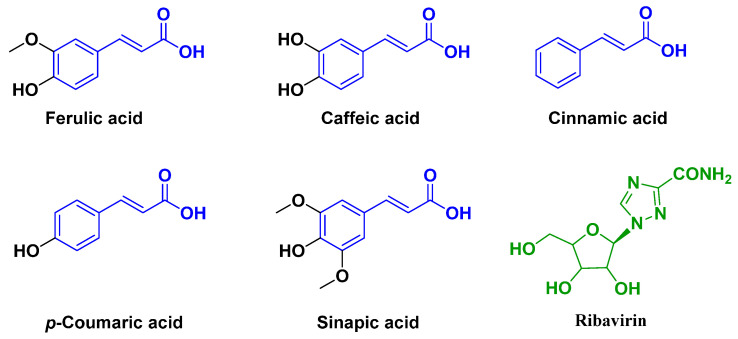
The structures of ferulic acid and its analogues, as well as the commercial antiviral agent (ribavirin).

Naturally occurring products possess structural diversity, low mammalian toxicity, environmental friendliness, specificity to target species, unique modes of action, and can be deployed as ideal pesticide scaffolds [21,22]. Ferulic acid (3-methoxy-4-hydroxycinnamic acid), as an active ingredient, is widely distributed in *Ferula ferulae*, *Angelica sinensis*, *Ligusticum wallichii*, *Cimicifuga foetida*, and other Chinese herbal medicine with a wide range of medicinal value. Undoubtedly, ferulic acid (FA), together with its analogs (Figure 1), exhibits broad-spectrum medicinal or pesticide bioactivities, such as antifungal, antibacterial, antioxidant, and herbicidal activity [23,24,25,26,27]. More importantly, research showed that FA [28,29,30] and FA analogues [31,32] possess potential virus helicase inhibitory activity, along with an excellent antiviral profile (cold viruses, respiratory syncytial virus, plant viruses, etc.) [28,33]. On the other hand, privileged versatile isopropylamine fragments have the functions of facilitating target scaffold flexibility, transforming water solubility, and enhancing ligand–receptor H-bond interactions, and they have been wildly applicable in pesticide design [34,35].

To excavate novel plant viral helicase inhibitors in view of the above considerations, a series of FA derivatives containing different substitutions of isopropanolamine fragments were successfully synthesized and screened for anti-TMV activities. Subsequently, the circular dichroism (CD) spectrum, microscale thermophoresis (MST), and ATPase activity analysis were applied to explore the preliminary mechanism of target compounds with the TMV helicase and simulated by molecular docking and density functional theory (DFT) calculation. Additionally, quantitative real-time polymerase chain reaction (RT-qPCR) experiments were conducted to assess potential inhibition of title compounds for TMV helicase *gene* expression. Moreover, pot experiments were conducted to further investigate the effect of target compounds on TMV propagation in the plant *Nicotiana benthamiana*. To explore the multifunctional applications of these compounds in agriculture, corresponding bioassays against phytopathogenic bacteria were also evaluated.

## 2. Results and Discussion

### 2.1. Chemistry and Antiviral Activity In Vivo

**Figure 2:**
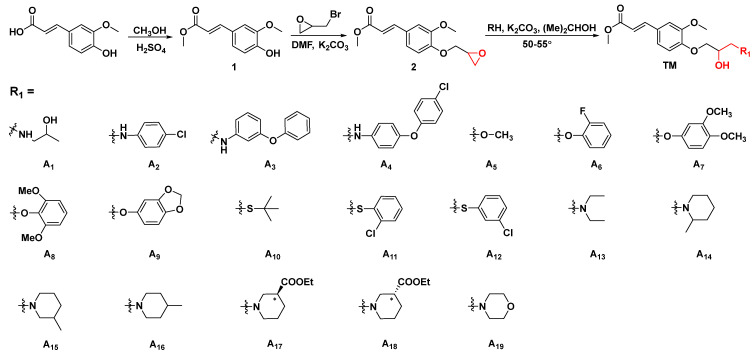
The synthetic route for the target molecules **A_1_**–**A_19_**.

In view of pesticidal candidate exploration, nature-derived low-cost structures containing elevated potency biological activity with a brief synthetic route can be seen as practical. The designed synthetic route of title derivatives is depicted in Figure 2. In short, the starting material FA was esterified by methanol to provide intermediate **1**. Then, through substitution with epibromohydrin, the crucial intermediate **2** was successfully prepared. After that, through a typically ring-opening reaction between intermediate **2** with substituents containing different heteroatoms, title FA-originated derivatives incorporating disparate substitutions of isopropanolamine fragments (**A_1_**–**A_19_**) were afforded. All molecular structures were confirmed by NMR and HRMS (Supporting Information). In this bioassay, a classical half leaf-inoculation approach was employed to validate the antiviral profile of **A_1_**–**A_19_** toward TMV in vivo, with the starting material FA and commercial virucide ribavirin (Ri) used as controls for comparison. The synthesized target compounds (**A_1_**–**A_19_**) of antiviral results are shown in Table 1. It could be found that various heteroatom substituted molecules exhibited divergent degrees of biological activities (focused on curative effect). Among them, **A_3_** and **A_4_** displayed higher and the highest antiviral efficacy, respectively, as follows: **A_4_** (R = 4-(4-chlorophenoxy)anilino, 43.8%) > Ri (42.1%) > **A_3_** (R = 3-phenoxyanilino, 38.6%) > **A_11_** (R = (2-chlorophenyl)thio, 36.1%) > **A_1_** (R = 2-hydroxypropylamino, 32.7%) ≈ FA (31.4%) > **A_5_** (R = methoxyl, 10.3%) at 500 μg/mL, indicating that the antiviral capacity of target compounds obtained by the ring opening of nitrogen-containing substituents was evidently higher than that substituted by oxygen or sulfur. Meaningfully, compared with the starting material, target FA derivatives formed by the ring opening of the nitrogen-containing group had the potential to confer enhanced biological activity. Based on this outcome, the noncyclic amine (**A_13_**) and cyclic amine (**A_14_**) fragments were introduced into the target structure to further evaluate the effect of the substituents on the biological activity of the molecular backbone. The therapeutic activity of piperidine substitution was generally higher than that of aniline and fatty amine, such as **A_14_** (R = 2-methylpiperidyl, 41.5%) > **A_13_** (R = diethylin, 28.3%) > **A_2_** (R = 4-chlorophenylamino, 19.2%) at 500 μg/mL. This result shows that the introduction of cyclic amine may refurbish the designed molecular antiviral potency. Therefore, some target compounds containing six-membered heterocyclic structures were derived (**A_15_**–**A_19_**). As shown in Table 1, compared with commercial Ri, the applausive antiviral abilities of **A_16_** and **A_19_** were obtained after incorporating methylpiperidyl or morpholine into the target structure. Moreover, the position of the methyl group on the piperidine ring also possessed a definite effect on the final anti-TMV effectiveness. The order is as follows: **A_16_** (R = 4-methylpiperidyl, 46.5%) > **A_15_** (R = 3-methylpiperidyl, 43.1%) > Ri (42.1%) ≈ **A_14_** (R = 2-methylpiperidyl, 41.5%) at 500 μg/mL, suggesting that the methyl group at the fourth position could more efficiently facilitate empower bioactivity. Additionally, when replacing the methyl of piperidyl by the ethyl formate group, the relevant **A_17_** and **A_18_** (21.7% and 27.1% at 500 μg/mL, respectively) had a significantly declined ability against TMV, demonstrating that the ester group with relatively steric hindrance and dipole interactions was unconducive to activity. Interestingly, when the heteroatom on the six-membered ring was increased, i.e., when piperidine was replaced by morpholine, the obtained **A_19_** showed the strongest antiviral competence (62.7% at 500 μg/mL).

**Figure 3:**
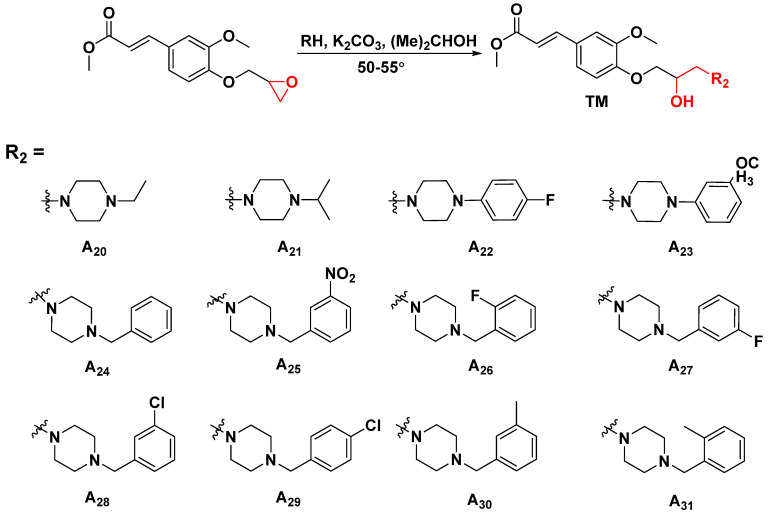
Synthetic routes for the target molecules **A_20_**–**A_31_**.

**Figure 4:**
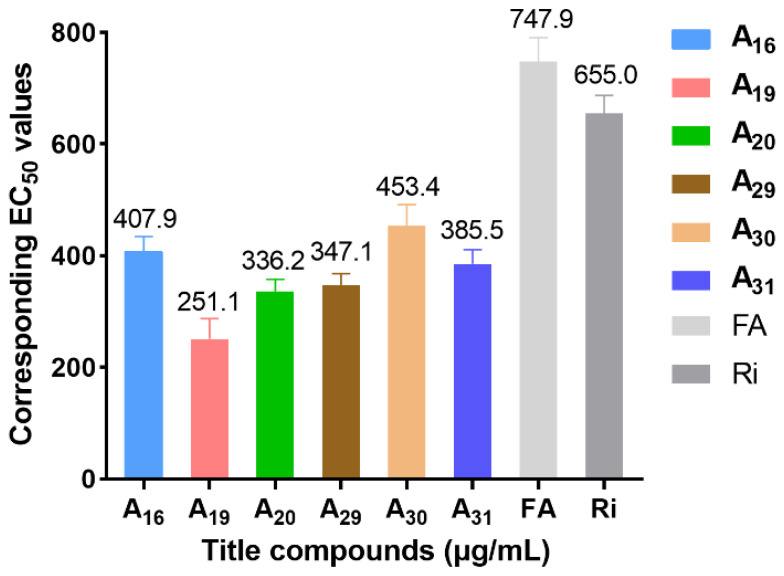
The EC_50_ values of curative activities of target compounds and Ri against TMV in vivo.

To explore the structural diversity of substituents based on piperidine structure and its influence on activity, different substituted piperazines were incorporated into the target structure under similar synthesis conditions (Figure 3). Bioassay results indicate that the ultimate activities (focused on curative effect) showed a marked divergence (Table 2). When the alkyl substituted piperazine was introduced to the molecule, the corresponding **A_20_** (R = 1-ethylpiperazinyl, 56.9%) and **A_21_** (R = 1-isopropylpiperazinyl, 45.2%) showed comparable antiviral potential to Ri at 500 μg/mL. This variation caused an increase in the hydrophobic property and sterically hindered the group of alkyl-substituted piperazine with slightly quenched antiviral activity. When the phenyl-substituted piperazine was constructed on the target structure, **A_22_** and **A_23_** (20.1% and 31.6% at 500 μg/mL) only showed weak efficacy, demonstrating that a rigid, steric, and unsaturated alkane directly substituted with nitrogen on piperazine was unfavorable for molecular bioactivity. On the other hand, the introduction of electron-donating groups at the *ortho* or *meta* position of the benzyl group in piperazine was beneficial to curative activity, e.g., **A_31_** (R = 2-methylbenzyl, 61.7%) > **A_30_** (R = 3-methylbenzyl, 51.1%) > **A_24_** (R = benzyl, 44.6%) at 500 μg/mL. Additionally, after introducing electron-withdrawing groups (-NO2, -F, -CI) at the *ortho* or *meta* position of the benzyl group in piperazine, the obtained **A_25_**–**A_28_** displayed moderate to weak activity (28.7–47.3%), suggesting that the electron-donating group located at the *ortho* or *meta* position of the benzyl group could block or scarcely contribute to target compound bioactivity. However, when introducing an electron-withdrawing group at the *para* position of the benzyl group, it could endow enhanced antiviral capacity, such as **A_29_** (R = 4-chlorobenzyl, 59.1%) > **A_24_** (R = benzyl, 44.6%) > **A_28_** (R = 3-chlorobenzyl, 41.8%) > **A_26_** (R = 2-fluorobenzyl, 28.7%) at 500 μg/mL. To accurately assess the antiviral efficacy of these compounds, typical compounds with moderate to strong primary screening activity were selected for different concentrations in the in vivo test (Figure 4), affording the corresponding EC_50_ values of 407.9 (**A_16_**), 251.1 (**A_19_**), 336.2 (**A_20_**), 347.1 (**A_29_**), 453.4 (**A_30_**), 385.5 (**A_31_**), 747.9 (FA), and 655.0 (Ri) μg/mL, which were generally consistent with the activity trend of the primary screening. Encouragingly, in contrast with parent compound FA, **A_19_** augmented about three-fold antiviral competence and conspicuousness surpassed that of commercial Ri. 

**Figure 5:**
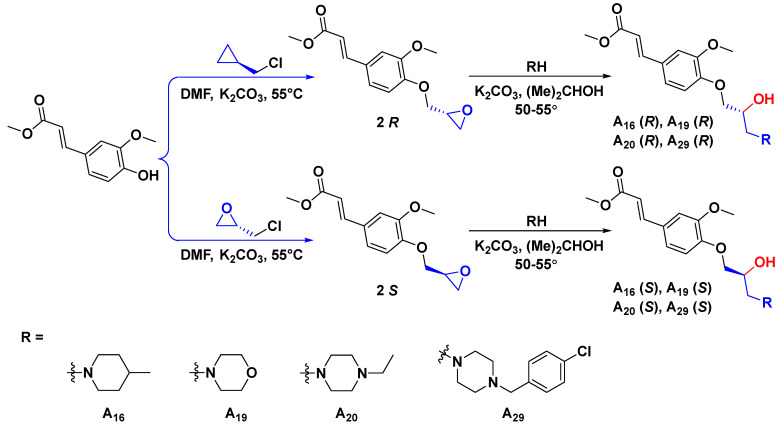
Synthetic routes for chiral target molecules.

**Figure 6:**
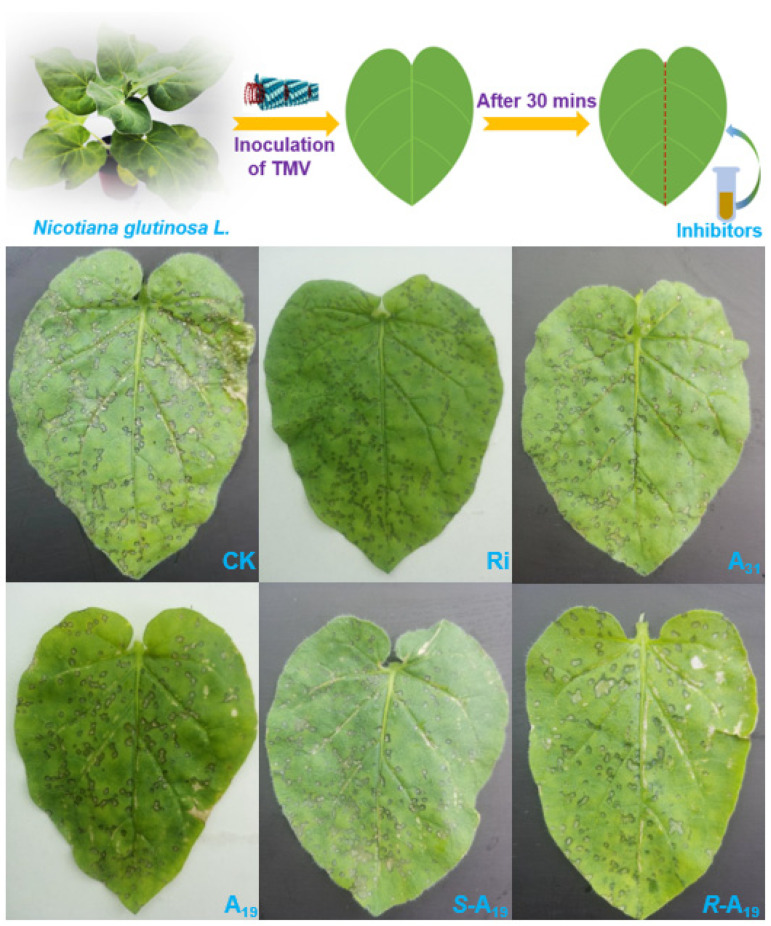
A schematic diagram of the classical half-leaf inoculation approach, and representative pictures of target compounds and the in vivo anti-TMV curative effect of ribavirin (Ri) at 500 μg/mL. The leaves were inoculated with 1% DMSO as a blank control (CK).

Since these compounds all have a unique chiral center, the differences in configuration may also often affect molecule biological activity in the host. As a result, to investigate the influence of absolute configuration of title molecules on overall antiviral activity or target receptor bioactivity, different antiviral levels of derivatives ***R***-**A_16_**, ***S***-**A_16_**, ***R***-**A_19_**, ***S***-**A_19_**, ***R***-**A_20_**, ***S***-**A_20_**, ***R***-**A_29_**, and ***S***-**A_29_** were synthesized (Figure 5). Their antiviral curative activities (Table 3) presented the following order: ***S***-forms (45.7%, ***S***-**A_16_**) < ***S***-forms (49.9%, ***S***-**A_20_**) < *R*-forms (55.1%, ***R***-**A_16_**) ≈ ***S***-forms (55.4%, ***S***-**A_19_**) < ***R***-forms (58.0%, ***R***-**A_20_**) < ***R***-forms (67.5%, ***R***-**A_19_**) at 500 μg/mL, suggesting that the *R*-forms of target compounds were more beneficial to aggrandize antiviral activity (Figure 6).

### 2.2. Circular Dichroism Spectroscopic Study

**Figure 7:**
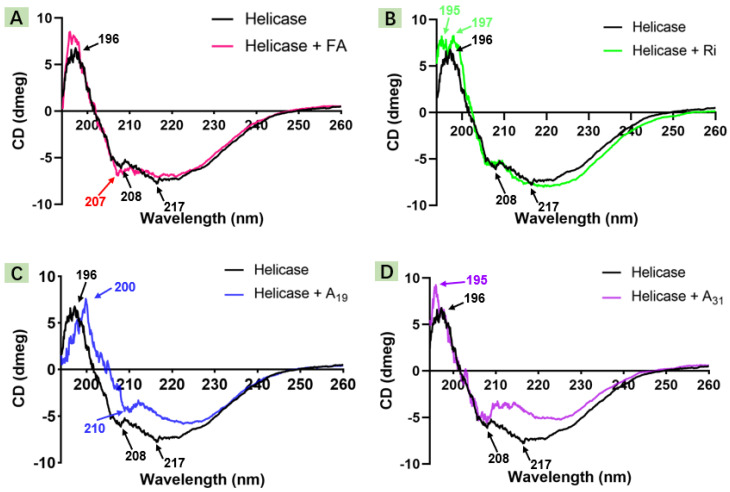
CD spectra changes in TMV helicase after with FA (**A**), Ri (**B**), **A_19_** (**C**), or **A_31_** (**D**), respectively.

Spectroscopic techniques have gained a wide reputation in the study of protein–ligand interactions [36,37,38]. Hence, in this bioassay, CD spectroscopy was engaged to identify the influence of the pattern of target compounds on the helicase secondary structure (190–260 nm). As seen in Figure 7, the purely helicase CD spectrum (black line) exhibited typical positive Cotton at 196 nm (*β*-sheet) and negative Cotton at 208 nm (*α*-helix) and 217 nm (*β*-sheet). However, after treated with FA or target compounds, the *α*-helix peak position of helicase was shifted, such as the FA-treated group (207 nm) and the **A_19_**-treated group (210 nm). Moreover, the *β*-sheet of helicase declined or disappeared, especially for the **A_19_**- or **A_31_**-treated group. These transitions may illustrate that the designed FA derivative had considerable capacity to bind to helicase and drive its secondary structure transfer.

### 2.3. Interaction Analysis

**Figure 8:**
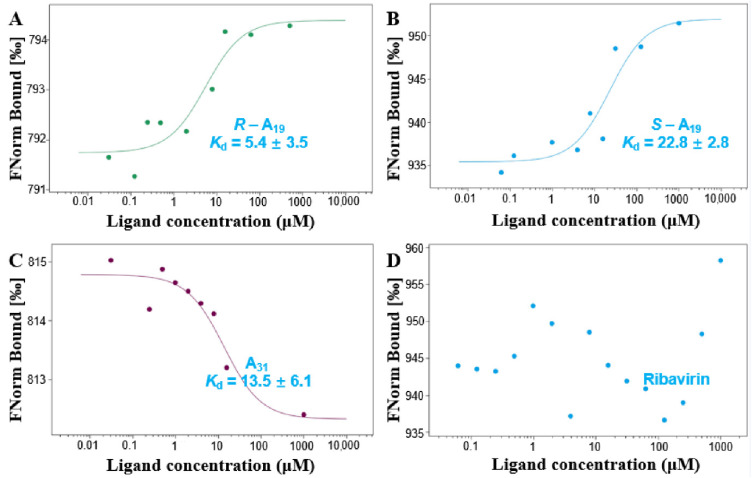
Interactions between compounds ***R***-**A_19_** (**A**), ***S***-**A_19_** (**B**), **A_31_** (**C**), or Ri (**D**) with helicase, measured by microscale thermophoresis. The inhibitory activity of target compounds and Ri against the TMV helicase at 200 μM.

Helicases represent a class of molecular motors with crucial roles in *gene* replication, transcription, translation, recombination, and repair. These features make it a potential molecular target for the drug design and mechanism of action studies [13,14]. To determine whether there was an evident interaction between the designed molecule and helicase, ***R***-**A_19_**, ***S***-**A_19_**, and **A_31_** were selected to interact with the TMV helicase for binding analysis. Besides exploring the influence of different substituents of FA on receptor affinity, it was also observed whether different chiral configurations had a significant influence on the interaction of helicase (Figure 8). The MST test results showed that ***R***-**A_19_** bound to helicase had a *K*_d_ value of 5.4 μM and ***S***-**A_19_** bound to helicase had a *K*_d_ value of 22.8 μM. Moreover, **A_31_** bound to helicase had a *K*_d_ value of 13.5 μM, while Ri had no apparent interaction with the TMV helicase. In contrast, ***R***-**A_19_** possessed the strongest binding ability to helicase. This result demonstrates that the *R* configuration may be more favorable for entering the active pocket of helicase and firmly interacting with the corresponding residues.

### 2.4. Inhibitory Activity of Helicase ATPase

**Figure 9:**
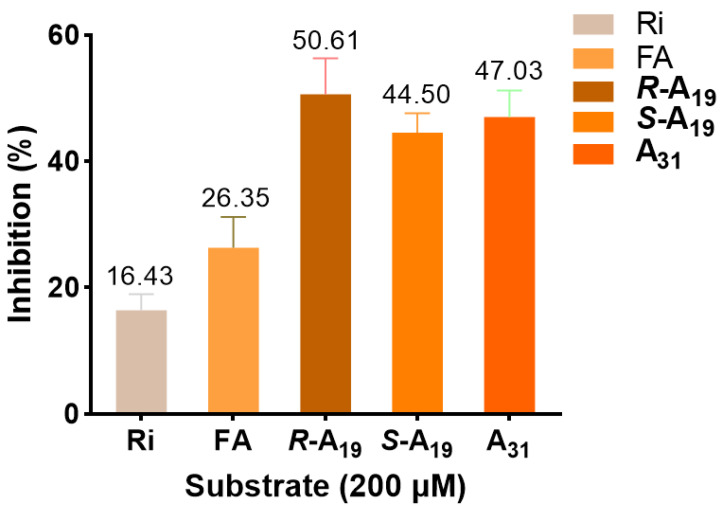
The inhibitory activity of target compounds and Ri against the TMV helicase at 200 μM.

Based on antiviral activity and MST consequences, ATPase activity assays were performed using ***R***-**A_19_**, ***S***-**A_19_**, and **A_31_** to explore the effect of different levels of antiviral activity and different chiral configurations of the compounds on helicase ATPase activity. As seen in Figure 9, compared with commercial Ri and FA, these target compounds empowered excellent blocking of TMV helicase ATPase activity. Illustrative examples are: ***R***-**A_19_** (50.61%) > **A_31_** (47.03%) > ***S***-**A_19_** (44.50%) > FA (26.35%) > Ri (16.43%) at 200 μM, which suggested that **A_19_** and **A_31_** had enhanced inhibitory effects targeting the TMV helicase, and the *R* configuration possessed stronger enzyme inhibition competence. The above results indicated that the designed FA-based inhibitors could restrain the ability of the helicase to hydrolyze ATP, thereby potentially interfering with its physiological function during virus replication. 

### 2.5. Docking Analysis

**Figure 10:**
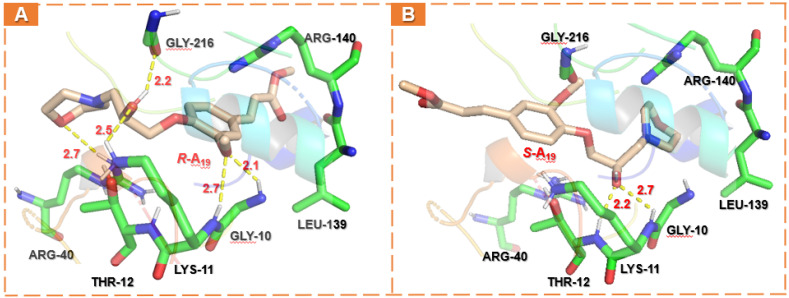
Molecular docking models of the TMV helicase with ***R***-**A_19_** (**A**) or ***S***-**A_19_** (**B**), respectively.

To profoundly understand the absolute configuration interaction (*R*-form or *S*-form) of **A_19_** on the TMV helicase, the receptor–ligand binding simulation was performed using SYBYL-X 2.0. As shown in Figure 10, the interactions of **A_19_** (***R***/***S***) and TMV helicase apparently occurred in the binding pocket defined by six residues (Gly10, Lys11, Thr12, Arg40, Leu139, Arg140, and Gly216). ***R***-**A_19_** displayed strong binding to helicase residues, including hydrogen–bond interactions with Gly10, Lys11, Thr12, Arg40, and Gly216 (2.1, 2.7, 2.5, 2.7, and 2.2 Å), polar interactions with Arg140, and hydrophobic interactions with Leu139 (Figure 10A). Comparably, the hydrogen bond of ***S***-**A_19_** with amino acid residues in the binding pocket was significantly reduced, mainly reflected in the morpholine ring and the methoxy group (Figure 10B), suggesting that the heteroatom of morpholine, the methoxy group of the benzene ring, and the hydroxyl group of the chiral carbon in *R* configuration were more conducive to forming hydrogen bonds in space. Moreover, docking results show that the C score value (the total scores of Surflex-Dock represent a -logK_d_ value) of ***R***-**A_19_** with the helicase (6.77) was higher than ***S***-**A_19_** (6.59). Collectively, the results of the molecular docking studies were basically consistent with the MST measurements, which could theoretically illustrate that ***R***-**A_19_** had higher helicase ATPase inhibitory activity compared to ***S***-**A_19_**.

### 2.6. Effect of Target Compounds on GFP-Labeled TMV and Relative Expression Levels of Helicase In Vivo

**Figure 11:**
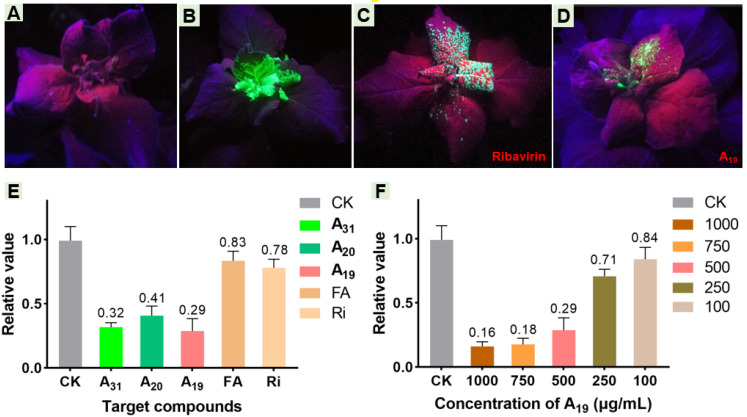
Inhibition effects of **A_19_** and Ri on the spread of TMV in *N. benthamiana:* leaves injected with 1% DMSO (**A**), TMV-GFP (**B**), TMV-GFP containing 0.25 mM Ri (**C**), or TMV-GFP containing 0.25 mM **A_19_** (**D**). Tested plants were photographed under long-wave UV light until 7 days after vaccination. The related transcript levels of the TMV helicase *gene* in *Nicotiana tabacum* cv. K326 treated with different target molecules (**E**) at 500 μg/mL, or different concentrations of **A_19_** (**F**).

As is vividly depicted in Figure 11, after 7 days, the plants inoculated with 1% DMSO could hardly observe green fluorescence under UV light in either the inoculated leaves or upper uninoculated leaves (Figure 11a). Inversely, when inoculated with TMV-GFP, the apical fresh leaves of plant showed nearly the entire area of fluorescence, intuitively showing that the virus had completely invaded the top of the tobacco (Figure 11b). Comparatively, the positive control (the Ri-treated group) showed obvious green fluorescence in the upper uninoculated leaves, along with a large-area diffused tendency on the newly top leaves (Figure 11c). However, when the plants were injected with TMV-GFP containing 0.25 mM **A_19_** (Figure 11d), green fluorescence was still visible but weak in the upper uninoculated leaves, and the distribution range was dramatically smaller than that of the positive control, which indicated that the virus only had a limited amount of expression in the host.

The relative quantitative *gene* expression of the TMV helicase in *Nicotiana tabacum* cv. K326 may indirectly shed light on the replication status of the virus in the host. To explore whether title compounds restrain the TMV helicase *gene*, different antiviral levels of **A_19_**, **A_20_**, and **A_31_** were used in pot experiments (500 μg/mL) and validated by RT-qPCR. Compared with the CK group, the relative expression of the TMV helicase *gene* in the **A_19_**-treated group, the **A_20_**-treated group, or the **A_31_**-treated group declined to 29%, 41%, or 32% within 3 days, respectively. Moreover, compared with the positive control (78%) or the FA-treated group (83%), these designed FA derivatives displayed an enhanced ability to down-regulate helicase *gene* relative expression in tobacco (Figure 11e). In addition, to further identify the inhibition of the helicase *gene* by **A_19_**, different concentrations of **A_19_** (100, 250, 500, 750, and 1000 μg/mL) were assessed. Subsequent results show a clear dose-dependent inhibition relationship between **A_19_** and the helicase *gene* from 100 to 750 μg/mL (18–84%). However, the relative expression of the TMV helicase *gene* barely changed when the concentration of **A_19_** was continuously raised from 750 μg/mL (Figure 11f). These results demonstrated that **A_19_** was able to repress the expression of the TMV helicase *gene*, disrupt helicase biosynthesis, and thereby delaying virus replication.

### 2.7. Computational Analysis

**Figure 12:**
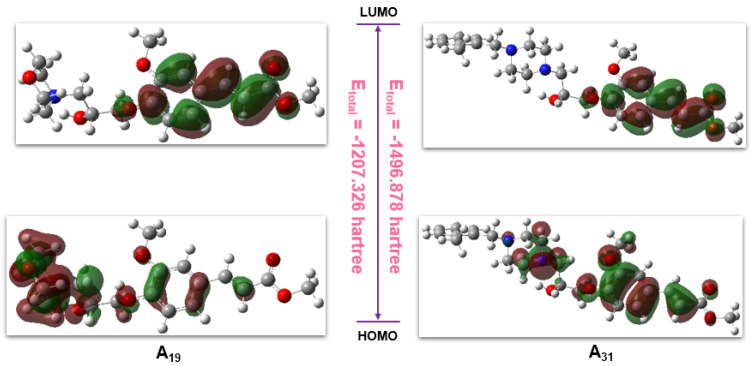
Frontier molecular orbitals of compounds **A_19_** and **A_31_**.

To reveal the skeleton structure characters of these family compounds and the potential surface variations resulting from different replacements, **A_19_** and **A_31_** were typically analyzed by DFT calculations with the B3LYP method (Gaussian view 6.0, 6-311G(d) and 6-311G(d, p) basis sets). The corresponding molecular total energy (MTE), the frontier molecular orbital (FMO) energy, the energy gap between the highest occupied molecular orbital (HOMO), the lowest unoccupied molecular orbital (LUMO), and the additional calculated parameters of **A_19_** and **A_31_** are listed in Table 4. In the light of frontier molecular orbital theory, MTE, HOMO, and LUMO play a predominant role in affecting pesticide physicochemical properties or binding with target receptors. Generally, HOMO could donate electrons, while LUMO could accept electrons, and lower pesticides in the HOMO−LUMO gap (ΔE) could result in higher antiphytopathogenic activity [39,40]. Figure 12 shows that the LUMOs of **A_19_** were primarily located on the FA skeleton, and the HOMOs of **A_19_** were mainly distributed on the benzene ring, the isopropanolamine bridge, and the morpholine ring fragments. Therefore, the total electron transition of **A_19_** was initiated from heterocyclic ring to the FA skeleton through the isopropanolamine bridge, and the total energy was −1207.326 hartree. For **A_31_**, the LUMOs were primarily located on the FA skeleton, and the HOMOs were mainly concentrated on the FA skeleton, the isopropanolamine bridge, and the piperazine ring. Hence, the total electron transition of **A_31_** was different from **A_19_**, and its total energy was lower (−1207.326 hartree). Moreover, **A_19_** exhibited a lower HOMO−LUMO gap (0.15 hartree) range than **A_31_** (0.152 hartree). These results suggested that **A_19_** may have a higher capacity to form ligand–receptor complexes, consistent with the enzyme activity assay described above. Energy calculations and electronic transitions provided a particularly meaningful perspective to distinguish and illustrate the different levels of the antiviral competence of **A_19_** and **A_31_**.

**Figure 13:**
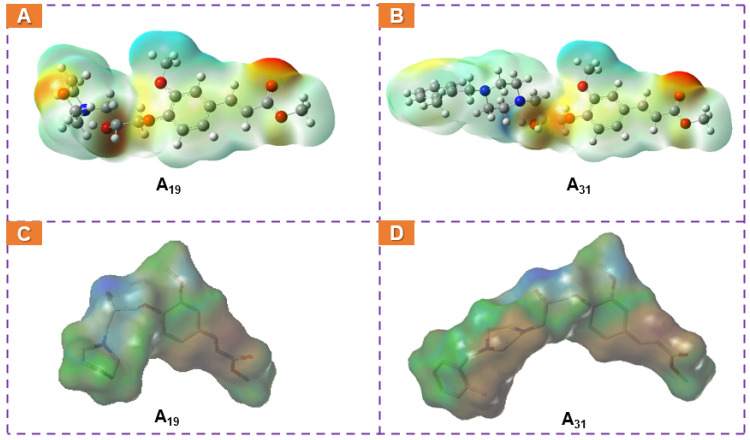
(**A**,**B**) ESP and (**C**,**D**) MLP of compounds **A_19_** and **A_31_**.

Molecular lipophilic potential (MLP) and electrostatic potential (ESP) have been wildly applied to identify H-bonding interactions or hydrophobic effects, visualize molecularly charged regions, and predict reactions or ligand–receptor interactions. Therefore, **A_19_** and **A_31_**, with different antiviral profile levels, were selected to perform MLP and ESP simulations. As depicted in Figure 13, the positively charged regions (blue) were mainly located in the FA ring and substituted in the heterocycle or phenyl group, while the negatively charged regions (red or yellow) were concentrated on the ester group and the methoxy group of the FA skeleton, the morpholine ring, as well as the hydroxyl group of the isopropanolamine bridge. These extremely charged structural regions may be considered to exhibit a significant role in binding to the target receptor. For **A_31_**, the domain of the positive charges on the substituent of 1-(2-methylbenzyl)piperazine ring displayed more positive and higher lipophilicity compared with **A_19_**, which presumably helped to hypothesize why **A_19_** had a stronger binding capacity than **A_19_**. These results were in accordance with the molecular docking analysis presented above.

### 2.8. Antibacterial Activity

To exploit the multiple potentialities of the target molecules, all synthesized derivatives were also evaluated for antibacterial activities, with the commercial agents bismerthiazol (BT) and thiodiazole copper (TC) as positive controls. Bioactivity screening shows (Table 5, Table 6 and Table 7) that most target compounds had weak antibacterial activity against *R. solanacearum*, except **A_1_** (70.5% at 100 μg/mL), which demonstrated comparable activity to the control agent TC (40.6% at 100 μg/mL). It is worth mentioning that a great number of target compounds showed moderate to strong antibacterial activity against *Xanthomonas oryzae pv oryzae*. Among them, **A_28_**, **A_29_**, and **A_30_** exhibited considerable antibacterial activities to suppress the growth of pathogenic bacteria. In order to further evaluate the antibacterial activity of the target compound against *Xoo*, we conducted an EC_50_ test for the compound whose activity exceeded 60% at 50 μg/mL. The results of bioassays indicate that compounds **A_28_** (12.65 μg/mL), **A_29_** (12.38 μg/mL), and **A_30_** (10.87 μg/mL) exhibited the best antibacterial activity compared to those commercial agents, i.e., BT (43.25 μg/mL) and TC (36.47 μg/mL). Furthermore, when the piperazine of the target compounds contained benzyl substitution, it was beneficial to improve the antibacterial activity, regardless of the introduction of electron-withdrawing groups or electron-donating groups on the aromatic ring. Similarly, the most synthesized FA derivatives exhibited better activity against *X. axonopodis pv citri.* The EC_50_ values of compounds **A_23_** (9.33 μg/mL) and **A_28_** (7.61 μg/mL) exhibited the best antibacterial activity which evidently exceeded TC (72.59 μg/mL), supporting the idea that the piperazine substituent group containing aromatic ring helped to improve the overall antibacterial activity of the target molecules. In view of the excellent activity of target molecules and the facile synthesis procedure, compounds **A_23_**, **A_28_**, **A_29_**, and **A_30_** may also become new leading compounds for the study of antibacterial activity.

## 3. Materials and Methods

### 3.1. Instruments and Chemicals

The melting points of the target products were measured using a WRX-4 micro-melting-point apparatus (Shanghai Yice Apparatus & Equipment Co., Ltd., Shanghai, China). ^1^H and ^13^C nuclear magnetic resonance (NMR) spectral analyses were performed on a JEOL-ECX 500 NMR spectrometer using CDCl_3_ or DMSO-*d6* as the solvent and tetramethylsilane (TMS) as an internal standard. High-resolution mass spectrometry (HRMS) was conducted on a LTQ Orbitrap (Thermo Scientific, Missouri, USA). The CD spectrum of helicase was performed through a JASCO J-1500 spectropolarimeter from JASCO (Tokyo, Japan) Co., Ltd. Binding studies were performed on a nanotemper monolith NT.115 instrument (Nanotemper, Munich, Germany) for microscale thermophoresis (MST). The ATPase activity experiment used enzyme assay reagent kits purchased from Sangon Biotech Co., Ltd. (Shanghai, China). All analytical reagents were used in the experiment obtained from Energy Chemical (Shanghai Saen Chemical Technology Co., Ltd., Shanghai, China), without further drying or purification.

### 3.2. General Procedures for Preparing Ferulic Acid Derivatives [41,42,43]

#### 3.2.1. General Procedures for Preparing Intermediate

Intermediate **1** was prepared using FA (10.0 g, 60 mmol) and methanol (50 mL) into a 150 mL three-necked flask equipped with a thermometer and a condenser, and concentrated sulfuric acid (60 mmol) was slowly added dropwise at room temperature. The temperature was raised to reflux and the reaction was completed after 8 h (indicated by thin-layer chromatography). Next, excess alcohol was removed under reduced pressure and enough saturated sodium bicarbonate solution was added to wash until no gas was generated. Afterwards, 30 mL of dichloromethane was added for extraction and dried with anhydrous sodium sulfate. Finally, in order to remove the solvent under vacuum, intermediate **1** was obtained. A yellow liquid, yield 82.1%; ^1^H NMR (400 MHz, CDCl_3_) δ 7.62 (d, *J* = 15.9 Hz, 1H, alkene-H), 7.06 (d, *J* = 8.2, 1.9 Hz, 1H), 7.01 (d, *J* = 1.8 Hz, 1H), 6.91 (d, *J* = 8.2 Hz, 1H), 6.33–6.24 (d, *J* = 9.0 Hz, 1H, alkene-H), 3.89 (s, 3H, O-CH_3_), 3.79 (s, 3H, O-CH_3_); ^13^C NMR (101 MHz, CDCl_3_) δ 167.9, 148.1, 146.9, 145.1, 126.8, 123.0, 114.9, 114.8, 109.4, 55.9, 51.6.

Intermediate **2** was prepared by adding intermediate **1** (10.0 mmol), potassium carbonate (11.0 mmol), and dimethylformamide to a 50 mL round bottom flask. After stirring at room temperature for 5 min, propylene oxide bromide (11.5 mmol) was added dropwise, and then the reaction was stopped at 60 °C for 8 h (indicated by thin-layer chromatography). The reaction mixture was diluted with dichloromethane and transferred to a 100 mL beaker. Subsequently, sodium hydroxide solution was added dropwise under an ice bath. After stirring for several minutes, dichloromethane (80 mL × 3) was added for extraction. The organic layers were combined and concentrated in vacuo. A white solid, yield 57.6%; m. p. 67.1–68.4 °C; ^1^H NMR (500 MHz, CDCl_3_) δ 7.58 (d, *J* = 15.9 Hz, 1H, alkene-H), 7.06–7.00 (m, 2H, Ar-H), 6.87 (d, *J* = 8.2 Hz, 1H, Ar-H), 6.28 (d, *J* = 15.9 Hz, 1H, alkene-H), 4.27 (dd, *J* = 11.4, 3.2 Hz, 1H, O-CH_2_), 4.00 (dd, *J* = 11.4, 5.7 Hz, 1H, O-CH_2_), 3.86 (s, 3H, O-CH_3_), 3.75 (s, 3H, O-CH_3_), 3.36 (m, *J* = 5.8, 4.1, 3.2, 2.8 Hz, 1H, O-CH), 2.88 (dd, *J* = 4.8, 4.2 Hz, 1H, O-CH_2_), 2.72 (dd, *J* = 4.9, 2.6 Hz, 1H, O-CH_2_); ^13^C NMR (126 MHz, CDCl_3_) δ 167.6, 150.0, 149.5, 144.6, 128.1, 122.3, 115.8, 113.2, 110.1, 69.9, 55.9, 51.7, 50.0, 44.8.

#### 3.2.2. General Synthetic Procedures for Title Compounds **A_1_**–**A_31_**

To prepare the target compound, intermediate **2** (1.0 mmol) was added in isopropanol (5 mL) containing heteroatom compounds (1.2 mmol) at 55 °C for an alkylation reaction lasting 16–24 h, and potassium carbonate (2.0 mmol) was added to the solution as well. The reaction progress was indicated by TLC. Then, the residue was washed with H_2_O (80 mL × 3) and extracted with CH_2_Cl_2_ (40 mL × 3). The organic layers were combined, dried over anhydrous MgSO_4_, filtered, and distilled under reduced pressure. After that, the crude product was further purified by flash column chromatography using CH_3_OH/CH_2_Cl_2_ (1:15–1:100) or hexane/EtOAc (10:1–1:1).

*3-methyl-(4-(2-hydroxy-3-((2-hydroxyethyl)amino)propoxy)-3methoxyphenyl)acrylate (**A_1_**)*

A yellow liquid, yield 63.2%; ^1^H NMR (500 MHz, CDCl_3_) δ 7.49 (d, *J* = 15.9 Hz, 1H, alkene-H), 6.94 (dd, *J* = 8.3, 1.9 Hz, 1H, Ar-H), 6.90 (d, *J* = 1.9 Hz, 1H, Ar-H), 6.77 (d, *J* = 8.3 Hz, 1H, Ar-H), 6.17 (d, *J* = 15.9 Hz, 1H, alkene-H), 3.96–3.92 (m, 1H, O-CH), 3.90 (d, *J* = 4.4 Hz, 2H, O-CH_2_), 3.74 (s, 3H, O-CH_3_), 3.65 (s, 3H, O-CH_3_), 2.55–2.47 (m, 3H, N-CH_2_&O-CH-CH_2_), 2.42 (dd, *J* = 11.7, 5.5 Hz, 2H, N-CH_2_), 0.89 (d, *J* = 7.1 Hz, 3H, CH-CH_3_); ^13^C NMR (126 MHz, CDCl_3_) δ 167.6, 150.5, 149.5, 144.7, 127.6, 122.4, 115.4, 112.9, 110.0, 71.7, 65.8, 55.8, 55.6, 51.6, 51.6, 47.1, 11.8; HRMS (ESI: *m*/*z* calculated for C_16_H_23_NO_6_^+^: 338.1962; found: 338.1966.

#### 3.2.3. General Synthetic Procedures for Title Compounds ***R***-**A_16_**, ***S***-**A_16_**, ***R***-**A_19_**, ***S***-**A_19_**, ***R***-**A_20_**, ***S***-**A_20_**, ***R***-**A_29_**, and ***S***-**A_29_**

For synthetic chiral compounds (***R***-**A_16_**, ***S***-**A_16_**, ***R***-**A_19_**, ***S***-**A_19_**, ***R***-**A_20_**, ***S***-**A_20_**, ***R***-**A_29_**, and ***S***-**A_29_**), chiral intermediate **2** was prepared by adding intermediate **1** (5.0 mmol), potassium carbonate (7.0 mmol), and dimethylformamide (5 mL) to a 25 mL round bottom flask. After stirring at room temperature for 5 min, chiral epichlorohydrin (7.5 mmol) was added dropwise, and then the reaction was stopped at 55 °C for 18 h (indicated by thin-layer chromatography). Other steps follow the previously mentioned method.

*S-3-methyl-(4-(2-hydroxy-3-(4-methylpiperidin-1-yl)propoxy)-3-methoxyphenyl)acrylate (**S**-**A_16_**)*

A yellow liquid, yield 64.1%; ^1^H NMR (400 MHz, CDCl_3_) δ 7.54 (dd, *J* = 15.9, 11.2 Hz, 1H, alkene-H), 7.03–6.94 (m, 2H, Ar-H), 6.83 (d, *J* = 8.3 Hz, 1H, Ar-H), 6.22 (dd, *J* = 15.9, 8.0 Hz, 1H, alkene-H), 4.17–4.08 (m, 1H, O-CH), 4.01–3.92 (m, 2H, O-CH_2_), 3.80 (s, 3H, O-CH_3_), 3.72 (s, 3H, O-CH_3_), 3.34–3.22 (m, 2H, N-CH_2_), 2.97 (d, *J* = 11.8 Hz, 1H, N-CH_2_), 2.84 (d, *J* = 12.0 Hz, 1H, N-CH_2_), 2.51 (d, *J* = 6.4 Hz, 2H, N-CH_2_), 2.26 (t, *J* = 10.3 Hz, 1H, CH_2_), 2.01 (t, *J* = 11.6 Hz, 1H, CH_2_), 1.58 (d, *J* = 12.6 Hz, 2H, CH_2_), 1.38–1.28 (m, 1H, CH), 0.86 (d, *J* = 6.2 Hz, 3H, CH_3_); ^13^C NMR (101 MHz, CDCl_3_) δ 166.9, 149.6, 148.9, 144.0, 127.1, 121.7, 114.9, 112.5, 109.5, 71.0, 64.6, 60.0, 55.1, 52.1, 50.8, 33.3, 29.6, 20.9; HRMS (ESI): *m*/*z* calculated for C_20_H_29_NO_5_^+^: 364.2118; found: 364.2103.

All the molecular structures were confirmed by nuclear magnetic resonance (NMR) spectroscopy and HRMS (Supplementary Materials).

### 3.3. Biological Assay

The antiviral activities against tobacco mosaic virus (TMV) and the antibacterial activities against *Ralstonia solanacearum* (*R. s.*), *Xanthomonas oryzae pv. oryzae* (*Xoo*), and *X. axonopodis pv. citri* (*Xac*) of all synthesized target compounds were tested by exerting a half-leaf inoculation approach and the classical turbidimetric method. All the detailed operations follow our group’s previously reported methods [44,45]. Measurements were performed in triplicate.

### 3.4. pPIC9K-HIS-TMV-Helicase Expression and Purification

The TMV helicase *gene* sequence was acquired from the NCBI database (GenBank: AF273221.1). The designed primers include the BamHI forward primer (5′-GGATCCGTTCTTGTGGACGGAGTT-3′) and the XhoI reverse primer (5′-CTCGAGCAGTGTAGTACTTGAGCGA-3′). The preparation of pPIC9K-HIS-TMV-helicase expression and purification was successful and identified by SDS-PAGE analysis [46]. For details, see Supporting Information (Figure S6).

### 3.5. Secondary Structural Analyzation

To identify the effect of title compounds upon target protein secondary structure, the TMV helicase (10.0 μg) was incubated with or without title compounds (5.0 μM) in a buffer solution (20 mM phosphate, 150 mM KCI, pH = 7.4) for 10 min. Subsequently, a JASCO J-1500 spectropolarimeter was used to detect the corresponding treated samples [36].

### 3.6. Binding Analysis between Antiviral Compounds and TMV Helicase

Microscale thermophoresis was performed on a Monolith NR 115 to compute the binding energy between the helicase and the target compound. The compound concentration was isocratically diluted with PBS (pH 7.2) from 2 mM to 0.1 mM in a different tube. Then, 10 μL of the 0.1 mM soluble labeled helicase was added into every diluted compound tube. After that, all tubes were incubated for 5 min and the samples were loaded into a special NanoTemper glass capillary. The LED power was modulated to 40% to perform microthermophoresis. The dissociation constant value (*K*_d_) was calculated from NanoTemper software [37].

### 3.7. TMV Helicase ATPase Activity

To validate the inhibition effect of TMV by target compounds on ATPase activity, a classical phospholipid malachite green assay was implemented by measuring the amount of Pi released in the mixed system. Hence, the commonly used phosphate buffer was replaced by deionized water for this assay. Firstly, the TMV helicase (10 μg) was incubated for 10 min without or with different ligands (20 μM), and centrifuged for 10 min (4000 g). Then, the supernatant (20 μL) was taken into the sample tube, and the phosphorus fixing reagent (200 μL) was added. After mixing and heating in a water bath (40 °C) for 10 min, the absorbance value of solution was recorded at 660 nm [38].

### 3.8. Homologous Modeling of TMV Helicase 3D Structure and Docking

The TMV helicase sequence (V829-T1085) was obtained from the protein NCBI database. Employing a sequence similarity search with BLAST in Protein Data Bank to find the ToMV helicase (PDB code: 3VKW) had a FASTA sequence identity (up to 90%) with the TMV helicase (Figure S1). Therefore, the TMV helicase homology model was built by SWISS-MODEL based on the crystal structure of the ToMV helicase (Figures S2–S5). All parameter settings used default values. The receptor protein was prepared by the SYBYL-X 2.0 software biopolymer module, according to the modeled TMV helicase structure. After that, the receptor protein was revised by adding lost residues and assigning Gasteiger–Huckel charges to the ligand and receptor. In addition, the lowest energy conformation was selected as the dominant conformation. Under the threshold and the expansion Bloat as the default values, the prototype molecules were generated and centered on the ligand ATP coordinates in the HEL-ATP complex. The docked structure could be scored and clustered by 0.8 Å of RMSD criteria. Other parameters used default values, and the results of ligand–receptor docking were shown in Table S1 [47,48].

### 3.9. Determination of Helicase Relative Gene Expression by RT-qPCR

The lower leaves of *Nicotiana tabacum* cv. K326 were selected to rub the virus juice with a brush after sprinkling the emery. Then, TMV-treated leaves were washed with distilled water after inoculating about 30 min and sprayed target compounds onto the leaves at different concentrations (1000, 750, 500, 250, and 100 μg/mL, respectively). After 3 days, the total RNA of sample leaves was extracted using a TransZol Up kit (TransGen Biotech, Beijing, China) and the reverse transcription assay was performed with a cDNA kit (TaKaRa, Dalian, China). The influence of compounds on TMV helicase *gene* relative expression was assessed using an iCycleriQ multicolor real-time PCR detection system (Bio-Rad, Hercules, California, USA). The relative level of *gene* expression was evaluated using the 2^−ΔΔCt^ method (actin *gene* as internal reference *gene*). Corresponding qRT-PCR primer sequences are presented in Table S2 [49]. Experimental data analysis was carried out by using GraphPad Prism (version 8).

### 3.10. Observation the Movement of GFP-Labeled TMV

The same batch of *Nicotiana benthamiana* in the seventh leaf stage, grown in a greenhouse, was selected for the pot experiment. Firstly, GFP-labeled TMV solution was inoculated onto plant leaves. After 30 min, different title compounds were smeared on the lower leaves at 500 μg/mL, respectively. Solvent (1% DMSO) and ribavirin were used as the controls. All the treated pots were then continued to be cultivated in the greenhouse. The GFP fluorescence intensity and distribution area of tobacco plants were observed with a portable UV lamp and the results were recorded on the seventh day [50].

### 3.11. DFT Calculation

The structures of target compounds with excellent antiviral profile were drawn with Gaussian view 6.0. The structural optimization, highest occupied molecular orbital (HOMO), lowest unoccupied molecular orbital (LUMO), and electrostatic potential (ESP) of compounds were calculated by DFT-B3LYP with 6-311G(d) and 6-311G(d,p) basis sets. All convergence accuracies were followed by system default values [39,40].

## 4. Conclusions

In summary, a series of novel FA derivatives incorporating substituted isopropanolamine moieties were designed and synthesized by targeting the TMV helicase. Structure–activity relationship research found that the introduction of benzylpiperazine, containing the electron-donating group (*ortho*) or the electron-withdrawing group (*para*), was favorable to empower therapeutic profiles, especially for **A_29_** (59.1%) and **A_31_** (61.7%) at 500 μg/mL. The absolute configuration research found that *R*-forms of target compounds were more beneficial to aggrandize antiviral abilities. Among them, optimized ***R***-**A_19_** exhibited excellent viral helicase ATPase inhibitory potency (50.61% at 200 μM), along with applausive antiviral abilities (EC_50_ = 251.1 μg/mL), about three-fold more than that of starting material FA and conspicuously surpassed commercial Ri. Additionally, **A_19_** could down-regulate the expression of the TMV helicase *gene* in the host to attenuate virus replication. The molecular docking model showed that ***R***-**A_19_** had stronger hydrophobic, polar, and hydrogen bonding interactions with the receptor residues in the binding pocket. Moreover, using DFT calculations, **A_19_**, with a higher total energy and a lower energy gap, was shown to be more feasible in presenting distinctive bioactivity. The above results provide fundamental support for the development of FA derivatives as potential plant viral helicase-based inhibitors for agricultural applications.

## Figures and Tables

**Table 1:** The in vivo anti-TMV activity of synthesized compounds (**A_1_**–**A_19_**).

Compound	Conc.	Inhibition Rate (%)	
	(μg/mL)	Curative Effect	Protective Effect
**A_1_**	500	32.7 ± 1.2	21.6 ± 0.2
	100	6.4 ± 0.8	0
**A_2_**	500	19.2 ± 0.7	0
	100	0	0
**A_3_**	500	38.6 ± 0.7	41.7 ± 2.6
	100	19.8 ± 3.1	15.4 ± 3.7
**A_4_**	500	43.8 ± 1.5	21.4 ± 4.0
	100	17.6 ± 1.0	0
**A_5_**	500	10.3 ± 3.9	23.1 ± 5.6
	100	0	0
**A_6_**	500	6.8 ± 4.0	16.8 ± 0.6
	100	0	0
**A_7_**	500	4.3 ± 2.3	47.7 ± 4.6
	100	0	21.6 ± 3.9
**A_8_**	500	7.0 ± 2.2	42.1 ± 2.7
	100	0	20.2 ± 3.4
**A_9_**	500	5.2 ± 2.9	11.7 ± 1.9
	100	0	0
**A_10_**	500	29.0 ± 2.1	0
	100	11.2 ± 2.6	0
**A_11_**	500	36.1 ± 4.3	54.2 ± 4.2
	100	17.1 ± 2.4	24.5 ± 2.2
**A_12_**	500	28.4 ± 3.0	25.4 ± 2.4
	100	0	0
**A_13_**	500	28.3 ± 0.3	32.7 ± 1.6
	100	5.2 ± 0.4	8.9 ± 3.5
**A_14_**	500	41.5 ± 3.1	19.4 ± 2.6
	100	7.1 ± 2.8	0
**A_15_**	500	43.1 ± 1.3	29.1 ± 2.7
	100	0	0
**A_16_**	500	46.5 ± 1.3	35.3 ± 3.6
	100	10.1 ± 2.7	10.2 ± 0.9
**A_17_**	500	21.7 ± 0.6	45.9 ± 1.7
	100	0	27.5 ± 1.6
**A_18_**	500	27.1 ± 2.4	57.8 ± 0.7
	100	0	32.1 ± 2.3
**A_19_**	500	62.7 ± 0.2	46.4 ± 0.4
	100	17.6 ± 1.9	13.5 ± 3.6
Ferulic acid	500	31.4 ± 3.3	43.5 ± 1.8
	100	8.0 ± 1.7	16.9 ± 4.5
Ribavirin	500	42.1 ± 1.4	39.5 ± 1.2
	100	11.7 ± 1.8	8.6 ± 2.1

**Table 2:** The in vivo anti-TMV activity of synthesized compounds (**A_20_**–**A_31_**).

Compound	Conc.	Inhibition Rate (%)	
	(μg/mL)	Curative Effect	Protective Effect
**A_20_**	500	56.9 ± 2.2	55.2 ± 3.0
	100	18.4 ± 1.7	20.1 ± 1.3
**A_21_**	500	45.2 ± 1.0	50.6 ± 1.2
	100	0	16.2 ± 2.5
**A_22_**	500	20.1 ± 1.3	7.9 ± 4.1
	100	0	0
**A_23_**	500	31.6 ± 4.9	15.7 ± 3.1
	100	9.1 ± 3.4	0
**A_24_**	500	44.6 ± 3.7	45.7 ± 4.2
	100	11.4 ± 0.6	19.4 ± 3.6
**A_25_**	500	47.3 ± 1.4	35.3 ± 1.0
	100	23.2 ± 1.5	12.4 ± 4.1
**A_26_**	500	28.7 ± 3.1	43.1 ± 2.5
	100	0	8.1 ± 2.3
**A_27_**	500	42.6 ± 1.3	60.8 ± 4.2
	100	0	31.6 ± 3.7
**A_28_**	500	41.8 ± 3.2	46.1 ± 2.1
	100	0	0
**A_29_**	500	59.1 ± 0.1	42.1 ± 2.9
	100	25.4 ± 2.5	5.3 ± 2.4
**A_30_**	500	51.1 ± 2.6	14.5 ± 0.8
	100	17.4 ± 0.1	0
**A_31_**	500	61.7 ± 3.3	24.9 ± 2.7
	100	27.1 ± 1.7	8.2 ± 0.9
Ferulic acid	500	31.4 ± 3.3	43.5 ± 1.8
	100	8.0 ± 1.7	16.9 ± 4.5
Ribavirin	500	42.1 ± 1.4	39.5 ± 1.2
	100	11.7 ± 1.8	8.6 ± 2.1

**Table 3:** The in vivo anti-TMV activity of ***R***-**A_16_** to ***S***–**A_29_**.

Compound	Conc.	Inhibition Rate (%)	
	(μg/mL)	Curative Effect	Protective Effect
**(*R*/*S*)-A_16_**	500	46.5 ± 1.3	35.3 ± 3.6
	100	10.1 ± 2.7	10.2 ± 0.9
***R*-A_16_**	500	55.1 ± 0.4	33.6 ± 1.5
	100	20.6 ± 3.1	8.5 ± 3.3
***S*-A_16_**	500	45.7 ± 0.9	15.7 ± 0.4
	100	16.8 ± 2.7	0
**(*R*/*S*)-A_19_**	500	62.7 ± 0.2	46.4 ± 0.4
	100	17.6 ± 1.9	13.5 ± 3.6
***R*-A_19_**	500	67.5 ± 2.3	44.9 ± 2.2
	100	26.3 ± 1.0	15.8 ± 1.4
***S*-A_19_**	500	55.4 ± 3.5	32.6 ± 1.7
	100	14.4 ± 1.2	8.4 ± 3.0
**(*R/S*)-A_20_**	500	56.9 ± 2.2	55.2 ± 3.0
	100	18.4 ± 1.7	20.1 ± 1.3
***R*-A_20_**	500	58.0 ± 3.9	46.7 ± 2.4
	100	24.8 ± 2.5	18.8 ± 2.8
***S*-A_20_**	500	49.9 ± 1.3	41.5 ± 1.7
	100	8.7 ± 2.4	17.9 ± 3.7
***(R/S)*-A_29_**	500	59.1 ± 0.1	42.1 ± 2.9
	100	25.4 ± 2.5	5.3 ± 2.4
***R*-A_29_**	500	56.7 ± 1.9	45.7 ± 4.1
	100	22.5 ± 1.4	0
***S*-A_29_**	500	45.1 ± 2.6	36.4 ± 2.6
	100	13.5 ± 4.2	0
Ferulic acid	500	31.4 ± 3.3	43.5 ± 1.8
	100	8.0 ± 1.7	16.9 ± 4.5
Ribavirin	500	42.1 ± 1.4	39.5 ± 1.2
	100	11.7 ± 1.8	8.6 ± 2.1

**Table 4:** The total energy, HOMO, LUMO, energy gap, C Log P, and TPSA of **A_19_** and **A_31_**.

Parameters	A_19_	A_31_
*E*total/hartree	−1207.326	−1496.878
*E*_HOMO_/hartree	−0.219	−0.219
*E*_LUMO_/hartree	−0.069	−0.067
△*E*/hartree	0.15	0.152
Clog *P*	1.386	4.118
TPSA	77.47	71.48

**Table 5:** In vitro antibacterial activities of target compounds against plant pathogen *R. s*.

Compound	Inhibition Rate (%)		Compound	Inhibition Rate (%)	
	100 μg/mL	50 μg/mL		100 μg/mL	50 μg/mL
**A_1_**	70.5 ± 4.0	46.6 ± 4.5	**A_19_**	0	0
**A_2_**	23.9 ± 0.7	0	***R*-A_19_**	7.4 ± 1.8	0
**A_3_**	0	0	***S*-A_19_**	0	0
**A_4_**	0	0	**A_20_**	0	0
**A_5_**	0	0	***R*-A_20_**	0	0
**A_6_**	0	0	***S*-A_20_**	0	0
**A_7_**	42.8 ± 2.7	22.1 ± 2.4	**A_21_**	58.4 ± 2.7	36.3 ± 3.6
**A_8_**	0	0	**A_22_**	41.4 ± 0.8	35.1 ± 5.3
**A_9_**	0	0	**A_23_**	0	0
**A_10_**	56.1 ± 2.3	23.3 ± 4.7	**A_24_**	12.2 ± 4.8	0
**A_11_**	30.2 ± 4.6	18.6 ± 0.4	**A_25_**	31.1 ± 2.6	0
**A_12_**	39.7 ± 4.2	24.3 ± 3.2	**A_26_**	37.1 ± 5.8	0
**A_13_**	0	0	**A_27_**	32.2 ± 3.9	0
**A_14_**	42.8 ± 5.0	29.8 ± 4.2	**A_28_**	29.8 ± 3.2	0
**A_15_**	0	0	**A_29_**	0	0
**A_16_**	46.4 ± 2.7	34.7 ± 3.9	***R*-A_29_**	9.1 ± 3.0	0
***R*-A_16_**	40.1 ± 2.6	28.8 ± 1.5	***S*-A_29_**	6.1 ± 1.0	0
***S*-A_16_**	26.7 ± 4.1	21.0 ± 4.3	**A_30_**	22.3 ± 4.8	0
**A_17_**	25.8 ± 1.1	17.0 ± 1.2	**A_31_**	38.5 ± 3.9	0
**A_18_**	7.3 ± 4.1	0	TC	40.6 ± 5.4	19.2 ± 0.1

**Table 6:** The in vitro antibacterial activities of target compounds against plant pathogen *Xoo*.

Compound	Inhibition Rate (%)		Toxic Regression Equation	EC_50_ (μg/mL)	r^2^
	100 μg/mL	50 μg/mL			
**A_1_**	45.5 ± 4.0	20.1 ± 1.7			
**A_2_**	29.0 ± 1.4	0			
**A_3_**	0	0			
**A_4_**	0	0			
**A_5_**	0	0			
**A_6_**	79.6 ± 1.2	0			
**A_7_**	27.3 ± 0.7	0			
**A_8_**	0	0			
**A_9_**	0	0			
**A_10_**	31.1 ± 5.6	11.6 ± 0.7			
**A_11_**	0	0			
**A_12_**	0	0			
**A_13_**	20.0 ± 0.7	0			
**A_14_**	0	0			
**A_15_**	33.4 ± 0.8	12.4 ± 4.8			
**A_16_**	0	0			
***R*-A_16_**	24.2 ± 2.8	0			
***S*-A_16_**	29.8 ± 3.1	0			
**A_17_**	68.2 ± 1.3	38.6 ± 5.8			
**A_18_**	69.0 ± 4.9	66.7 ± 1.7	y = 2.591x + 1.012	34.61 ± 0.64	0.995
**A_19_**	23.6 ± 0.6	9.2 ± 0.6			
***R*-A_19_**	0	0			
***S*-A_19_**	26.1 ± 3.8	17.3 ± 5.0			
**A_20_**	23.5 ± 1.2	13.1 ± 4.0			
***R*-A_20_**	0	0			
***S*-A_20_**	21.9 ± 0.7	0			
**A_21_**	0	0			
**A_22_**	60.6 ± 0.6	25.3 ± 2.2			
**A_23_**	100	100	y = 7.801x − 5.505	22.21 ± 0.43	0.941
**A_24_**	97.3 ± 0.3	84.9 ± 5.7	y = 3.543x + 0.014	25.54 ± 0.70	0.984
**A_25_**	91.8 ± 0.9	40.9 ± 4.6			
**A_26_**	97.9 ± 0.1	72.9 ± 1.9	y = 1.987x + 2.276	23.49 ± 1.30	0.999
**A_27_**	97.4 ± 0.1	44.7 ± 1.1			
**A_28_**	100	100	y = 5.312x − 0.855	12.65 ± 0.10	0.992
**A_29_**	100	100	y = 3.528x + 1.145	12.38 ± 0.72	0.915
***R*-A_29_**	100	100	y = 5.975x − 2.562	18.43 ± 0.08	0.812
***S*-A_29_**	100	100	y = 5.713x − 0.863	10.62 ± 0.37	0.994
**A_30_**	100	100	y = 2.629x + 2.275	10.87 ± 0.23	0.955
**A_31_**	100	41.1 ± 2.7			
TC	91.3 ± 2.5	60.9 ± 1.5	y = 1.098x + 0.835	36.47 ± 1.27	0.992

**Table 7:** The in vitro antibacterial activities of target compounds against plant pathogen *Xac*.

Compound	Inhibition Rate (%)		Toxic Regression Equation	EC_50_ (μg/mL)	r^2^
	100 μg/mL	50 μg/mL			
**A_1_**	14.3 ± 3.3	0			
**A_2_**	71.3 ± 2.7	44.0 ± 0.2			
**A_3_**	0	0			
**A_4_**	32.1 ± 1.3	0			
**A_5_**	15.3 ± 3.1	0			
**A_6_**	23.7 ± 4.4	21.8 ± 1.3			
**A_7_**	27.2 ± 4.3	0			
**A_8_**	17.4 ± 3.1	0			
**A_9_**	14.3 ± 1.7	0			
**A_10_**	100	94.7 ± 1.0	y = 3.267x − 0.289	41.58 ± 0.65	0.926
**A_11_**	0	0			
**A_12_**	86.7 ± 0.9	40.6 ± 3.0			
**A_13_**	20.1 ± 2.6	12.2 ± 2.2			
**A_14_**	46.8 ± 5.5	22.3 ± 4.3			
**A_15_**	47.8 ± 2.9	29.2 ± 2.0			
**A_16_**	68.5 ± 5.0	30.2 ± 3.2			
***R*-A_16_**	61.2 ± 2.6	35.5 ± 4.0			
***S*-A_16_**	64.7 ± 3.6	30.6 ± 3.1			
**A_17_**	0	0			
**A_18_**	31.0 ± 0.6	33.8 ± 2.9			
**A_19_**	23.9 ± 5.2	0			
***R*-A_19_**	25.4 ± 3.9	0			
***S*-A_19_**	20.1 ± 4.6	0			
**A_20_**	24.7 ± 2.0	11.4 ± 3.9			
***R*-A_20_**	28.1 ± 4.1	0			
***S*-A_20_**	22.5 ± 3.4	15.1 ± 2.8			
**A_21_**	28.4 ± 4.3	16.3 ± 3.6			
**A_22_**	59.1 ± 5.7	40.2 ± 5.7			
**A_23_**	100	95.1 ± 1.1	y = 1.354x + 3.687	9.33 ± 0.16	0.867
**A_24_**	100	100	y = 1.332x + 3.561	12.03 ± 0.19	0.954
**A_25_**	29.5 ± 1.3	19.2 ± 0.7			
**A_26_**	90.8 ± 0.8	78.2 ± 1.7	y = 2.041x + 2.630	14.49 ± 0.54	0.867
**A_27_**	86.7 ± 0.8	37.1 ± 2.6			
**A_28_**	100	83.2 ± 4.0	y = 1.622x + 3.570	7.61 ± 0.22	0.994
**A_29_**	100	100	y = 4.209x + 0.647	10.66 ± 0.16	0.982
***R*-A_29_**	100	100	y = 0.824x + 4.154	10.63 ± 0.51	0.983
***S*-A_29_**	100	96.4 ± 1.4	y = 1.939x + 3.076	9.82 ± 0.32	0.992
**A_30_**	100	79.1 ± 3.5	y = 1.564x + 3.202	14.11 ± 0.60	0.992
**A_31_**	92.8 ± 1.7	30.1 ± 3.0			
TC	56.1 ± 2.3	32.4 ± 2.3	y = 2.153x + 0.941	72.59 ± 2.73	0.962

## Data Availability

Not applicable.

## Supplementary Materials

The following supporting information can be downloaded at: https://www.mdpi.com/article/10.3390/ijms232213991/s1.

## Abbreviations

Ferulic acid	FA
Ribavirin	Ri
Tobacco mosaic virus	TMV
Circular dichroism	CD
Quantitative real-time polymerase chain reaction	RT-qPCR
Microscale thermophoresis	MST
Density functional theory	DFT
*Xanthomonas oryzae* pv. *oryzae*	*Xoo*
*Xanthomonas*. *axonopodis* pv *citri*	*X* *ac*
