# Supplementary material for: The Discovery of Novel Ferulic Acid Derivatives Incorporating Substituted Isopropanolamine Moieties as Potential Tobacco Mosaic Virus Helicase Inhibitors
Source: Int J Mol Sci. 2022 Nov 13;23(22):13991. doi: 10.3390/ijms232213991 (PMC9698358; doi:10.3390/ijms232213991)
Supplement: Supplementary file 1 [file ijms-23-13991-s001.zip › ijms-2010244-supplementary.pdf]

The Discovery of Novel Ferulic Acid Derivatives Incorporating Substituted Isopropanolamine Moieties as Potential Tobacco Mosaic Virus Helicase Inhibitors

Zhen-Xing Li, Bin-Xin Yang, Hong-Wu Liu, Yue Ding, Zi-Mian Fang, Wu-Bin Shao, Pu-Ying Qi, Xiang Zhou, Li-Wei Liu, and Song Yang *

State Key Laboratory Breeding Base of Green Pesticide and Agricultural Bioengineering, Key Laboratory of Green Pesticide and Agricultural Bioengineering, Ministry of Education, Center for R&D of Fine Chemicals of Guizhou University, Guiyang 550025, China.

* Corresponding author.
E-mail: jhzx.msm@gmail.com (YS).

1. Homology Modelling and Docking

The TMV helicase sequence was obtained from the protein NCBI database. A sequence similarity search with BLAST in the Protein Data Bank highlighted the ToMV helicase (PDB code: 3VKW, 1.9 Å resolution) as the most similar protein. The 3D structure of ToMV helicase was crystallized in its monomer conformation and it showed a FASTA sequence identity of 90% with the TMV helicase. The homology model was built by SWISS-MODEL when the crystal structure of the ToMV helicase was used as a template to imitate the TMV helicase's 3D structure based on counter sequences (V829-T1085). All other prime parameters were set to their default values (Figure S1). Model evaluation was performed by generating a Ramachandran plot (Figure S2).

Molecular dynamics (MDs) simulations were performed on the generated model of the TMV helicase in the absence of ATP. The ligands were parameterized with the antechamber module of Amber 12. The forcefield used to describe the protein parameters was the standard AMBER12 FF12SB forcefield for bio-organic systems. Before MD simulations, the explicit water model was used to optimize TMV helicase complexes, until the RMS of energy gradient fell below 0.5 kcal/mol/Å. The minimized structures were used as starting points for MD simulations. The MD simulations were performed at a constant temperature of 300 K. A time step of 2 fs was used for the simulation operation for 30 ns. The consequence of the 30th ns average structure was used as an initial structure to research the TMV helicase hydrolysis spot, named HEL-MD (Figure S3). By using Dali server to compare the HEL-MD structure with complexes from the Protein Data Bank, we found that the TMV helicase core had a similar structure similar to Upf1, UvrD, and Pif1. After overlapping crucial residues from HEL-MD with counterpart helicase containing ATP analogues by SYBYL-X Fit Monomers Function, the initial complexes of TMV HEL-ATP were obtained (Figure S4). To make HEL-ATP complexes rational, we used the explicit water model to optimize TMV helicase complexes in the FF12SB forcefield, until the RMS of the energy gradient fell below 0.5kcal/mol/Å. Molecular dynamics simulations were performed on the generated optimized model. The MD simulations were performed at

a constant temperature of 300 K, a time step of 2 fs was used for the 15 ns simulation operation, and the consequence of a 15th ns average structure was employed as a receptor protein for virtual screening and docking. The final representation structure of the TMV helicase was generated using PyMOL software (Figure S5).

```
Query                     1     VLVDGVPGCGKTKEILSRVNFDEDLILVPGKQAAEMIRRRANSSGIIVATKDNVKTVDSF    60
                                VLVDGVPGCGKTKEILS+VNF+EDLILVPGKQAAEMI+RRAN+SGII AT+DNV+TVDSF
A0A0S2T050 RDRP_TBRFV    829     VLVDGVPGCGKTKEILSKVNFEEDLILVPGKQAAEMIKRRANASGIIQATRDNVRTVDSF    888

Query                     61    MMNFGKSTRCQFKRLFIDEGLMLHTGCVNFLVAMSLCEIAYVYGDTQQIPYINRVSGFPY    120
                                +MN+GK TRCQFKRLFIDEGLMLHTGCVNFLV+MSLCEIAYVYGDTQQIPYINRVSGFPY
A0A0S2T050 RDRP_TBRFV    889     IMNYGKGTRCQFKRLFIDEGLMLHTGCVNFLVSMSLCEIAYVYGDTQQIPYINRVSGFPY    948

Query                     121   PAHFAKLEVDEVETRRTTLRCPADVTHYLNRRYEGFVMSTSSVKKSVSQEMVGGAAVINP    180
                                PAHFAK+EVDEVETRRTTLRCPAD+THYLNRRYEG+VM TSSVKKSVSQEMV GAA+INP
A0A0S2T050 RDRP_TBRFV    949     PAHFAKIEVDEVETRRTTLRCPADITHYLNRRYEGYVMCTSSVKKSVSQEMVSGAAMINP    1008

Query                     181   ISKPLHGKILTFTQSDKEALLSRGYSDVHTVHEVQGETYSDVSLVRLTPTPVSIIAGDSP    240
                                +SKPL+GK+LTFTQSDKEALLSRGY+DVHTVHEVQGETY+DVSLVRLTPTPVSIIAGDSP
A0A0S2T050 RDRP_TBRFV    1009    VSKPLNGKVLTFTQSDKEALLSRGYTDVHTVHEVQGETYADVSLVRLTPTPVSIIAGDSP    1068

Query                     241   HVLVALSRHTCSLKYYT                                               257
                                HVLVALSRHT +LKYYT
A0A0S2T050 RDRP_TBRFV    1069    HVLVALSRHTQTLKYYT                                               1085
```

Figure S1. FASTA sequence alignment, TMV helicase, and ToMV helicase (PDB：3VKW).

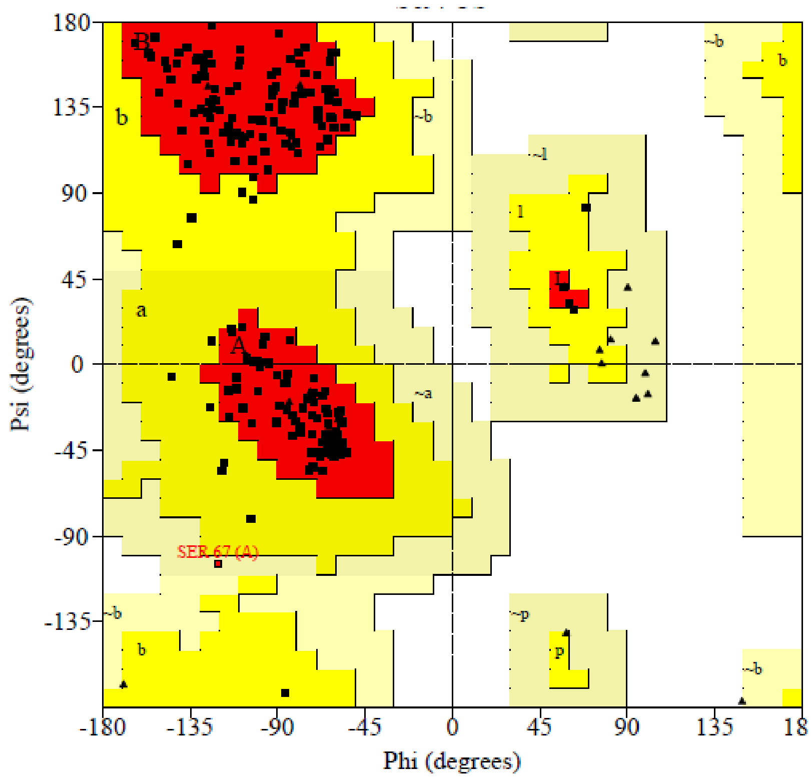

Figure S2. Ramachandran plot of the homology-modeled structure of the TMV helicase close conformation. The different colored areas indicate “disallowed” (white), “allowed” (yellow), and “most favored” (red) regions.

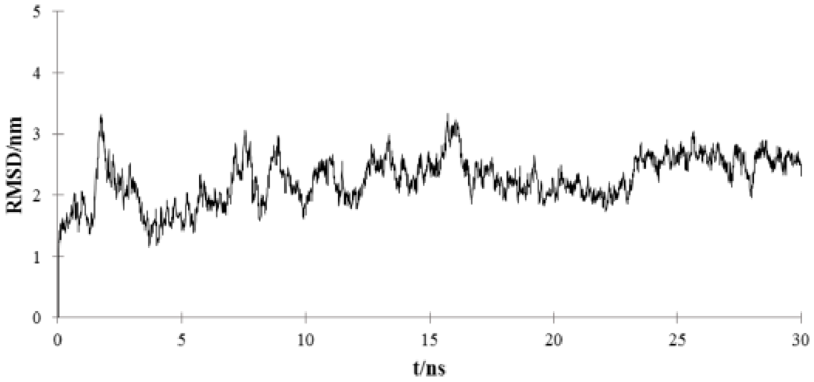

Figure S3. The RMSD-t relationship of the TMV helicase from the initial position during 30 ns.

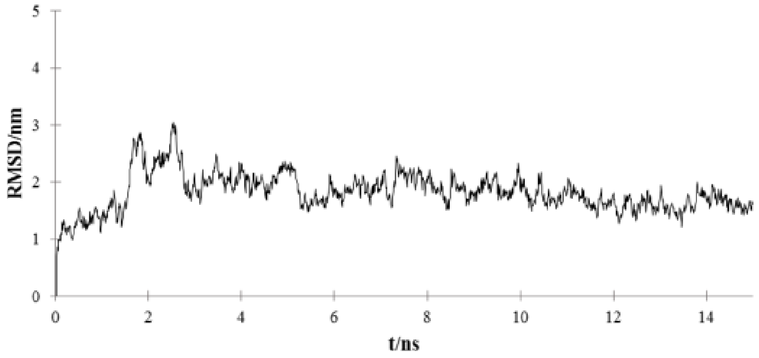

Figure S4. The RMSD-t relationship of the helicase–ATP complex from the initial position during 15 ns.

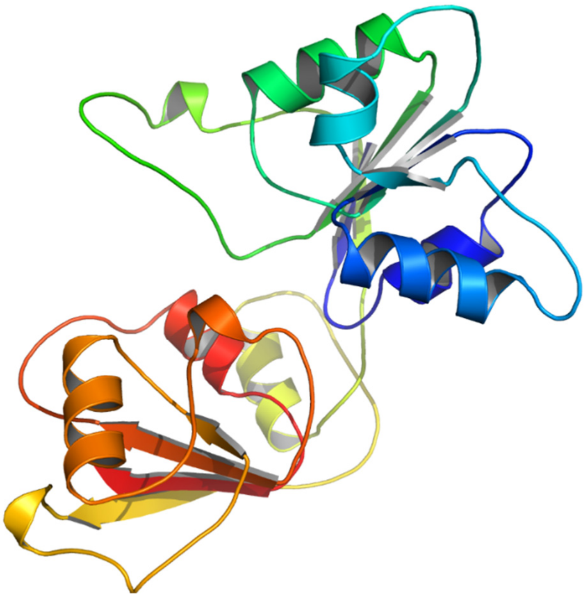

Figure S5. The structure of TMV helicase (homology modeling by the ToMV helicase 3D structure).

In order to explore the mechanism of action of the target compounds on helicase, ***R*-A19**, ***S*-A19**, and **A31** were selected for docking simulations. The specific calculation parameters are shown in Table S1.

Table S1. Surflex-Dock results of target molecules with the TMV helicase.

Compd	Total_Score	Crash	Polar	D_Score	PMF_Score	G_Score
Ri	3.89	-0.66	4.39	-48.799	-65.164	-128.984
***R*-A19**	6.77	-1.38	2.18	-112.759	-28.484	-156.579
***S*-A19**	6.59	-1.68	3.99	-116.018	-44.569	-137.726
**A31**	7.22	-1.59	2.32	-129.875	-4.365	-209.15

2. pPIC9K-HIS-TMV-Hel expression and purification

The gene sequence of the TMV helicase was acquired from the NCBI database (GenBank: AF273221.1). The designed primers include the *Bam*HI forward primer (5'-GGATCCGTTCTTGTGGACGGAGTT-3') and the *Xho*I reverse primer (5'-CTCGAGCAGTGTAGTACTTGAGCGA-3'). To obtain the pure helicase for biochemical analyses, we inoculated the plasmid pPIC9K-HIS-TMV-Hel recombinant E. coli bacterial solution in the 3 mL LB medium (Amp concentration 1 mg/mL, Kan concentration 50 μg/mL) and cultivated it at 37 °C overnight. The plasmid was extracted after the bacterial solution was taken out and the purity of the plasmid was measured by an ultraviolet spectrophotometer ($OD_{260/280}$ = 1.8). In the situation that plasmid concentration was above 1 μg/μL, GS115 competent cells were transformed with Eppendorf electroporation (parameters were: 1500 V; 5 ms), and the OD_{600} value of GS115 was controlled at 0.8. After that, GS115 was coated on RDB plates. Twenty single colonies were picked out for screening after 4 days and identified by PCR. This result indicates that most of them were transferred to the host. Subsequently, we inoculated 10 μL of the bacterial solution into 10 ml of the YPG liquid culture. After 24 hours, the culture solution became milky white, and then 10 ml of the culture solution was inoculated into the 1 L YPG medium (inoculation amount 1%, 30 °C, 230 rpm). After about 24 hours, the OD_{600} value of the culture solution was measured, and the YPG medium was used as the control. When the OD_{600} value of the bacteria after

being diluted 50 times reached 0.6-0.7, the culture solution was poured into two sterilized and cooled 500 mL centrifuge tubes for centrifugation (6000 rpm, 4 °C, 5 min). Then, the supernatant was discarded, and a 900 mL YP medium was used to resuspend the bacteria. The resuspension system was returned to a 5 L Erlenmeyer flask, and 100 mL of the 1 M phosphate buffer solution and 10 × 2 mL of anhydrous methanol were added to start the induction process. About 48 hours later, the culture solution was poured into two 500 mL centrifuge tubes for centrifugation (6,000 rpm, 4 °C, 10 min) and the supernatant was collected. Finally, the crude protein was purified by nickel column affinity chromatography and eluted with 250 mM imidazole (Figure S6).

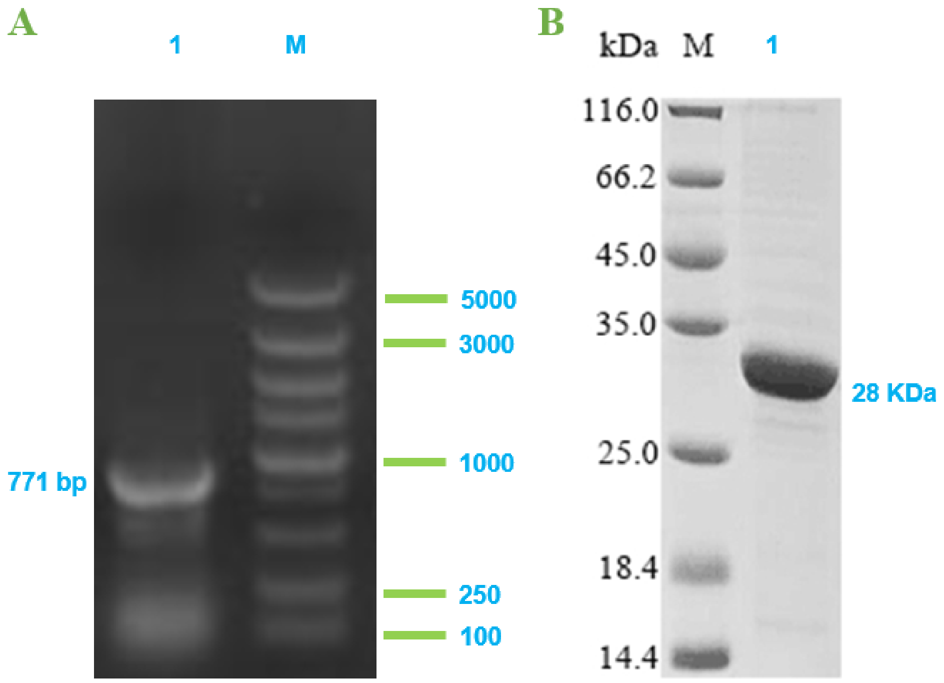

Figure S6. The construction of TMV-Hel in pPIC9K plasmid. (A) The amplified PCR and 1% agarose gel electrophoresis results show that helicase-length segments reached 771 bp. M was the DNA marker, and 1 was the positive monoclonal of pPIC9K-HIS-TMV-Hel with 771 bp. The characterization of TMV helicase. (B) M was the protein marker, and the helicase protein was purified by 12% SDS-PAGE analysis with 28 kDa in lane 1.

3. qPCR Analysis

To determine whether these FA derivatives had a regulatory effect on the genes

related to helicase, the target molecules with higher helicase ATPase inhibitory activity were selected for in vivo experiments in *Nicotiana tabacum* cv. K326, and the corresponding qRT-PCR primer sequences are presented in Table S2.

Table S2. Primer sequences of related helicase genes for RT-qPCR.

Gene	Forward primer	Reverse primer
AIL	CCAATCGGATAAAGAAGC	ATGGAGACTGGTGTAGGG
Actin	AAGGGATGCGAGGATGGA	CAAGGAAATCACCGCTTTGG

4. Characterization data of intermediates and target compounds

(*E*)-methyl-3-(3-methoxy-4-(oxiran-2-ylmethoxy)phenyl)acrylate (intermediate 2)

^{1}H NMR (500 MHz, CHLOROFORM-D) δ 7.58 (d, *J* = 15.9 Hz, 1H, alkene-H), 7.06 – 7.00 (m, 2H, Ar-H), 6.87 (d, *J* = 8.2 Hz, 1H, Ar-H), 6.28 (d, *J* = 15.9 Hz, 1H, alkene-H), 4.27 (dd, *J* = 11.4, 3.2 Hz, 1H, O-CH_2), 4.00 (dd, *J* = 11.4, 5.7 Hz, 1H, O-CH_2), 3.86 (s, 3H, O-CH_3), 3.75 (s, 3H, O-CH_3), 3.36 (m, *J* = 5.8, 4.1, 3.2, 2.8 Hz, 1H, O-CH), 2.88 (dd, *J* = 4.8, 4.2 Hz, 1H, O-CH_2), 2.72 (dd, *J* = 4.9, 2.6 Hz, 1H, O-CH_2); ^{13}C NMR (126 MHz, CHLOROFORM-D) δ 167.6, 150.0, 149.5, 144.6, 128.1, 122.3, 115.8, 113.2, 110.1, 69.9, 55.9, 51.7, 50.0, 44.8.

3-methyl-(4-(2-hydroxy-3-((2-hydroxyethyl)amino)propoxy)-3-methoxyphenyl)acrylate (A_1)

A yellow liquid, yield 63.2%; ^{1}H NMR (500 MHz, CHLOROFORM-D) δ 7.49 (d, *J* = 15.9 Hz, 1H, alkene-H), 6.94 (dd, *J* = 8.3, 1.9 Hz, 1H, Ar-H), 6.90 (d, *J* = 1.9 Hz, 1H, Ar-H), 6.77 (d, *J* = 8.3 Hz, 1H, Ar-H), 6.17 (d, *J* = 15.9 Hz, 1H, alkene-H), 3.96 – 3.92 (m, 1H, O-CH), 3.90 (d, *J* = 4.4 Hz, 2H, O-CH_2), 3.74 (s, 3H, O-CH_3), 3.65 (s, 3H, O-CH_3), 2.55 – 2.47 (m, 3H, N-C$\underline{H_2}$ & O-C$\underline{H}$-CH_2), 2.42 (dd, *J* = 11.7, 5.5 Hz, 2H, N-CH_2), 0.89 (d, *J* = 7.1 Hz, 3H, CH-C$\underline{H_3}$); ^{13}C NMR (126 MHz, CHLOROFORM-D) δ 167.6, 150.5, 149.5, 144.7, 127.6, 122.4, 115.4, 112.9, 110.0, 71.7, 65.8, 55.8, 55.6, 51.6, 51.6, 47.1, 11.8; HRMS (ESI): m/z calculated for $C_{16}H_{23}NO_6^+$: 338.1962; found: 338.1966.

3-methyl-(4-(3-((4-chlorophenyl)amino)-2-hydroxypropoxy)-3-methoxyphenyl)acrylate (A_2)

A yellow solid, yield 68.1%, m. p. 108.2-110.4 ℃; ^{1}H NMR (500 MHz, CHLOROFORM-D) δ 7.64 – 7.56 (m, 1H, alkene-H), 7.11 – 7.06 (m, 2H, Ar-H), 7.04 (dd, *J* = 8.4, 2.0 Hz, 1H, Ar-H), 7.01 (d, *J* = 2.0 Hz, 1H, Ar-H), 6.82 (dd, *J* = 8.1, 2.7 Hz, 1H, Ar-H), 6.59 – 6.50 (m, 2H, Ar-H), 6.30 (d, *J* = 16.0 Hz, 1H, alkene-H), 4.26 – 4.22 (m, 1H, O-CH), 4.09 (dd, *J* = 9.6, 3.8 Hz, 1H, O-CH_2), 4.03 (dd, *J* = 9.6, 6.3 Hz, 1H, O-CH_2), 3.85 (s, 3H, O-CH_3), 3.78 (s, 3H, O-CH_3), 3.36 (dd, *J* = 12.8, 4.4 Hz, 1H, N-CH_2), 3.22 (dd, *J* = 12.8, 6.6 Hz, 1H, N-CH_2); ^{13}C NMR (126 MHz, CHLOROFORM-D) δ 167.7, 149.9, 149.4, 146.8, 144.6, 129.1, 128.2, 122.5, 122.3, 115.9, 114.3, 113.3, 110.1, 77.4, 77.1, 76.9, 71.9, 68.3, 55.8, 51.7, 46.6; HRMS (ESI): m/z calculated for $C_{20}H_{22}ClNO_5^+$: 392.1259; found: 392.1244.

3-methyl-(4-(2-hydroxy-3-((3-phenoxyphenyl)amino)propoxy)-3-methoxyphenyl)acrylate (A_3)

A yellow liquid, yield 55.8%; ^{1}H NMR (500 MHz, CHLOROFORM-D) δ 7.63 (d, *J* = 16.0 Hz, 1H, alkene-H), 7.36 – 7.29 (m, 1H, Ar-H), 7.15 – 7.07 (m, 2H, Ar-H), 7.03 (dd, *J* = 10.6, 2.9 Hz, 2H, Ar-H), 6.87 (d, *J* = 8.3 Hz, 1H, Ar-H), 6.42 (dd, *J* = 8.8, 1.5 Hz, 1H, Ar-H), 6.37 (dd, *J* = 6.9, 1.9 Hz, 1H, Ar-H), 6.33 (dd, *J* = 9.1, 6.8 Hz, 1H, alkene -H), 4.37 (s, 1H, O-H), 4.31 – 4.25 (m, 1H, O-CH), 4.15 (dd, *J* = 9.7, 3.6 Hz, 1H, O-CH_2), 4.07 (dd, *J* = 9.7, 6.3 Hz, 1H, O-CH_2), 3.86 (s, 3H, O-CH_3), 3.81 (s, 3H, O-CH_3), 3.41 (s, 1H, N-H), 3.30 (d, *J* = 5.2 Hz, 2H, N-CH_2); ^{13}C NMR (126 MHz, CHLOROFORM-D) δ 167.7, 158.5, 157.3, 150.0, 149.9, 149.6, 144.7, 130.4, 129.7, 128.3, 123.1, 122.5, 119.0, 116.0, 113.5, 110.1, 108.5, 108.3, 103.8, 72.2, 68.4, 55.8, 51.8, 46.5; HRMS (ESI): m/z calculated for $C_{26}H_{27}NO_6^+$: 450.1911; found: 450.1908.

3-methyl-(4-(3-((4-(4-chlorophenoxy)phenyl)amino)-2-hydroxypropoxy)-3-methoxyphenyl)acrylate (A_4)

A yellow liquid, yield 48.6%; ^{1}H NMR (500 MHz, CHLOROFORM-D) δ 7.62 (d, *J* = 15.9 Hz, 1H, alkene-H), 7.23 – 7.18 (m, 2H, Ar-H), 7.05 (dd, *J* = 15.3, 5.1 Hz, 2H, Ar-H), 6.90 – 6.86 (m, 3H, Ar-H), 6.85 – 6.80 (m, 2H, Ar-H), 6.66 (d, *J* = 8.9 Hz, 2H,

Ar-H), 6.31 (d, *J* = 16.0 Hz, 1H, alkene-H), 4.29 (dd, *J* = 7.1, 3.0 Hz, 1H, O-CH), 4.16 (dd, *J* = 9.7, 3.7 Hz, 1H, O-CH_2), 4.09 (dd, *J* = 9.6, 6.4 Hz, 1H, O-CH_2), 3.88 (s, 3H, O-CH_3), 3.79 (s, 3H, O-CH_3), 3.42 (dd, *J* = 12.7, 4.5 Hz, 1H, O-CH_2), 3.28 (dd, *J* = 12.7, 6.5 Hz, 1H, O-CH_2); ^{13}C NMR (126 MHz, CHLOROFORM-D) δ 167.7, 157.7, 150.0, 149.6, 147.8, 145.9, 144.6, 129.5, 128.4, 126.9, 122.5, 121.3, 118.4, 116.1, 114.4, 113.6, 110.2, 72.2, 68.5, 55.9, 51.8, 47.1; HRMS (ESI): m/z calculated for $C_{26}H_{26}ClNO_6^+$: 484.1521; found: 484.1561.

3-methyl-(4-(2-hydroxy-3-methoxypropoxy)-3-methoxyphenyl)acrylate (A_5)

A white solid, yield 70.6%, m. p. 42.1-44.2 ℃; ^{1}H NMR (500 MHz, CHLOROFORM-D) δ 7.59 (d, *J* = 16.0 Hz, 1H, alkene-H), 7.04 (dd, *J* = 8.4, 2.0 Hz, 1H, Ar-H), 7.00 (d, *J* = 2.0 Hz, 1H, Ar-H), 6.87 (d, *J* = 8.3 Hz, 1H, Ar-H), 6.28 (d, *J* = 16.0 Hz, 1H, alkene-H), 4.21 – 4.15 (m, 1H, O-CH), 4.05 (m, 2H, O-CH_2), 3.84 (s, 3H, O-CH_3), 3.76 (s, 3H, O-CH_3), 3.53 (t, *J* = 5.3 Hz, 2H, O-CH_2), 3.37 (s, 1H, O-CH_3); ^{13}C NMR (126 MHz, CHLOROFORM-D) δ 158.1, 144.2, 143.7, 139.8, 126.4, 122.0, 116.6, 114.8, 112.2, 82.7, 80.6, 79.1, 71.4, 68.7, 65.3; HRMS (ESI): m/z calculated for $C_{15}H_{20}O_6^+$: 297.1332; found: 297.1322.

3-methyl-(4-(3-(2-fluorophenoxy)-2-hydroxypropoxy)-3-methoxyphenyl)acrylate (A_6)

A white solid, yield 73.6%, m. p. 77.5-79.3 ℃; ^{1}H NMR (500 MHz, CHLOROFORM-D) δ 7.59 (d, *J* = 15.9 Hz, 1H, alkene-H), 7.07 – 6.95 (m, 5H, Ar-H), 6.91 – 6.85 (m, 2H, Ar-H), 6.29 (d, *J* = 15.9 Hz, 1H, alkene-H), 4.46 – 4.40 (m, 1H, O-CH), 4.26 – 4.16 (m, 4H, O-CH_2), 3.83 (s, 3H, O-CH_3), 3.77 (s, 3H, O-CH_3); ^{13}C NMR (126 MHz, CHLOROFORM-D) δ 167.6, 152.6 (d, $^1J_{C\text{-}F}$ = 245.8 Hz), 150.0, 149.5, 146.5 (d, $^3J_{C\text{-}F}$ = 10.6 Hz), 128.1, 124.3 (d, $^4J_{C\text{-}F}$ = 3.7 Hz), 122.4, 121.7, 121.6, 116.2 (d, $^2J_{C\text{-}F}$ = 18.1 Hz), 115.8, 115.2, 113.5, 110.2, 77.4, 77.1, 76.9, 70.2, 70.0, 68.5, 55.8, 51.6. ^{19}F NMR (471 MHz, CHLOROFORM-D) δ -89.96; HRMS (ESI): m/z calculated for $C_{20}H_{21}FO_6^+$: 377.1394; found: 377.1378.

3-methyl-(4-(3-(3,4-dimethoxyphenoxy)-2-hydroxypropoxy)-3 methoxyphenyl)acrylate (A_7)

A white solid, yield 68.7%, m. p. 52.3-54.5 ℃; ^{1}H NMR (400 MHz, $CDCl_3$) δ 7.63 (d, *J* = 15.9 Hz, 1H, alkene-H), 7.08 (dd, *J* = 8.3, 1.7 Hz, 1H, Ar-H), 7.04 (d, *J* = 1.7 Hz, 1H, Ar-H), 6.93 (d, *J* = 8.3 Hz, 1H, Ar-H), 6.77 (d, *J* = 8.8 Hz, 1H, Ar-H), 6.54 (d, *J* = 2.8 Hz, 1H, Ar-H), 6.42 (dd, *J* = 8.7, 2.8 Hz, 1H, Ar-H), 6.32 (d, *J* = 15.9 Hz, 1H, alkene-H), 4.42 (dt, *J* = 10.4, 5.2 Hz, 1H, O-CH), 4.28 – 4.17 (m, 2H, O-CH_2), 4.11 (d, *J* = 5.9 Hz, 2H, O-CH_2), 3.87 (s, 3H, O-CH_3), 3.84 (s, 3H, O-CH_3), 3.83 (s, 3H, O-CH_3), 3.80 (s, 3H, O-CH_3), 3.19 (s, 1H, O-H); ^{13}C NMR (101 MHz, $CDCl_3$) δ 167.6, 153.0, 150.1, 149.8, 149.7, 144.5, 143.8, 128.3, 122.4, 115.9, 113.8, 111.7, 110.3, 103.9, 100.9, 70.6, 69.1, 68.6, 56.4, 55.8, 55.8, 51.6; HRMS (ESI): m/z calculated for $C_{22}H_{26}O_8^+$: 419.1700; found: 419.1686.

3-methyl-(4-(3-(2,6-dimethoxyphenoxy)-2-hydroxypropoxy)-3-methoxyphenyl)acrylate (A_8)

A white solid, yield 77.1%, m. p. 84.2-86.5 ℃ ; ^{1}H NMR (500 MHz, CHLOROFORM-D) δ 7.62 (d, *J* = 15.9 Hz, 1H, alkene-H), 7.07 (dd, *J* = 8.4, 2.0 Hz, 1H, Ar-H), 7.04 – 6.97 (m, 2H, Ar-H), 6.92 (d, *J* = 8.3 Hz, 1H, Ar-H), 6.57 (d, *J* = 8.4 Hz, 2H, Ar-H), 6.30 (d, *J* = 15.9 Hz, 1H, alkene-H), 4.29 – 4.25 (m, 2H, O-CH_2), 4.16 (d, *J* = 5.4 Hz, 2H, O-CH), 4.14 – 4.08 (m, 2H, O-CH_2), 3.86 (s, 3H, O-CH_3), 3.84 (s, 7H, O-CH_3), 3.78 (s, 3H, O-CH_3); ^{13}C NMR (126 MHz, CHLOROFORM-D) δ 167.7, 153.2, 150.5, 149.7, 144.8, 136.9, 127.8, 124.2, 122.5, 115.6, 113.3, 110.3, 105.1, 77.4, 77.1, 76.9, 75.5, 69.9, 68.8, 56.1, 55.9, 51.7; HRMS (ESI): m/z calculated for $C_{22}H_{26}O_8^+$: 419.1700; found: 419.1687.

3-methyl-(4-(3-(benzo[d][1,3]dioxol-5-yloxy)-2-hydroxypropoxy)-3-methoxyphenyl)acrylate (A_9)

A white solid, yield 59.4%, m. p. 89.6-91.2 ℃ ; ^{1}H NMR (500 MHz, CHLOROFORM-D) δ 7.60 (d, *J* = 16.0 Hz, 1H, alkene-H), 7.05 (dd, *J* = 8.3, 1.9 Hz, 1H, Ar-H), 7.01 (d, *J* = 2.0 Hz, 1H, Ar-H), 6.89 (d, *J* = 8.3 Hz, 1H, Ar-H), 6.67 (d, *J* = 8.4 Hz, 1H, Ar-H), 6.49 (d, *J* = 2.5 Hz, 1H, Ar-H), 6.31 (dd, *J* = 15.8, 9.3 Hz, 2H, Ar-H＆alkene-H), 5.88 (s, 2H, O-CH_2-O), 4.37 (dd, *J* = 10.4, 5.1 Hz, 1H, O-CH), 4.21 – 4.14 (m, 2H, O-CH_2), 4.05 (dd, *J* = 5.4, 1.6 Hz, 2H, O-CH_2), 3.85 (s, 3H, O-CH_3), 3.78

(s, 3H, O-CH_3), 3.42 (s, 1H, O-H); ^{13}C NMR (126 MHz, CHLOROFORM-D) δ 167.7, 153.9, 150.1, 149.6, 148.3, 144.7, 142.0, 128.2, 122.5, 115.9, 113.6, 110.3, 108.0, 105.8, 101.2, 98.2, 70.5, 69.6, 68.6, 55.9, 55.8, 51.7, 51.7; HRMS (ESI): m/z calculated for $C_{21}H_{22}O_8^+$: 403.1387; found: 403.1374.

3-methyl-(4-(3-(tert-butylthio)-2-hydroxypropoxy)-3-methoxyphenyl)acrylate (A_{10})

A yellow solid, yield 73.8%, m. p. 57.1-58.9 ℃; ^1H NMR (500 MHz, CHLOROFORM-D) δ 8.19 (d, *J* = 15.9 Hz, 1H, alkene-H), 7.76 – 7.59 (m, 2H, Ar-H), 7.47 (d, *J* = 8.3 Hz, 1H, Ar-H), 6.88 (d, *J* = 15.9 Hz, 1H, alkene-H), 4.71 (d, *J* = 6.8 Hz, 2H, O-CH_2), 4.61 (dd, *J* = 9.2, 6.1 Hz, 1H, O-CH), 4.44 (s, 3H, O-CH_3), 4.37 (s, 3H, O-CH_3), 3.71 (s, 1H, O-H), 3.47 – 3.33 (m, 2H, S-CH_2), 1.91 (s, 9H, CH_3); ^{13}C NMR (126 MHz, CHLOROFORM-D) δ 158.6, 144.6, 144.3, 140.2, 127.0, 122.4, 117.2, 115.5, 112.7, 82.4, 79.9, 69.2, 65.8, 58.6, 50.1, 49.3; HRMS (ESI): m/z calculated for $C_{18}H_{26}O_5S^+$: 355.1573; found: 355.1559.

3-methyl-(4-(3-((2-chlorophenyl)thio)-2-hydroxypropoxy)-3-methoxyphenyl)acrylate (A_{11})

A white liquid, yield 54.9%; ^1H NMR (400 MHz, $CDCl_3$) δ 7.62 (d, *J* = 15.9 Hz, 1H, alkene-H), 7.42 (dd, *J* = 7.8, 1.5 Hz, 1H, Ar-H), 7.36 (dd, *J* = 7.8, 1.4 Hz, 1H, Ar-H), 7.19 (td, *J* = 7.6, 1.5 Hz, 1H, Ar-H), 7.12 (td, *J* = 7.7, 1.6 Hz, 1H, Ar-H), 7.09 – 7.02 (m, 2H, Ar-H), 6.86 (d, *J* = 8.2 Hz, 1H, Ar-H), 6.32 (d, *J* = 15.9 Hz, 1H, alkene-H), 4.18 (dd, *J* = 9.1, 5.0 Hz, 1H, O-CH), 4.13 (dd, *J* = 10.9, 4.2 Hz, 2H, O-CH_2), 3.87 (s, 3H, O-CH_3), 3.80 (s, 3H, O-CH_3), 3.19 (dd, *J* = 12.2, 7.9 Hz, 2H, S-CH_2); ^{13}C NMR (101 MHz, $CDCl_3$) δ 167.6, 149.9, 149.7, 144.6, 134.5, 134.2, 129.9, 129.5, 128.3, 127.3, 127.2, 122.3, 116.0, 113.9, 110.2, 71.8, 68.5, 55.8, 51.6, 36.1; HRMS (ESI): m/z calculated for $C_{20}H_{21}ClO_5S^+$: 409.0871; found: 409.0853.

3-methyl-(4-(3-((3-chlorophenyl)thio)-2-hydroxypropoxy)-3-methoxyphenyl)acrylate (A_{12})

A white liquid, yield 60.1%; ^1H NMR (400 MHz, $CDCl_3$) δ 7.60 (d, *J* = 15.9 Hz, 1H, alkene-H), 7.33 (s, 1H, Ar-H), 7.21 (d, *J* = 7.6 Hz, 1H, Ar-H), 7.16 – 7.07 (m, 2H,

Ar-H), 7.04 – 6.96 (m, 2H, Ar-H), 6.79 (d, *J* = 8.2 Hz, 1H, Ar-H), 6.30 (d, *J* = 15.9 Hz, 1H, alkene-H), 4.16 (dt, *J* = 11.2, 5.5 Hz, 1H, O-CH), 4.07 (dd, *J* = 14.2, 8.2 Hz, 2H, O-CH_2), 3.90 (s, 1H, O-H), 3.88 (s, 3H, O-CH_3), 3.80 (s, 3H, O-CH_3), 3.21 (m, 2H, S-CH_2); ^{13}C NMR (101 MHz, $CDCl_3$) δ 167.6, 149.9, 149.4, 144.6, 137.9, 134.7, 130.0, 128.5, 128.1, 126.9, 126.3, 122.4, 115.8, 113.3, 110.2, 71.5, 68.6, 55.8, 51.6, 36.6; HRMS (ESI): m/z calculated for $C_{20}H_{21}ClO_5S^+$: 409.0871; found: 409.0853.

3-methyl-(4-(3-(diethylamino)-2-hydroxypropoxy)-3-methoxyphenyl)acrylate (A_{13})

A yellow liquid, yield 63.7%; ^{1}H NMR (500 MHz, CHLOROFORM-D) δ 7.61 (d, *J* = 15.9 Hz, 1H, alkene-H), 7.07 (dd, *J* = 8.3, 2.0 Hz, 1H, Ar-H), 7.03 (d, *J* = 2.0 Hz, 1H, Ar-H), 6.90 (d, *J* = 8.3 Hz, 1H, Ar-H), 6.30 (d, *J* = 15.9 Hz, 1H, alkene-H), 4.07 (dd, *J* = 8.4, 4.0 Hz, 1H, O-CH), 4.03 (dd, *J* = 9.7, 4.8 Hz, 2H, O-CH_2), 3.87 (s, 3H, O-CH_3), 3.78 (s, 3H, O-CH_3), 2.73 – 2.62 (m, 2H, N-CH_3), 2.55 (dd, *J* = 13.1, 7.0 Hz, 4H, N-CH_3), 1.04 (t, *J* = 7.1 Hz, 6H, CH_2-CH_3); ^{13}C NMR (126 MHz, CHLOROFORM-D) δ 167.8, 150.6, 149.6, 144.9, 127.7, 122.5, 115.6, 113.0, 110.1, 71.8, 65.8, 55.9, 55.8, 51.7, 47.2, 11.9; HRMS (ESI): m/z calculated for $C_{18}H_{27}NO_5^+$: 338.1962; found: 338.1957.

3-methyl-(4-(2-hydroxy-3-(2-methylpiperidin-1-yl)propoxy)-3-methoxyphenyl)acrylate (A_{14})

A yellow liquid, yield 70.5%; ^{1}H NMR (500 MHz, CHLOROFORM-D) δ 7.61 (d, *J* = 15.9 Hz, 1H, alkene-H), 7.06 (dd, *J* = 8.3, 1.9 Hz, 1H, Ar-H), 7.02 (d, *J* = 2.0 Hz, 1H, Ar-H), 6.89 (d, *J* = 8.3 Hz, 1H, Ar-H), 6.31 (s, 1H, alkene-H), 4.20 – 4.15 (m, 1H, O-CH), 4.07 – 3.99 (m, 2H, O-CH_2), 3.86 (s, 3H, O-CH_3), 3.78 (s, 3H, O-CH_3), 3.11 – 2.89 (m, 2H, N-CH_2), 2.86 – 2.71 (m, 1H, N-CH), 2.59 – 2.42 (m, 2H, N-CH_2), 1.65 (dd, *J* = 27.0, 19.9 Hz, 4H, CH_2), 1.40 (dd, *J* = 23.8, 15.0 Hz, 2H, CH_2), 1.16 – 1.10 (m, 3H, CH_3); ^{13}C NMR (126 MHz, CHLOROFORM-D) δ 167.7, 150.4, 149.6, 144.8, 127.9, 122.5, 115.7, 113.2, 110.2, 71.7, 66.2, 65.0, 55.9, 51.7, 34.1, 32.6, 25.5, 25.1, 23.4, 22.0; HRMS (ESI): m/z calculated for $C_{20}H_{29}NO_5^+$: 364.2118; found: 364.2105.

3-methyl-(4-(2-hydroxy-3-(3-methylpiperidin-1-yl)propoxy)-3-methoxyphenyl)acrylate (A_{15})

A yellow liquid, yield 58.4%; ^{1}H NMR (500 MHz, CHLOROFORM-D) δ 7.59 (d, *J* = 15.9 Hz, 1H, alkene-H), 7.04 (dd, *J* = 8.3, 2.0 Hz, 1H, Ar-H), 7.00 (d, *J* = 2.0 Hz, 1H, Ar-H), 6.87 (d, *J* = 8.2 Hz, 1H, Ar-H), 6.27 (d, *J* = 15.9 Hz, 1H, alkene-H), 4.15 – 4.08 (m, 1H, O-CH), 4.00 (d, *J* = 5.0 Hz, 2H, O-CH_2), 3.84 (s, 3H, O-CH_3), 3.76 (s, 3H, O-CH_3), 2.92 – 2.66 (m, 2H, N-CH_2), 2.50 – 2.41 (m, 2H, N-CH_2), 2.16 (t, *J* = 9.8 Hz, 1H, N-CH_2), 1.86 (d, *J* = 10.5 Hz, 1H, N-CH_2), 1.71 – 1.53 (m, 4H, N-CH_2), 0.87 (m, 1H, CH), 0.82 (d, *J* = 4.8 Hz, 3H, CH_3); ^{13}C NMR (126 MHz, CHLOROFORM-D) δ 167.7, 150.5, 149.6, 144.8, 127.7, 122.5, 115.6, 113.1, 110.1, 71.8, 65.4, 60.9, 60.8, 55.9, 55.5, 51.7, 32.7, 31.2, 22.0, 19.6; HRMS (ESI): m/z calculated for $C_{20}H_{29}NO_5^+$: 364.2118; found: 364.2114.

3-methyl-(4-(2-hydroxy-3-(4-methylpiperidin-1-yl)propoxy)-3-methoxyphenyl)acrylate (A_{16})

A yellow liquid, yield 71.1%; ^{1}H NMR (500 MHz, CHLOROFORM-D) δ 7.65 – 7.56 (m, 1H, alkene-H), 7.06 (dt, *J* = 8.2, 1.7 Hz, 1H, Ar-H), 7.03 (t, *J* = 2.2 Hz, 1H, Ar-H), 6.89 (dd, *J* = 8.3, 3.0 Hz, 1H, Ar-H), 6.29 (dd, *J* = 15.9, 10.4 Hz, 1H, alkene-H), 4.13 (td, *J* = 10.3, 5.2 Hz, 1H, O-CH), 4.03 (dd, *J* = 5.0, 2.6 Hz, 2H, O-CH_2), 3.87 (s, 3H, O-CH_3), 3.78 (s, 3H, O-CH_3), 2.96 (d, *J* = 12.1 Hz, 1H, N-CH_2), 2.80 (d, *J* = 11.6 Hz, 1H, N-CH_2), 2.53 – 2.45 (m, 2H, N-CH_2), 2.26 (td, *J* = 11.5, 2.2 Hz, 1H, N-CH_2), 1.97 (td, *J* = 11.7, 2.3 Hz, 1H, N-CH_2), 1.61 (d, *J* = 13.1 Hz, 2H, CH_2), 1.37 (dd, *J* = 16.7, 8.8 Hz, 1H, CH), 1.26 – 1.12 (m, 2H, CH_2), 0.91 (d, *J* = 6.5 Hz, 3H, CH_3); ^{13}C NMR (126 MHz, CHLOROFORM-D) δ 167.8, 150.5, 149.7, 144.8, 127.8, 122.5, 115.6, 113.1, 110.1, 71.8, 65.5, 60.7, 55.9, 52.8, 34.6, 34.2, 30.7, 22.07; HRMS (ESI): m/z calculated for $C_{20}H_{29}NO_5^+$: 364.2118; found: 364.2104.

***S*-1-ethyl-(2-hydroxy-3-(2-methoxy-4-(3-methoxy-3-oxoprop-1-en-1-yl)phenoxy)propyl)piperidine-3-carboxylate (A_{17})**

A yellow liquid, yield 52.4%; ^{1}H NMR (500 MHz, CHLOROFORM-D) δ 8.20 (dd, *J* = 15.9, 2.5 Hz, 1H, alkene-H), 7.71 – 7.61 (m, 2H, Ar-H), 7.53 – 7.46 (m, 1H, Ar-H), 6.89 (dd, *J* = 15.9, 2.8 Hz, 1H, alkene-H), 4.76 – 4.68 (m, 3H, O-CH & O-CH_2), 4.63 (d, *J* = 2.6 Hz, 2H, O-CH_2), 4.46 (s, 3H, O-CH_3), 4.37 (s, 3H, O-CH_3), 3.37 (s, 1H, CH), 3.17 (dd, *J* = 48.4, 19.9 Hz, 4H, N-CH_2), 2.89 (dd, *J* = 48.9, 39.3 Hz, 2H,

N-CH_2), δ 2.38 (d, *J* = 88.6 Hz, 2H, CH_2), 2.23 – 2.04 (m, 2H, CH_2), 1.83 (t, *J* = 5.0 Hz, 3H, CH_3); ^{13}C NMR (126 MHz, CHLOROFORM-D) δ 174.5, 168.3, 151.1, 150.2, 145.4, 128.4, 123.1, 116.2, 113.8, 110.8, 72.3, 66.2, 61.3, 61.1, 56.5, 55.5, 52.3, 42.4, 27.3, 25.2, 25.0, 14.9; HRMS (ESI): m/z calculated for $C_{22}H_{31}NO_7^+$: 422.2173; found: 422.2155.

***R*-1-ethyl-(2-hydroxy-3-(2-methoxy-4-(3-methoxy-3-oxoprop-1-en-1-yl)phenoxy)propyl)piperidine-3-carboxylate (A_{18})**

A yellow liquid, yield 57.8%; ^{1}H NMR (500 MHz, CHLOROFORM-D) δ 8.20 (d, *J* = 15.9 Hz, 1H, alkene-H), 7.69 – 7.57 (m, 2H, Ar-H), 7.48 (d, *J* = 8.3 Hz, 1H, Ar-H), 6.88 (d, *J* = 15.9 Hz, 1H, alkene-H), 4.77 – 4.65 (m, 3H, O-CH＆O-CH_2), 4.62 (d, *J* = 4.7 Hz, 2H, O-CH_2), 4.45 (s, 3H, O-CH_3), 4.37 (s, 3H, O-CH_3), 3.69 – 3.33 (m, 2H, N-CH_2), 3.12 (t, *J* = 10.0 Hz, 4H, N-CH_2), 2.81 (d, *J* = 54.2 Hz, 1H, CH), 2.33 – 2.22 (m, 2H, CH_2), 2.14 (dd, *J* = 25.8, 12.6 Hz, 2H, CH_2), 1.82 (t, *J* = 7.1 Hz, 3H, CH_3); ^{13}C NMR (126 MHz, CHLOROFORM-D) δ 174.6, 168.3, 151.1, 150.3, 145.4, 128.4, 123.1, 116.2, 113.8, 110.8, 72.2, 66.3, 61.3, 61.1, 56.5, 55.4, 52.3, 42.4, 27.3, 25.2, 25.0, 14.9; HRMS (ESI): m/z calculated for $C_{22}H_{31}NO_7^+$: 422.2173; found: 422.2157.

3-methyl-(4-(2-hydroxy-3-morpholinopropoxy)-3-methoxyphenyl)acrylate (A_{19})

A yellow liquid, yield 72.0%; ^{1}H NMR (500 MHz, CHLOROFORM-D) δ 7.56 (d, *J* = 15.9 Hz, 1H, alkene-H), 7.08 – 6.95 (m, 2H, Ar-H), 6.84 (d, *J* = 8.3 Hz, 1H, Ar-H), 6.25 (dd, *J* = 15.9, 8.3 Hz, 1H, alkene-H), 4.13 (dd, *J* = 9.5, 3.8 Hz, 1H, O-CH), 4.04 – 3.93 (m, 2H, O-CH_2), 3.82 (s, 3H, O-CH_3), 3.73 (s, 3H, O-CH_3), 3.66 (dt, *J* = 6.2, 3.1 Hz, 4H, O-CH_2), 3.32 (s, 1H, O-H), 2.62 – 2.57 (m, 2H, N-CH_2), 2.50 (t, *J* = 6.9 Hz, 2H, N-CH_2), 2.46 – 2.39 (m, 2H, N-CH_2); ^{13}C NMR (126 MHz, CHLOROFORM-D) δ 167.7, 150.3, 149.5, 144.7, 127.8, 122.5, 115.7, 113.6, 110.1, 71.7, 66.9, 65.6, 61.0, 55.8, 53.8, 51.7; HRMS (ESI): m/z calculated for $C_{18}H_{25}NO_6^+$: 352.1754; found: 352.1751.

3-methyl-(4-(3-(4-ethylpiperazin-1-yl)-2-hydroxypropoxy)-3-methoxyphenyl)acrylate (A_{20})

A yellow liquid, yield 67.4%; ^{1}H NMR (500 MHz, CHLOROFORM-D) δ 7.56

(dd, J = 15.9, 12.4 Hz, 1H, alkene-H), 7.04 – 6.97 (m, 2H, Ar-H), 6.85 (d, J = 8.3 Hz, 1H, Ar-H), 6.25 (dd, J = 15.9, 8.9 Hz, 1H, alkene-H), 4.28 (m, 2H, O-CH_2), 4.20 – 4.14 (m, 1H, O-CH), 3.99 (m, J = 24.7, 9.8, 4.6 Hz, 2H, N-CH_2), 3.82 (s, 3H, O-CH_3), 3.74 (s, 2H, O-CH_3), 3.10 – 2.88 (m, 2H, N-CH_2), 2.58 – 2.43 (m, 2H, N-CH_2), 1.62 (dd, J = 23.8, 14.9 Hz, 4H, N-CH_2), 1.37 (q, J = 18.0, 8.5 Hz, 2H, CH_2), 1.12 (t, J = 5.7 Hz, 3H, CH_3); ^{13}C NMR (126 MHz, CHLOROFORM-D) δ 167.7, 150.3, 149.5, 144.8, 127.8, 122.5, 115.6, 113.0, 110.2, 71.4, 66.1, 65.0, 56.8, 55.9, 55.8, 52.7, 51.7, 22.0; HRMS (ESI): m/z calculated for $C_{20}H_{31}N_2O_5^+$: 379.2227; found: 379.2233.

3-methy-(4-(2-hydroxy-3-(4-isopropylpiperazin-1-yl)propoxy)-3-methoxyphenyl)acrylate (A_{21})

A yellow liquid, yield 52.8%; ^1H NMR (500 MHz, CHLOROFORM-D) δ 7.59 (d, J = 15.9 Hz, 1H, alkene-H), 7.07 – 6.99 (m, 2H, Ar-H), 6.86 (d, J = 8.3 Hz, 1H, Ar-H), 6.27 (dd, J = 15.9, 9.6 Hz, 1H, alkene-H), 4.20 – 4.10 (m, 1H, O-CH), 4.04 – 3.97 (m, 2H, O-CH_2), 3.84 (s, 3H, O-CH_3), 3.76 (s, 3H, O-CH_3), 3.61 (s, 1H, O-H), 2.78 – 2.47 (m, 11H, N-CH_2 & N-CH), 1.07 (d, J = 6.5 Hz, 6H, CH_3); ^{13}C NMR (126 MHz, CHLOROFORM-D) δ 167.7, 150.4, 149.6, 144.8, 127.9, 122.5, 115.7, 113.2, 110.1, 71.8, 65.7, 60.3, 55.9, 54.9, 51.7, 48.5, 22.0, 18.4; HRMS (ESI): m/z calculated for $C_{21}H_{32}N_2O_5^+$: 393.2384; found: 393.2389.

3-methyl-(4-(3-(4-(4-fluorophenyl)piperazin-1-yl)-2-hydroxypropoxy)-3-methoxyphenyl)acrylate (A_{22})

A yellow liquid, yield 71.6%; ^1H NMR (400 MHz, $CDCl_3$) δ 7.62 (d, J = 15.9 Hz, 1H, alkene-H), 7.07 (dd, J = 8.3, 1.9 Hz, 1H, Ar-H), 7.04 (d, J = 1.9 Hz, 1H, Ar-H), 6.99 – 6.91 (m, 3H, Ar-H), 6.88 – 6.82 (m, 2H, Ar-H), 6.31 (d, J = 15.9 Hz, 1H, alkene-H), 4.19 (q, J = 9.0, 4.6 Hz, 1H, O-CH), 4.09 – 4.05 (m, 2H, O-CH_2), 3.88 (s, 3H, O-CH_3), 3.79 (s, 3H, O-CH_3), 3.18 – 3.06 (m, 4H, N-CH_2), 2.85 – 2.77 (m, 2H, N-CH_2), 2.69 – 2.55 (m, 4H, N-CH_2); ^{13}C NMR (101 MHz, $CDCl_3$) δ 167.7, δ 157.3 (d, $^1J_{C-F}$ = 238.9 Hz), 156.1, 150.4, 149.7, 147.8 (d, $^4J_{C-F}$ = 2.2 Hz), 144.7, 128.0, 122.4, 117.9 (d, $^3J_{C-F}$ = 7.6 Hz), 115.6 (d, $^2J_{C-F}$ = 22.1 Hz), 113.3, 110.2, 77.4, 77.1, 76.84, 71.81, 65.7, 60.5, 55.9, 53.4, 51.7, 50.3; ^{19}F NMR (471 MHz, CHLOROFORM-D) δ -114.43; HRMS (ESI): m/z calculated for $C_{24}H_{29}N_2O_5F^+$: 445.2133; found: 445.2114.

3-methyl-(4-(2-hydroxy-3-(4-(3-methoxyphenyl)piperazin-1-yl)propoxy)-3-methoxyphenyl)acrylate (A_{23})

A white solid, yield 64.6%, m. p. 45.1-47.1 ℃; ^{1}H NMR (500 MHz, CHLOROFORM-D) δ 7.62 (d, J = 15.9 Hz, 1H, alkene-H), 7.15 (t, J = 8.2 Hz, 1H, Ar-H), 7.06 (dd, J = 8.3, 2.0 Hz, 1H, Ar-H), 7.03 (d, J = 2.0 Hz, 1H, Ar-H), 6.90 (d, J = 8.3 Hz, 1H, Ar-H), 6.52 (dd, J = 7.8, 2.2 Hz, 1H, Ar-H), 6.45 (t, J = 2.3 Hz, 1H, Ar-H), 6.41 (dd, J = 7.6, 2.3 Hz, 1H, Ar-H), 6.30 (d, J = 16.0 Hz, 1H, alkene-H), 4.19 (q, J = 9.0, 4.4 Hz, 1H, O-CH), 4.11 – 4.02 (m, 2H, O-CH_2), 3.86 (s, 3H, O-CH_3), 3.78 (s, 3H, O-CH_3), 3.77 (s, 3H, O-CH_3), 3.27 – 3.12 (m, 4H, N-CH_2), 2.81 – 2.74 (m, 2H, N-CH_2), 2.65 – 2.53 (m, 4H, N-CH_2); ^{13}C NMR (126 MHz, CHLOROFORM-D) δ 167.6, 160.5, 152.5, 150.3, 149.6, 144.7, 129.7, 127.8, 122.4, 115.6, 113.2, 110.1, 108.8, 104.4, 102.4, 77.4, 77.1, 76.9, 71.7, 65.7, 60.4, 55.8, 55.1, 53.3, 51.6, 49.0; HRMS (ESI): m/z calculated for $C_{25}H_{32}N_2O_6^+$: 457.2333; found: 457.2319.

3-methyl-(4-(3-(4-benzylpiperazin-1-yl)-2-hydroxypropoxy)-3-methoxyphenyl)acrylate (A_{24})

A yellow liquid, yield 68.4%; ^{1}H NMR (500 MHz, CHLOROFORM-D) δ 7.48 (dd, J = 15.9, 12.9 Hz, 1H, alkene-H), 7.22 (td, J = 7.5, 1.7 Hz, 1H, Ar-H), 7.11 (m, J = 8.0, 5.5, 3.5 Hz, 1H, Ar-H), 6.97 (td, J = 7.5, 1.1 Hz, 1H, Ar-H), 6.93 – 6.88 (m, 3H, Ar-H), 6.76 (dd, J = 8.3, 2.3 Hz, 1H), 6.17 (dd, J = 15.9, 9.2 Hz, 1H, alkene-H), 4.00 (dt, J = 9.0, 4.5 Hz, 1H, O-CH), 3.91 – 3.89 (m, 2H, O-CH_2), 3.74 (s, 3H, O-CH_3), 3.66 (s, 3H, O-CH_3), 3.46 (s, 2H, N-CH_2), 2.55 (m, 2H, N-CH_2), 2.44 – 2.29 (m, 8H, N-CH_2); ^{13}C NMR (101 MHz, $CDCl_3$) δ 167.6, 150.4, 149.7, 144.7, 136.6, 132.7, 130.4, 128.4, 127.9, 122.4, 115.7, 113.3, 110.2, 71.7, 67.6, 65.5, 62.1, 60.3, 55.9, 53.0, 51.6; HRMS (ESI): m/z calculated for $C_{25}H_{32}N_2O_5^-$: 441.2384; found: 441.2528.

3-methyl-(4-(2-hydroxy-3-(4-(3-nitrobenzyl)piperazin-1-yl)propoxy)-3 methoxyphenyl)acrylate(A_{25})

A yellow liquid, yield 60.7%; ^{1}H NMR (400 MHz, $CDCl_3$) δ 8.21 (s, 1H, alkene-H), 8.11 (d, J = 8.1 Hz, 1H, alkene-H), 7.67 (d, J = 7.6 Hz, 1H, Ar-H), 7.62 (d, J = 15.9 Hz, 1H, Ar-H), 7.49 (t, J = 7.9 Hz, 1H, Ar-H), 7.12 – 7.01 (m, 2H, Ar-H), 6.91 (d, J =

8.3 Hz, 1H, Ar-H), 6.30 (dd, *J* = 15.9, 7.4 Hz, 1H, alkene-H), 4.15 (dt, *J* = 9.1, 4.9 Hz, 1H, O-CH), 4.05 (d, *J* = 4.0 Hz, 2H, O-CH_2), 3.88 (s, 3H, O-CH_3), 3.80 (s, 3H, O-CH_3), 3.60 (s, 2H, N-CH_2), 2.71 (s, 2H, N-CH_2), 2.56 (td, *J* = 19.0, 11.7 Hz, 8H, piperazine-H); ^{13}C NMR (101 MHz, $CDCl_3$) δ 167.6, 150.4, 149.6, 148.3, 144.7, 140.6, 135.0, 129.1, 127.9, 123.7, 122.4, 122.2, 115.7, 113.3, 110.2, 71.7, 65.6, 61.9, 60.3, 55.8, 53.1, 51.6, 21.9; HRMS (ESI): m/z calculated for $C_{26}H_{34}N_2O_5^+$: 486.2234; found: 486.2216.

(*E*)-methyl-3-(4-(3-(4-(2-fluorobenzyl)piperazin-1-yl)-2-hydroxypropoxy)-3-methoxyphenyl)acrylate (A_{26})

A yellow liquid, yield 73.4%; ^{1}H NMR (500 MHz, CHLOROFORM-D) δ 8.18 (t, *J* = 15.0 Hz, 1H, alkene-H), 7.93 (t, *J* = 7.5 Hz, 1H, Ar-H), 7.80 (dd, *J* = 10.4, 4.9 Hz, 1H, Ar-H), 7.68 (td, *J* = 7.5, 1.1 Hz, 1H, Ar-H), 7.66 – 7.59 (m, 1H, Ar-H), 7.47 (dd, *J* = 8.3, 2.8 Hz, 3H, Ar-H), 6.87 (dd, *J* = 15.9, 10.1 Hz, 1H, alkene-H), 4.71 (dd, *J* = 9.2, 4.7 Hz, 1H, O-CH), 4.61 (d, *J* = 5.3 Hz, 2H, O-CH_2), 4.44 (s, 3H, O-CH_3), 4.36 (s, 3H, O-CH_3), 4.17 (s, 2H, N-CH_2), 3.26 (s, 2H, N-CH_2), 3.17 – 3.05 (m, 8H, N-CH_2); ^{13}C NMR (126 MHz, CHLOROFORM-D) δ 168.3, 162.1 (d, $^1J_{C-F}$ = 246.2 Hz), 151.0, 144.8, 132.3 (d, $^4J_{C-F}$ = 3.6 Hz), 129.54 (d, $^3J_{C-F}$ = 7.6 Hz), 125.1, 124.5, 123.1, 117.3, 115.9 (d, $^2J_{C-F}$ = 21.9 Hz), 113.9, 110.8, 72.4, 68.3, 66.1, 61.1, 56.5, 55.8, 53.4, 52.3; ^{19}F NMR (471 MHz, CHLOROFORM-D) δ -112.47; HRMS (ESI): m/z calculated for $C_{25}H_{31}N_2O_5F^+$: 459.2289; found: 459.2273.

3-methyl-(4-(3-(4-(3-fluorobenzyl)piperazin-1-yl)-2-hydroxypropoxy)-3-methoxyphenyl)acrylate (A_{27})

A yellow liquid, yield 76.3%; ^{1}H NMR (500 MHz, CHLOROFORM-D) δ 7.52 (d, *J* = 15.9 Hz, 1H, alkene-H), 7.16 (dd, *J* = 8.0, 2.0 Hz, 1H, Ar-H), 6.96 (dd, *J* = 11.5, 9.8 Hz, 4H, Ar-H), 6.87 – 6.77 (m, 2H, Ar-H), 6.19 (dd, *J* = 15.9, 10.4 Hz, 1H, alkene-H), 4.04 (dd, *J* = 9.2, 4.7 Hz, 1H, O-CH), 3.94 (d, *J* = 5.3 Hz, 2H, O-CH_2), 3.77 (s, 3H, O-CH_3), 3.69 (s, 3H, O-CH_3), 3.39 (s, 2H, N-CH_2), 2.59 (s, 2H, N-CH_2), 2.50 – 2.37 (m, 8H, N-CH_2); ^{13}C NMR (126 MHz, CHLOROFORM-D) δ 158.1, 154.3 (d, $^1J_{C-F}$ = 236.5 Hz), 144.3, 143.7, 139.7, 139.3, 136.7 (d, $^3J_{C-F}$ = 5.7 Hz), 127.7 (d, $^3J_{C-F}$ = 6.4 Hz), 123.6, 121.9, 116.5 (d, $^2J_{C-F}$ = 14.9 Hz), 115.1 (d, $^2J_{C-F}$ = 16.9 Hz), 112.1, 81.3, 78.1, 76.3, 73.8, 72.2, 68.6, 66.4, 65.3; ^{19}F NMR (471 MHz, CHLOROFORM-D) δ -112.46;

HRMS (ESI): m/z calculated for $C_{25}H_{31}N_2O_5F^+$: 459.2289; found: 459.2272.

3-methyl-(4-(3-(4-(3-chlorobenzyl)piperazin-1-yl)-2-hydroxypropoxy)-3-methoxyphenyl)acrylate (A_{28})

A yellow liquid, yield 74.9%; ^{1}H NMR (400 MHz, $CDCl_3$) δ 7.62 (d, *J* = 15.9 Hz, 1H, alkene-H), 7.33 (s, 1H, Ar-H), 7.21 (dt, *J* = 7.0, 5.1 Hz, 3H, Ar-H), 7.10 – 7.01 (m, 2H, Ar-H), 6.91 (d, *J* = 8.3 Hz, 1H, Ar-H), 6.31 (d, *J* = 15.9 Hz, 1H, alkene-H), 4.19 – 4.10 (m, 1H, O-CH), 4.04 (d, *J* = 4.4 Hz, 2H, O-CH_2), 3.87 (s, 3H, O-CH_3), 3.79 (s, 3H, O-CH_3), 3.47 (s, 2H, N-CH_2), 2.69 (s, 2H, N-CH_2), 2.62 – 2.41 (m, 8H, piperazine-H); ^{13}C NMR (101 MHz, $CDCl_3$) δ 167.6, 150.4, 149.7, 144.7, 140.3, 134.1, 129.5, 129.0, 127.9, 127.2, 127.1, 122.1, 115.7, 113.3, 110.2, 71.8, 65.6, 62.3, 60.3, 55.9, 53.1, 51.6, 21.9; HRMS (ESI): m/z calculated for $C_{25}H_{31}N_2O_5Cl^+$: 475.1994; found: 475.1978.

(*E*)-methyl-3-(4-(3-(4-(4-chlorobenzyl)piperazin-1-yl)-2-hydroxypropoxy)-3-methoxyphenyl)acrylate (A $_{29}$)

A yellow liquid, yield 66.3%; ^{1}H NMR (400 MHz, $CDCl_3$) δ 7.83 – 7.44 (m, 1H, alkene-H), 7.28 (t, *J* = 4.2 Hz, 2H, Ar-H), 7.25 (d, *J* = 9.0 Hz, 2H, Ar-H), 7.10 – 7.03 (m, 2H, Ar-H), 6.91 (d, *J* = 8.5 Hz, 1H, Ar-H), 6.30 (dd, *J* = 15.9, 8.4 Hz, 1H, alkene-H), 4.14 (dd, *J* = 8.3, 4.3 Hz, 1H, O-CH), 4.05 (d, *J* = 4.4 Hz, 2H, O-CH_2), 3.87 (s, 3H, O-CH_3), 3.80 (s, 3H, O-CH_3), 3.47 (s, 2H, N-CH_2), 2.64 (dd, *J* = 28.4, 5.7 Hz, 2H, N-CH_2), 2.52 (dd, *J* = 23.8, 13.9 Hz, 8H, N-CH_2); ^{13}C NMR (101 MHz, $CDCl_3$) δ 167.6, 149.7, 144.7, 144.1, 136.6, 132.7, 130.4, 128.4, 127.9, 122.4, 115.7, 113.3, 110.2, 71.7, 67.6, 65.5, 62.1, 60.3, 55.9, 53.0, 51.6; HRMS (ESI): m/z calculated for $C_{25}H_{31}N_2O_5Cl^+$: 475.1994; found: 475.1979.

3-methyl-(4-(2-hydroxy-3-(4-(3-methylbenzyl)piperazin-1-yl)propoxy)-3-methoxyphenyl)acrylate (A_{30})

A yellow liquid, yield 78.2%; ^{1}H NMR (400 MHz, $CDCl_3$) δ 7.62 (d, *J* = 15.9 Hz, 1H, alkene-H), 7.20 (t, *J* = 7.5 Hz, 1H, alkene-H), 7.14 – 7.08 (m, 3H, Ar-H), 7.07 – 7.02 (m, 2H, Ar-H), 6.91 (d, *J* = 8.3 Hz, 1H, Ar-H), 6.31 (d, *J* = 15.9 Hz, 1H, Ar-H), 4.14 (dd, *J* = 8.9, 4.7 Hz, 1H, O-CH), 4.04 (d, *J* = 5.0 Hz, 2H, O-CH_2), 3.87 (s, 3H, O-CH_3), 3.79 (s, 3H, O-CH_3), 3.47 (s, 2H, N-CH_2), 2.69 (s, 2H, N-CH_2), 2.58 – 2.43 (m, 8H, piperazine-H), 2.34 (s, 3H, Ar-CH_3); ^{13}C NMR (101 MHz, $CDCl_3$) δ 167.6, 150.4,

149.7, 144.7, 137.8, 129.9, 128.1, 127.8, 126.3, 124.6, 122.4, 116.8, 115.6, 113.3, 110.2, 71.8, 65.5, 63.0, 60.3, 55.9, 53.1, 51.6, 51.4, 21.4; HRMS (ESI): m/z calculated for $C_{26}H_{34}N_2O_5^+$: 455.2540; found: 455.2520.

3-methyl-(4-(2-hydroxy-3-(4-(2-methylbenzyl)piperazin-1-yl)propoxy)-3-methoxyphenyl)acrylate (A_{31})

A yellow liquid, yield 72.9%; ^{1}H NMR (400 MHz, $CDCl_3$) δ 7.63 (d, *J* = 15.9 Hz, 1H, alkene-H), 7.24 (d, *J* = 1.5 Hz, 1H, Ar-H), 7.18 – 7.13 (m, 3H, Ar-H), 7.10 – 7.03 (m, 2H, Ar-H), 6.91 (d, *J* = 8.3 Hz, 1H, Ar-H), 6.30 (dd, *J* = 15.9, 8.2 Hz, 1H, alkene-H), 4.16 – 4.10 (m, 1H, O-CH), 4.04 (d, *J* = 5.0 Hz, 2H, O-CH_2), 3.87 (s, 3H, O-CH_3), 3.80 (s, 3H, O-CH_3), 3.46 (s, 2H, N-CH_2), 2.75 (d, *J* = 69.1 Hz, 2H, N-CH_2), 2.61 – 2.47 (m, 4H, piperazine-H), 2.36 (s, 3H, Ar-CH_3); ^{13}C NMR (101 MHz, $CDCl_3$) δ 167.6, 150.4, 149.7, 144.7, 137.5, 136.3, 130.2, 129.8, 127.8, 127.0, 125.4, 122.4, 115.6, 113.3, 110.2, 71.7, 65.4, 60.7, 60.3, 55.91, 53.22, 51.6, 21.9, 19.2.

HRMS (ESI): m/z calculated for $C_{26}H_{34}N_2O_5^+$: 455.2540; found: 455.2522.

5. ^{1}H NMR, ^{13}C NMR, ^{19}F NMR, and HRMS spectra for the intermediates and target compounds

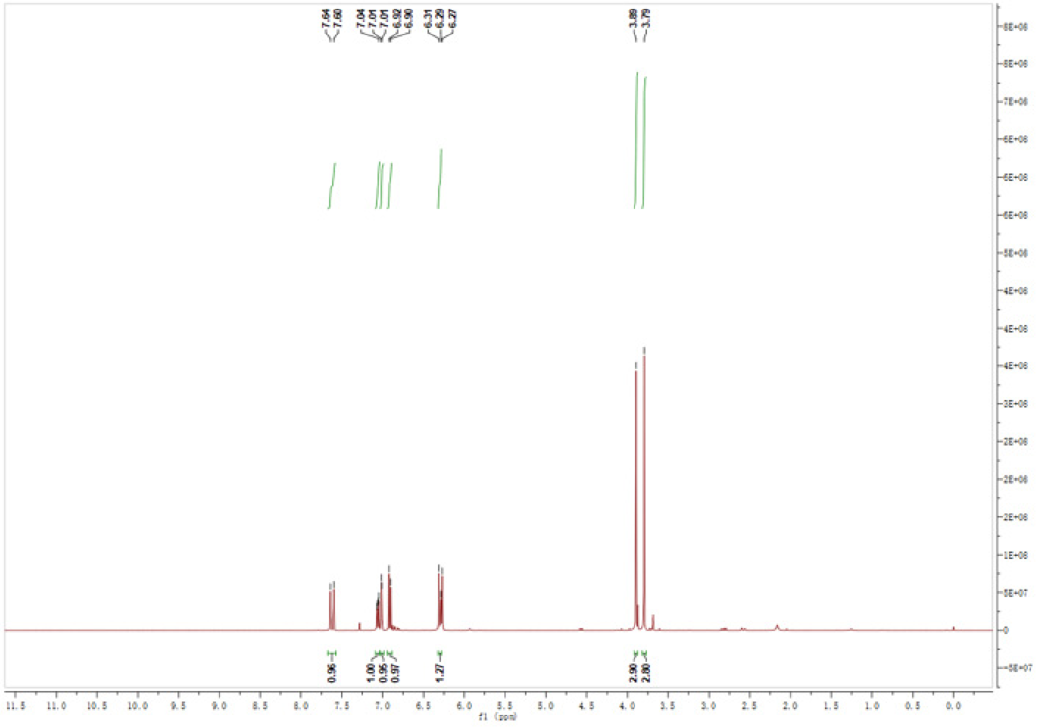

^{1}H NMR spectrum ($CDCl_3$, 400 MHz) of intermediate **1**.

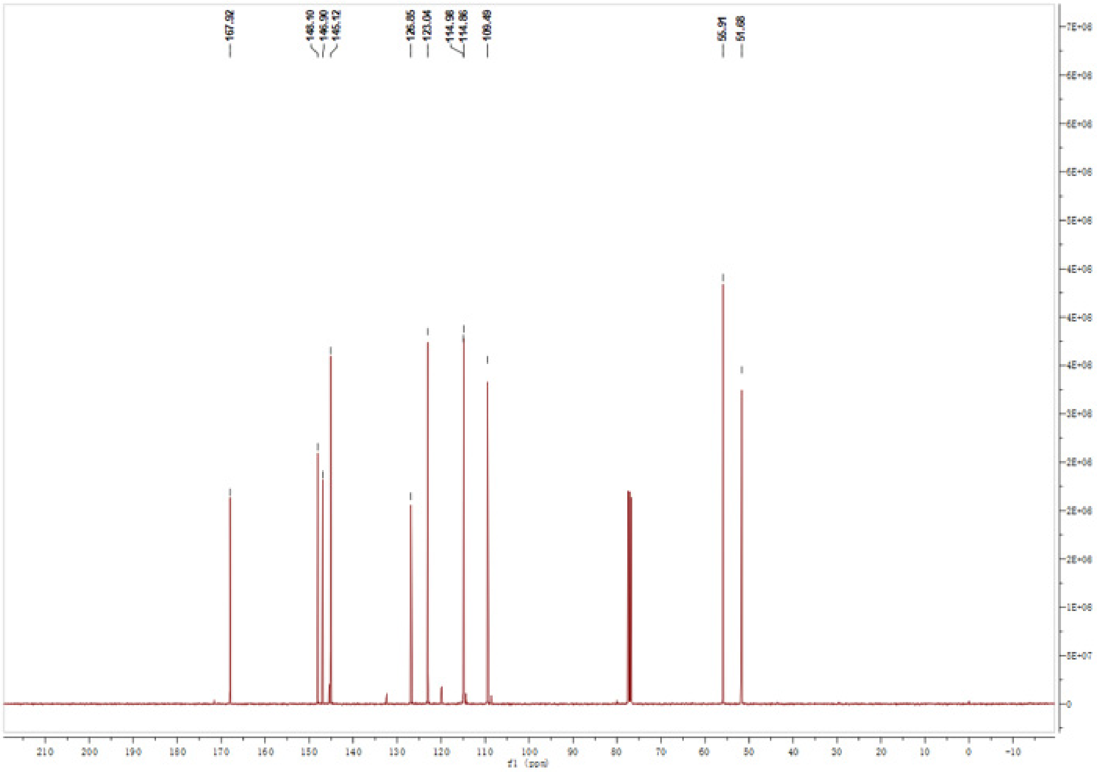

^{13}C NMR spectrum (CDCl3, 101 MHz) of intermediate **1**.

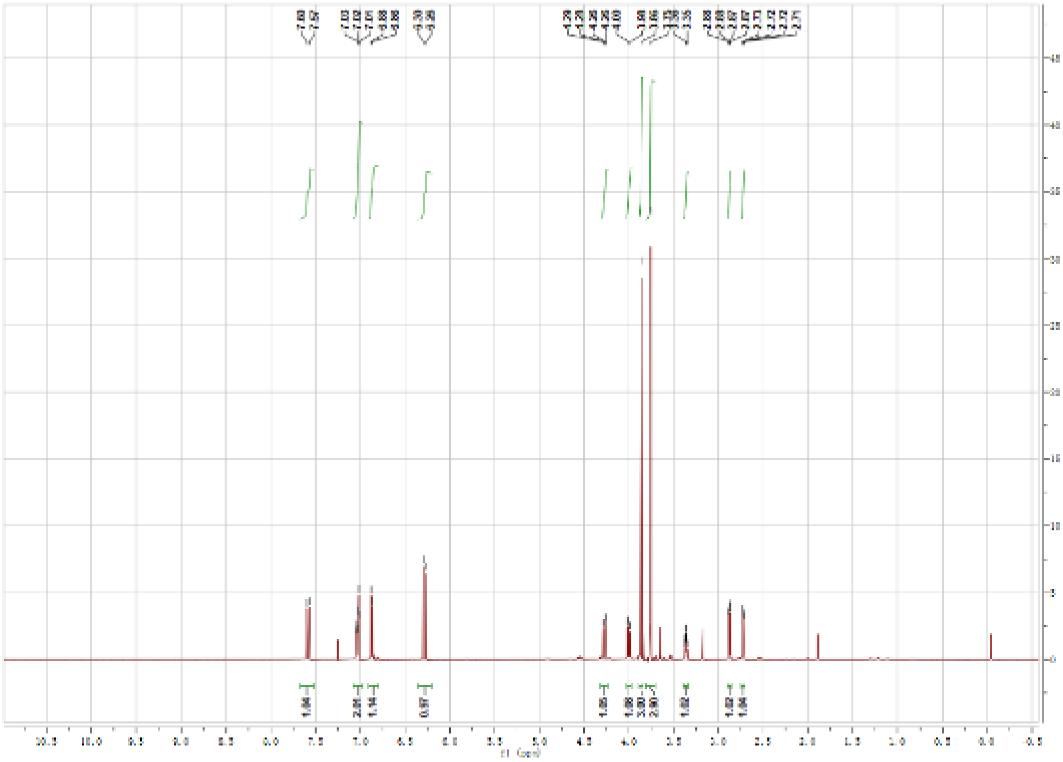

^{1}H NMR spectrum ($CDCl_3$, 500 MHz) of intermediate **2**.

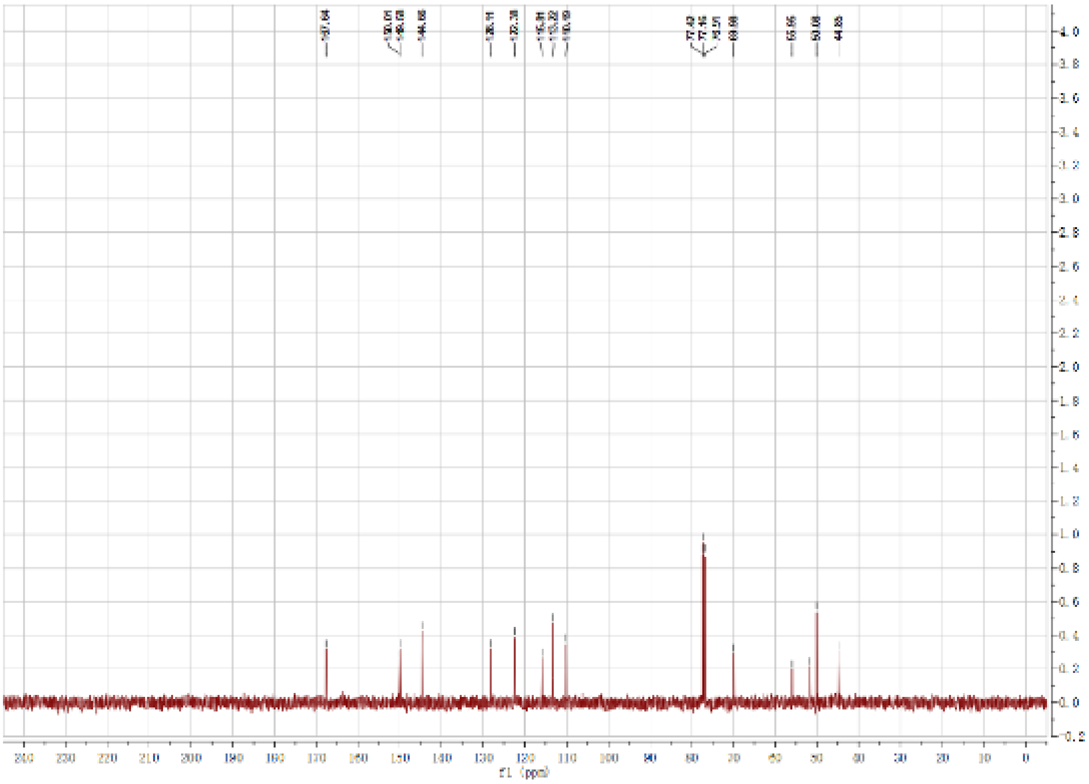

^{13}C NMR spectrum (CDCl3, 126 MHz) of intermediate **2**.

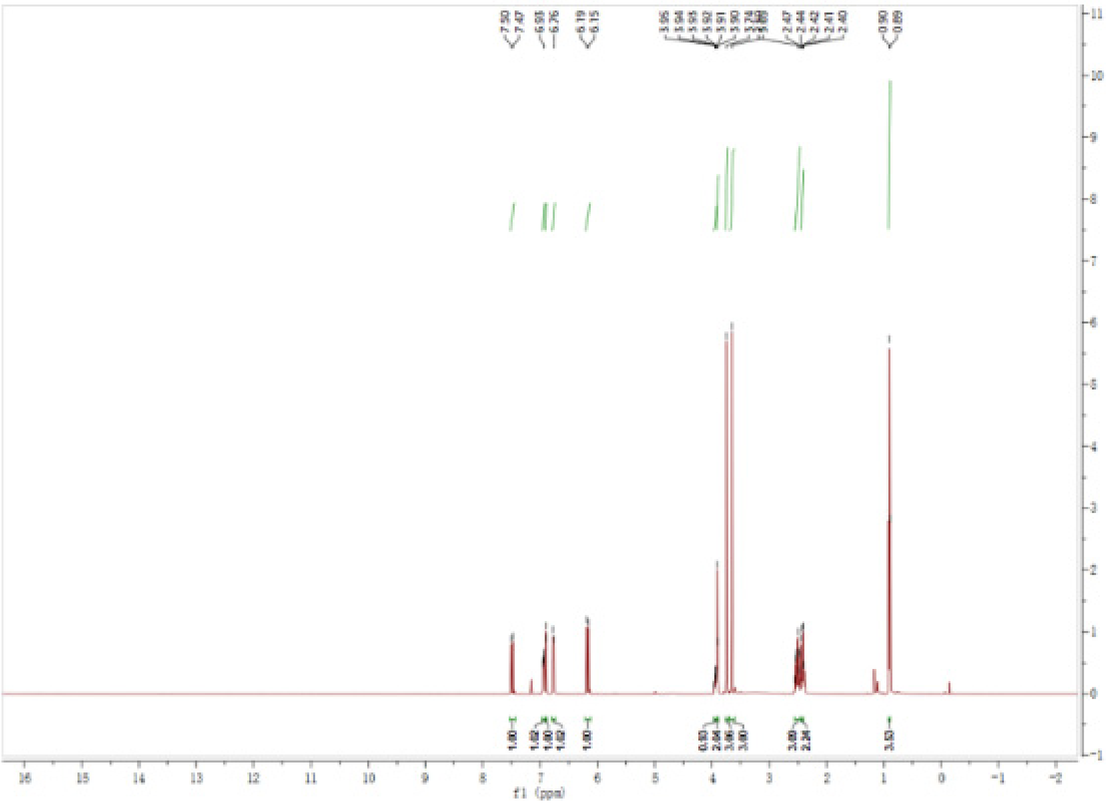

^{1}H NMR spectrum ($CDCl_3$, 500 MHz) of **A_1**.

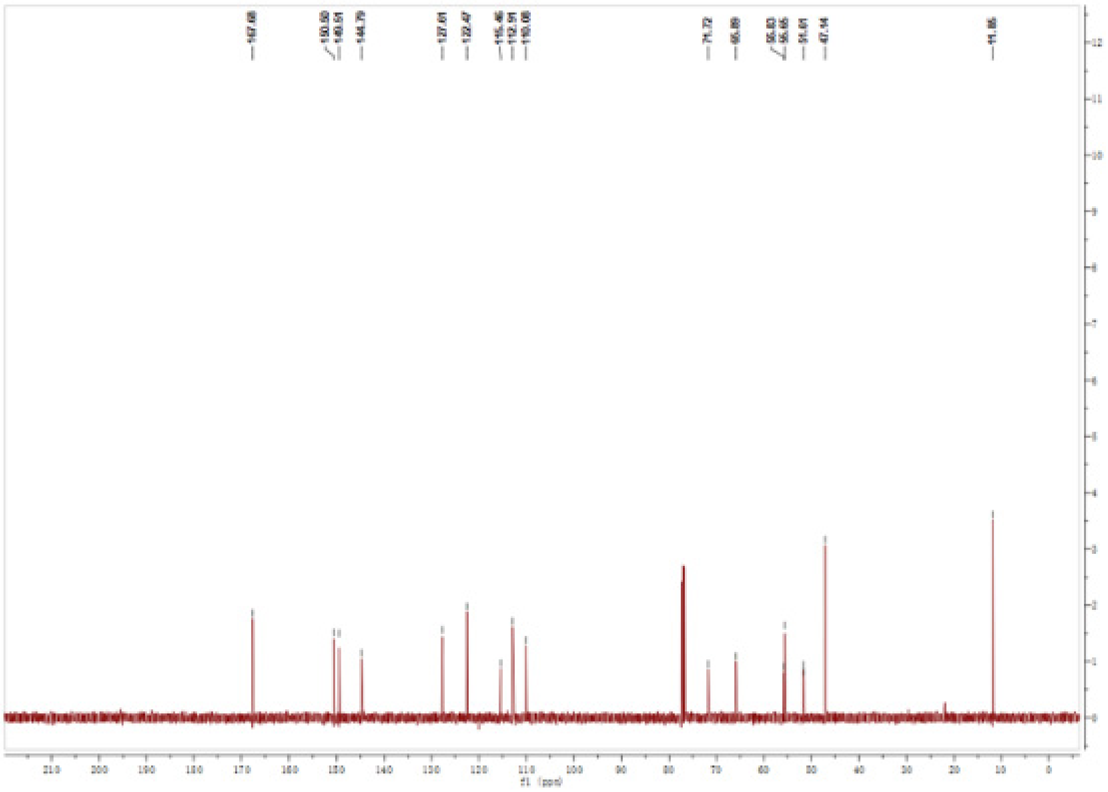

^{13}C NMR spectrum ($CDCl_3$, 126 MHz) of **A_1**.

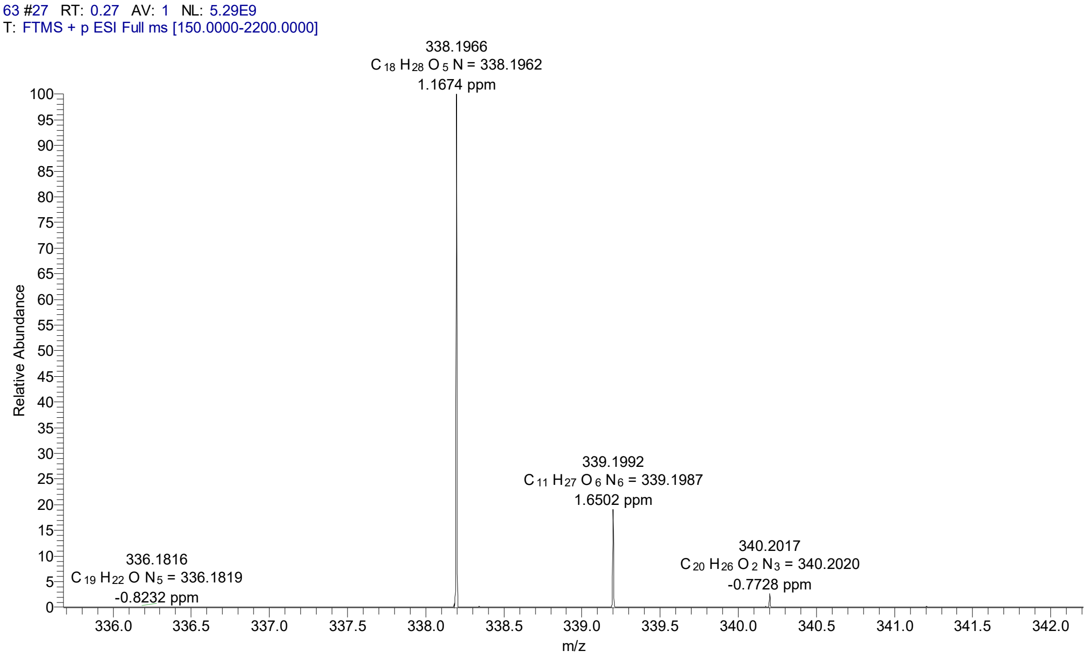

HRMS spectrum of target compound **A_1**.

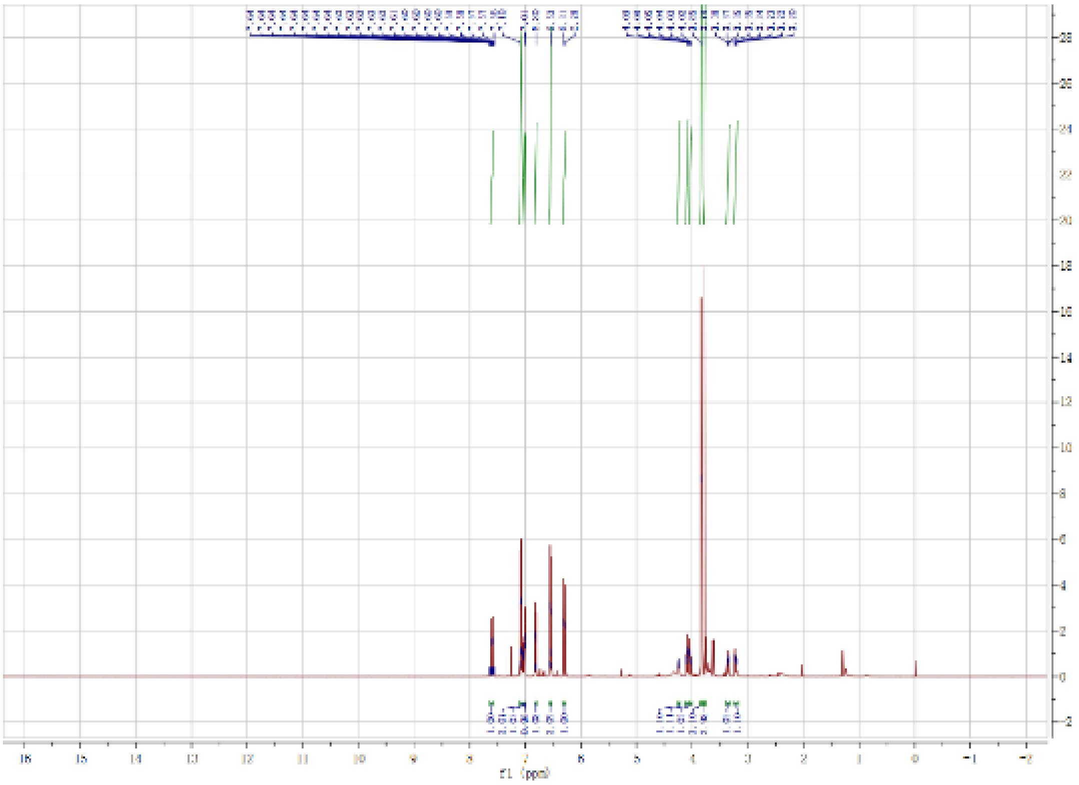

^{1}H NMR spectrum ($CDCl_3$, 500 MHz) of **A_2**.

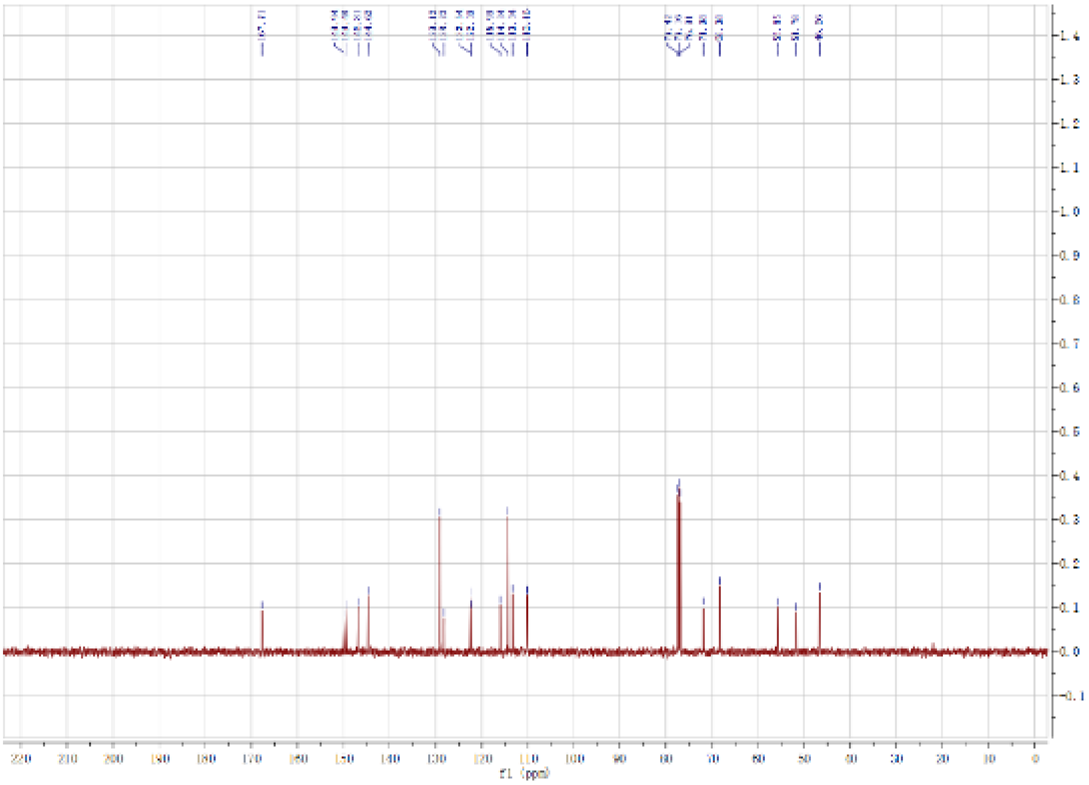

^{13}C NMR spectrum ($CDCl_3$, 126 MHz) of **A_2**.

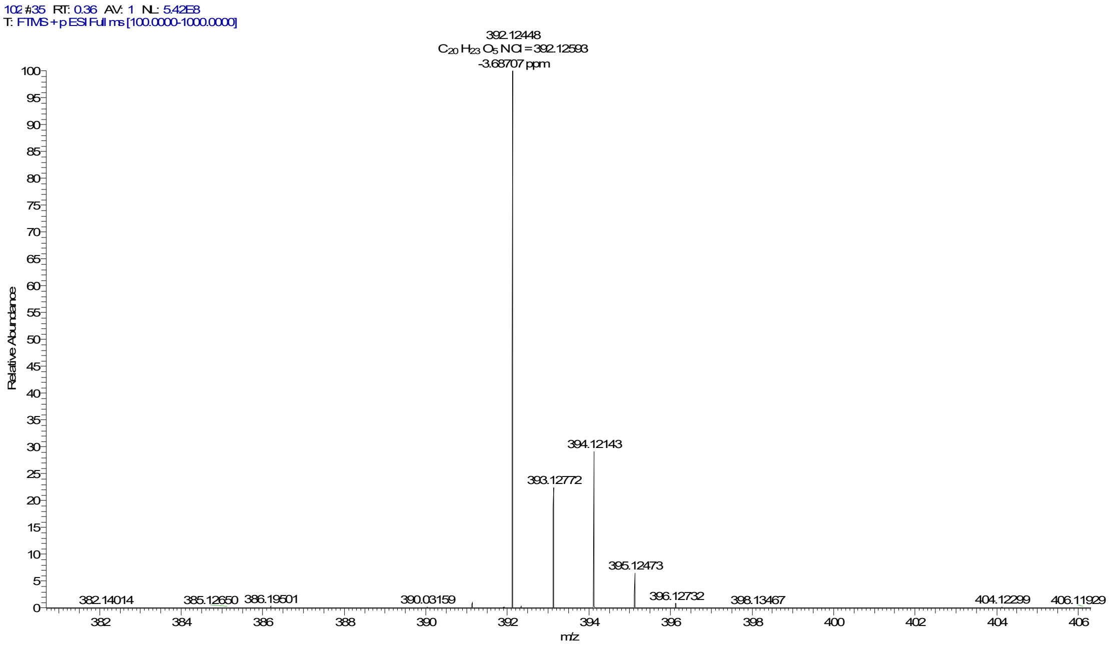

HRMS spectrum of target compound **A₂**.

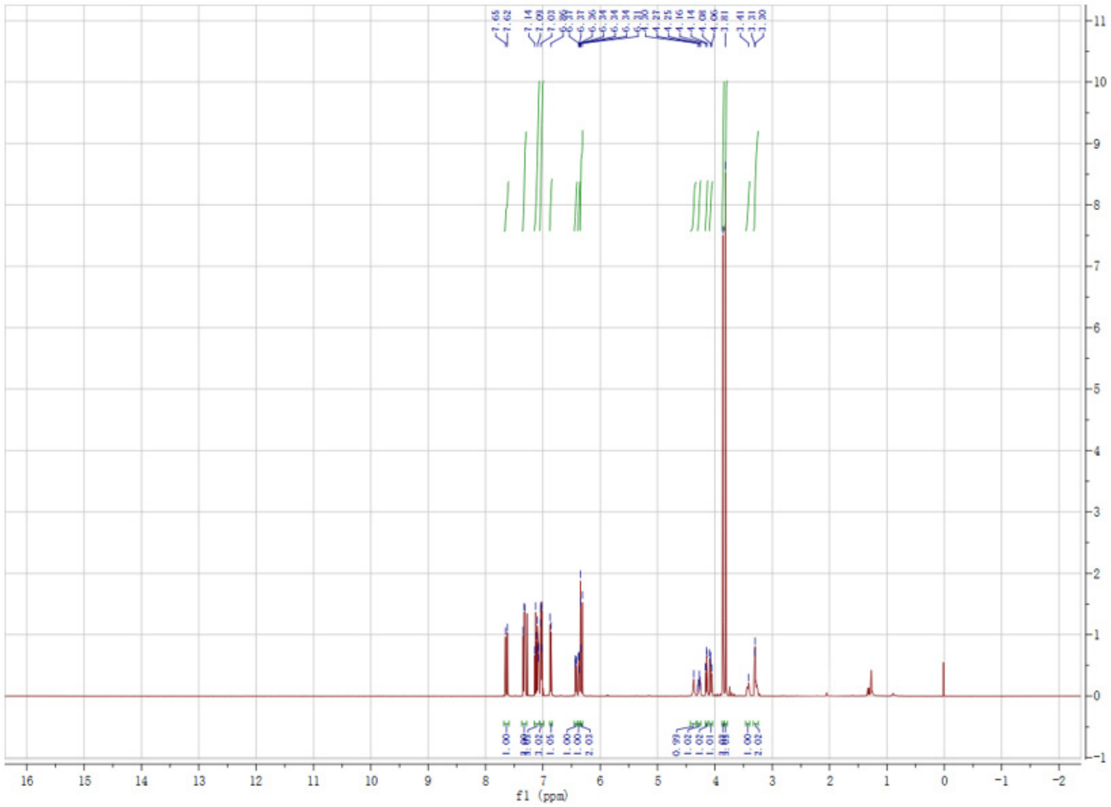

^{1}H NMR spectrum ($CDCl_3$, 500 MHz) of **A₃**.

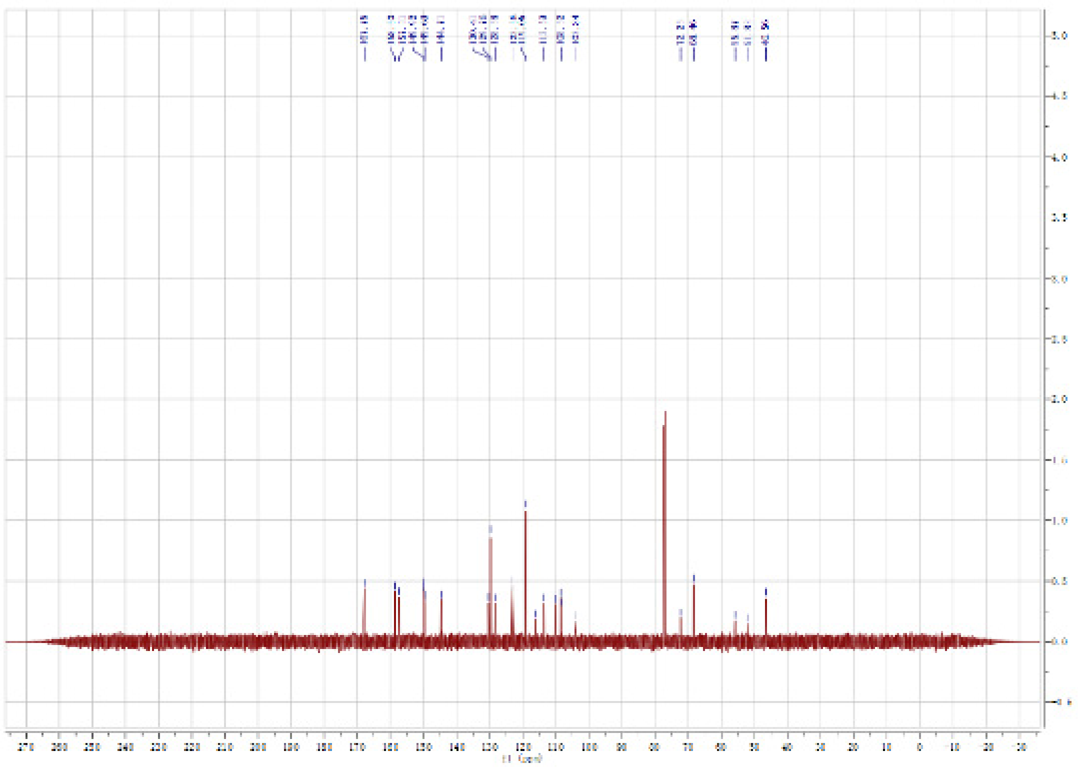

^{13}C NMR spectrum ($CDCl_3$, 126 MHz) of **A_3**.

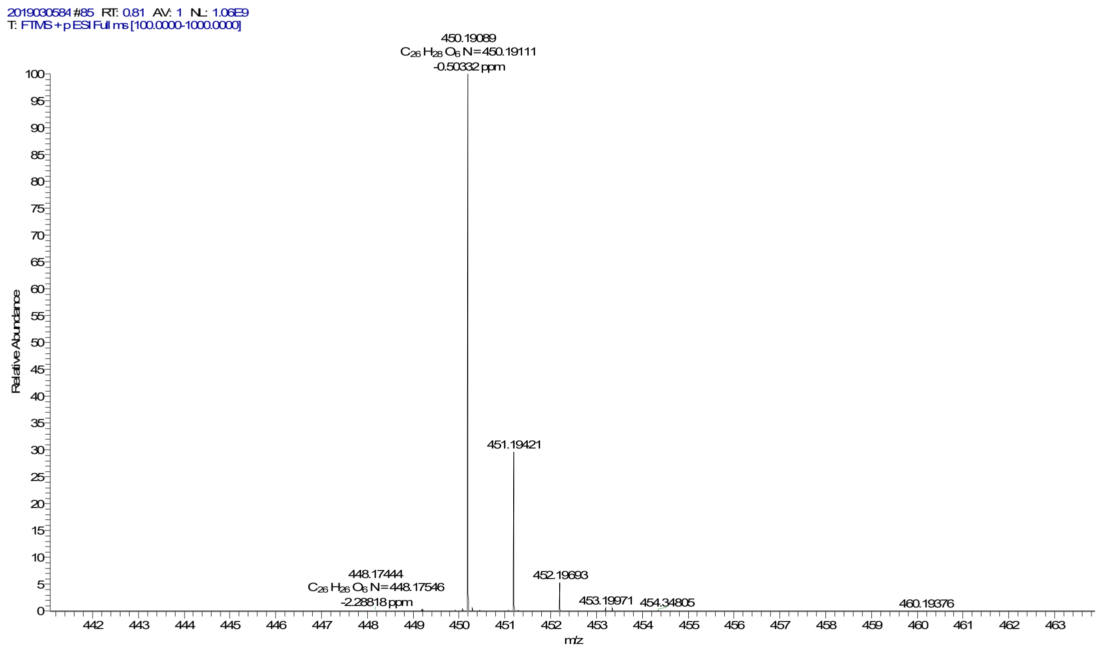

HRMS spectrum of target compound **A_3**.

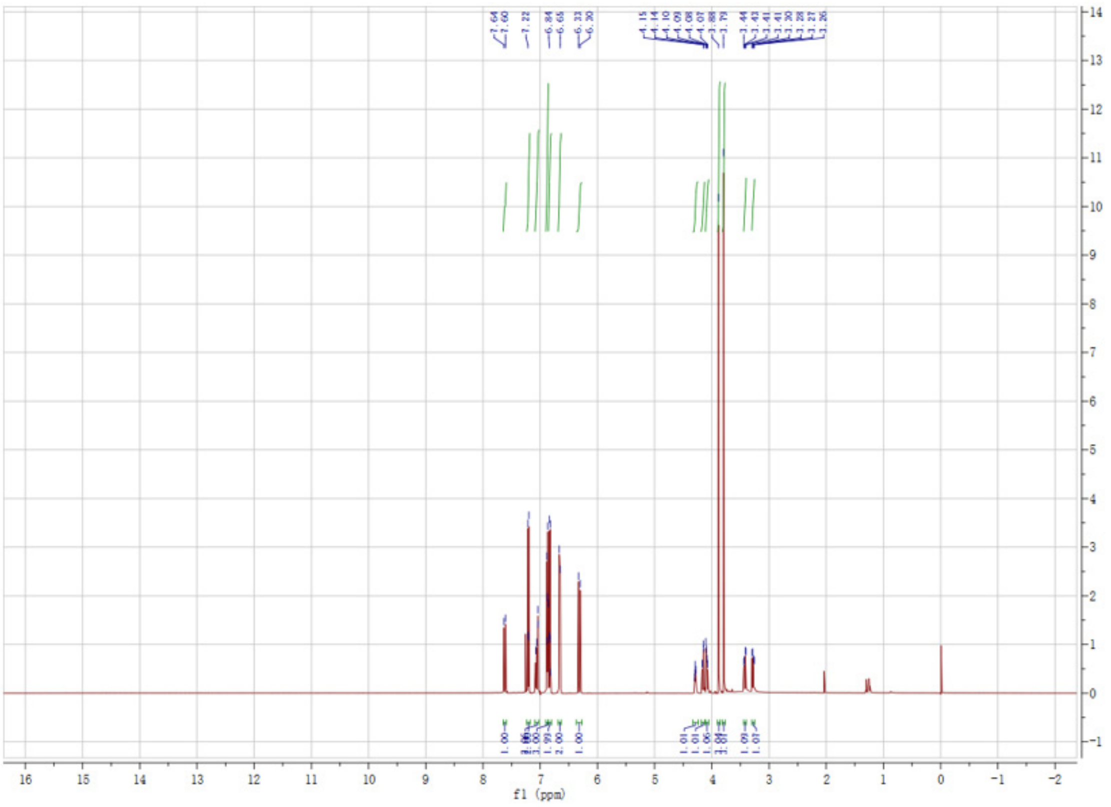

^{1}H NMR spectrum ($CDCl_3$, 500 MHz) of **A4**.

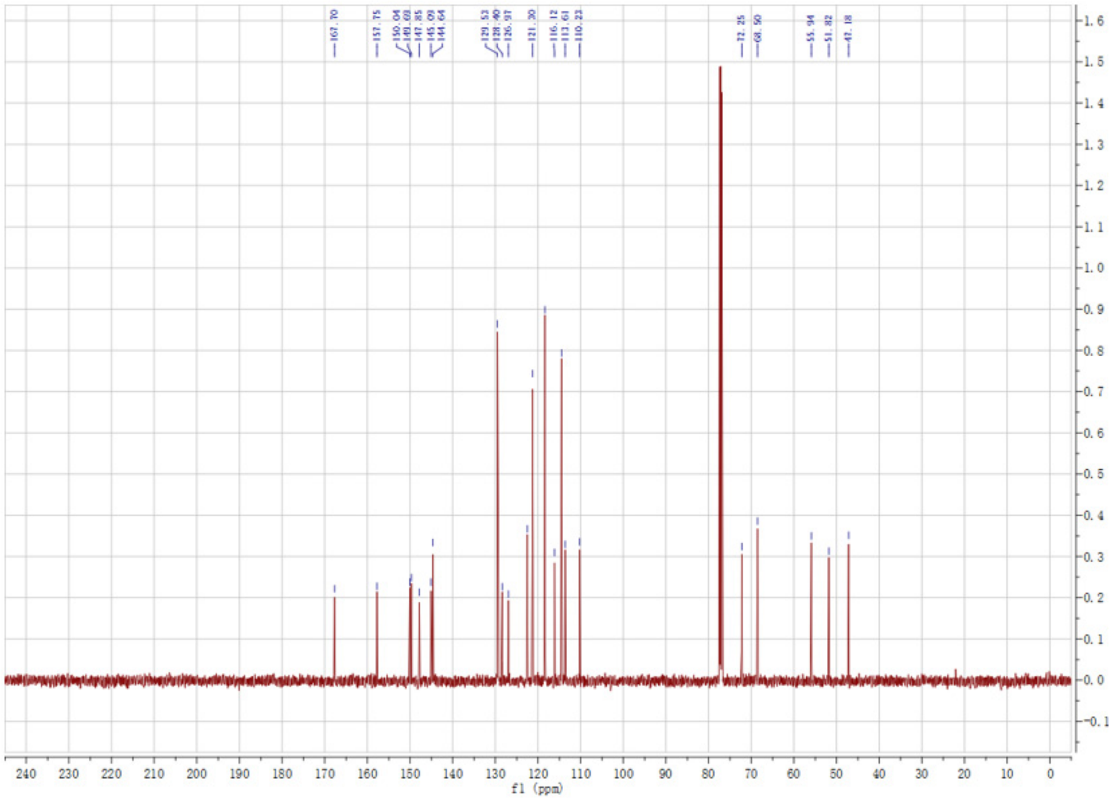

^{13}C NMR spectrum ($CDCl_3$, 126 MHz) of **A4**.

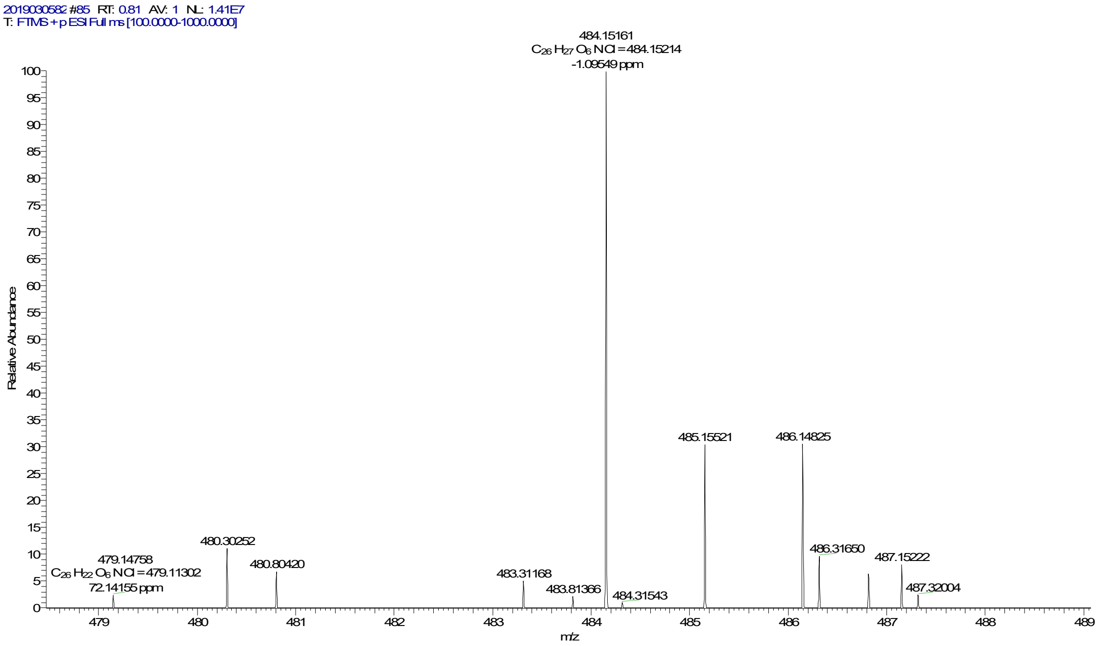

HRMS spectrum of target compound **A4**.

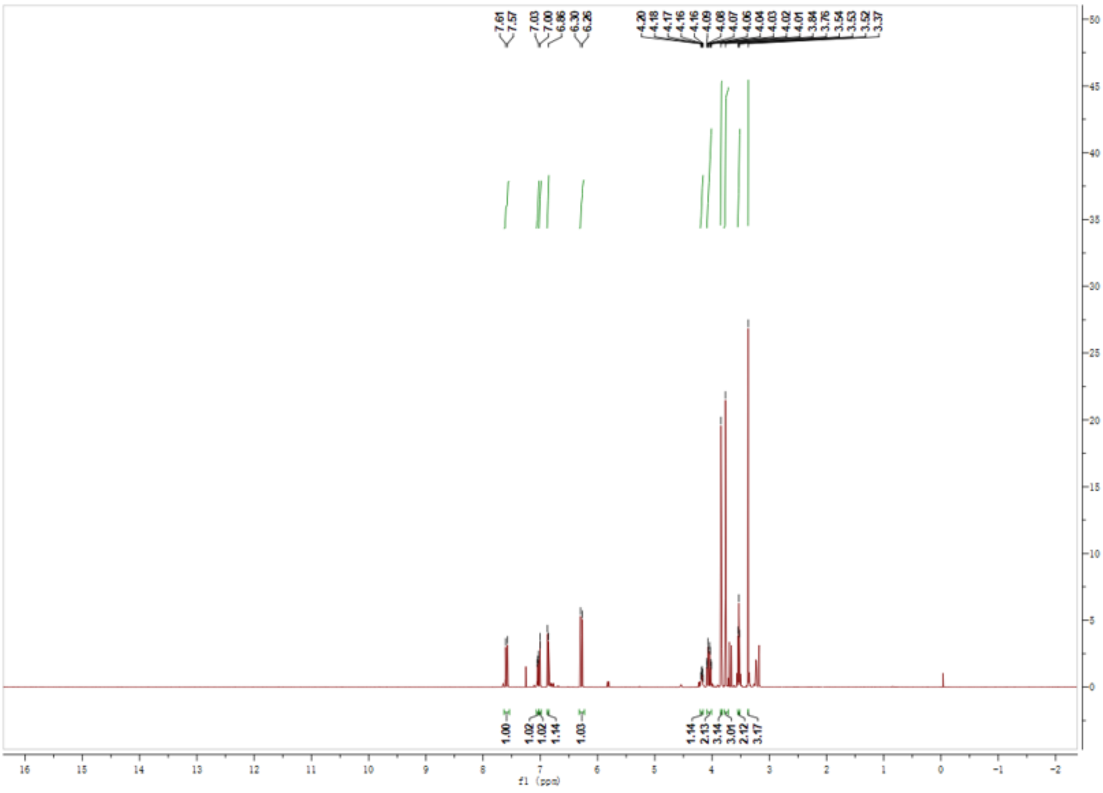

^{1}H NMR spectrum ($CDCl_3$, 500 MHz) of **A5**.

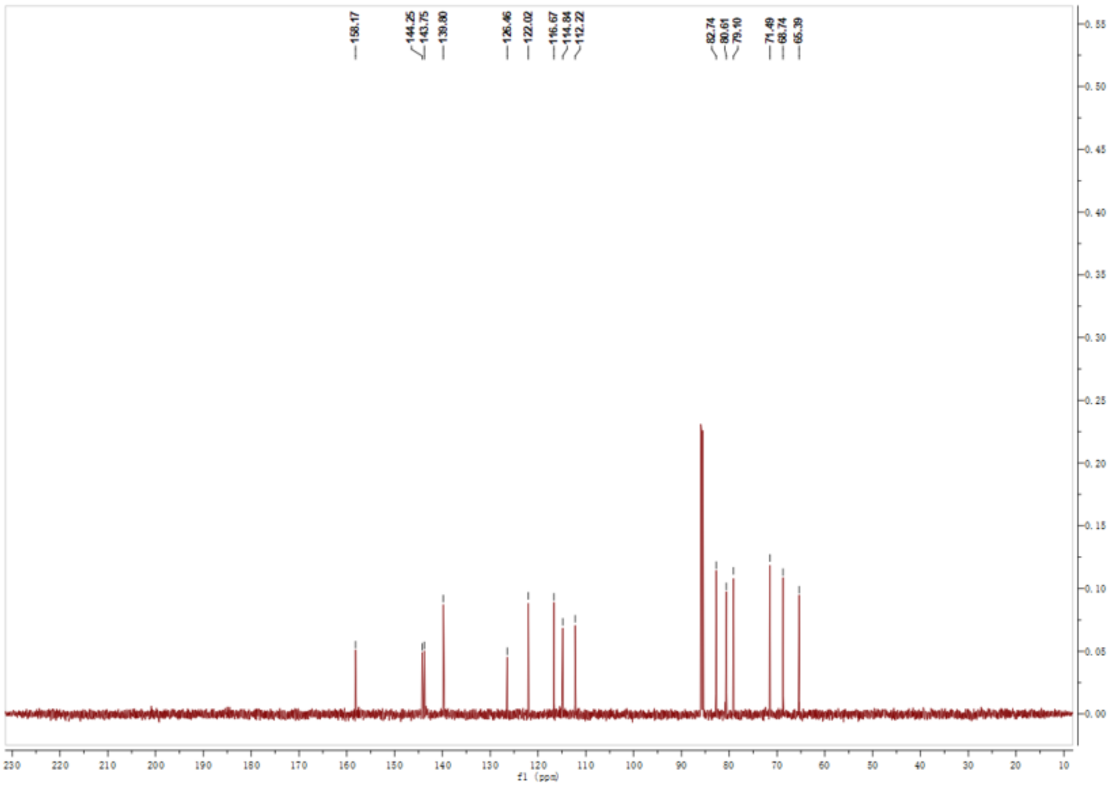

^{13}C NMR spectrum ($CDCl_3$, 126 MHz) of **A5**.

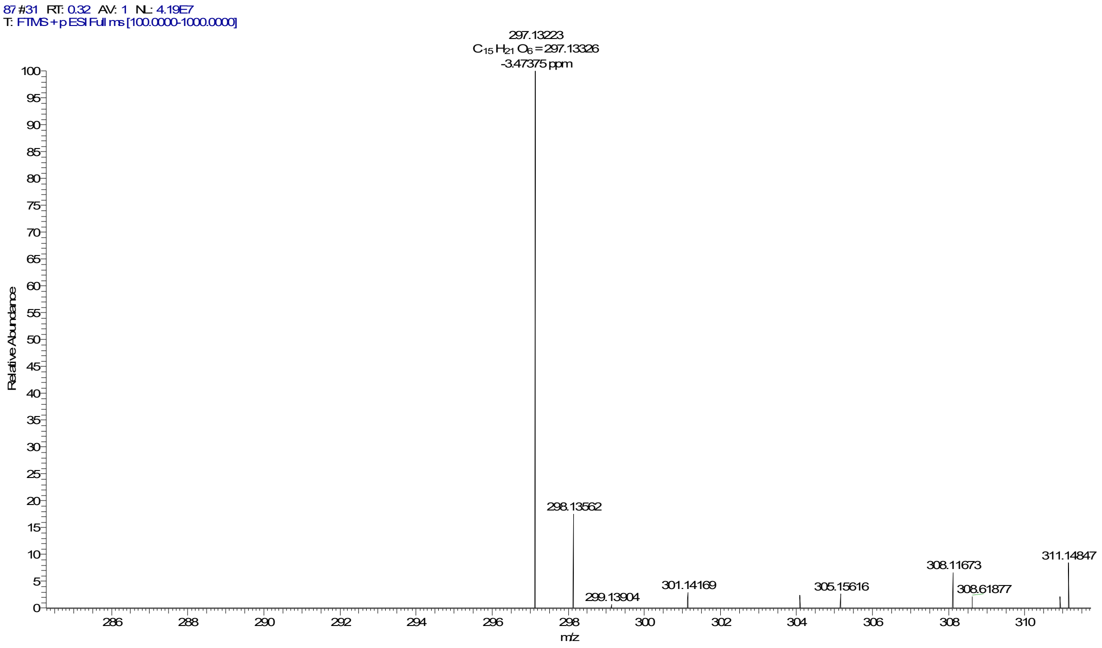

HRMS spectrum of target compound **A5**.

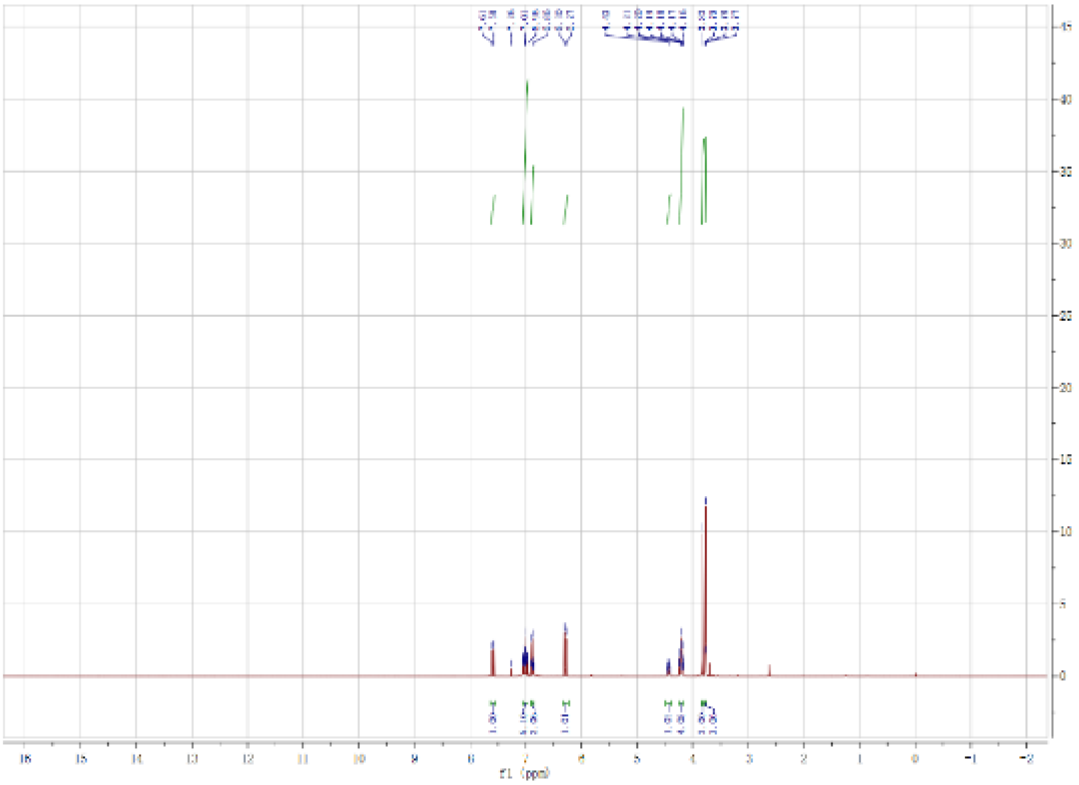

^{1}H NMR spectrum ($CDCl_3$, 500 MHz) of $\mathbf{A_6}$.

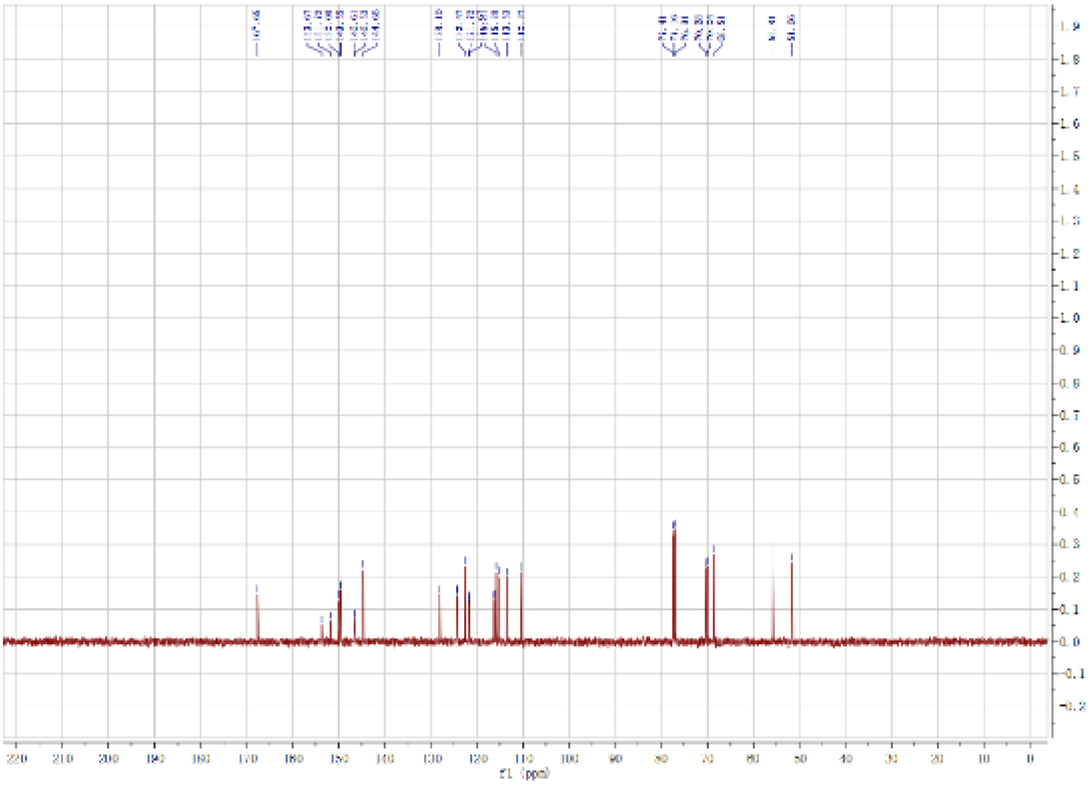

^{13}C NMR spectrum ($CDCl_3$, 126 MHz) of $\mathbf{A_6}$.

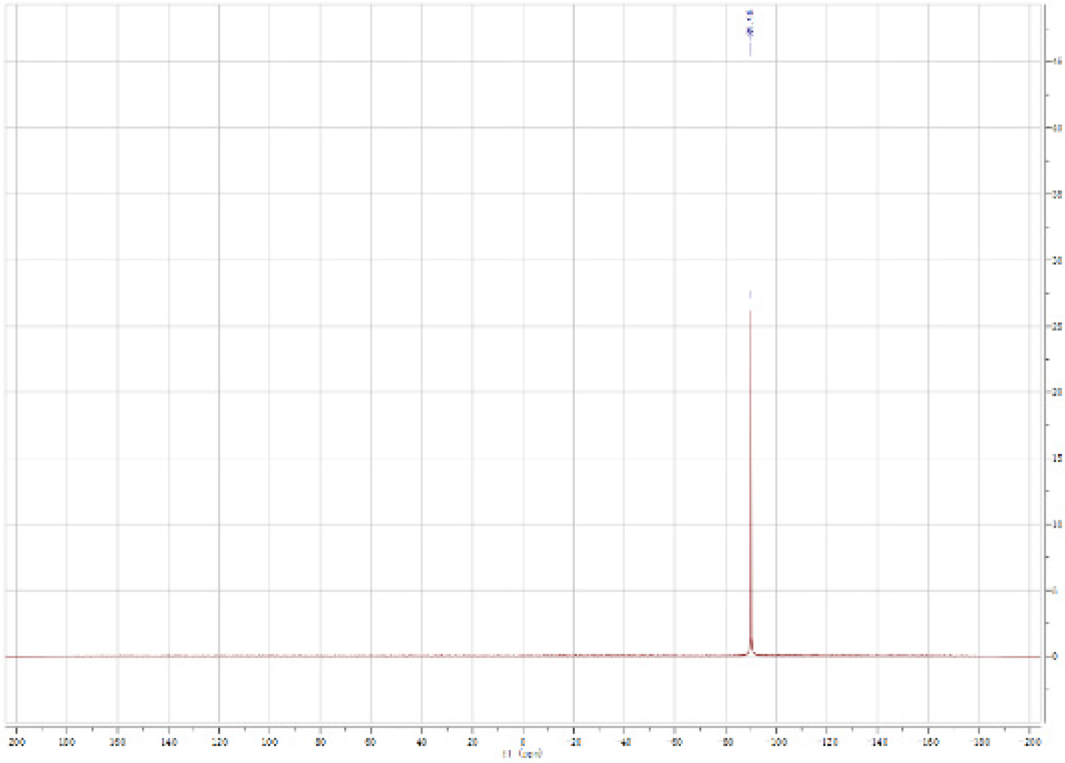

^{19}F NMR spectrum ($CDCl_3$, 471 MHz) of **A_6**.

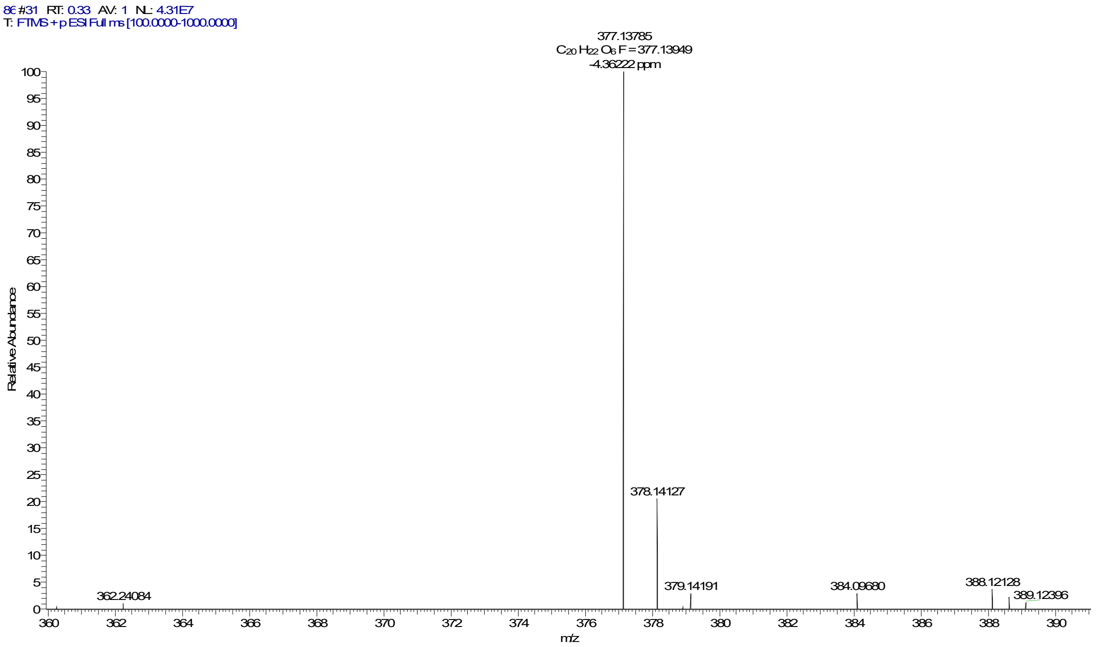

HRMS spectrum of target compound **A_6**.

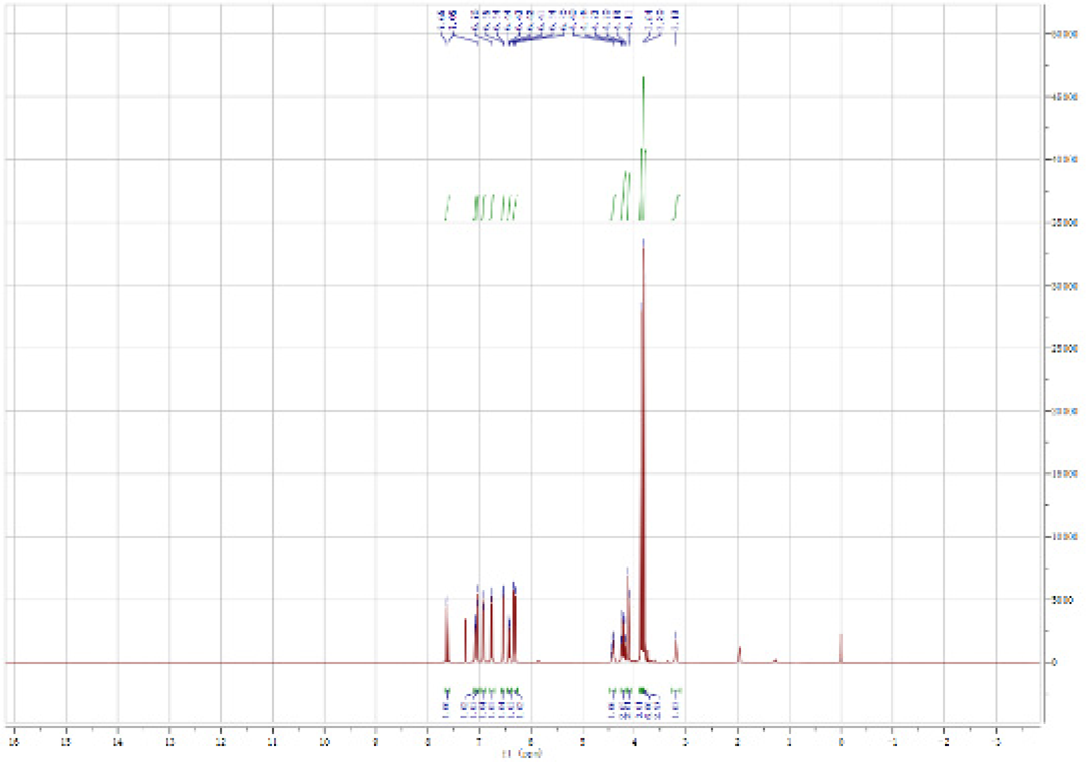

^{1}H NMR spectrum ($CDCl_3$, 500 MHz) of **A$_7$**.

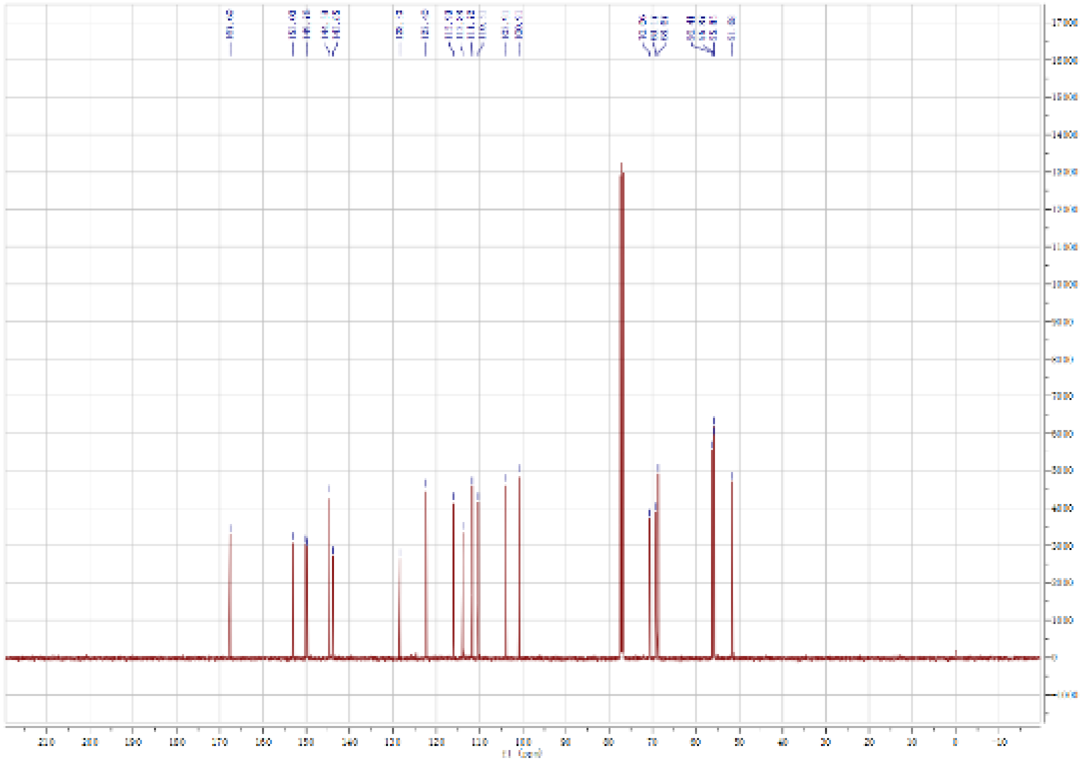

^{13}C NMR spectrum ($CDCl_3$, 126 MHz) of **A$_7$**.

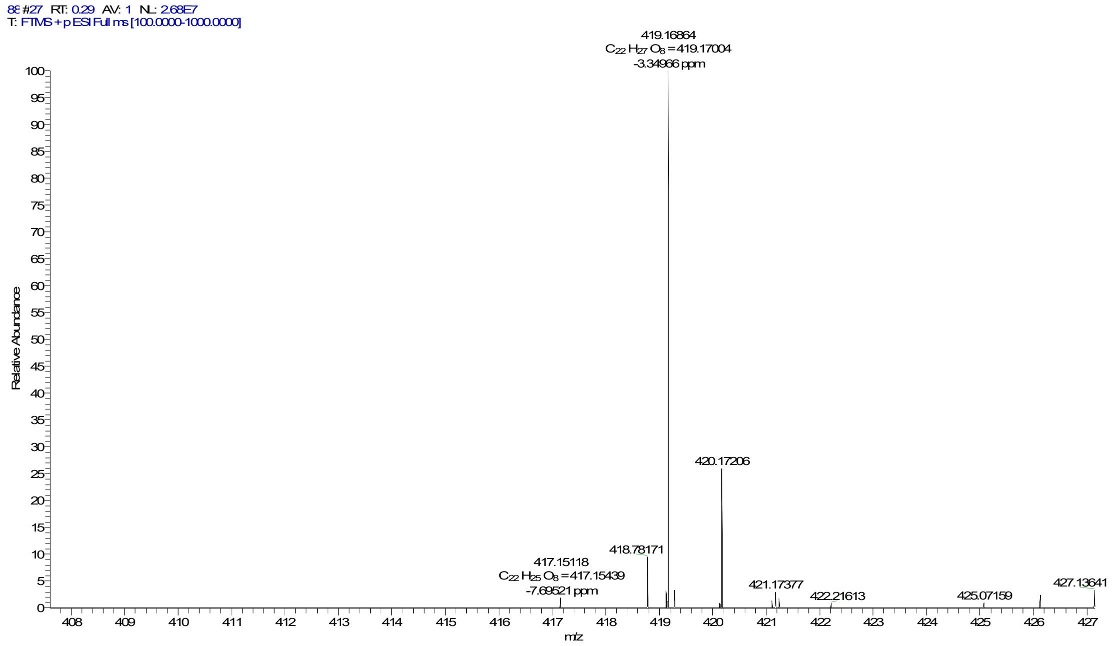

HRMS spectrum of target compound **A$_7$**.

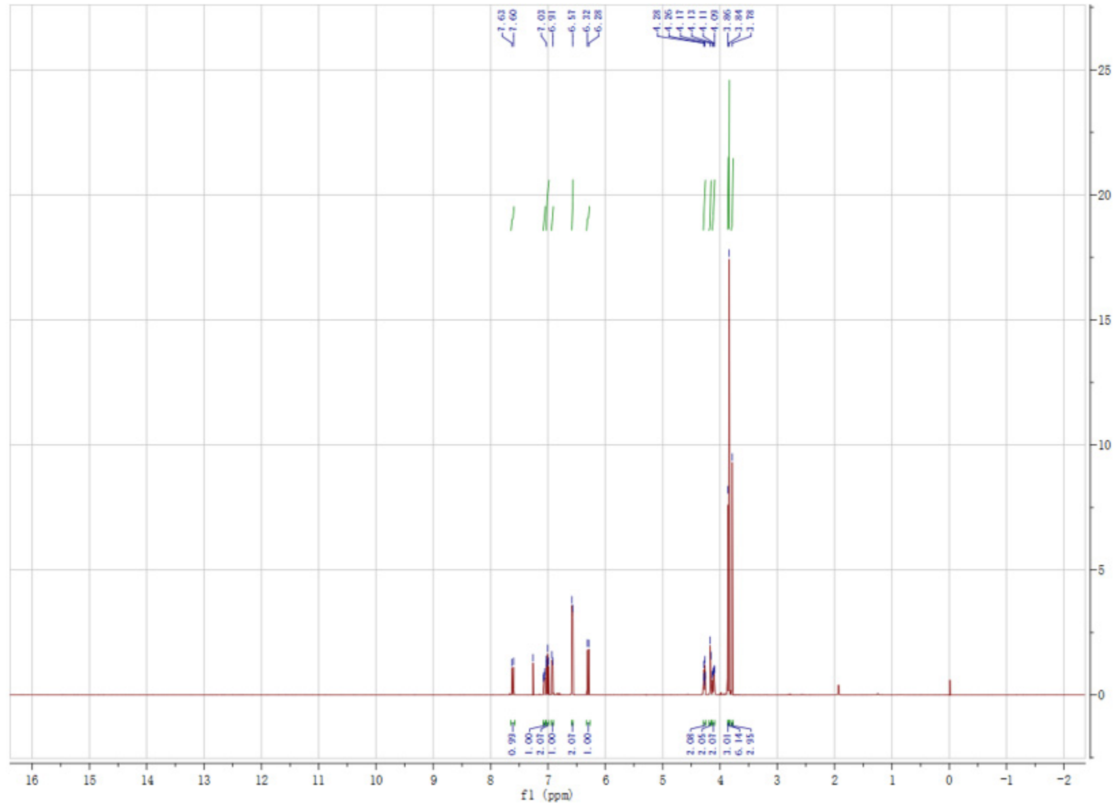

^{1}H NMR spectrum ($CDCl_3$, 500 MHz) of **A$_8$**.

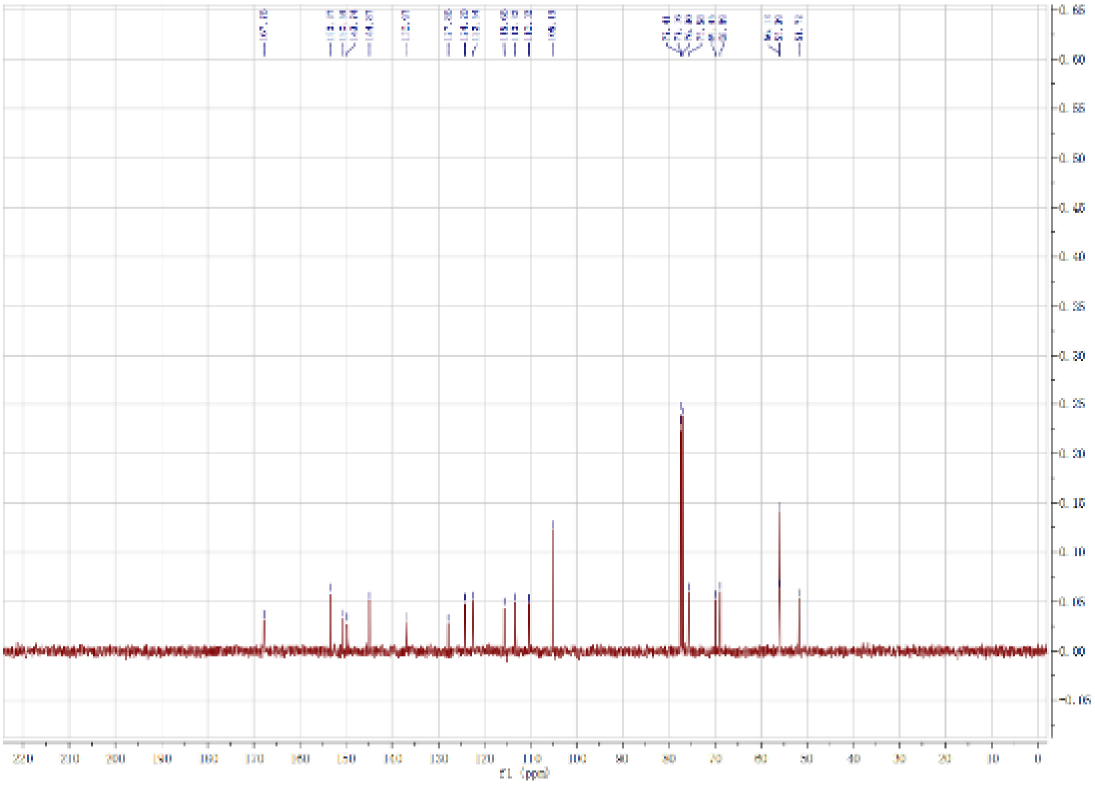

^{13}C NMR spectrum (CDCl$_3$, 126 MHz) of **A$_8$**.

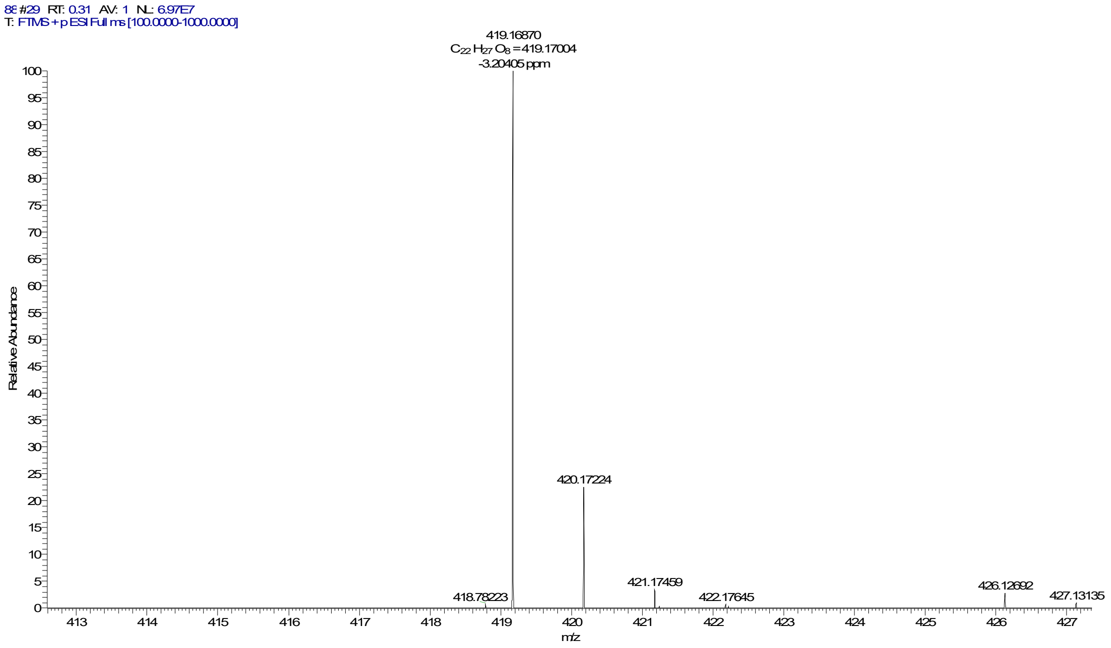

HRMS spectrum of target compound **A$_8$**.

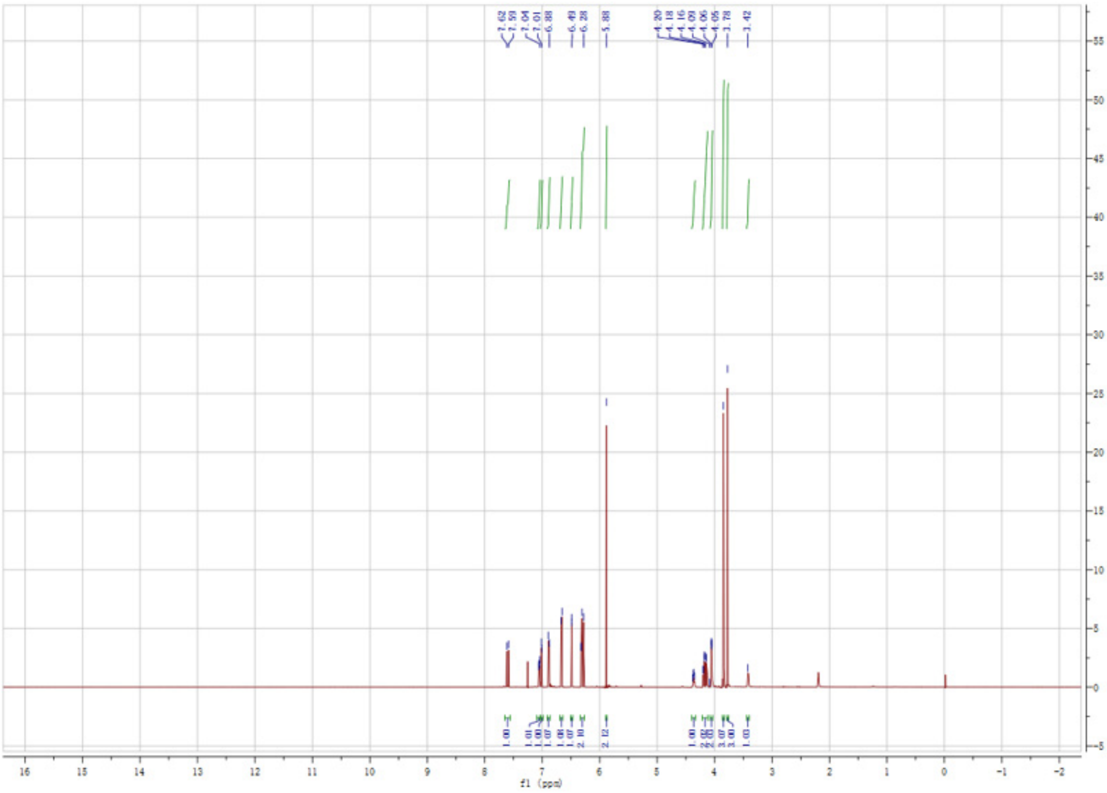

^{1}H NMR spectrum ($CDCl_3$, 500 MHz) of $\mathbf{A_9}$.

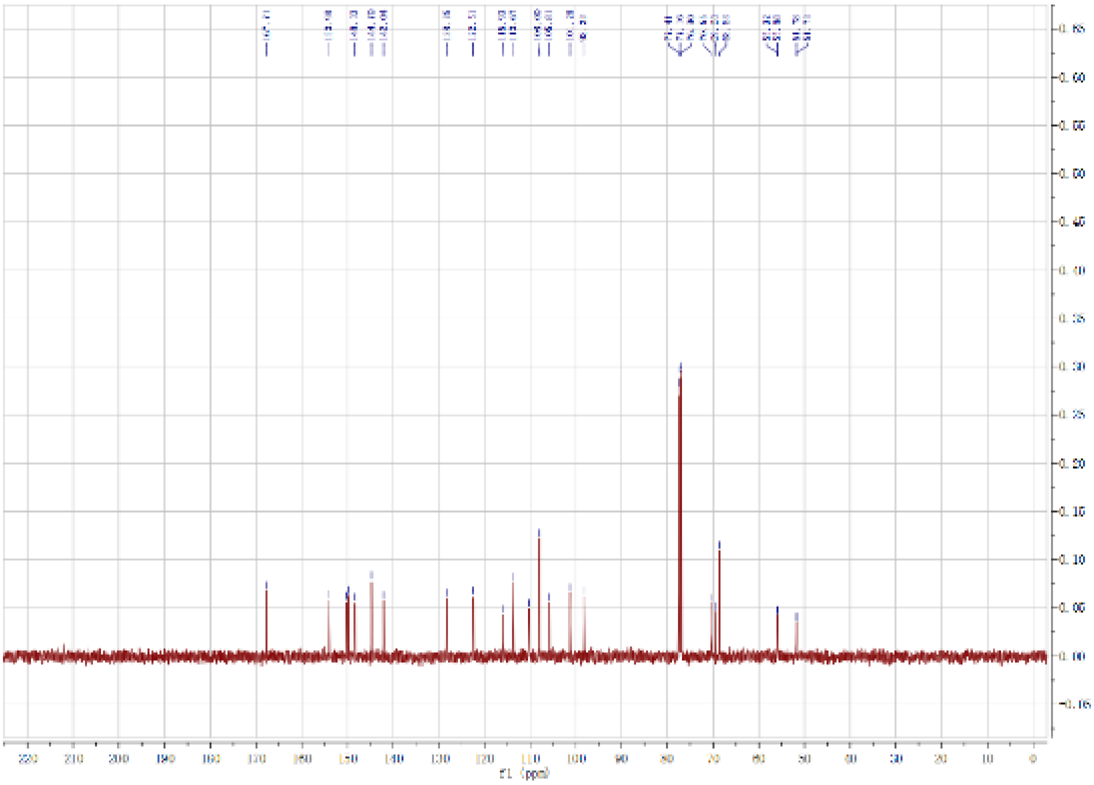

^{13}C NMR spectrum ($CDCl_3$, 126 MHz) of $\mathbf{A_9}$.

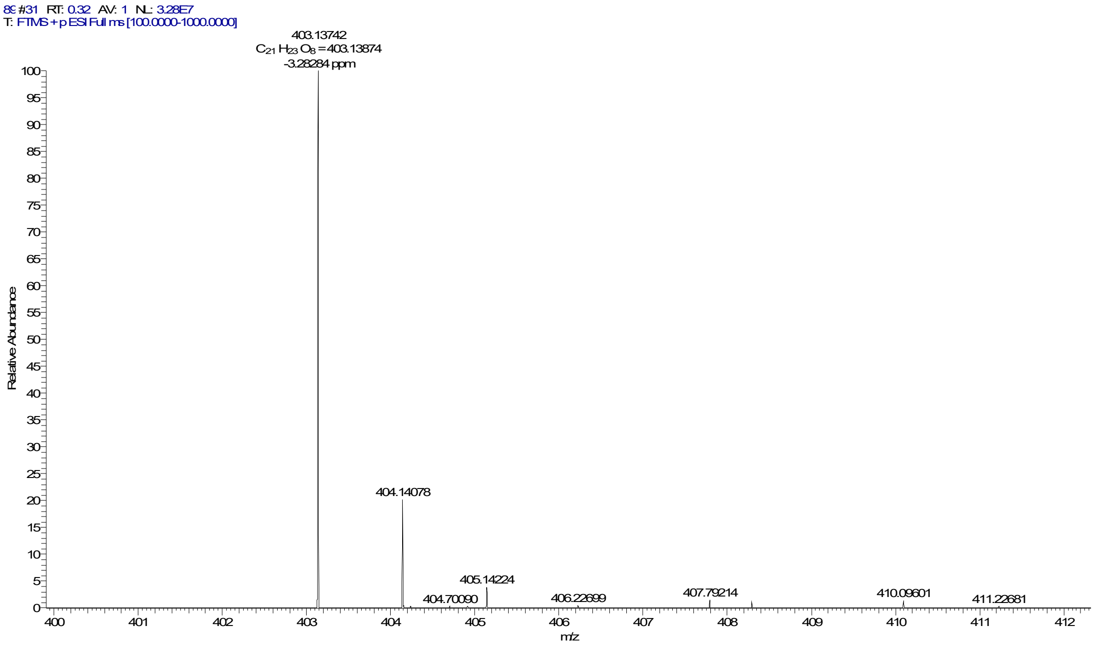

HRMS spectrum of target compound **A$_9$**.

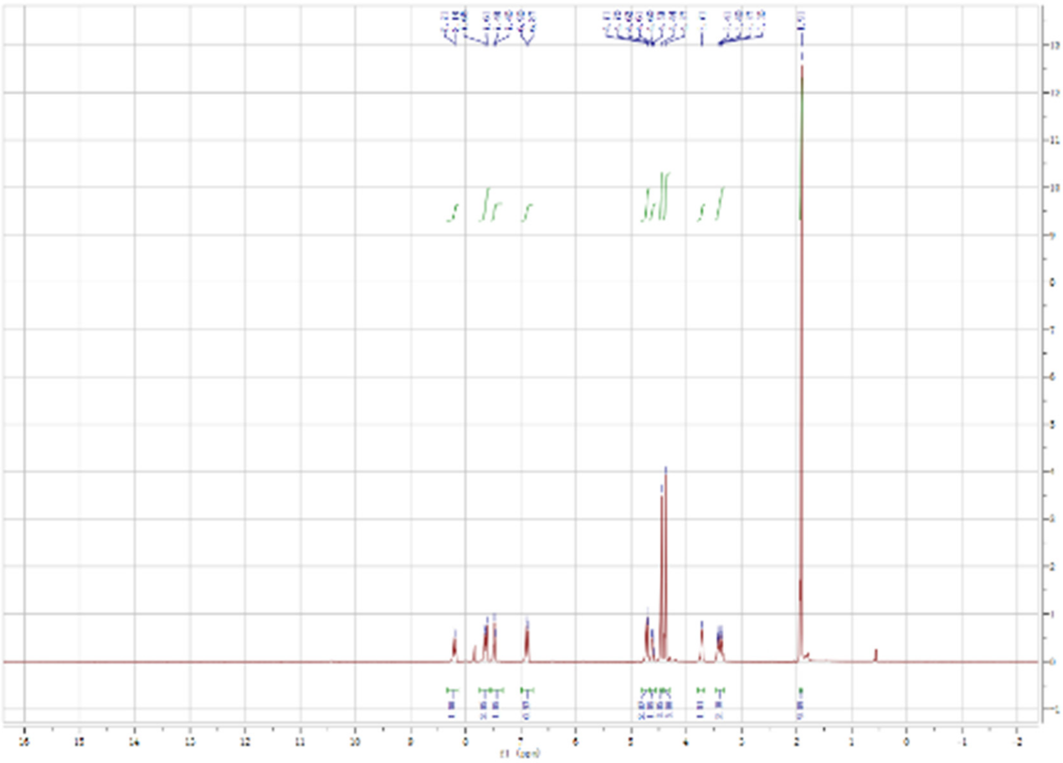

^{1}H NMR spectrum (CDCl$_3$, 500 MHz) of **A$_{10}$**.

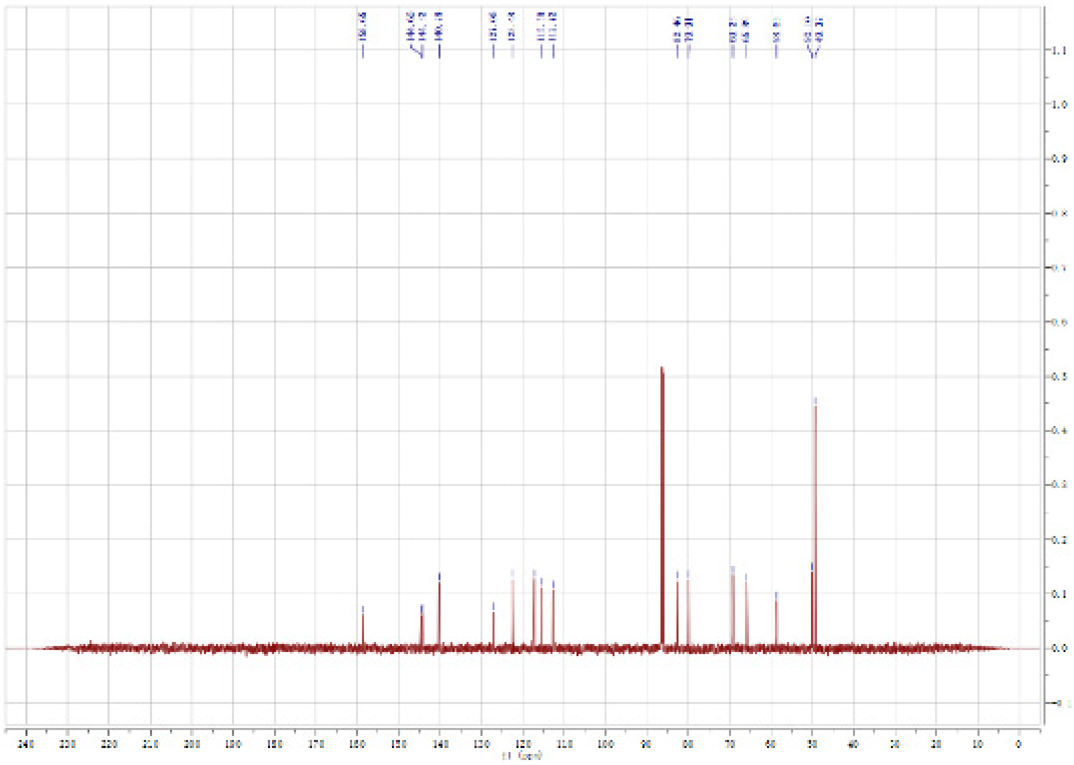

^{13}C NMR spectrum ($CDCl_3$, 126 MHz) of **A10**.

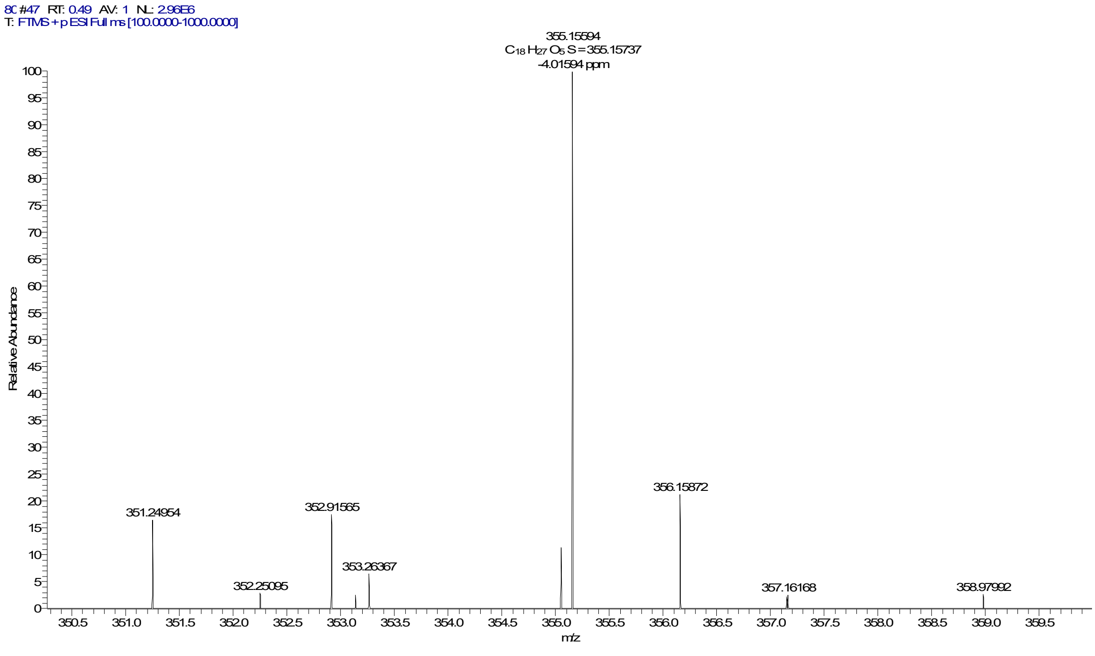

HRMS spectrum of target compound **A10**.

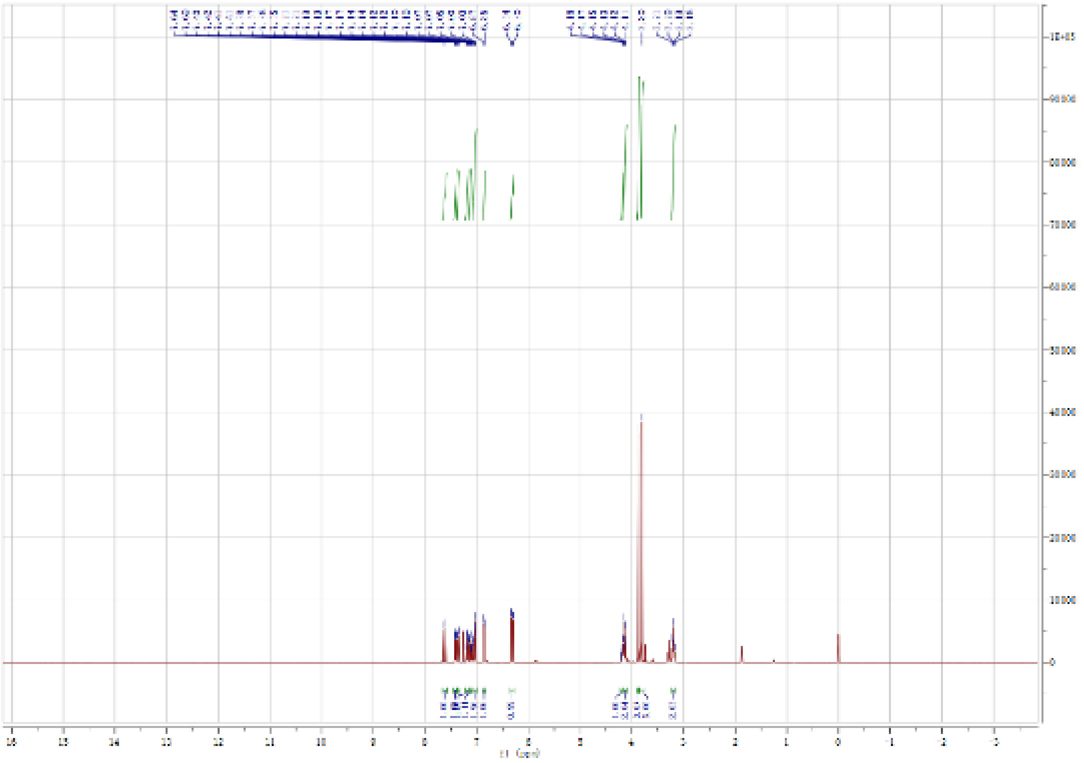

^{1}H NMR spectrum (CDCl$_3$, 400 MHz) of **A$_{11}$**.

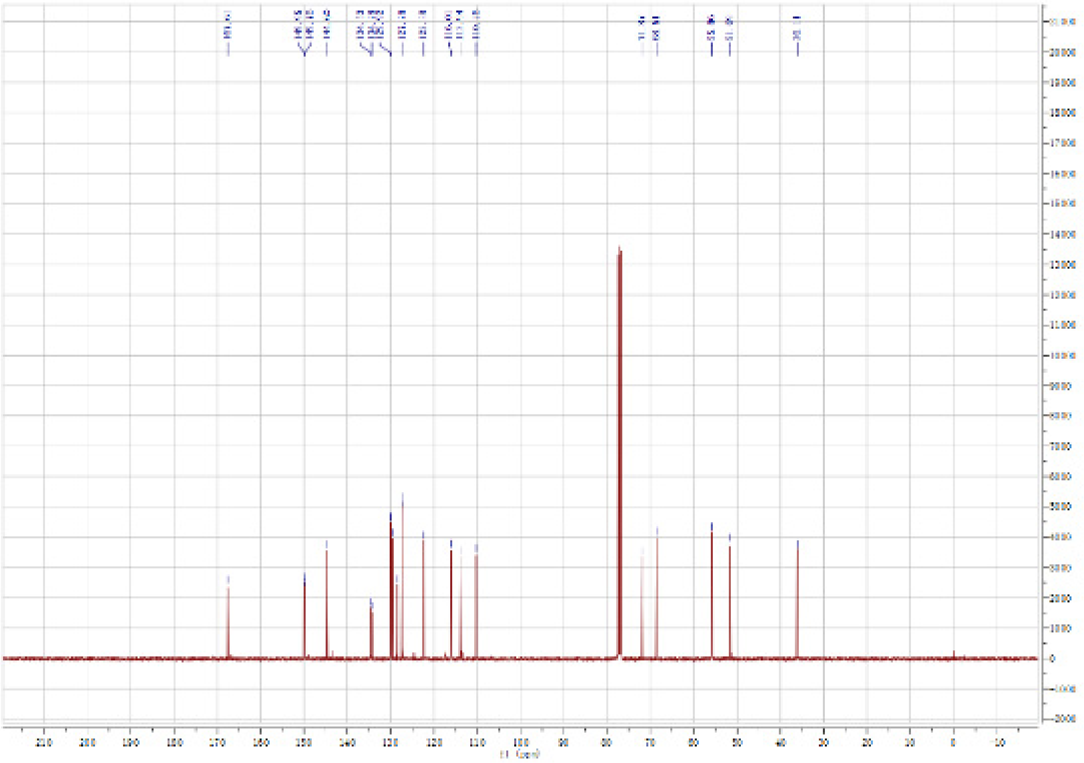

^{13}C NMR spectrum (CDCl$_3$, 101 MHz) of **A$_{11}$**.

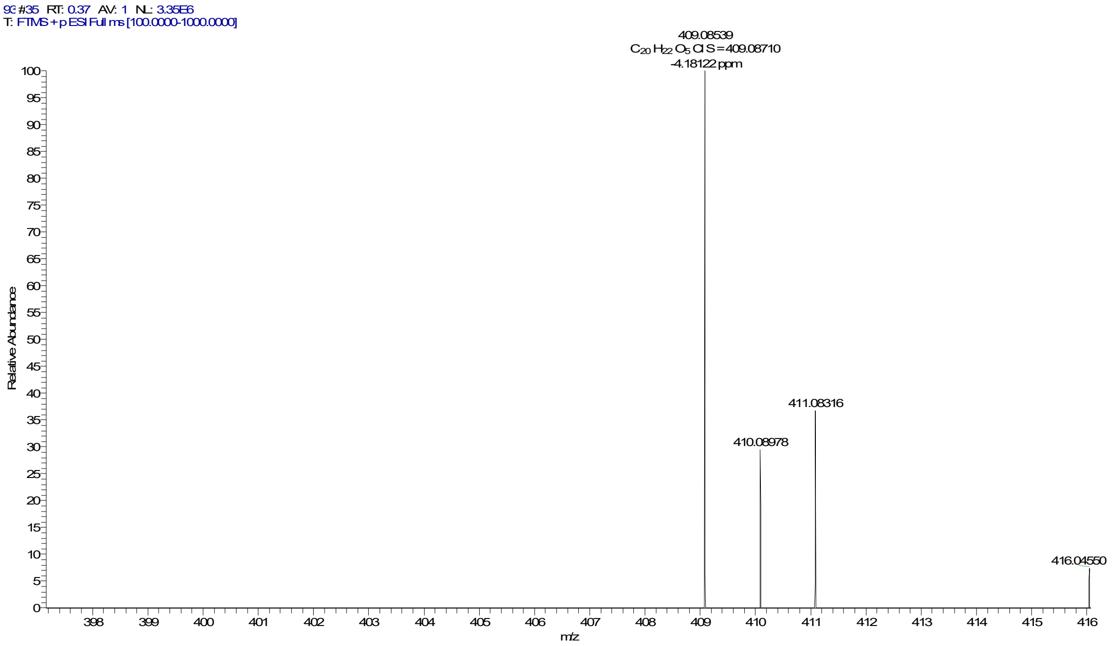

HRMS spectrum of target compound **A$_{11}$**.

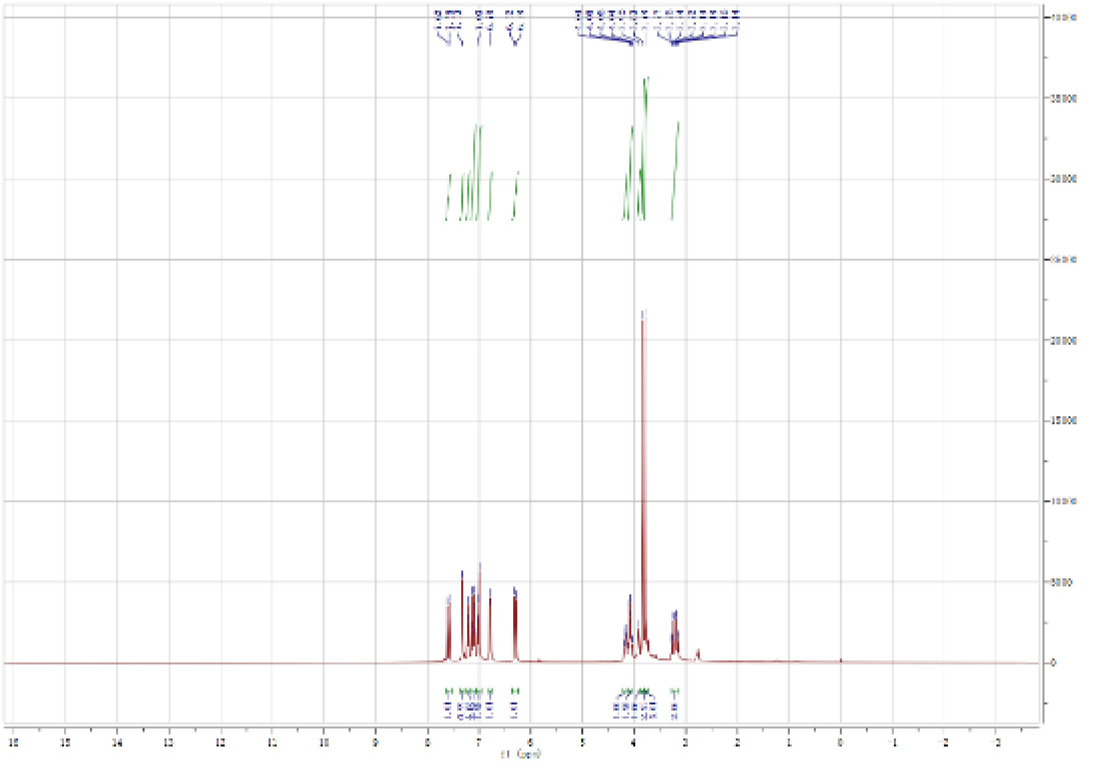

^{1}H NMR spectrum ($CDCl_3$, 400 MHz) of **A$_{12}$**.

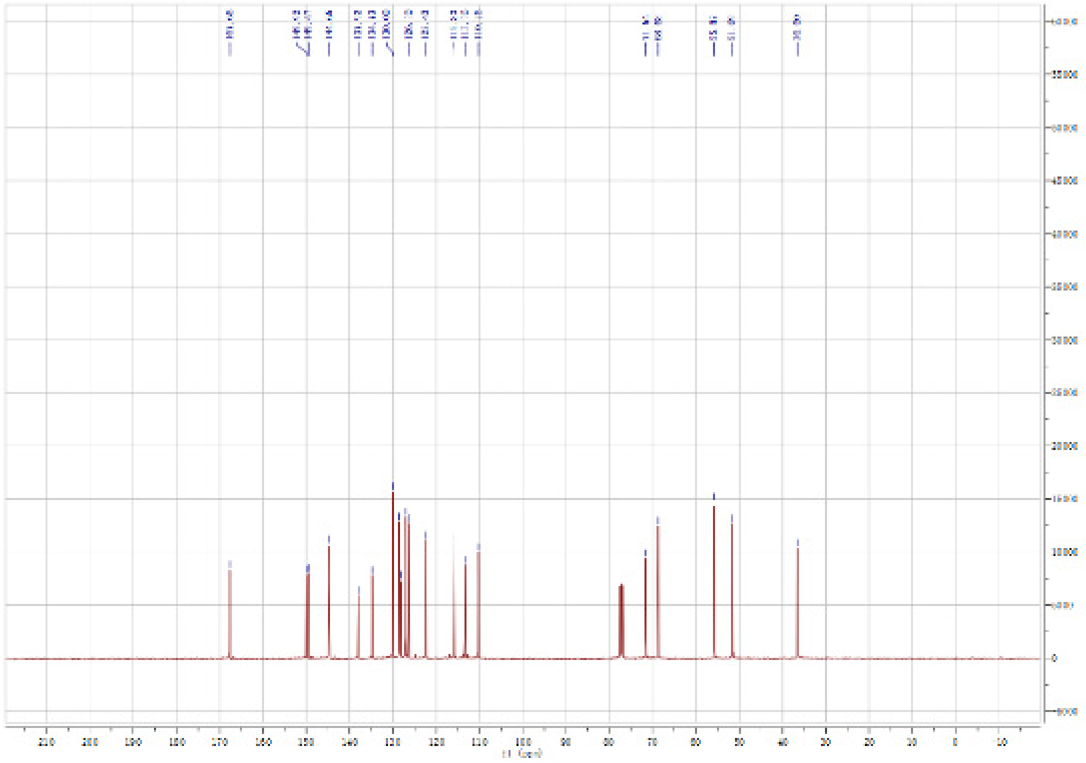

^{13}C NMR spectrum ($CDCl_3$, 101 MHz) of **A12**.

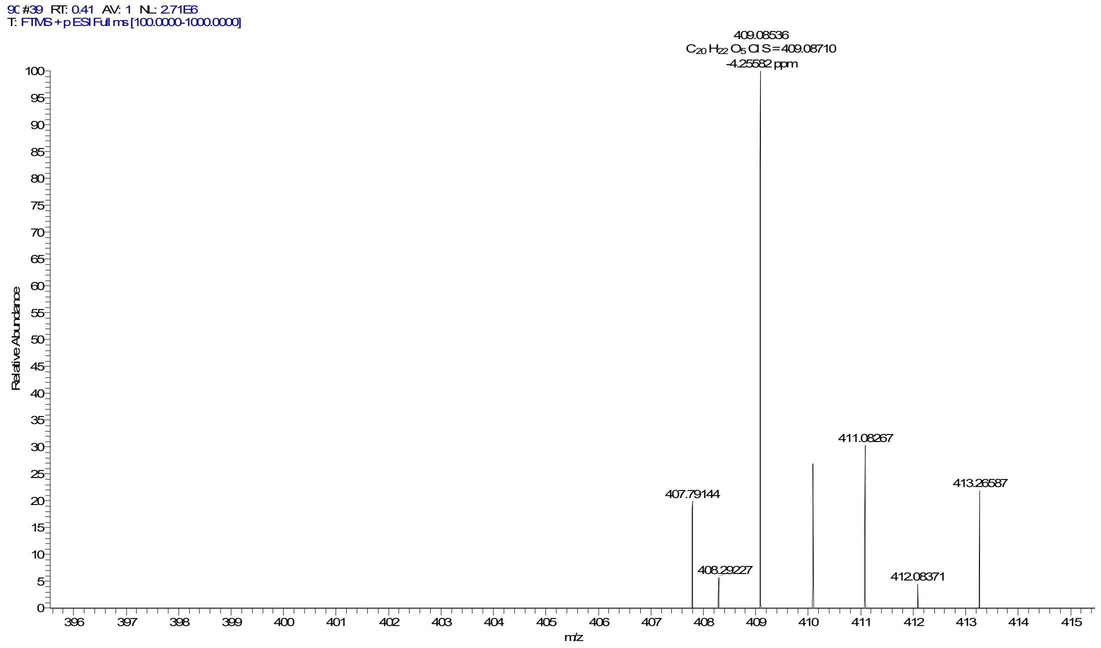

HRMS spectrum of target compound **A12**.

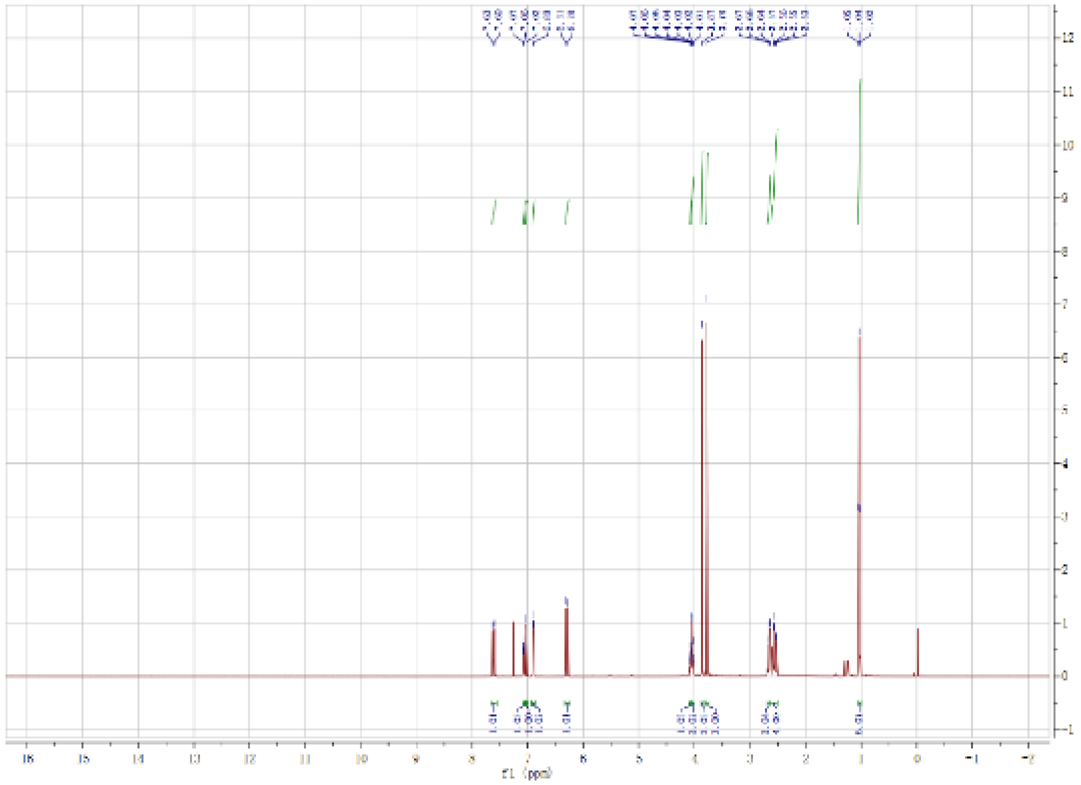

^{1}H NMR spectrum ($CDCl_3$, 500 MHz) of $\mathbf{A_{13}}$.

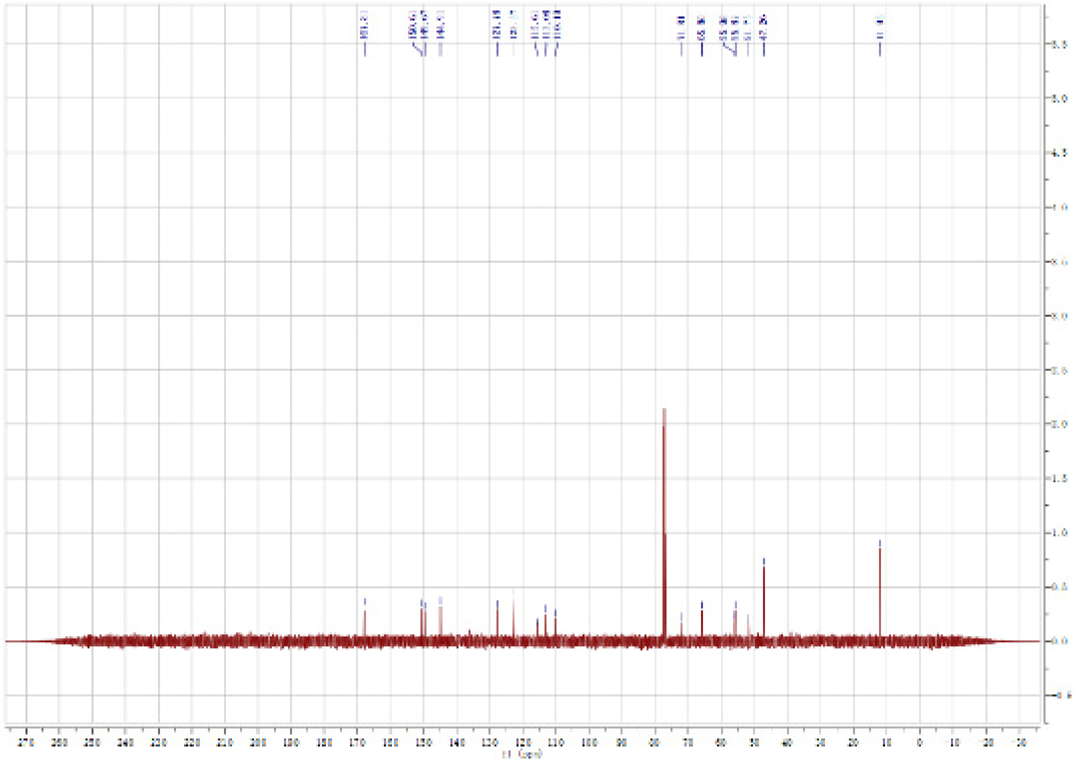

^{13}C NMR spectrum ($CDCl_3$, 126 MHz) of $\mathbf{A_{13}}$.

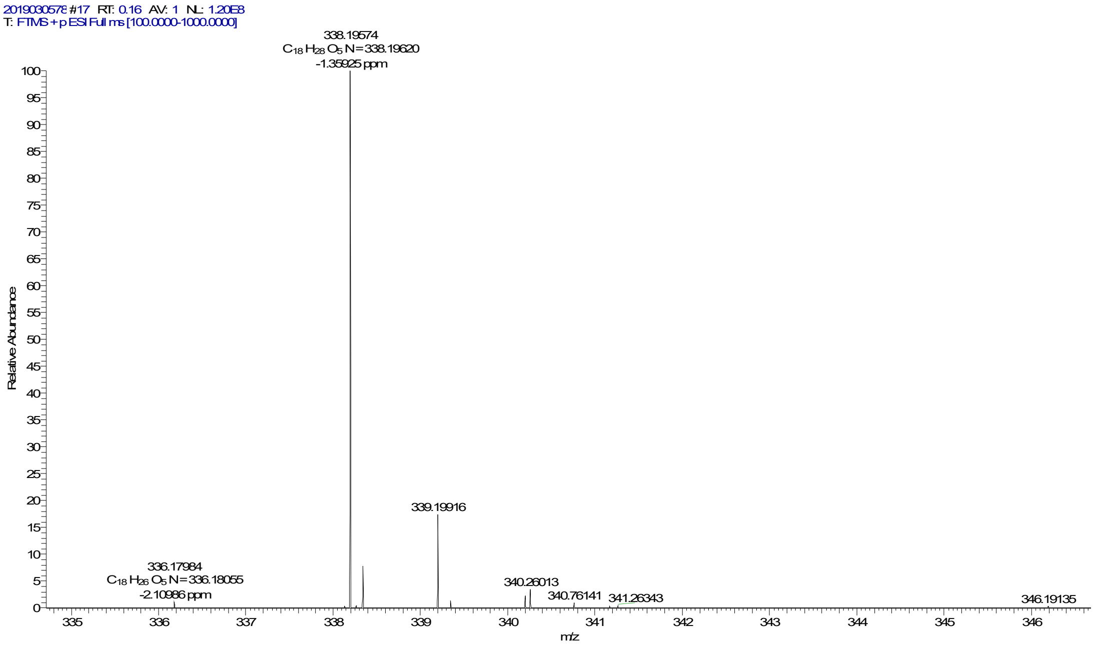

HRMS spectrum of target compound **A13**.

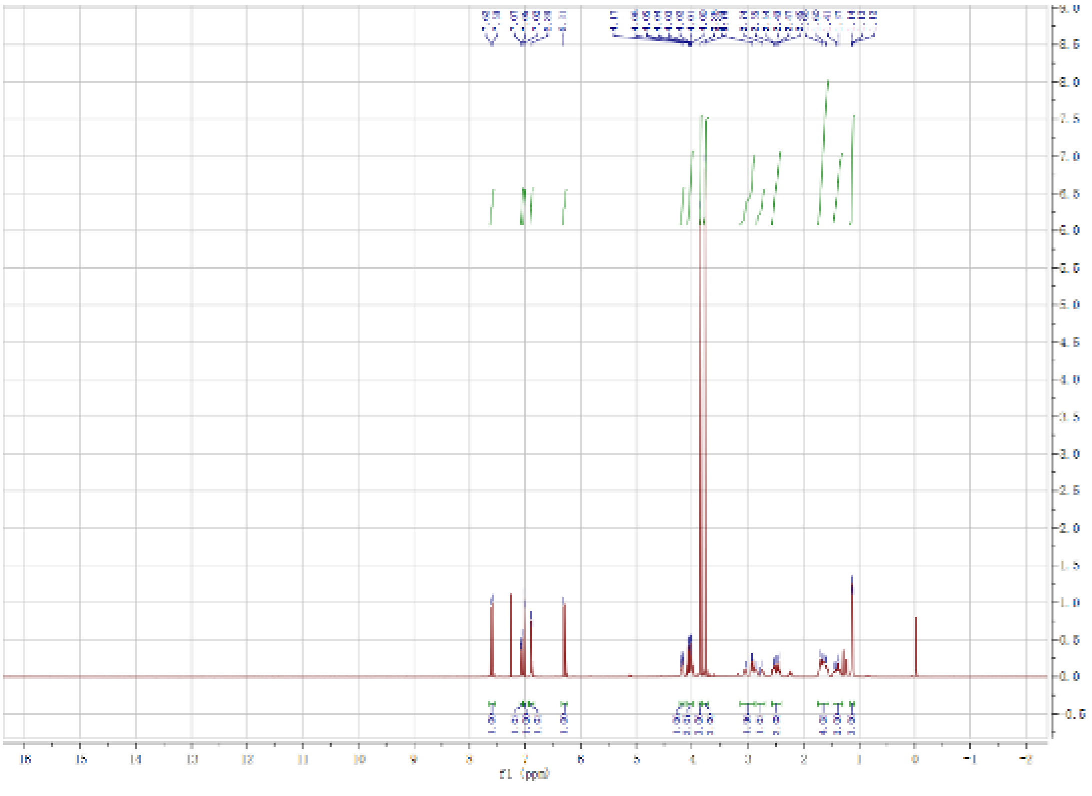

^{1}H NMR spectrum ($CDCl_3$, 500 MHz) of **A14**.

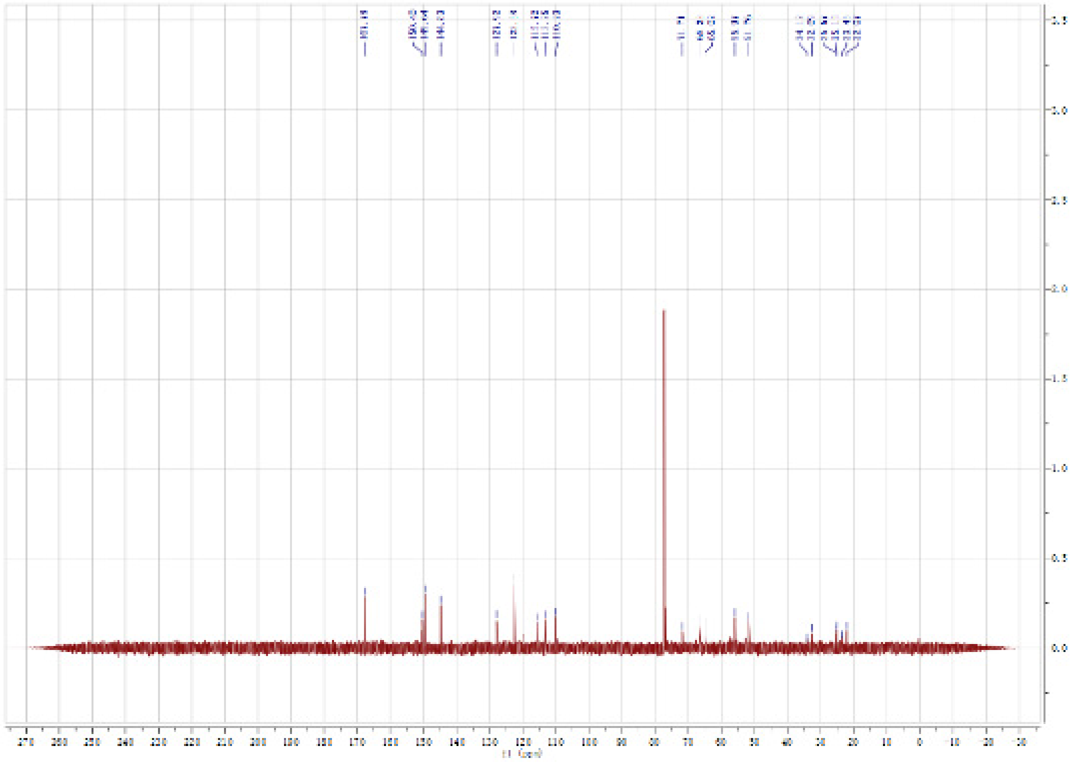

^{13}C NMR spectrum (CDCl$_3$, 126 MHz) of **A$_{14}$**.

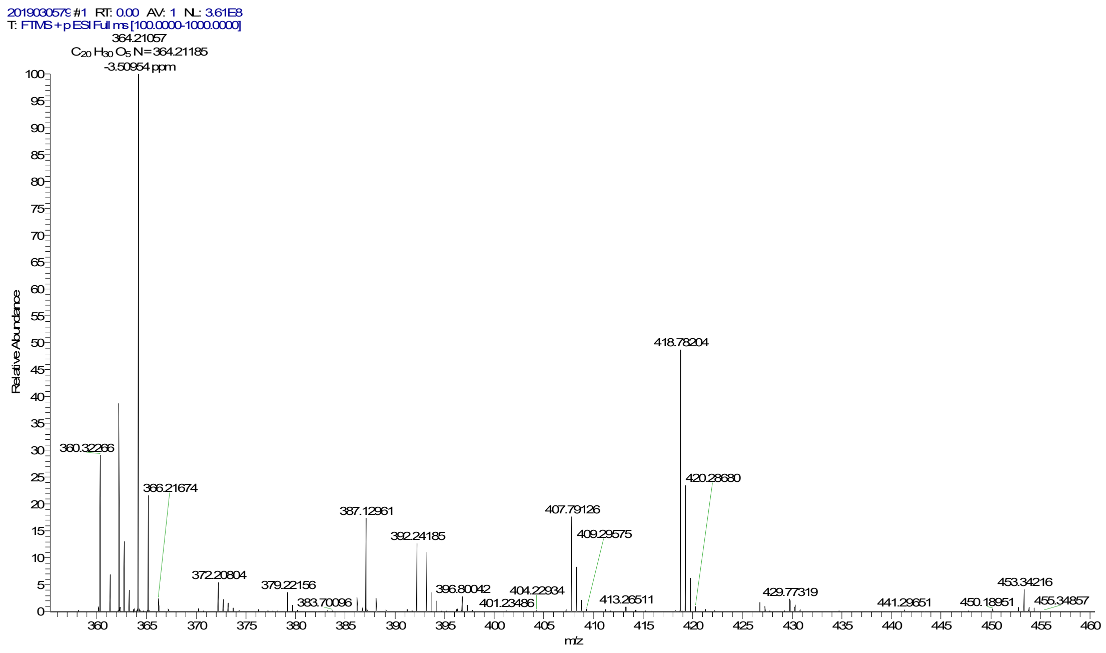

HRMS spectrum of target compound **A$_{14}$**.

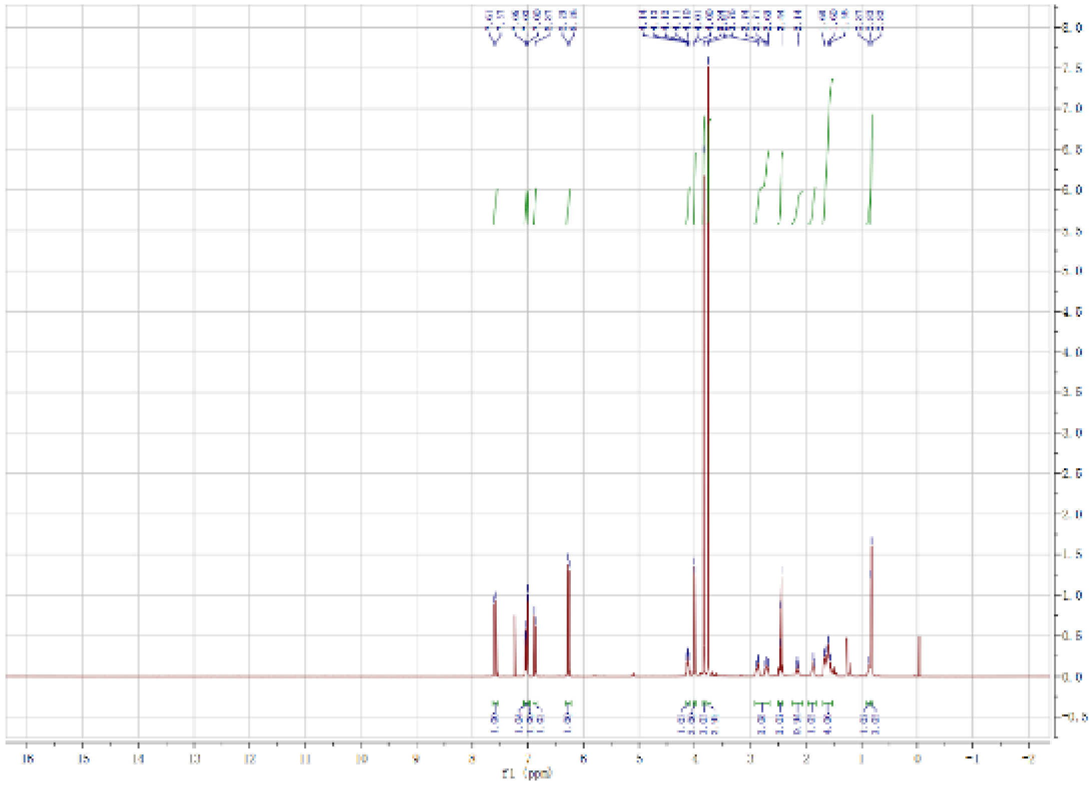

^{1}H NMR spectrum ($CDCl_3$, 500 MHz) of **A$_{15}$**.

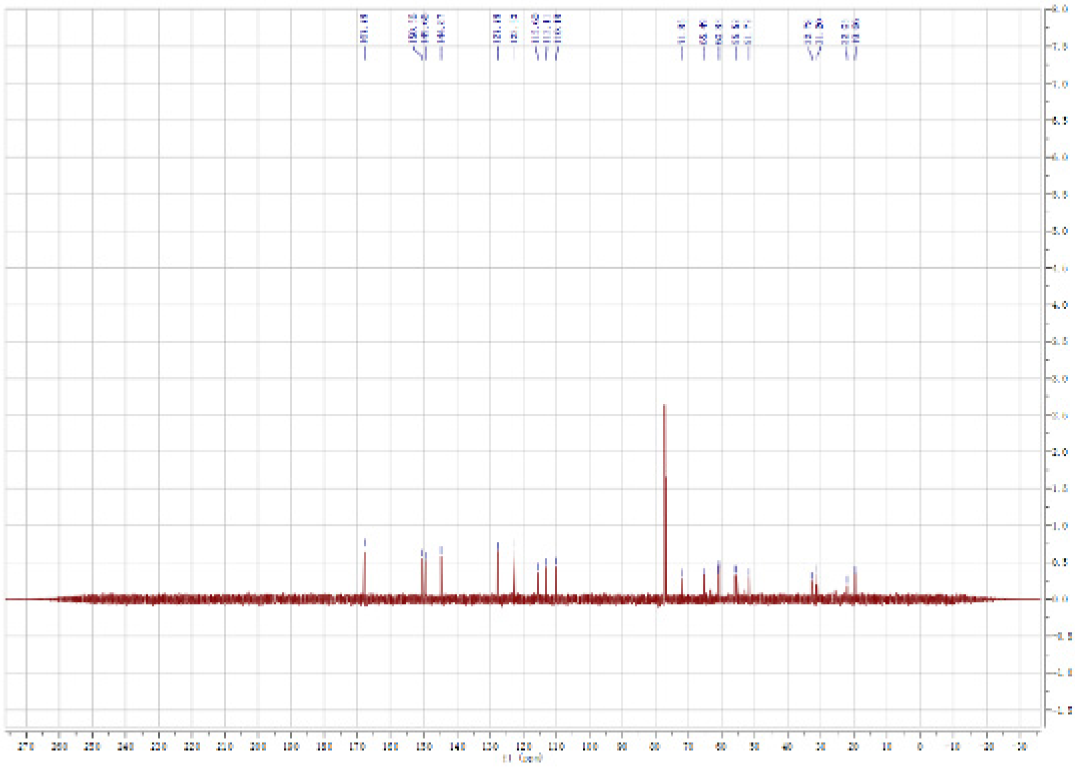

^{13}C NMR spectrum ($CDCl_3$, 126 MHz) of **A$_{15}$**.

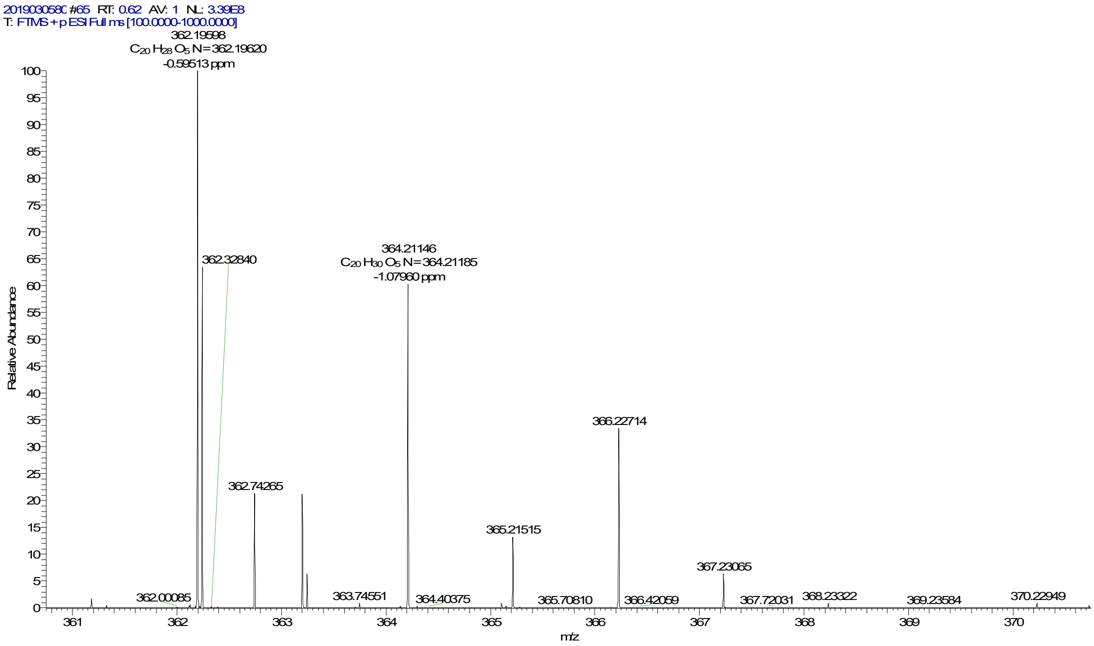

HRMS spectrum of target compound **A15**.

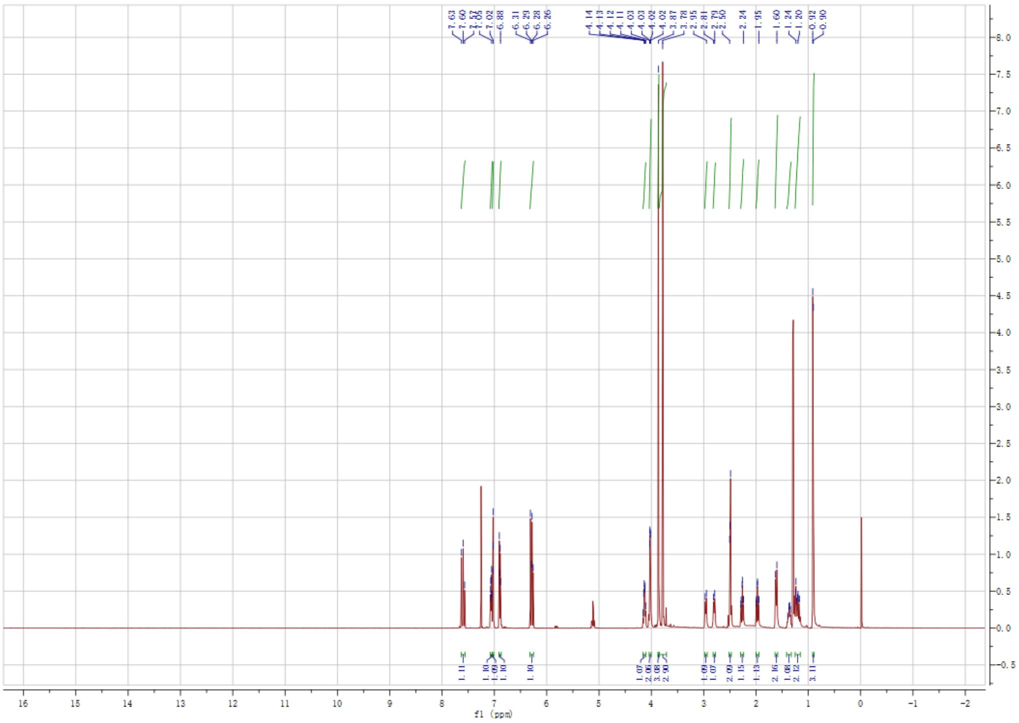

^{1}H NMR spectrum ($CDCl_3$, 500 MHz) of **A16**.

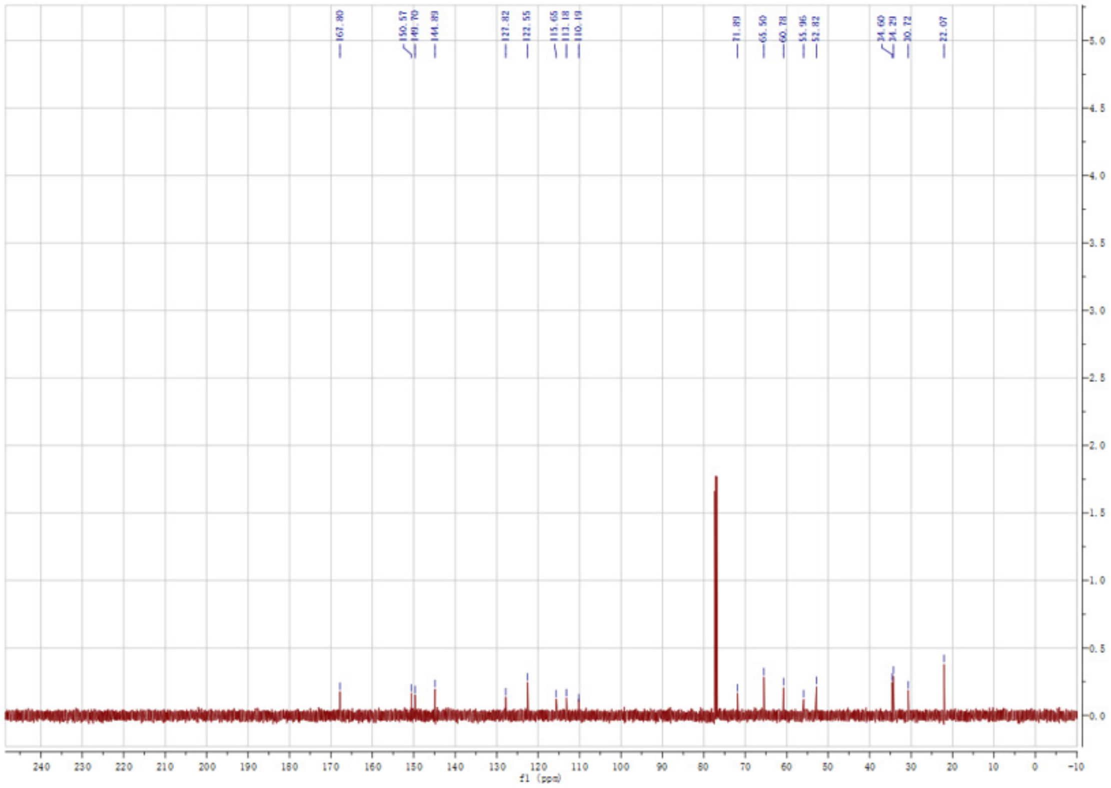

^{13}C NMR spectrum (CDCl$_3$, 126 MHz) of **A$_{16}$**.

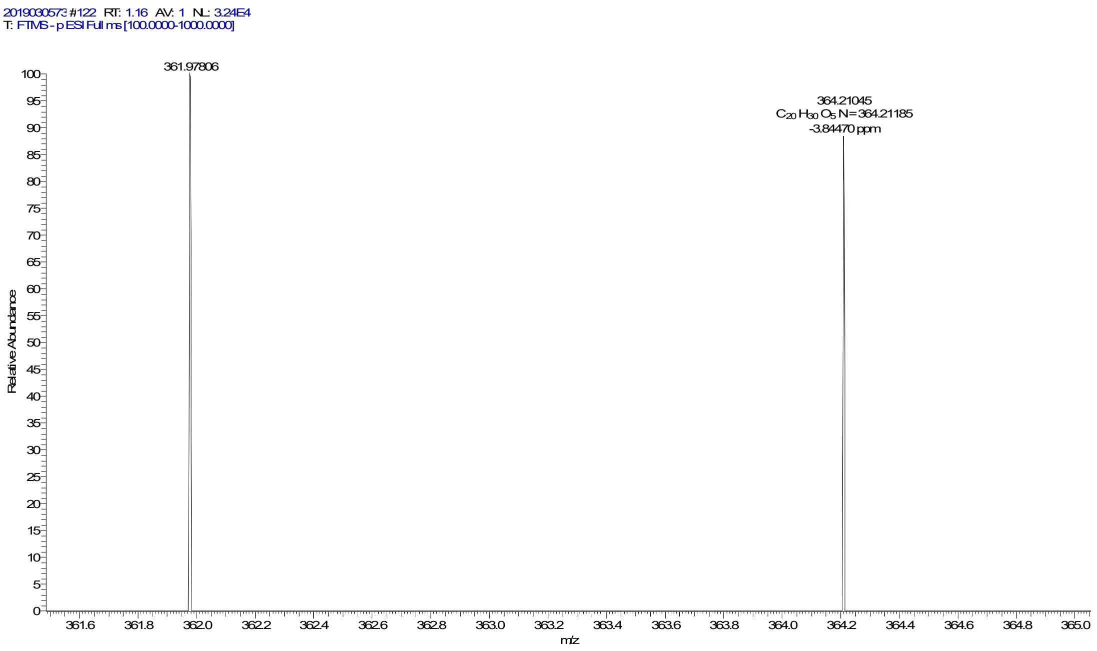

HRMS spectrum of target compound **A$_{16}$**.

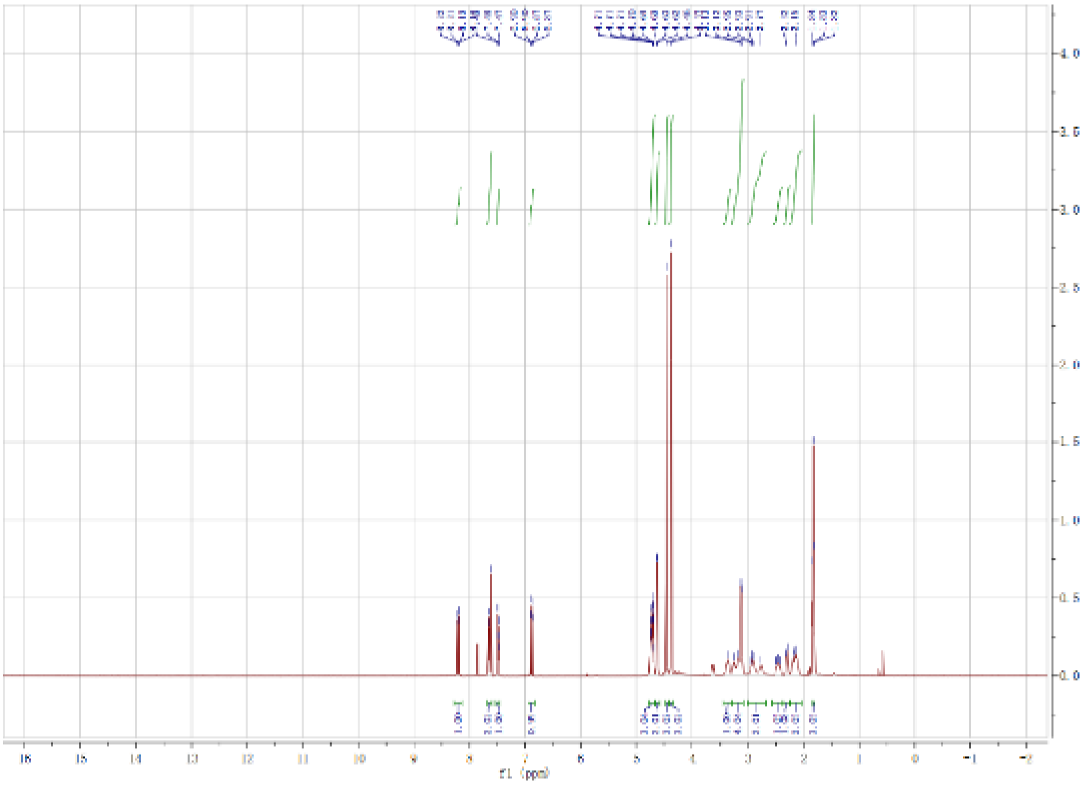

^{1}H NMR spectrum ($CDCl_3$, 500 MHz) of **A_{17}**.

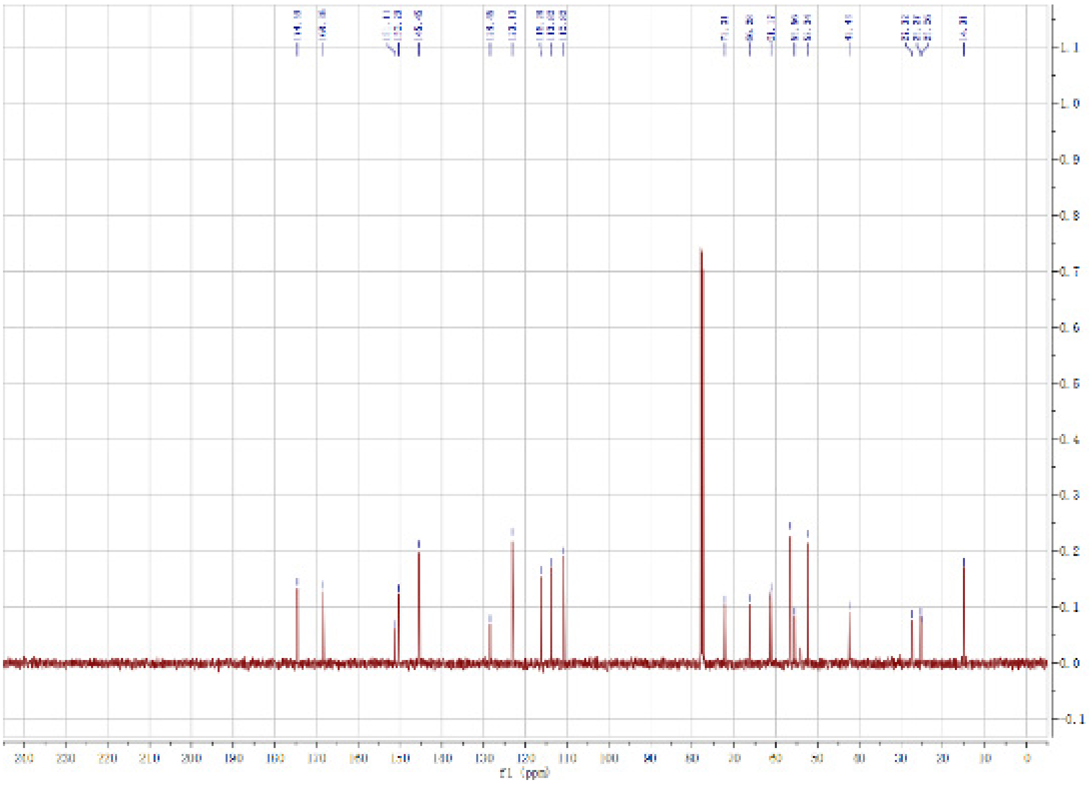

^{13}C NMR spectrum ($CDCl_3$, 126 MHz) of **A_{17}**.

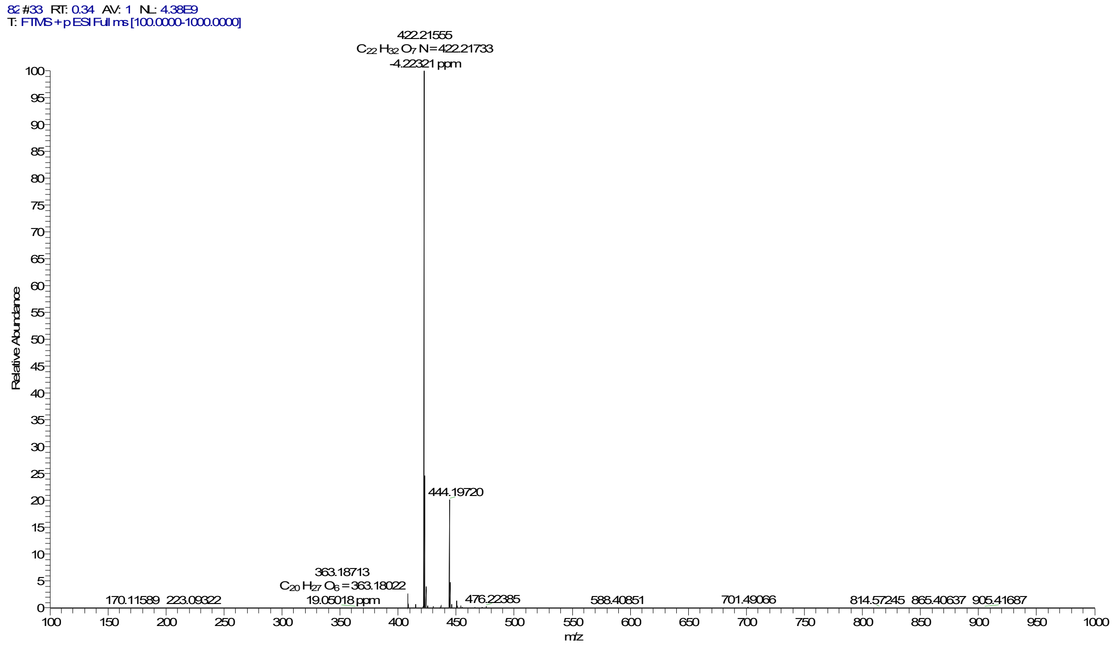

HRMS spectrum of target compound **A**$_{\mathbf{17}}$.

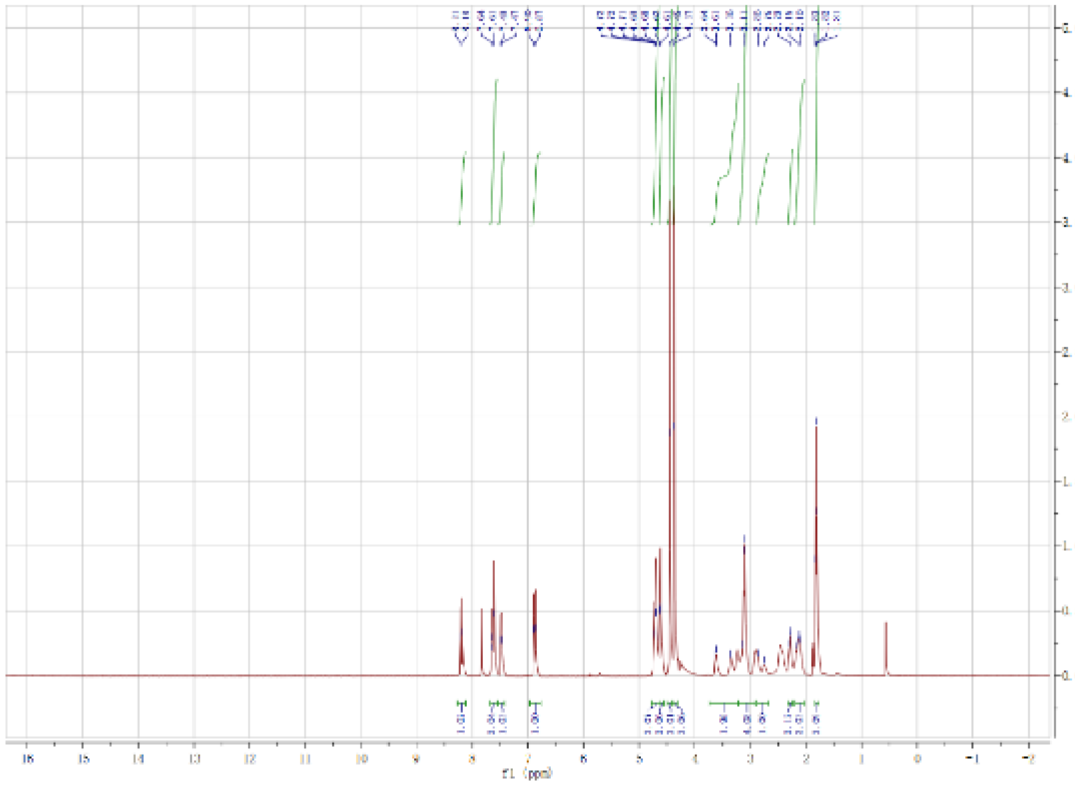

^{1}H NMR spectrum ($CDCl_3$, 500 MHz) of **A**$_{\mathbf{18}}$.

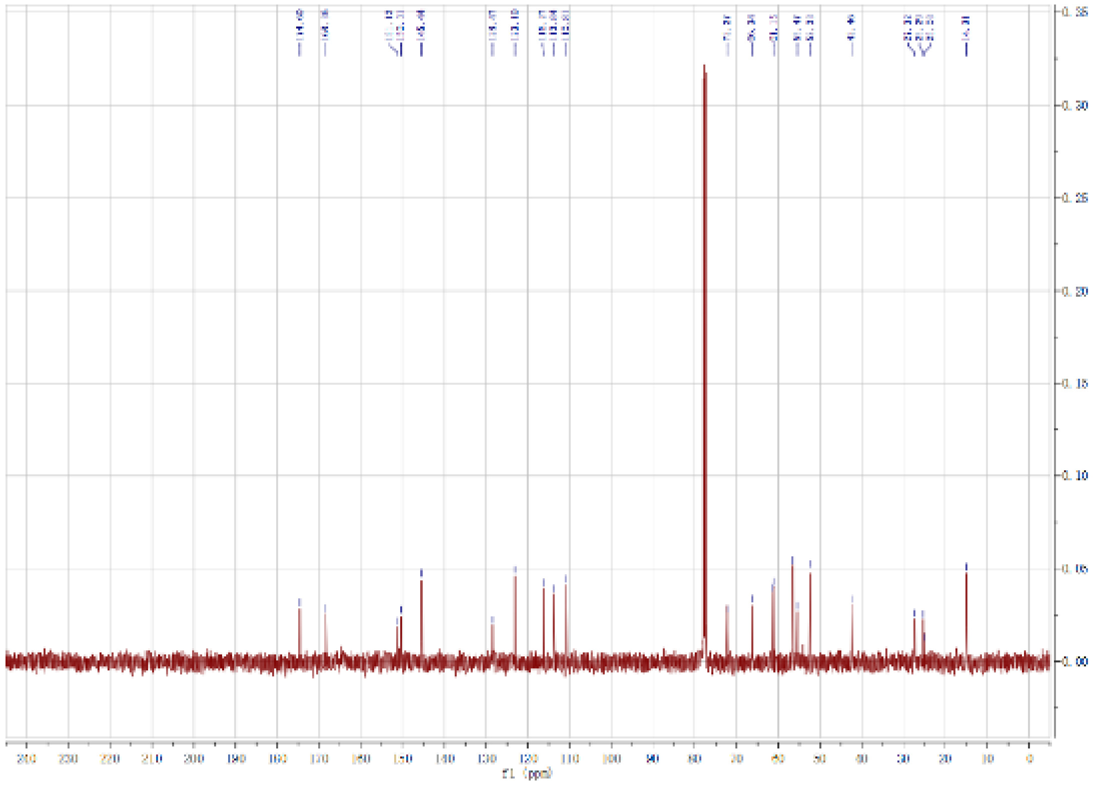

^{13}C NMR spectrum ($CDCl_3$, 126 MHz) of **A18**.

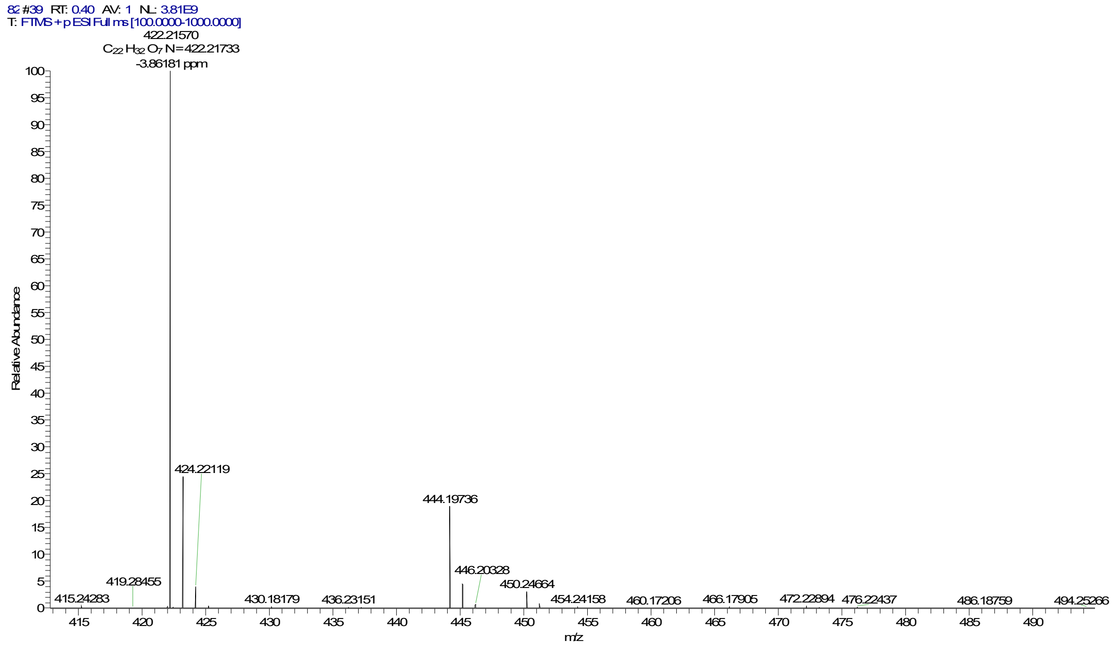

HRMS spectrum of target compound **A18**.

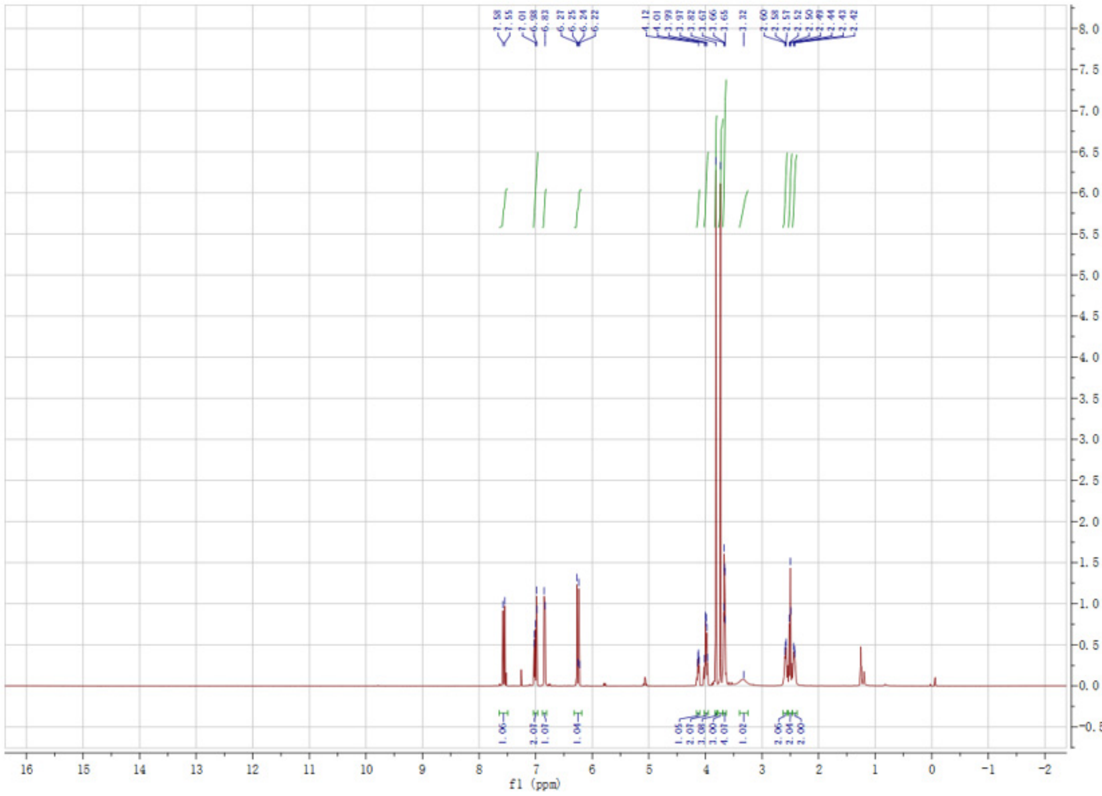

^{1}H NMR spectrum ($CDCl_3$, 500 MHz) of $\mathbf{A_{19}}$.

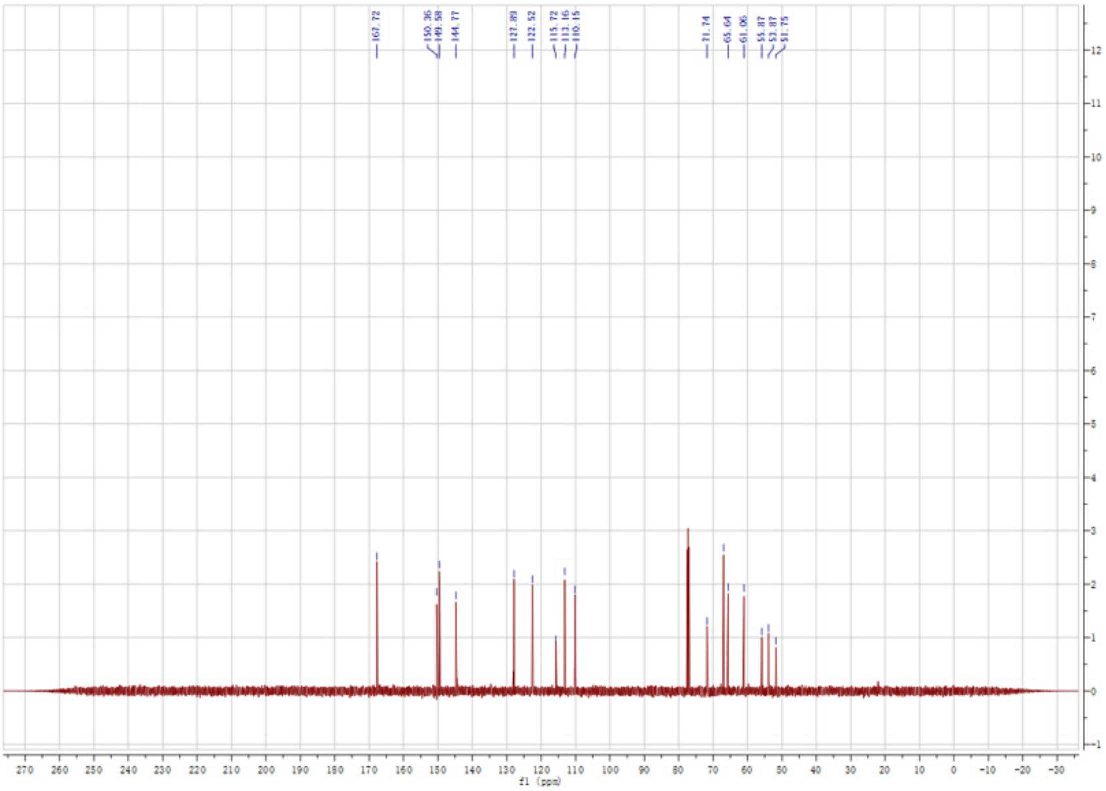

^{13}C NMR spectrum ($CDCl_3$, 126 MHz) of $\mathbf{A_{19}}$.

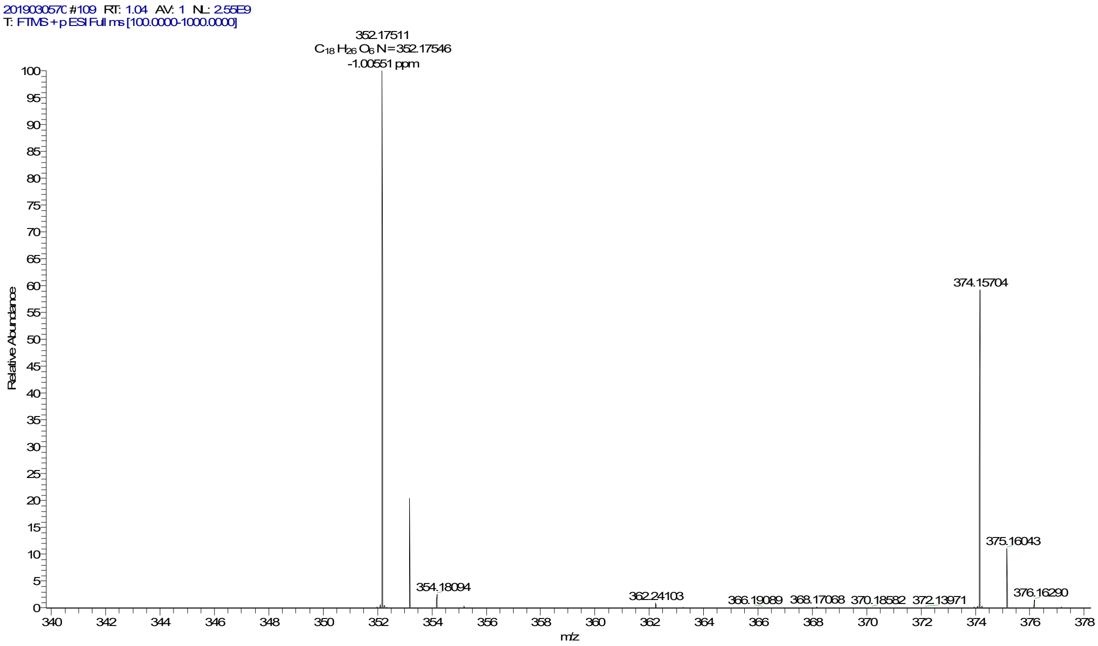

HRMS spectrum of target compound **A_{19}**.

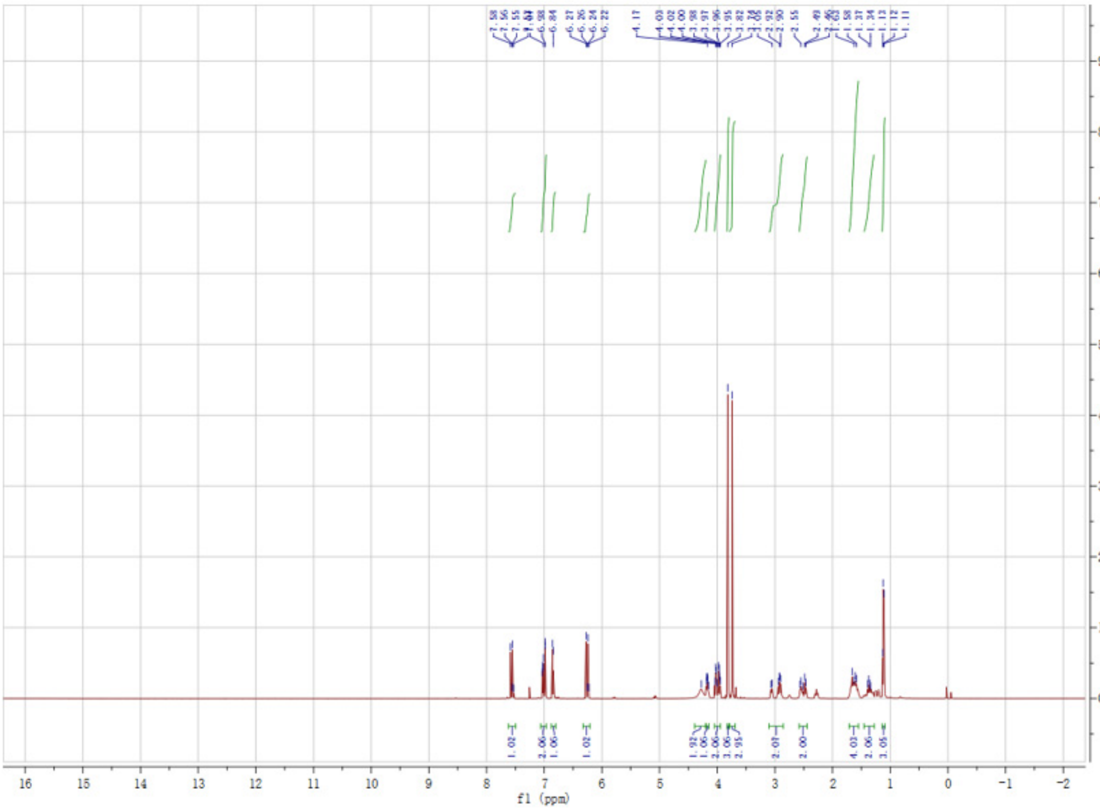

^{1}H NMR spectrum ($CDCl_3$, 500 MHz) of **A_{20}**.

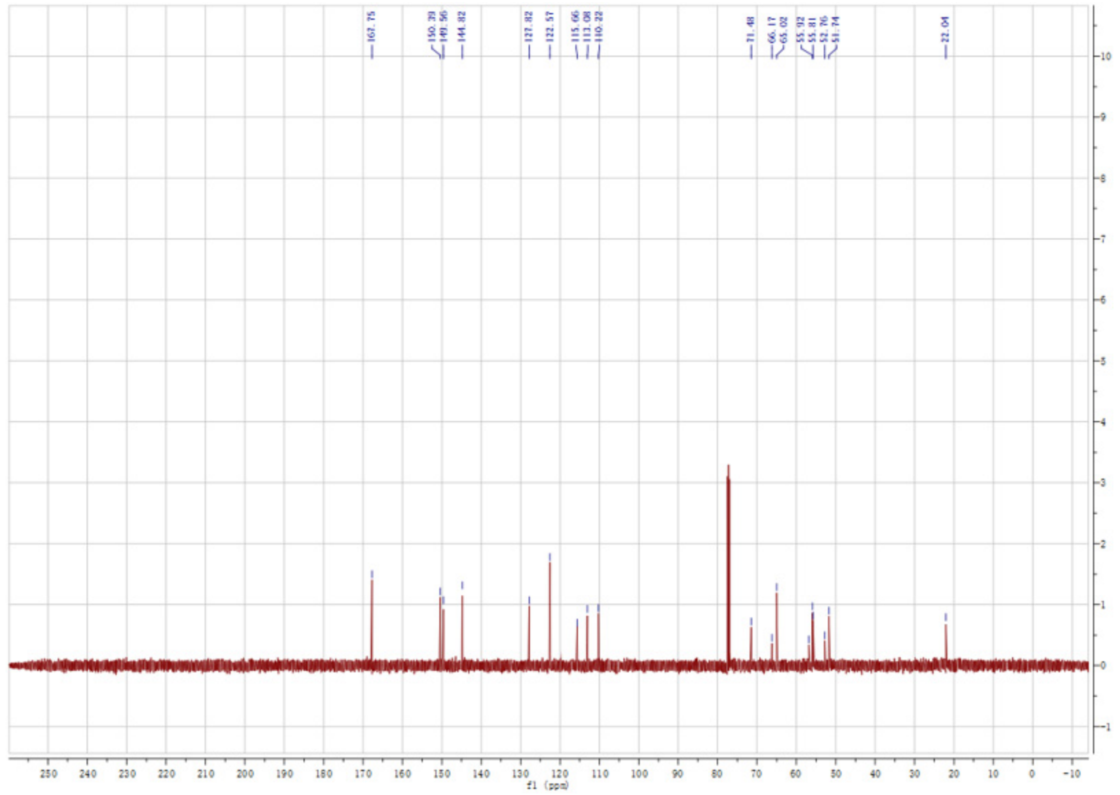

^{13}C NMR spectrum ($CDCl_3$, 126 MHz) of **A_{20}**.

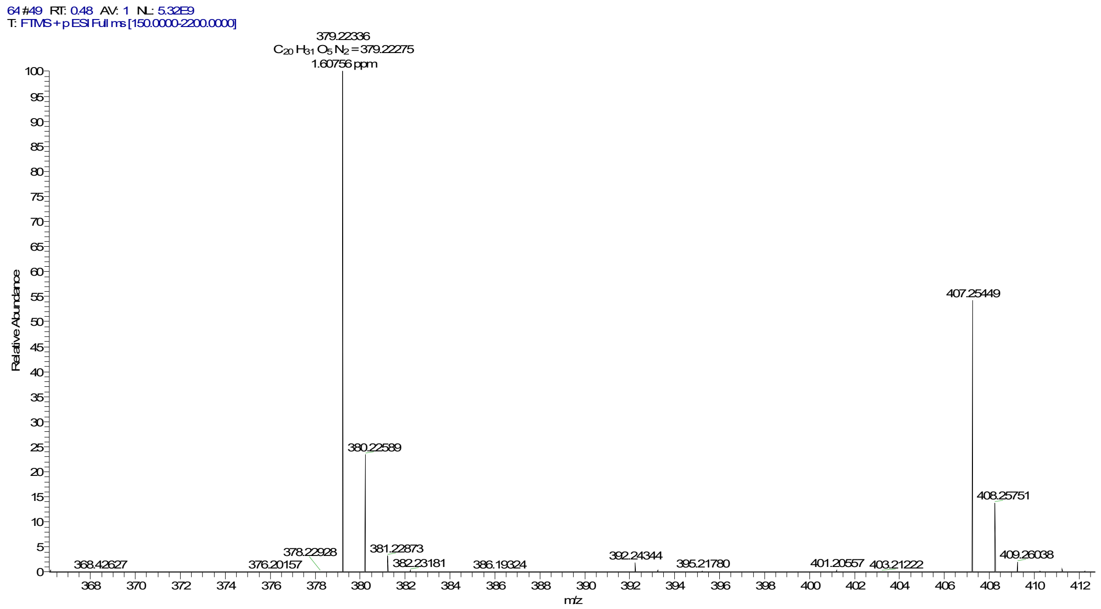

HRMS spectrum of target compound **A_{20}**.

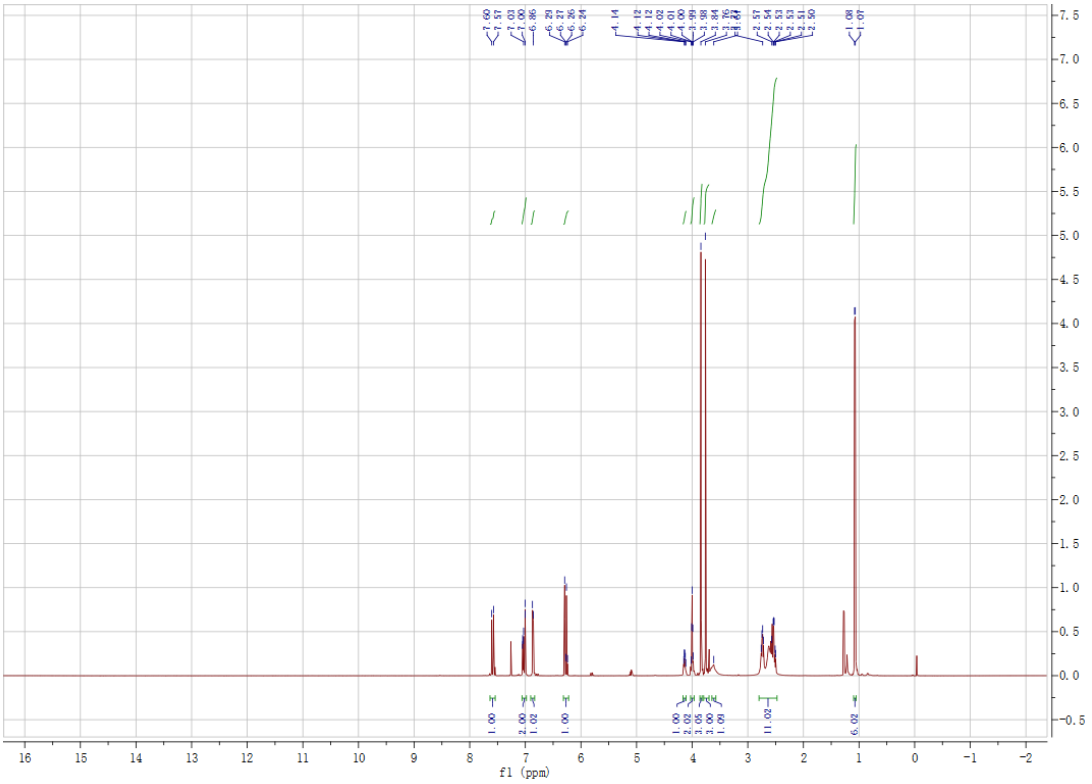

^{1}H NMR spectrum ($CDCl_3$, 500 MHz) of **A_{21}**.

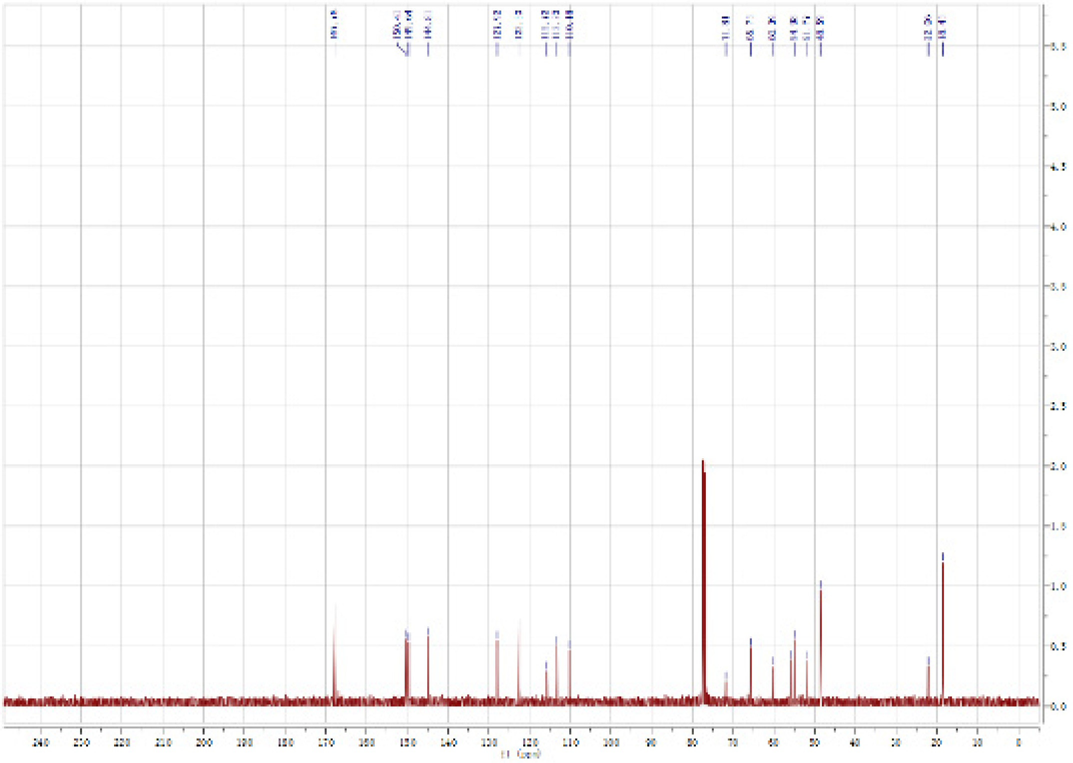

^{13}C NMR spectrum ($CDCl_3$, 126 MHz) of **A_{21}**.

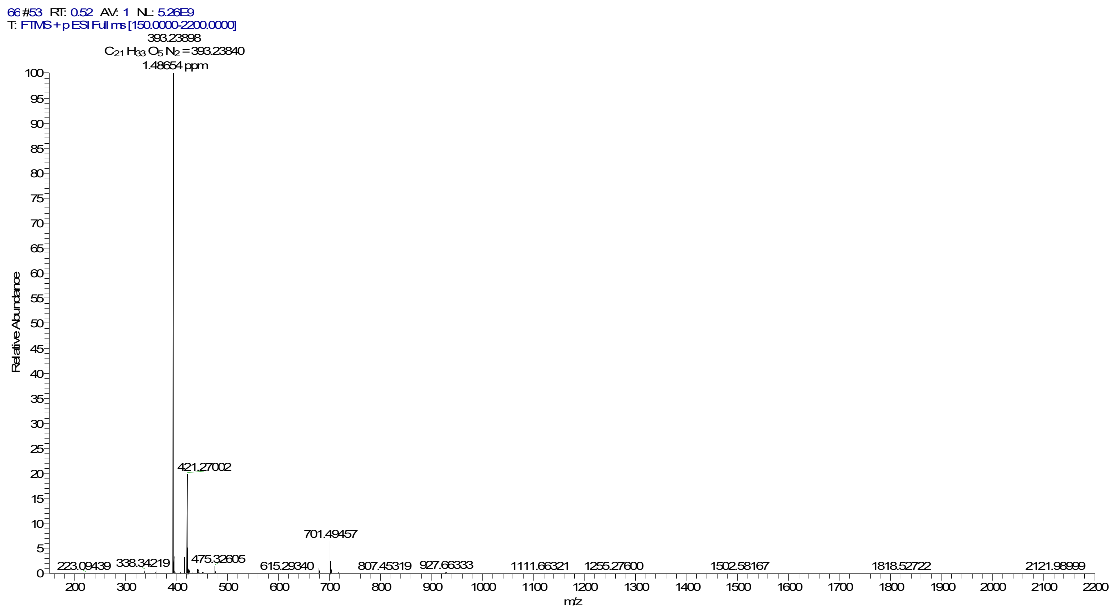

HRMS spectrum of target compound **A21**.

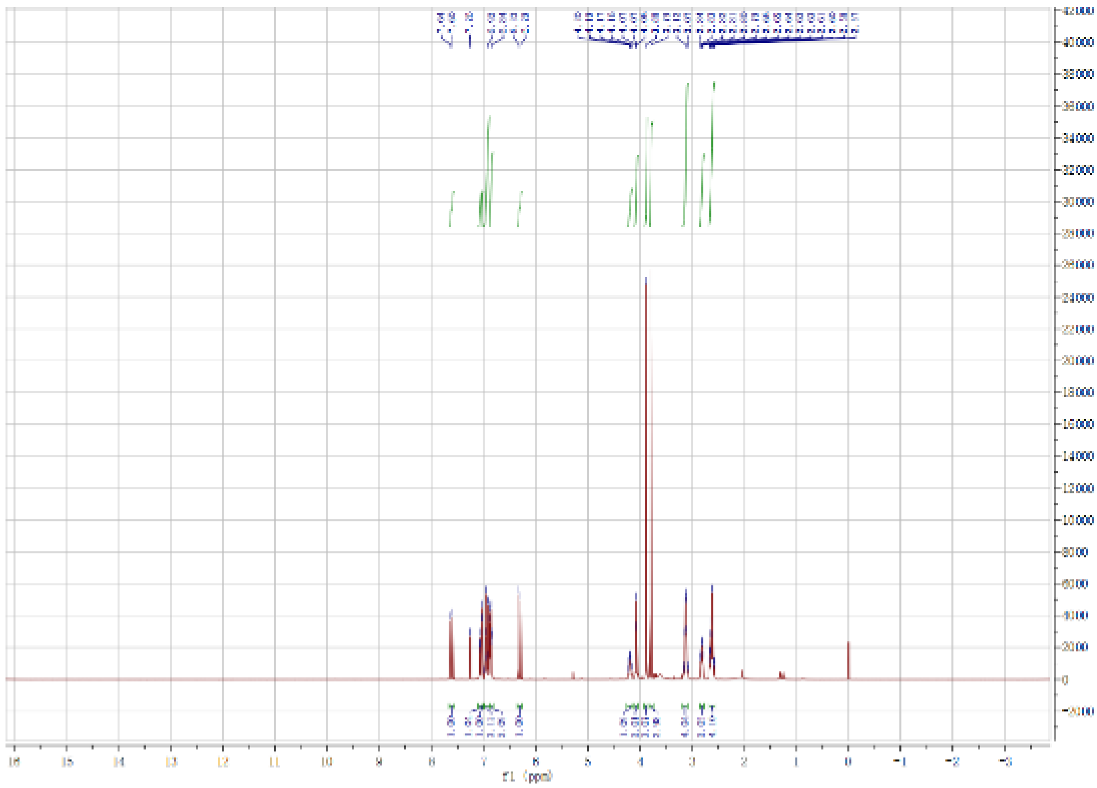

^{1}H NMR spectrum ($CDCl_3$, 400 MHz) of **A22**.

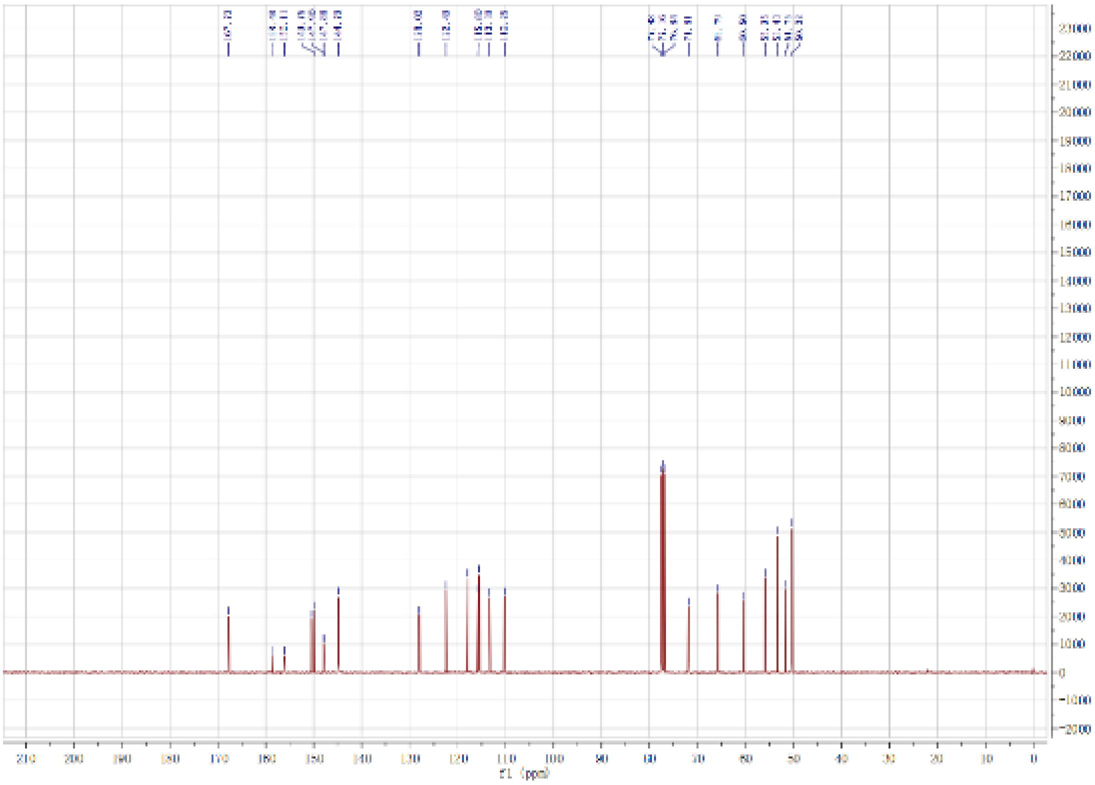

^{13}C NMR spectrum ($CDCl_3$, 101 MHz) of **A_{22}**.

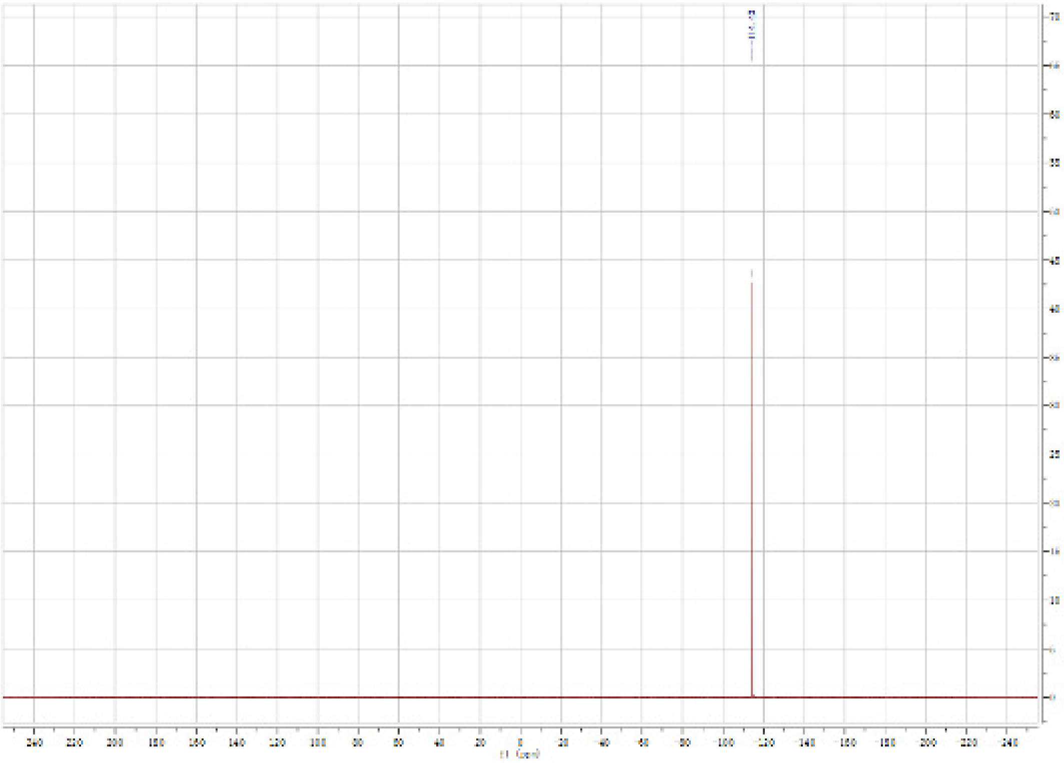

^{19}F NMR spectrum ($CDCl_3$, 471 MHz) of **A_{22}**.

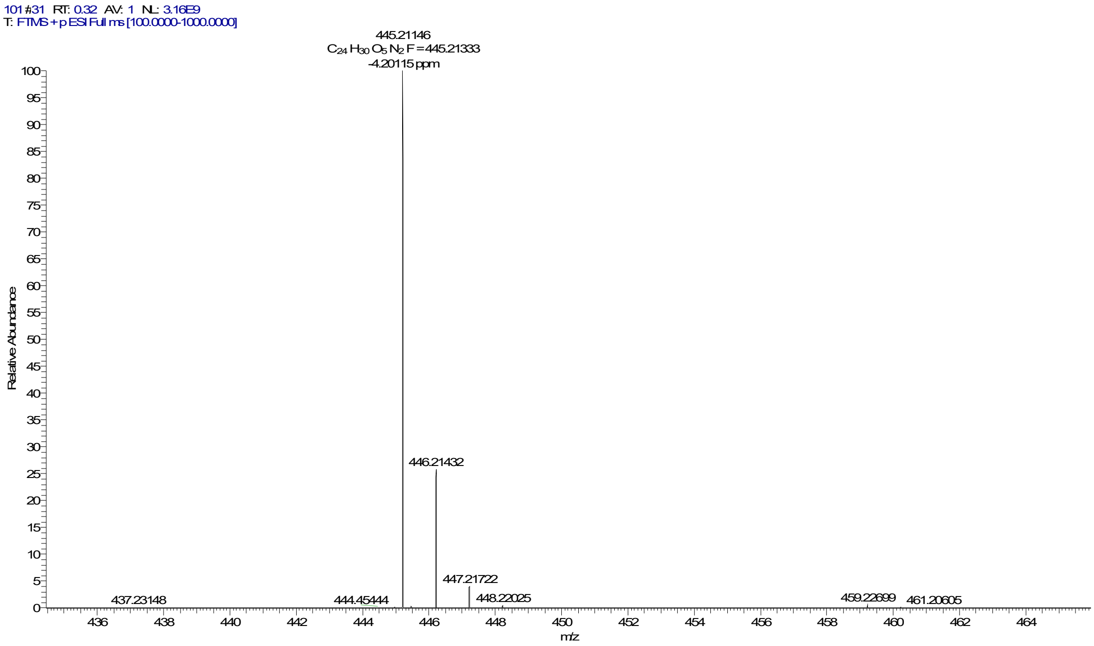

HRMS spectrum of target compound **A$_{22}$**.

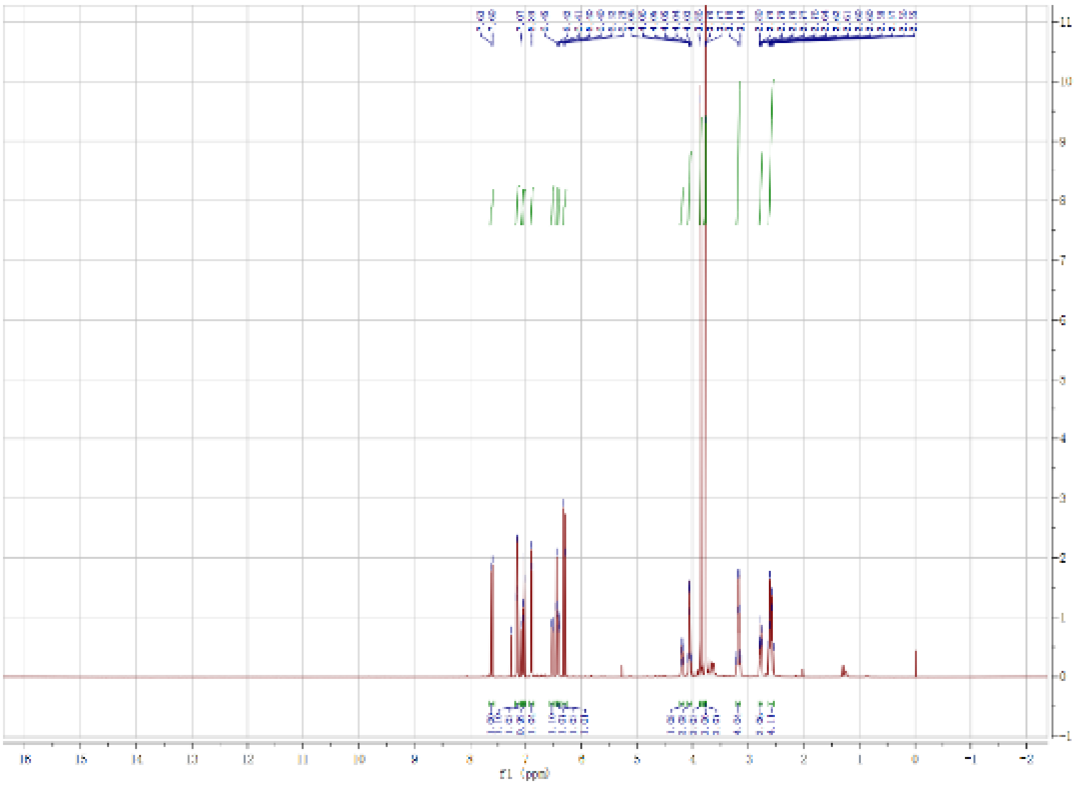

^{1}H NMR spectrum ($CDCl_3$, 500 MHz) of **A$_{23}$**.

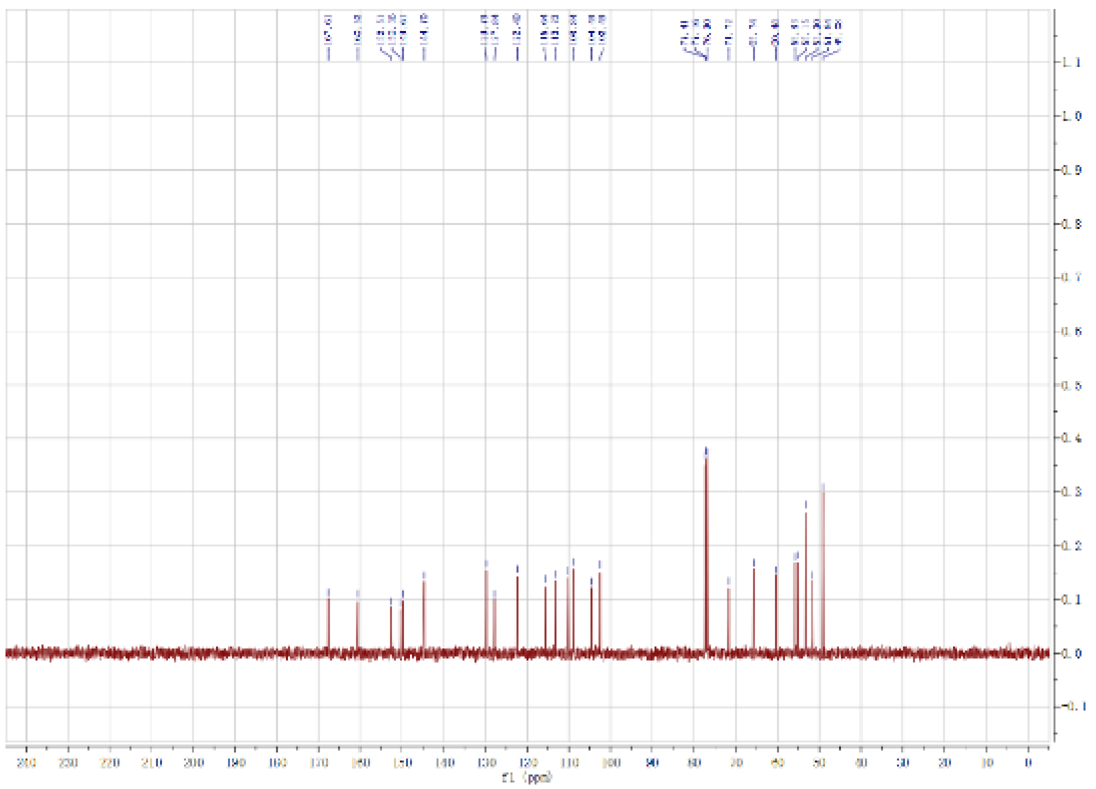

^{13}C NMR spectrum ($CDCl_3$, 126 MHz) of **A$_{23}$**.

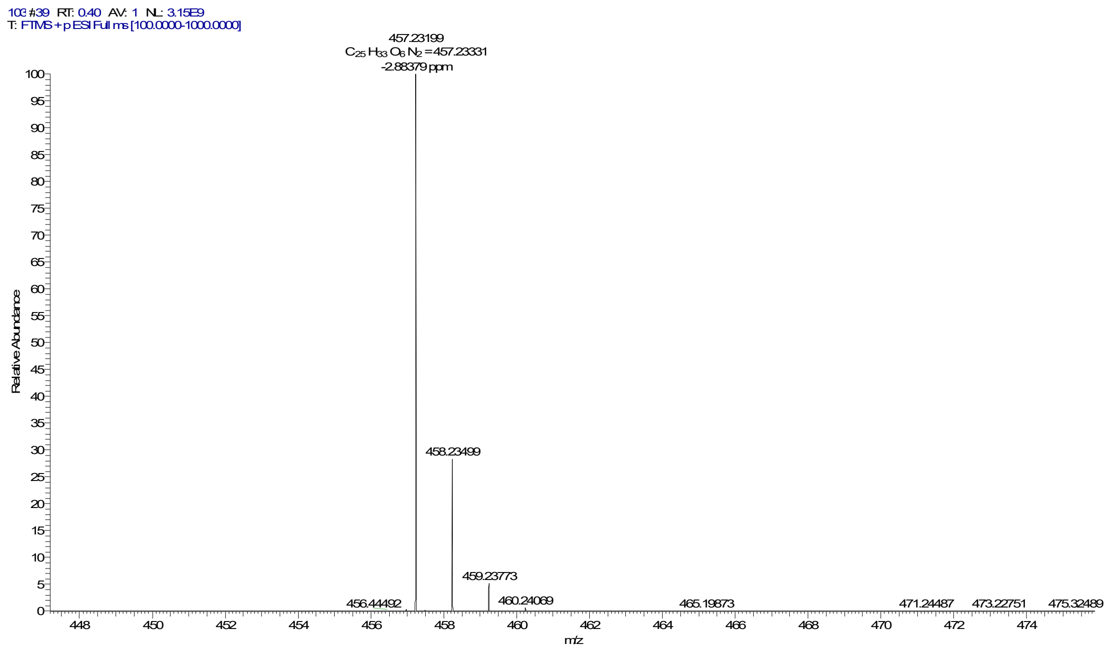

HRMS spectrum of target compound **A$_{23}$**.

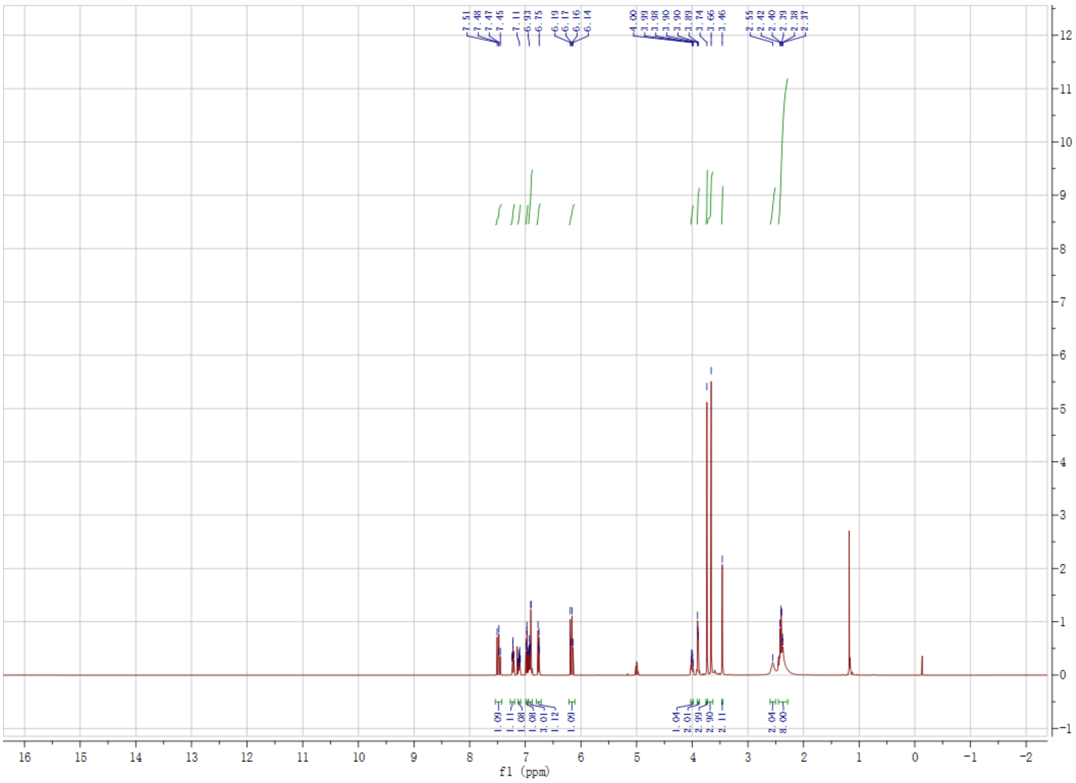

^{1}H NMR spectrum ($CDCl_3$, 500 MHz) of $\mathbf{A_{24}}$.

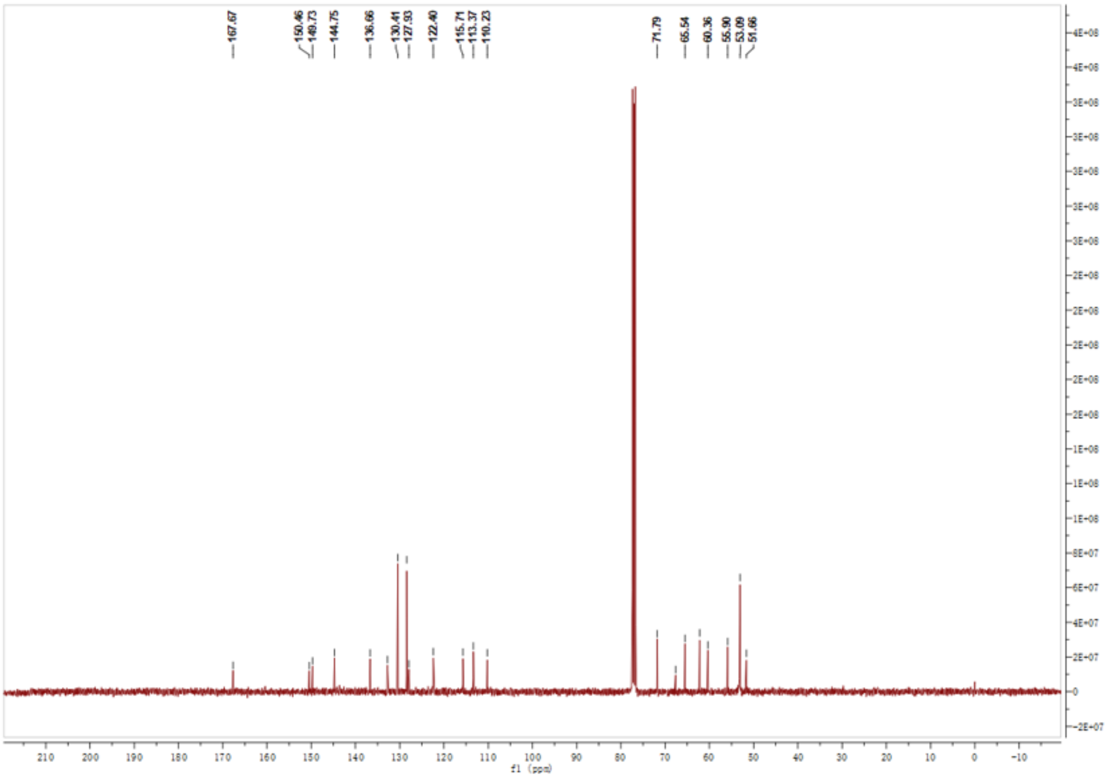

^{13}C NMR spectrum ($CDCl_3$, 126 MHz) of $\mathbf{A_{24}}$.

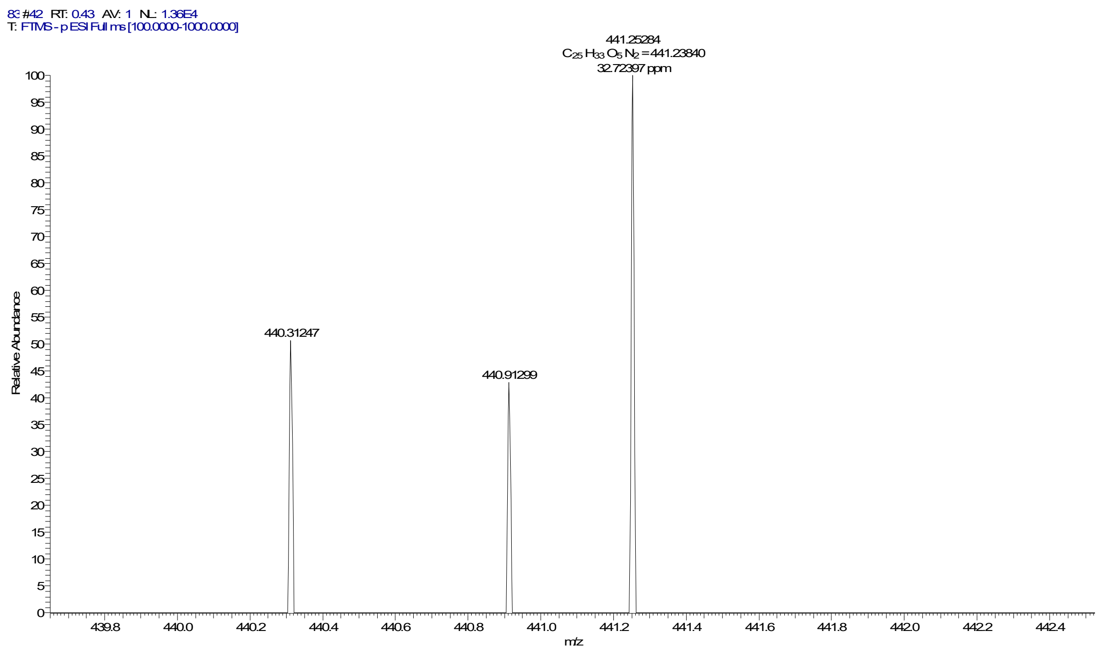

HRMS spectrum of target compound **A₂₄**.

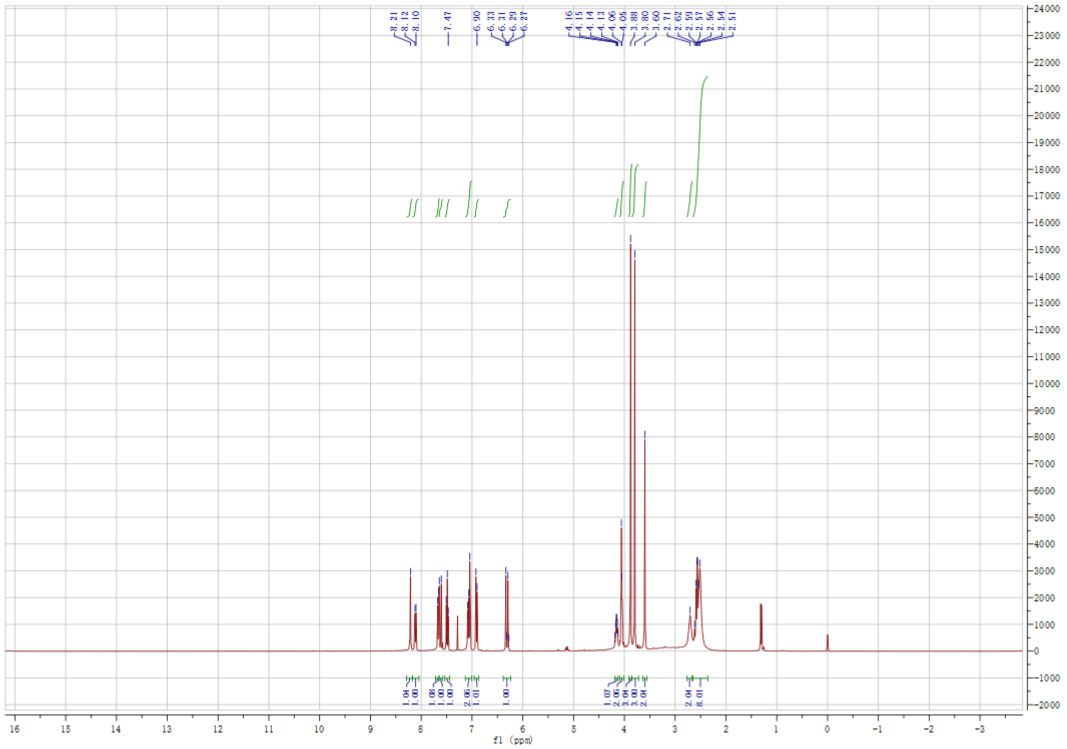

^{1}H NMR spectrum ($CDCl_3$, 400 MHz) of **A₂₅**.

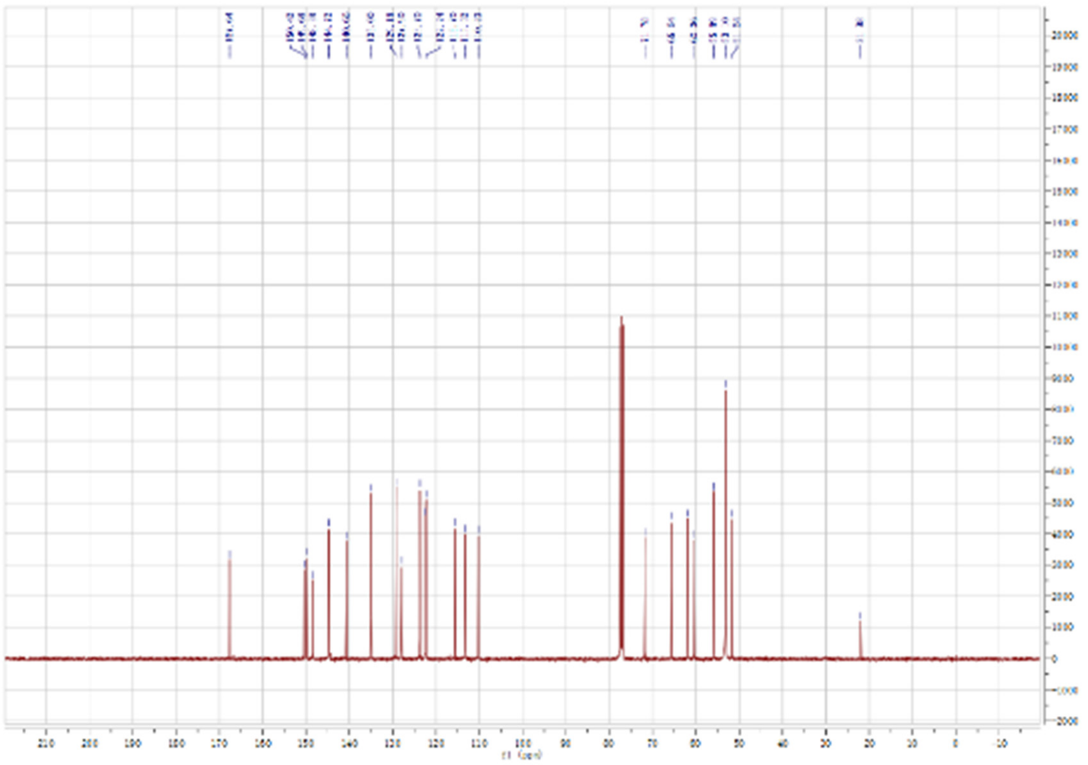

^{13}C NMR spectrum ($CDCl_3$, 101 MHz) of **A_{25}**.

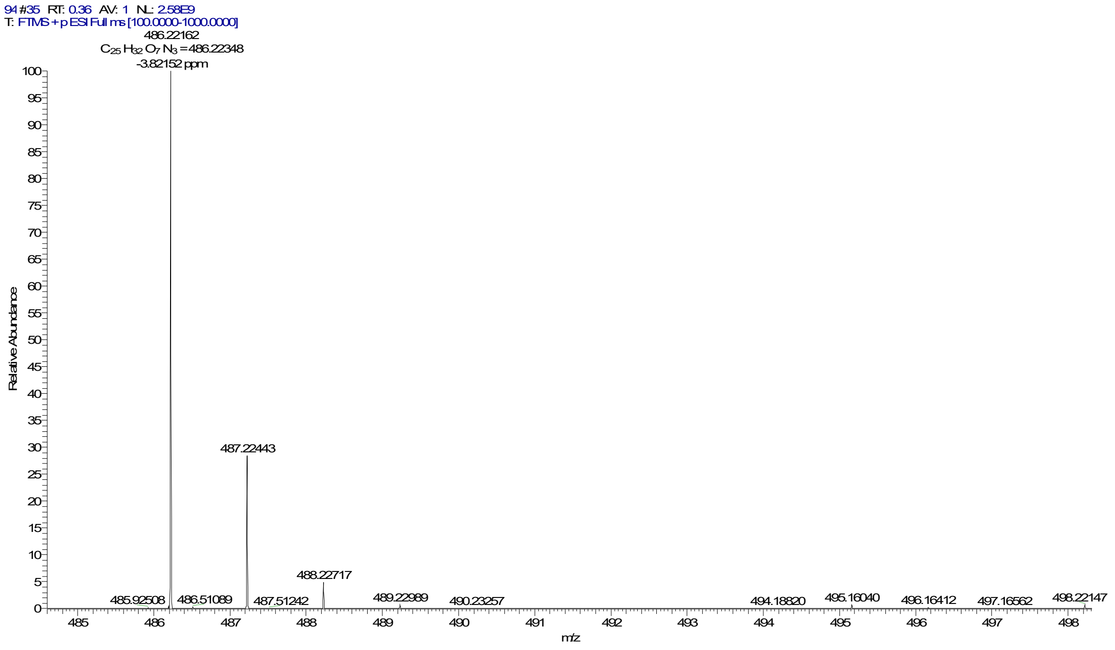

HRMS spectrum of target compound **A_{25}**.

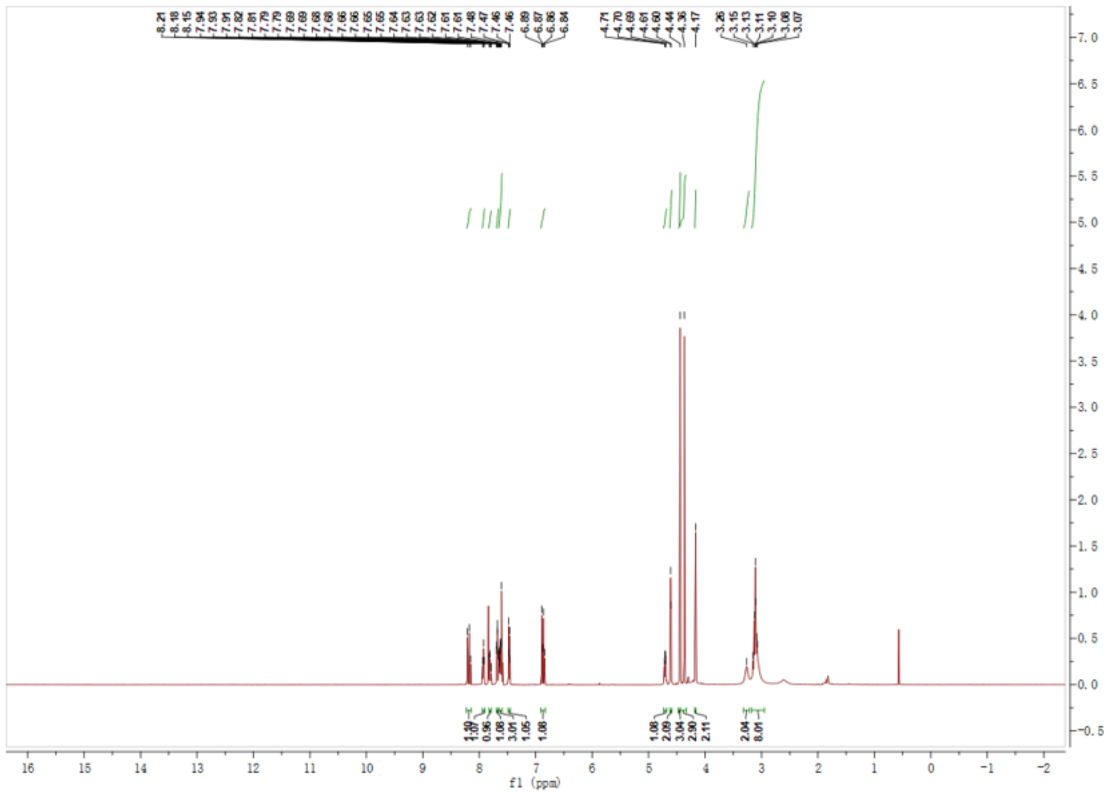

^{1}H NMR spectrum ($CDCl_3$, 500 MHz) of **A_{26}**.

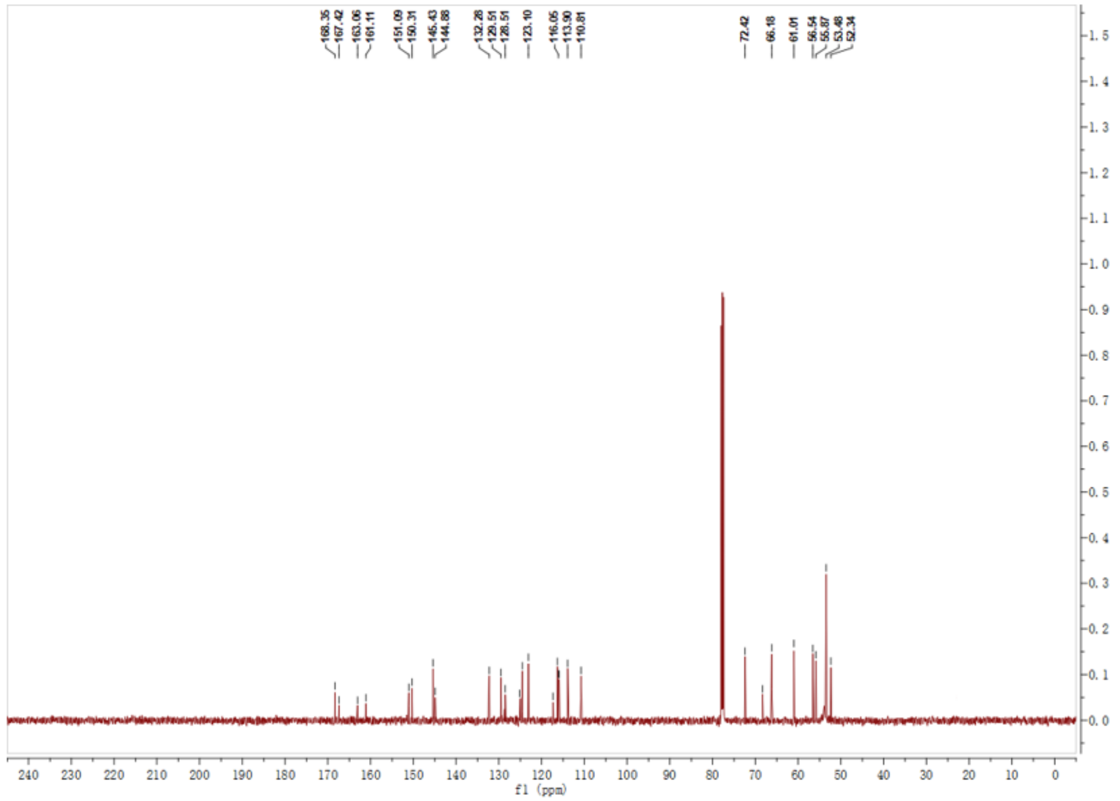

^{13}C NMR spectrum ($CDCl_3$, 126 MHz) of **A_{26}**.

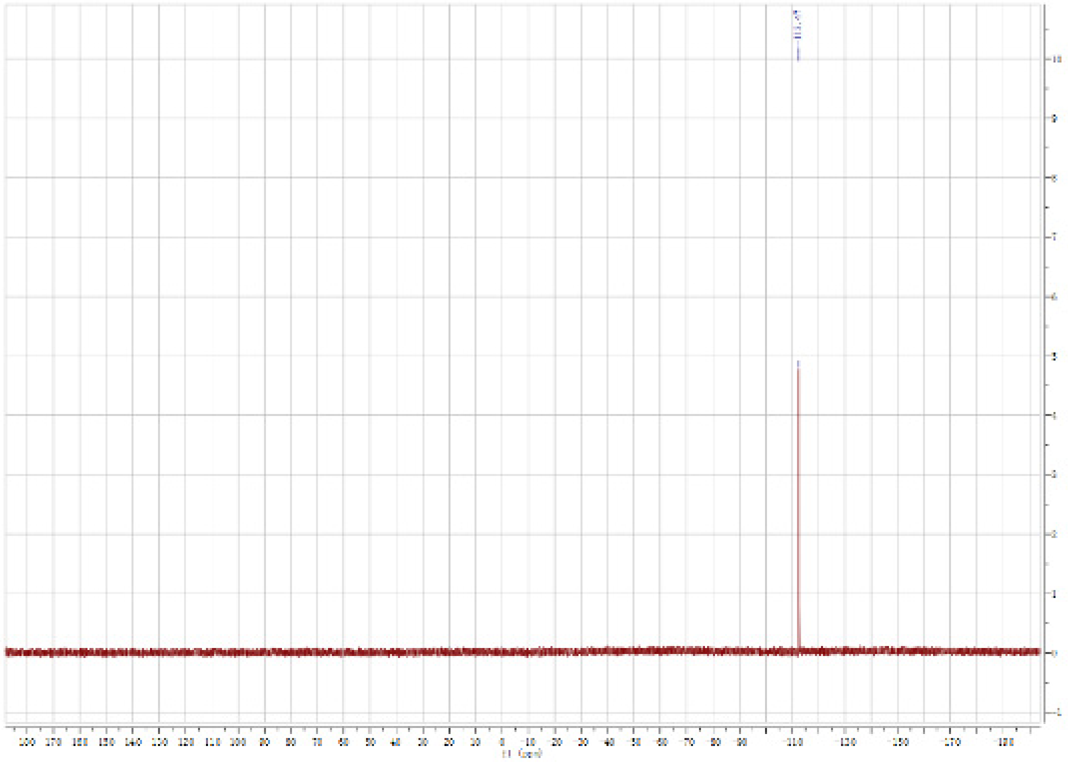

^{19}F NMR spectrum ($CDCl_3$, 471 MHz) of **A_{26}**.

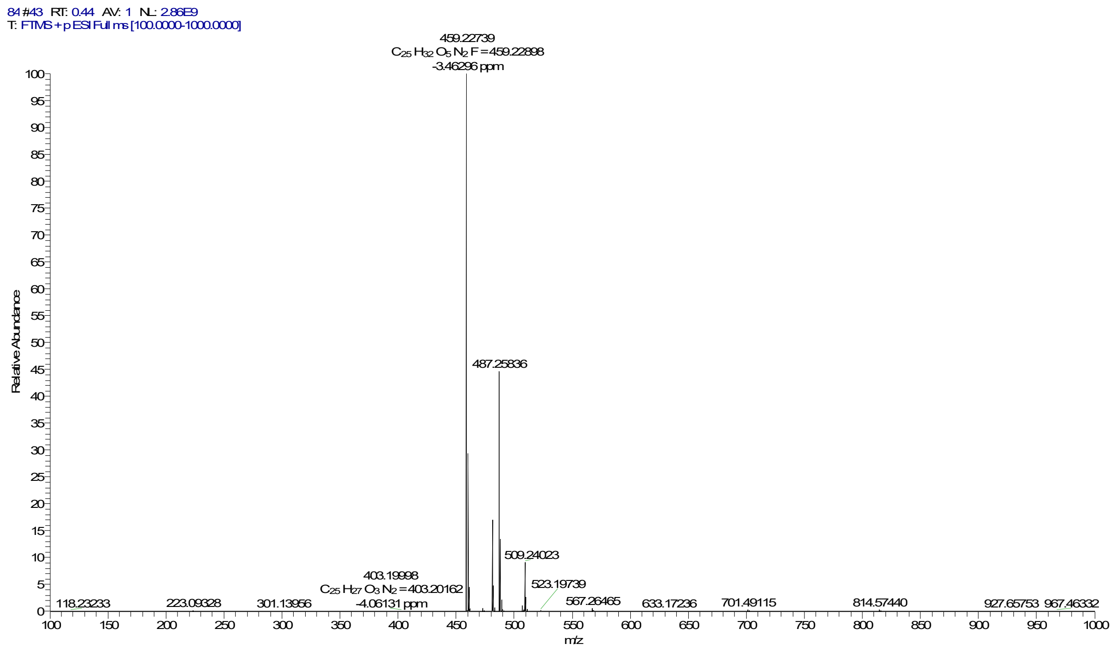

HRMS spectrum of target compound **A_{26}**.

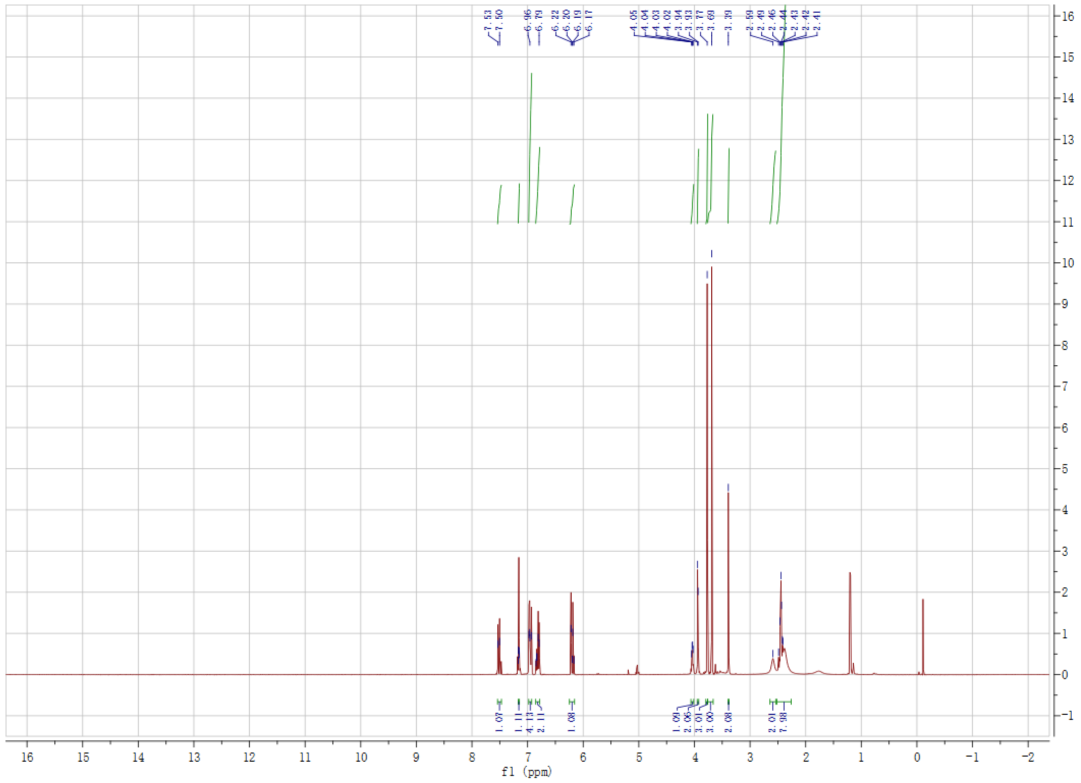

^{1}H NMR spectrum ($CDCl_3$, 500 MHz) of **A$_{27}$**.

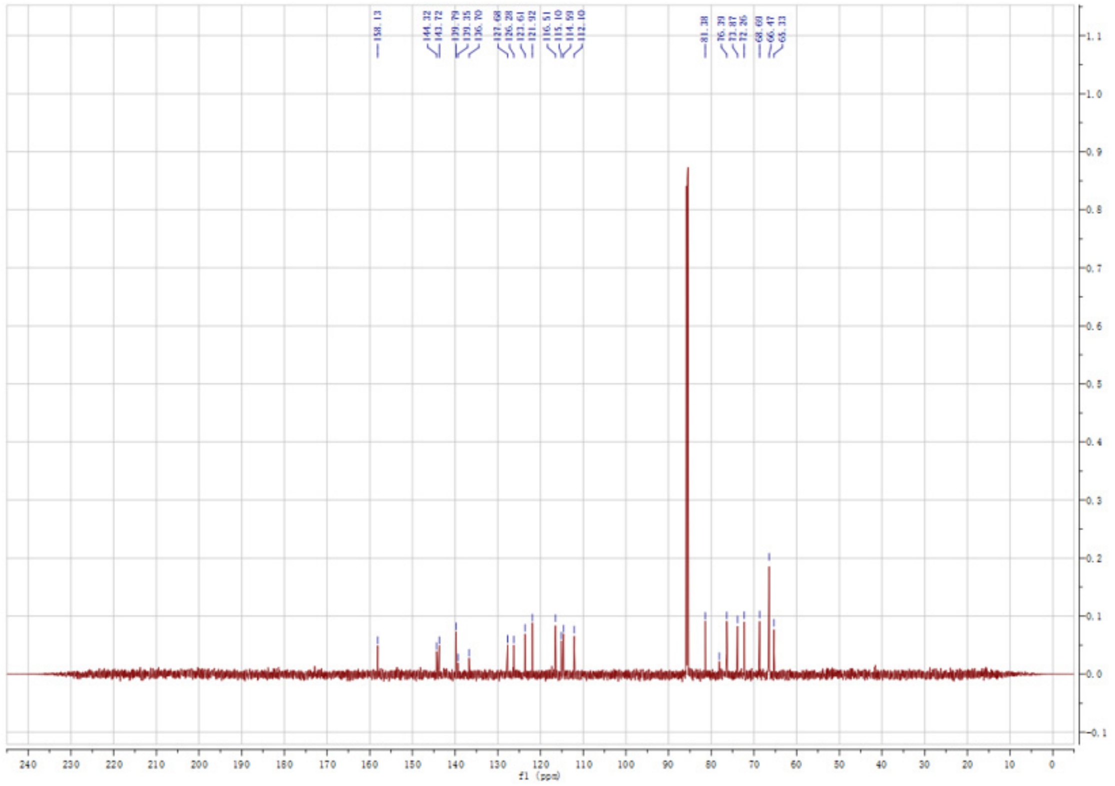

^{13}C NMR spectrum ($CDCl_3$, 126 MHz) of **A$_{27}$**.

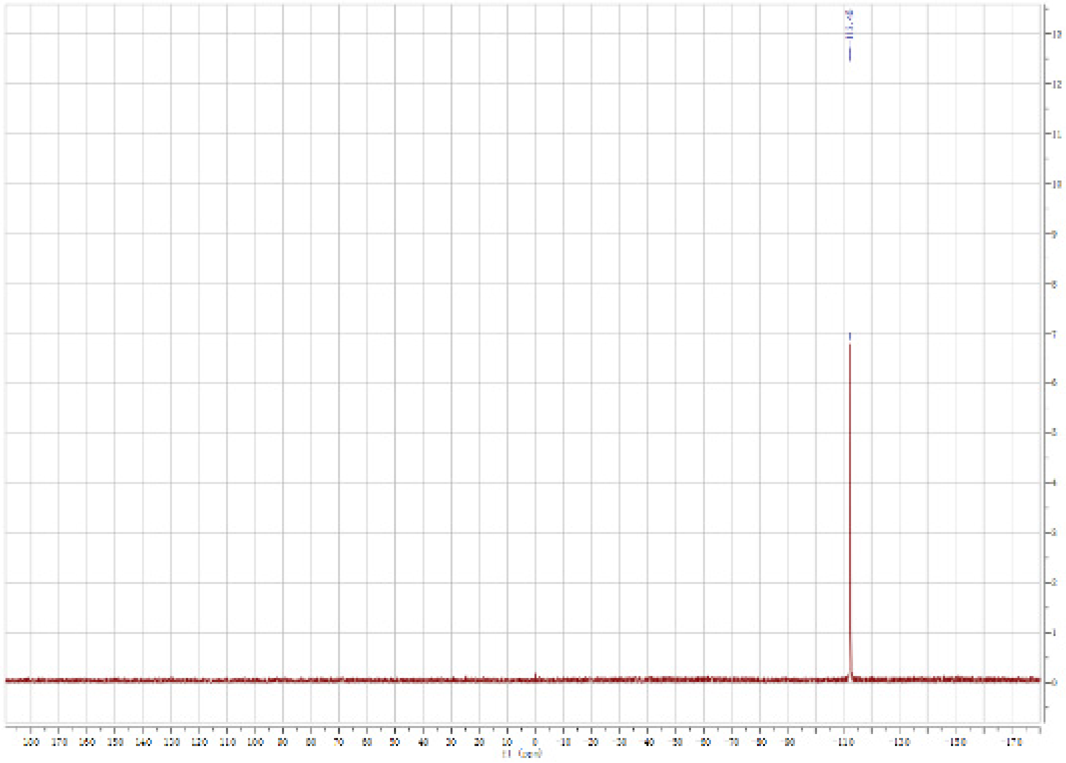

^{19}F NMR spectrum ($CDCl_3$, 471 MHz) of **A$_{27}$**.

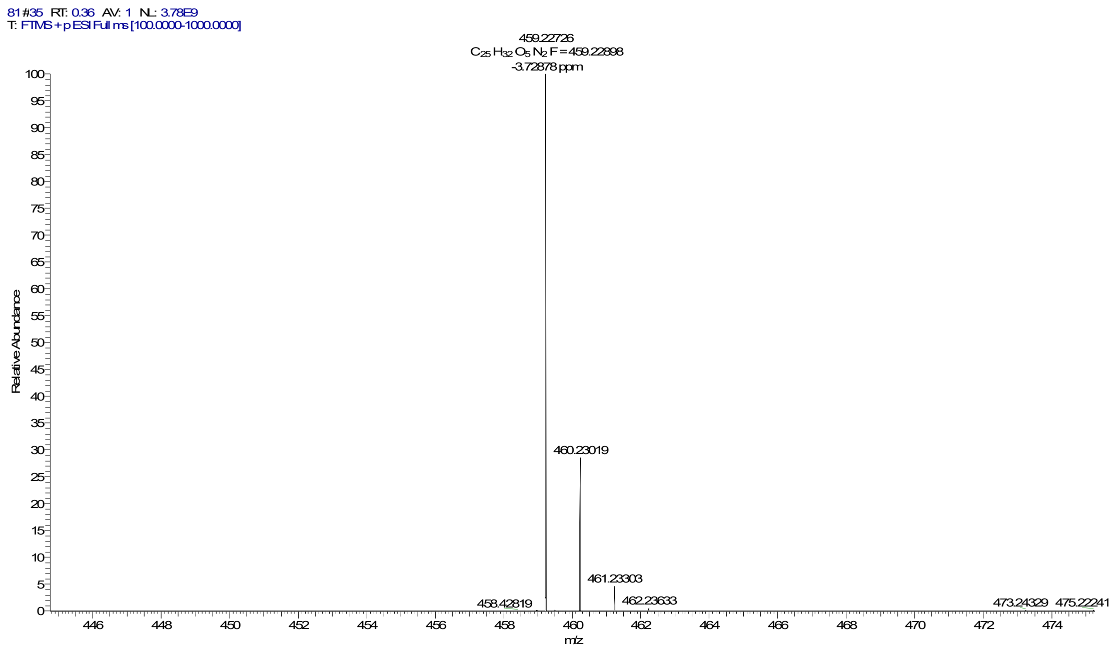

HRMS spectrum of target compound **A$_{27}$**.

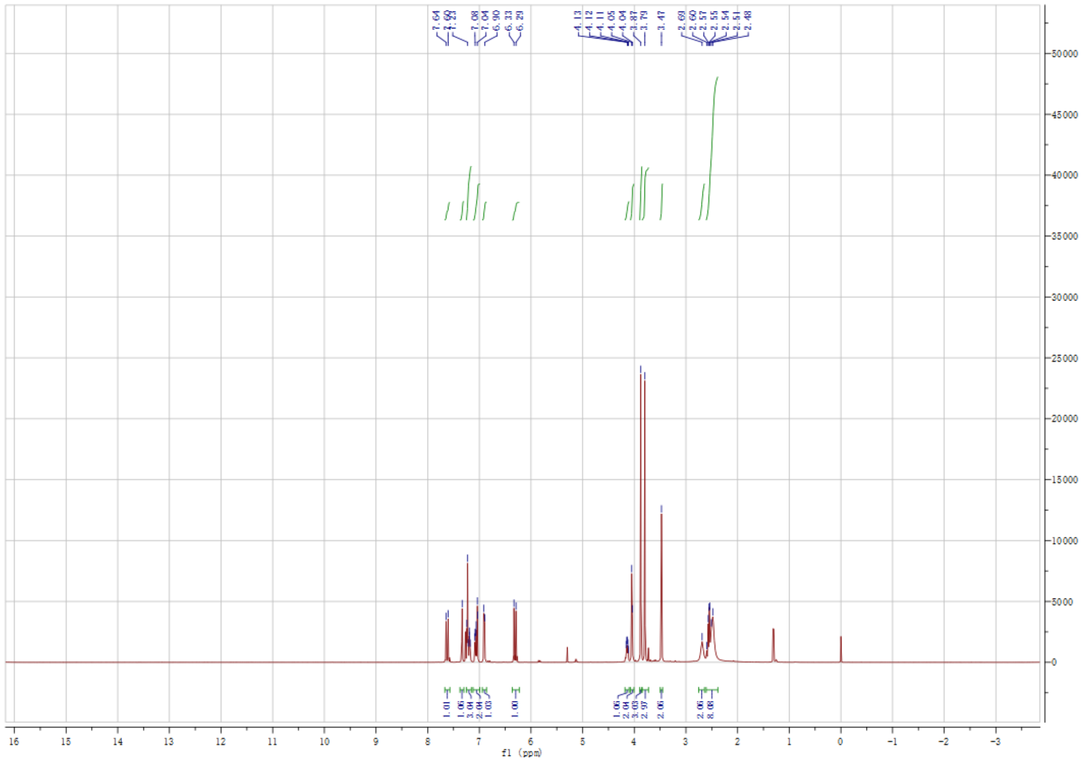

^{1}H NMR spectrum (CDCl$_3$, 400 MHz) of **A$_{28}$**.

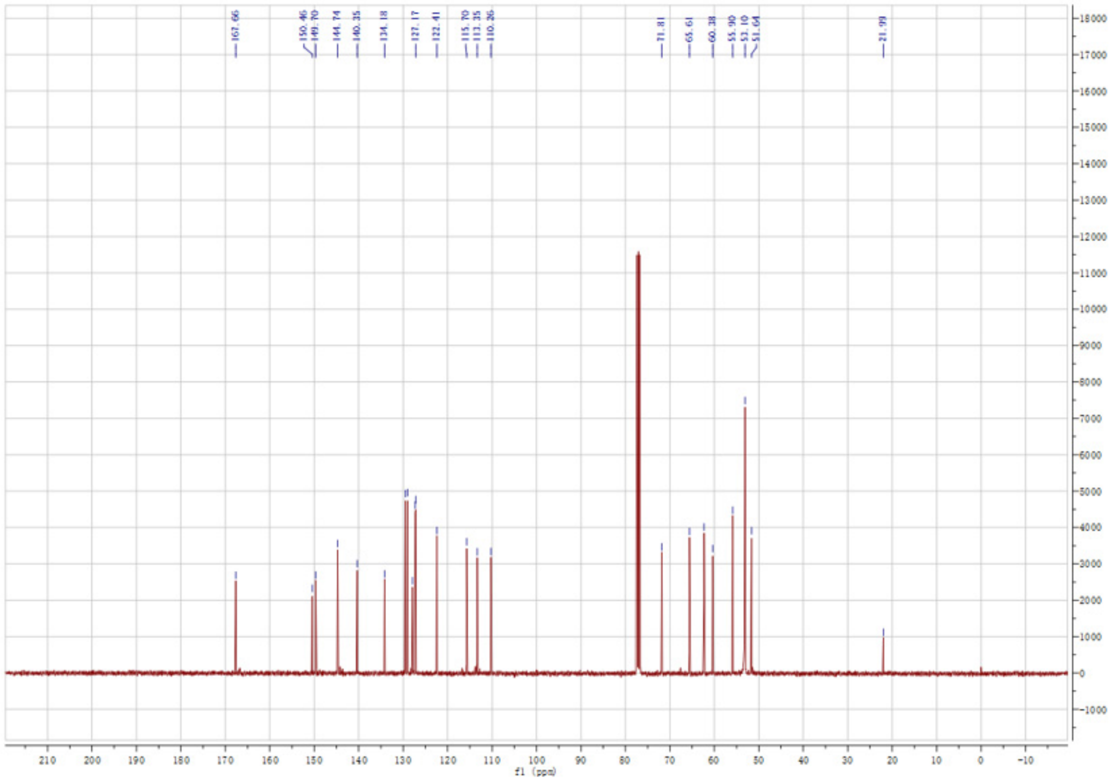

^{13}C NMR spectrum (CDCl$_3$, 101 MHz) of **A$_{28}$**.

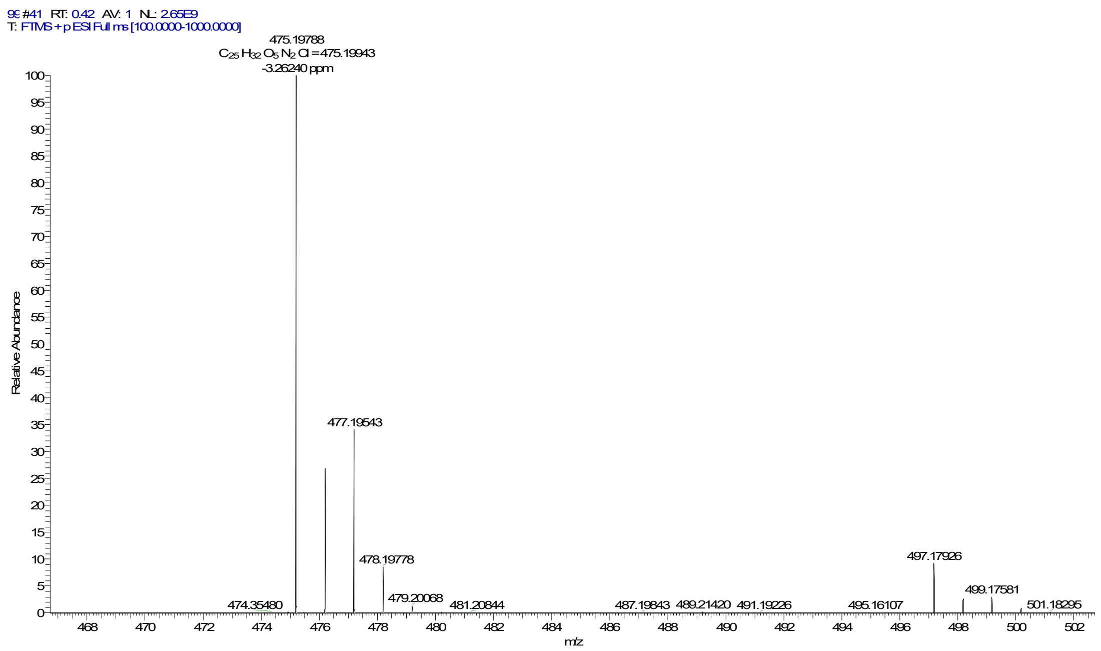

HRMS spectrum of target compound **A28**.

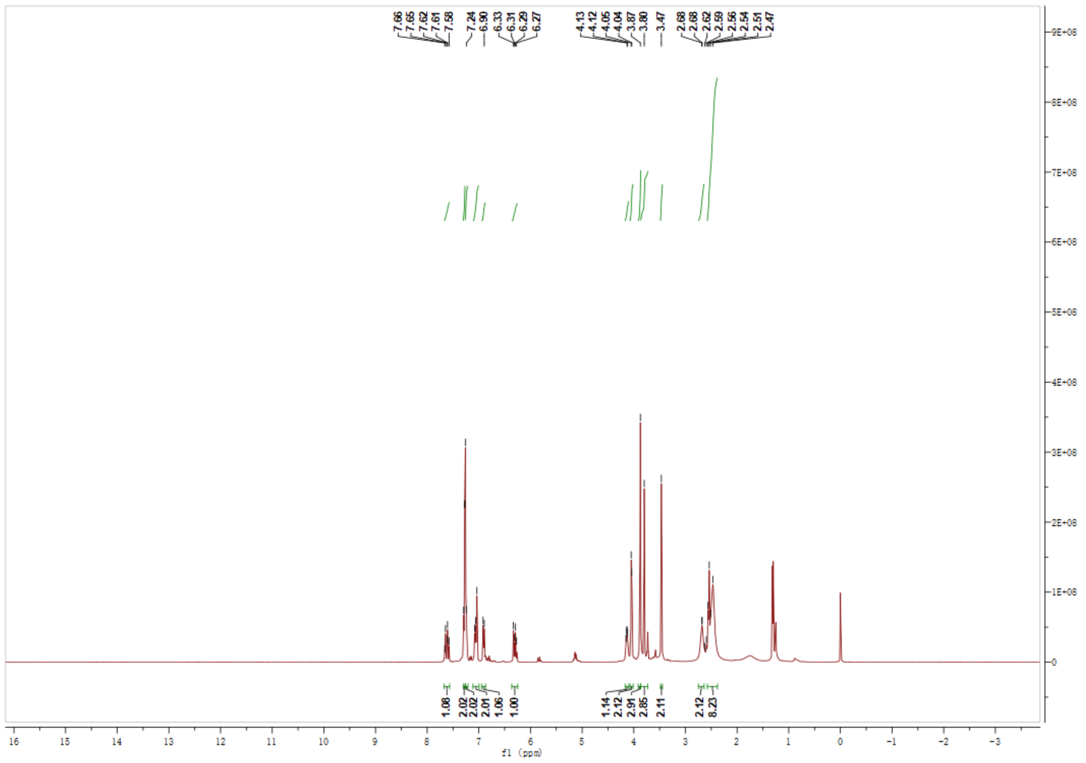

^{1}H NMR spectrum ($CDCl_3$, 400 MHz) of **A29**.

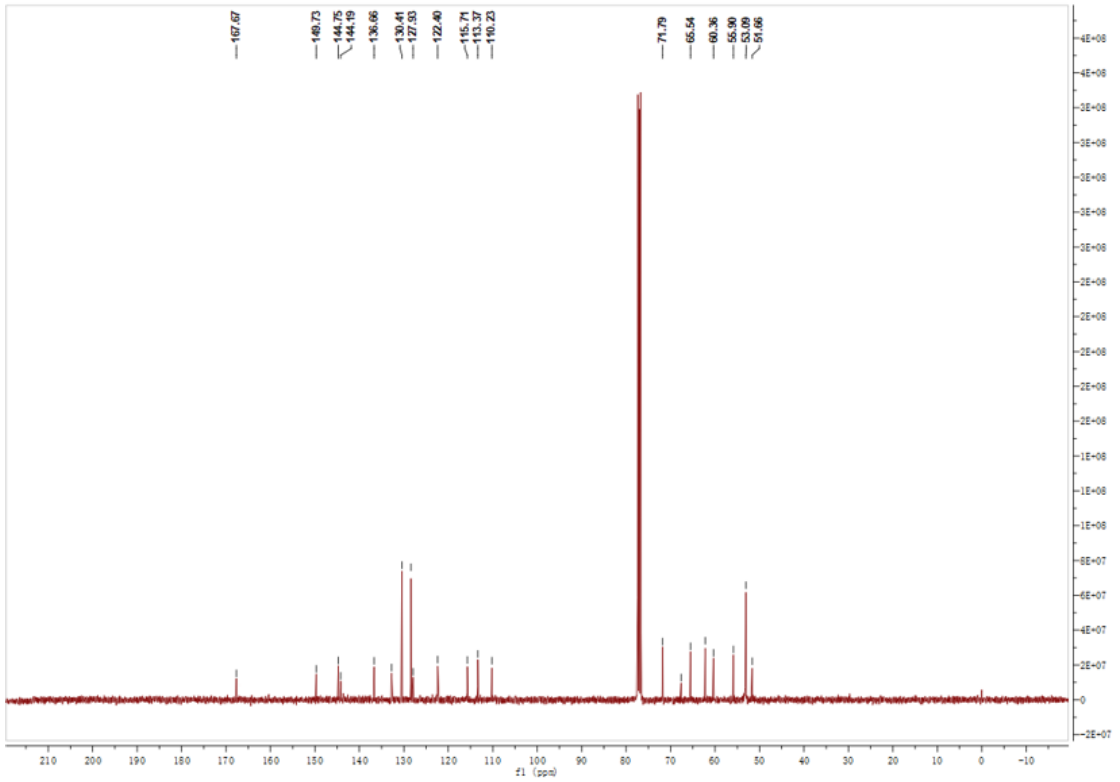

^{13}C NMR spectrum ($CDCl_3$, 101 MHz) of **A29**.

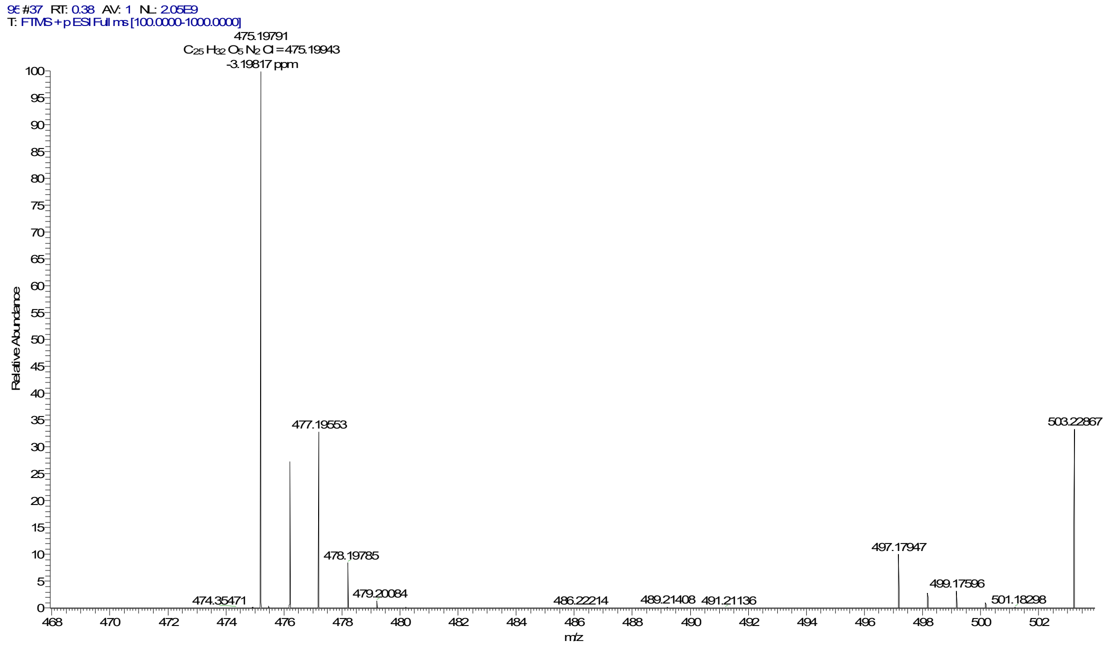

HRMS spectrum of target compound **A29**.

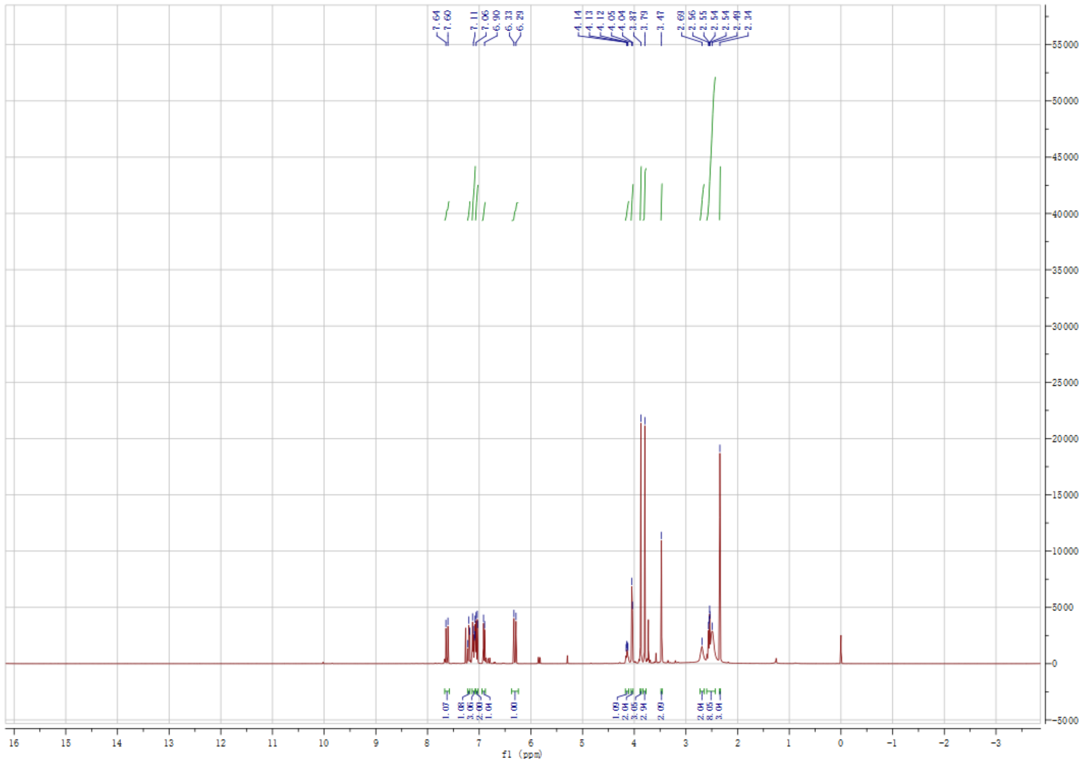

^{1}H NMR spectrum ($CDCl_3$, 400 MHz) of **A30**.

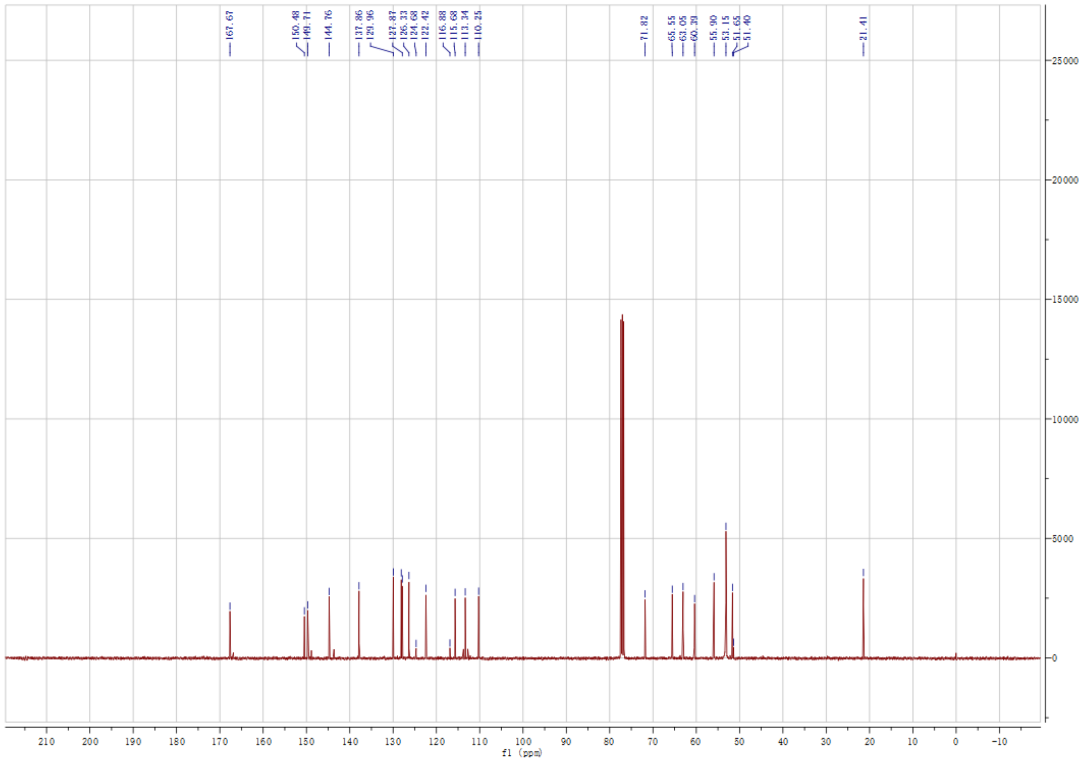

^{13}C NMR spectrum ($CDCl_3$, 101 MHz) of **A30**.

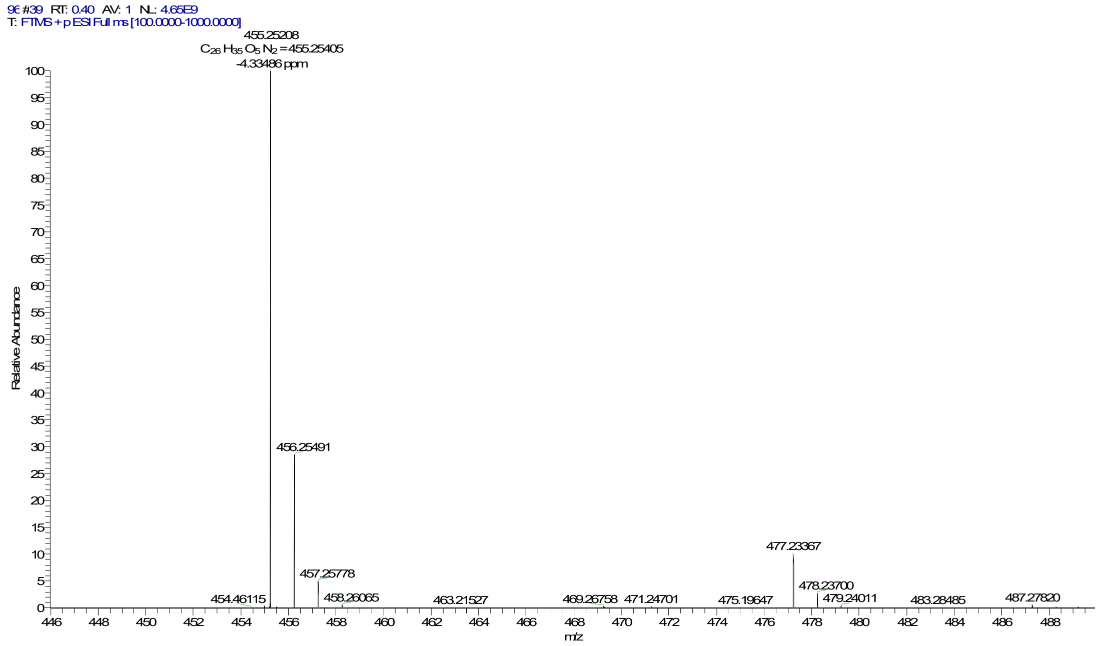

HRMS spectrum of target compound **A$_{30}$**.

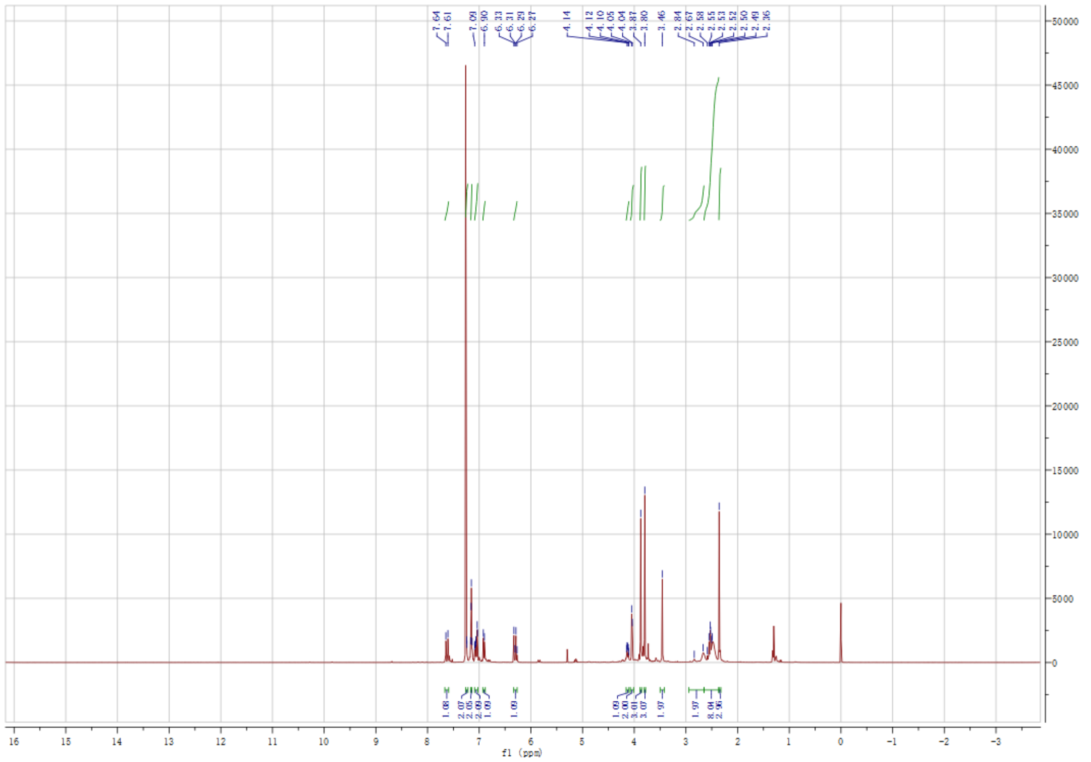

^{1}H NMR spectrum ($CDCl_3$, 400 MHz) of **A$_{31}$**.

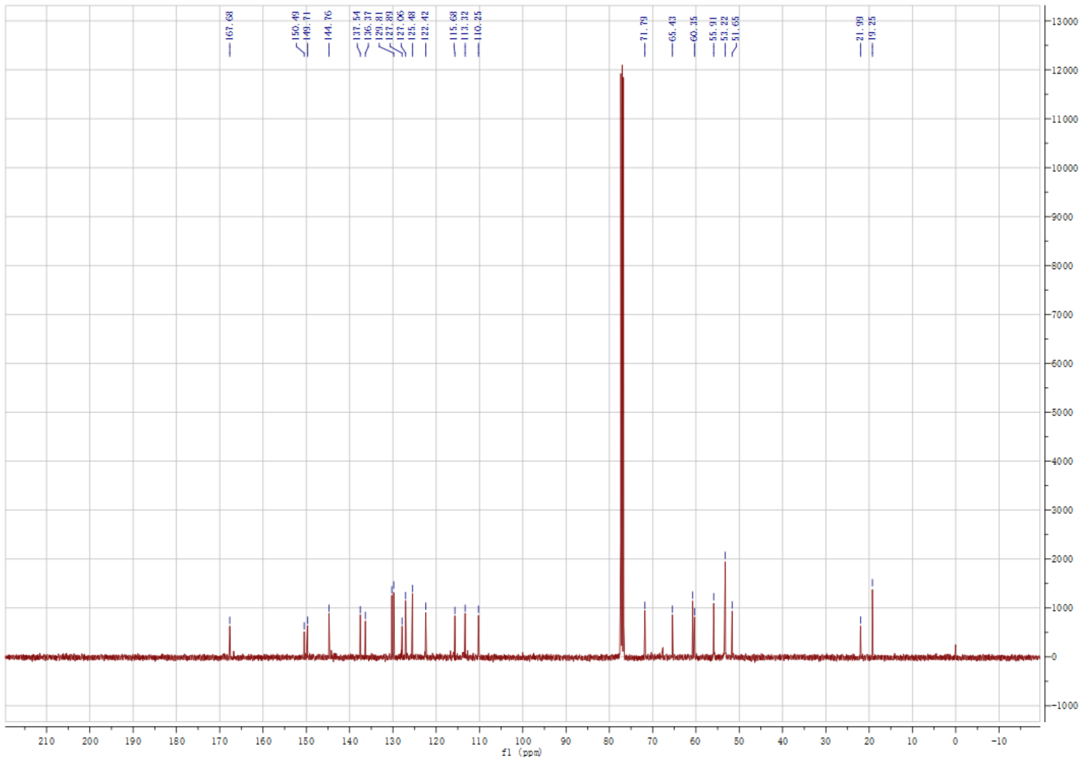

^{13}C NMR spectrum ($CDCl_3$, 101 MHz) of **A_{31}**.

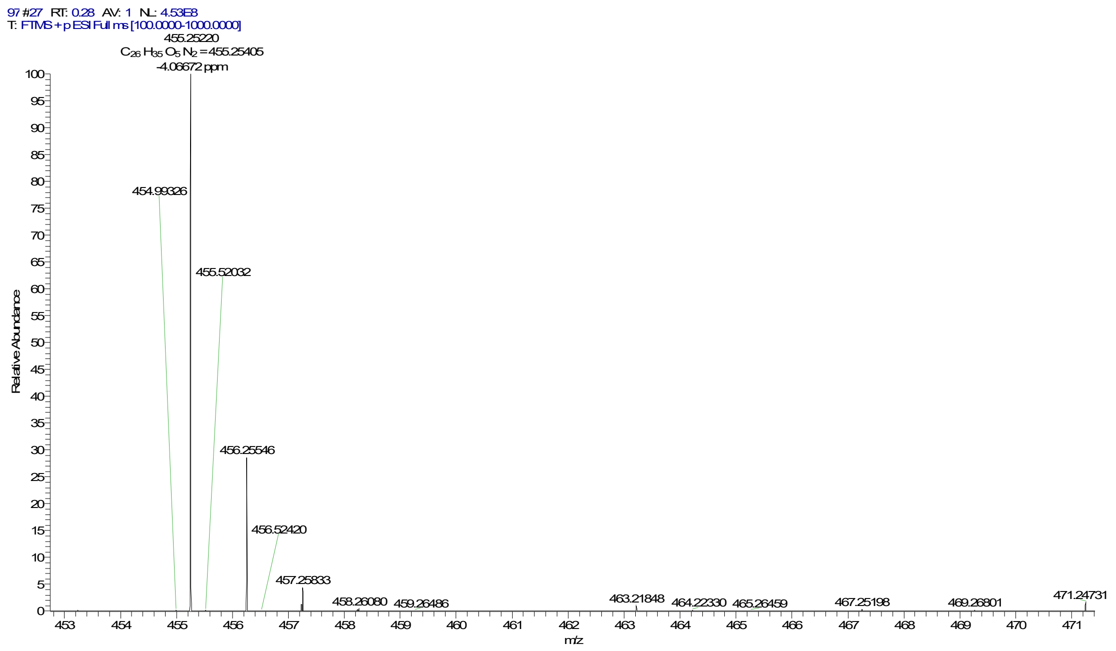

HRMS spectrum of target compound **A_{31}**.

6. Optical rotation test; characterization data; and ^{1}H NMR, ^{13}C NMR, and HRMS Spectra of *S*-A_{16}, *R*-A_{16}, *S*-A_{19}, *R*-A_{19}, *S*-A_{20}, *R*-A_{20}, *S*-A_{29}, and *R*-A_{29}.

6.1 Optical rotation results

Tabel S3. Optical rotation test results of ***S*-A_{16}**, ***R*-A_{16}**, ***S*-A_{19}**, ***R*-A_{19}**, ***S*-A_{20}**, ***R*-A_{20}**, ***S*-A_{29}**, and ***R*-A_{29}**.

Compound	***S*-A_{16}**	***R*-A_{16}**	***S*-A_{19}**	***R*-A_{19}**	***S*-A_{20}**	***R*-A_{20}**	***S*-A_{29}**	***R*-A_{29}**
Concentration	5 g/L	5 g/L	5 g/L	5 g/L	5 g/L	5 g/L	5 g/L	5 g/L
Optical rotation (max)	-0.801	34.196	31.797	25.998	-1.204	32.396	0.598	7.8
Optical rotation (min)	-1.802	31.999	30.998	25.199	-1.601	31.999	0	7
Average value	-1.44	33.019	31.398	25.519	-1.4	32.258	0.382	7.36
Measurement count	10 times	10 times	10 times	10 times	10 times	10 times	10 times	10 times
Mean square error	0.2636	0.7678	0.2974	0.269	0.1323	0.1334	0.1747	0.2787

6.2 Characterization data for compounds *S*-A_{16}, *R*-A_{16}, *S*-A_{19}, *R*-A_{19}, *S*-A_{20}, *R*-A_{20}, *S*-A_{29}, and *R*-A_{29}

***S*-3-methyl-(4-(2-hydroxy-3-(4-methylpiperidin-1-yl)propoxy)-3-methoxyphenyl)acrylate (*S*-A_{16})**

A yellow liquid, yield 64.1%; ^{1}H NMR (400 MHz, $CDCl_3$) δ 7.54 (dd, *J* = 15.9, 11.2 Hz, 1H, alkene-H), 7.03 – 6.94 (m, 2H, Ar-H), 6.83 (d, *J* = 8.3 Hz, 1H, Ar-H), 6.22 (dd, *J* = 15.9, 8.0 Hz, 1H, alkene-H), 4.17 – 4.08 (m, 1H, O-CH), 4.01 – 3.92 (m, 2H, O-CH_2), 3.80 (s, 3H, O-CH_3), 3.72 (s, 3H, O-CH_3), 3.34 – 3.22 (m, 2H, N-CH_2), 2.97 (d, *J* = 11.8 Hz, 1H, N-CH_2), 2.84 (d, *J* = 12.0 Hz, 1H, N-CH_2), 2.51 (d, *J* = 6.4 Hz, 2H, N-CH_2), 2.26 (t, *J* = 10.3 Hz, 1H, CH_2), 2.01 (t, *J* = 11.6 Hz, 1H, CH_2), 1.58 (d, *J* = 12.6 Hz, 2H, CH_2), 1.38 – 1.28 (m, 1H, CH), 0.86 (d, *J* = 6.2 Hz, 3H, CH_3); ^{13}C NMR (101 MHz, $CDCl_3$) δ 166.9, 149.6, 148.9, 144.0, 127.1, 121.7, 114.9, 112.5, 109.5, 71.0, 64.6, 60.0, 55.1, 52.1, 50.8, 33.3, 29.6, 20.9; HRMS (ESI): m/z calculated for $C_{20}H_{29}NO_5^+$: 364.2118; found: 364.2103.

***R*-3-methyl-(4-(2-hydroxy-3-(4-methylpiperidin-1-yl)propoxy)-3-methoxyphenyl)acrylate (*R*-A_{16})**

A yellow liquid, yield 74.7%; ^{1}H NMR (400 MHz, $CDCl_3$) δ 7.53 (dd, *J* = 15.9, 11.1 Hz, 1H, alkene-H), 6.98 (dd, *J* = 14.6, 5.0 Hz, 2H, Ar-H), 6.84 (d, *J* = 8.3 Hz, 1H,

Ar-H), 6.22 (dd, J = 15.9, 7.9 Hz, 1H, alkene-H), 4.18 – 4.06 (m, 1H, O-CH), 4.04 – 3.91 (m, 2H, O-CH_2), 3.80 (s, 3H, O-CH_3), 3.72 (s, 3H, O-CH_3), 3.56 (d, J = 20.4 Hz, 2H, N-CH_2), 2.96 (d, J = 11.8 Hz, 1H, N-CH_2), 2.83 (d, J = 11.9 Hz, 1H, N-CH_2), 2.51 (d, J = 6.8 Hz, 2H, N-CH_2), 2.26 (t, J = 11.6 Hz, 1H, CH_2), 2.00 (t, J = 11.6 Hz, 1H, CH_2), 1.58 (d, J = 12.6 Hz, 2H, CH_2), 1.33 (dd, J = 12.7, 4.7 Hz, 1H, CH), 0.86 (d, J = 6.2 Hz, 3H, CH_3); ^{13}C NMR (101 MHz, $CDCl_3$) δ 165.6, 148.4, 147.6, 142.7, 125.8, 120.4, 113.6, 111.3, 108.2, 69.7, 63.4, 58.8, 53.9, 50.9, 49.6, 31.8, 28.4, 19.7; HRMS (ESI): m/z calculated for $C_{20}H_{29}NO_5^+$: 364.2118; found: 364.2110.

(*S,E*)-methyl-3-(4-(2-hydroxy-3-morpholinopropoxy)-3-methoxyphenyl)acrylate (*S*-A_{19})

A yellow liquid, yield 76.8%; ^{1}H NMR (400 MHz, $CDCl_3$) δ 7.54 (dd, J = 15.9, 11.4 Hz, 1H, alkene-H), 7.04 – 6.96 (m, 2H, Ar-H), 6.83 (dd, J = 8.3, 2.0 Hz, 1H, Ar-H), 6.23 (dd, J = 15.9, 8.1 Hz, 1H, alkene-H), 4.11 (dd, J = 7.6, 4.9 Hz, 1H, O-CH), 4.02 – 3.96 (m, 2H, O-CH_2), 3.81 (s, 3H, O-CH_3), 3.72 (s, 3H, O-CH_3), 3.68 – 3.63 (m, 4H, O-CH_2), 2.59 (dd, J = 10.2, 5.3 Hz, 3H, N-CH_2), 2.53 – 2.47 (m, 2H, N-CH_2), 2.43 (dd, J = 10.2, 4.9 Hz, 2H, N-CH_2); ^{13}C NMR (101 MHz, $CDCl_3$) δ 166.6, 149.3, 148.6, 143.6, 126.9, 121.3, 114.7, 112.3, 109.2, 70.7, 65.8, 64.5, 59.9, 54.8, 52.7, 50.6; HRMS (ESI): m/z calculated for $C_{18}H_{25}NO_6^+$: 352.1754; found: 352.1750.

(*R,E*)-methyl-3-(4-(2-hydroxy-3-morpholinopropoxy)-3-methoxyphenyl)acrylate(*R*-A_{19})

A yellow liquid, yield 67.1%; ^{1}H NMR (400 MHz, $CDCl_3$) δ 7.54 (dd, J = 15.9, 11.3 Hz, 1H, alkene-H), 7.03 – 6.96 (m, 2H, Ar-H), 6.83 (dd, J = 8.3, 2.0 Hz, 1H, Ar-H), 6.23 (dd, J = 15.9, 8.0 Hz, 1H, alkene-H), 4.13 – 4.08 (m, 1H, O-CH), 4.00 – 3.97 (m, 2H, O-CH_2), 3.81 (s, 3H, O-CH_3), 3.72 (s, 3H, O-CH_3), 3.69 – 3.64 (m, 4H, O-CH_2), 2.60 (dd, J = 10.3, 5.3 Hz, 2H, N-CH_2), 2.54 – 2.49 (m, 2H, N-CH_2), 2.47 – 2.40 (m, 2H, N-CH_2); ^{13}C NMR (101 MHz, $CDCl_3$) δ 166.6, 149.3, 148.6, 143.6, 126.9, 121.3, 114.7, 112.3, 109.2, 70.7, 65.8, 64.4, 60.0, 54.8, 52.7, 50.6; HRMS (ESI): m/z calculated for $C_{18}H_{25}NO_6^+$: 352.1754; found: 352.1748.

(*S,E*)-methyl-3-(4-(3-(4-ethylpiperazin-1-yl)-2-hydroxypropoxy)-3-methoxyphenyl)acrylate (*S*-A_{20})

A yellow liquid, yield 65.7%; ^{1}H NMR (400 MHz, $CDCl_3$) δ 7.63 (d, *J* = 15.9 Hz, 1H, alkene-H), 7.08 (dd, *J* = 8.3, 1.9 Hz, 1H, Ar-H), 7.04 (d, *J* = 1.9 Hz, 1H, Ar-H), 6.91 (d, *J* = 8.3 Hz, 1H, Ar-H), 6.31 (d, *J* = 15.9 Hz, 1H, alkene-H), 4.16 (dq, *J* = 9.0, 4.6 Hz, 1H, O-CH), 4.07 – 4.01 (m, 2H, O-CH_2), 3.88 (s, 3H, O-CH_3), 3.80 (s, 3H, O-CH_3), 3.01 (s, 2H, N-CH_2), 2.73 (s, 2H, N-CH_2), 2.63 – 2.40 (m, 8H, O-CH_2), 1.10 (t, *J* = 7.2 Hz, 3H, CH_3); ^{13}C NMR (101 MHz, CDCl3) δ 167.5, 150.3, 149.8, 144.4, 127.8, 122.3, 115.6, 113.23, 110.1, 71.7, 65.5, 60.2, 55.8, 53.0, 52.5, 52.1, 51.5, 11.7; HRMS (ESI): m/z calculated for $C_{20}H_{30}N_2O_5^-$: 377.2071; found: 377.2064.

(*R,E*)-methyl-3-(4-(3-(4-ethylpiperazin-1-yl)-2-hydroxypropoxy)-3-methoxyphenyl)acrylate (*R*-A$_{20}$)

A yellow liquid, yield 70.3%; ^{1}H NMR (400 MHz, $CDCl_3$) δ 7.61 (dd, *J* = 15.9, 11.6 Hz, 1H, alkene-H), 7.11 – 7.01 (m, 2H, Ar-H), 6.91 (dd, *J* = 8.3, 2.2 Hz, 1H, Ar-H), 6.30 (dd, *J* = 15.9, 8.3 Hz, 1H, alkene-H), 4.20 – 4.12 (m, 1H, O-CH), 4.07 – 4.02 (m, 2H, O-CH_2), 3.88 (s, 3H, O-CH_3), 3.80 (s, 3H, O-CH_3), 2.74 (d, *J* = 1.6 Hz, 4H, N-CH_2), 2.50 (m, *J* = 21.7, 13.2, 5.7 Hz, 8H), 1.11 (t, *J* = 7.2 Hz, 3H, CH_3); ^{13}C NMR (101 MHz, $CDCl_3$) δ 165.5, 148.2, 147.5, 142.5, 125.7, 120.2, 113.5, 111.2, 108.1, 69.6, 65.5, 63.5, 58.2, 53.7, 50.5, 50.1, 49.5, 9.6; HRMS (ESI): m/z calculated for $C_{20}H_{30}N_2O_5^+$: 379.2227; found: 379.2164.

(*S,E*)-methyl-3-(4-(3-(4-(4-chlorobenzyl)piperazin-1-yl)-2-hydroxypropoxy)-3-methoxyphenyl)acrylate (*S*-A$_{29}$)

A yellow liquid, yield 63.7%; ^{1}H NMR (400 MHz, $CDCl_3$) δ 7.61 (dd, *J* = 15.9, 11.1 Hz, 1H, alkene-H), 7.31 – 7.27 (m, 3H, Ar-H), 7.24 (d, *J* = 2.0 Hz, 1H, Ar-H), 7.10 – 7.00 (m, 2H, Ar-H), 6.90 (d, *J* = 8.3 Hz, 1H, Ar-H), 6.30 (dd, *J* = 15.9, 7.9 Hz, 1H, alkene-H), 4.16 (dt, *J* = 9.5, 4.9 Hz, 1H, O-CH), 4.04 (d, *J* = 5.0 Hz, 2H, O-CH_2), 3.87 (s, 3H, O-CH_3), 3.79 (s, 3H, O-CH_3), 3.47 (s, 2H, N-CH_2), 2.76 – 2.63 (m, 2H, N-CH_2), 2.58 (dd, *J* = 6.6, 4.6 Hz, 2H, N-CH_2), 2.55 – 2.47 (m, 4H, N-CH_2); ^{13}C NMR (101 MHz, $CDCl_3$) δ 165.9, 148.6, 147.9, 143.0, 134.7, 131.1, 128.7, 126.7, 126.2, 120.7, 114.0, 111.6, 108.5, 70.0, 65.9, 63.8, 60.4, 58.7, 54.1, 51.1, 49.9; HRMS (ESI): m/z calculated for $C_{25}H_{31}N_2O_5Cl^+$: 475.1994; found: 475.1981.

(*R,E*)-methyl-3-(4-(3-(4-(4-chlorobenzyl)piperazin-1-yl)-2-hydroxypropoxy)-

3-methoxyphenyl)acrylate (*R*-A$_{29}$)

A yellow liquid, yield 70.3%; ^{1}H NMR (400 MHz, $CDCl_3$) δ 7.62 (d, *J* = 15.9 Hz, 1H, alkene-H), 7.32 – 7.20 (m, 4H, Ar-H), 7.11 – 7.01 (m, 2H, Ar-H), 6.90 (d, *J* = 8.3 Hz, 1H, Ar-H), 6.31 (d, *J* = 15.9 Hz, 1H, alkene-H), 4.16 (td, *J* = 9.5, 4.9 Hz, 1H, O-CH), 4.04 (d, *J* = 5.0 Hz, 2H, O-CH_2), 3.87 (s, 3H, O-CH_3), 3.79 (s, 3H, O-CH_3), 3.47 (s, 2H, N-CH_2), 2.73 – 2.68 (m, 2H, N-CH_2), 2.58 (dd, *J* = 16.4, 8.0 Hz, 4H, N-CH_2), 2.54 – 2.43 (m, 4H, N-CH_2); ^{13}C NMR (101 MHz, $CDCl_3$) δ 167.0, 149.8, 149.0, 144.1, 135.9, 132.2, 129.8, 127.8, 127.3, 121.8, 115.1, 112.7, 109.6, 71.1, 67.0, 65.0, 61.5, 59.8, 55.3, 52.3, 51.0; HRMS (ESI): m/z calculated for $C_{25}H_{31}N_2O_5Cl^+$: 475.1994; found: 475.1980.

6.3 ^{1}H NMR, ^{13}C NMR, and HRMS spectra of the target compounds *S*-A$_{16}$, *R*-A$_{16}$, *S*-A$_{19}$, *R*-A$_{19}$, *S*-A$_{20}$, *R*-A$_{20}$, *S*-A$_{29}$, and *R*-A$_{29}$

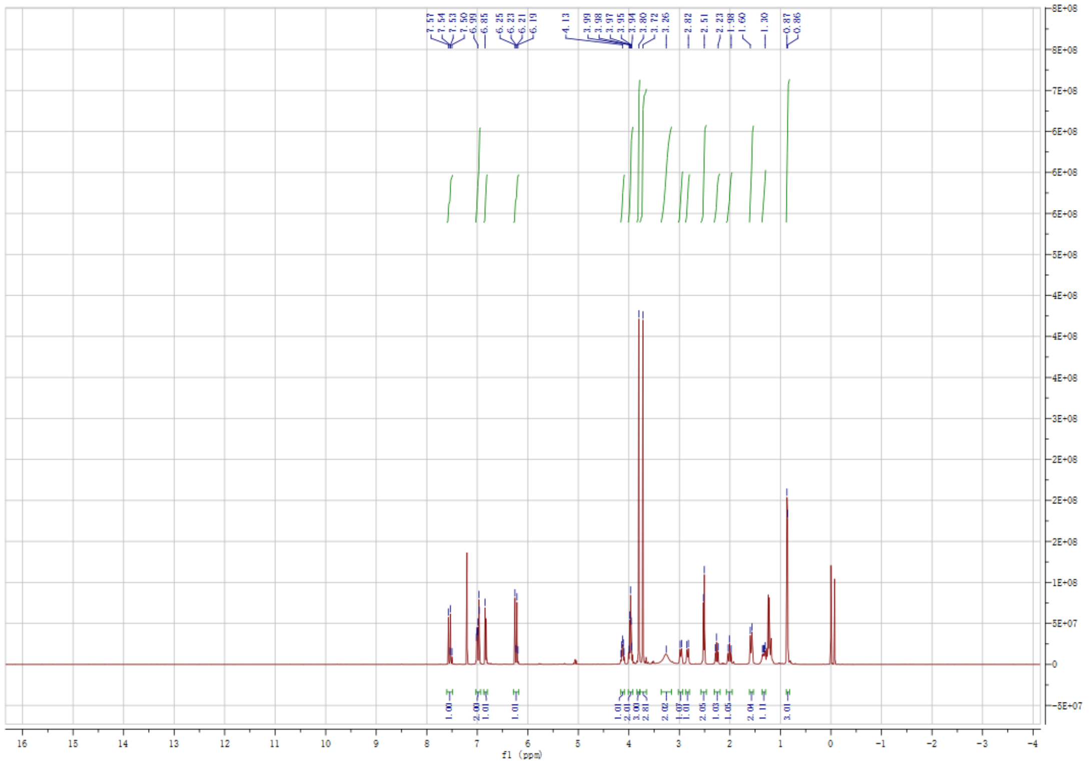

^{1}H NMR spectrum ($CDCl_3$, 400 MHz) of ***S*-A$_{16}$**.

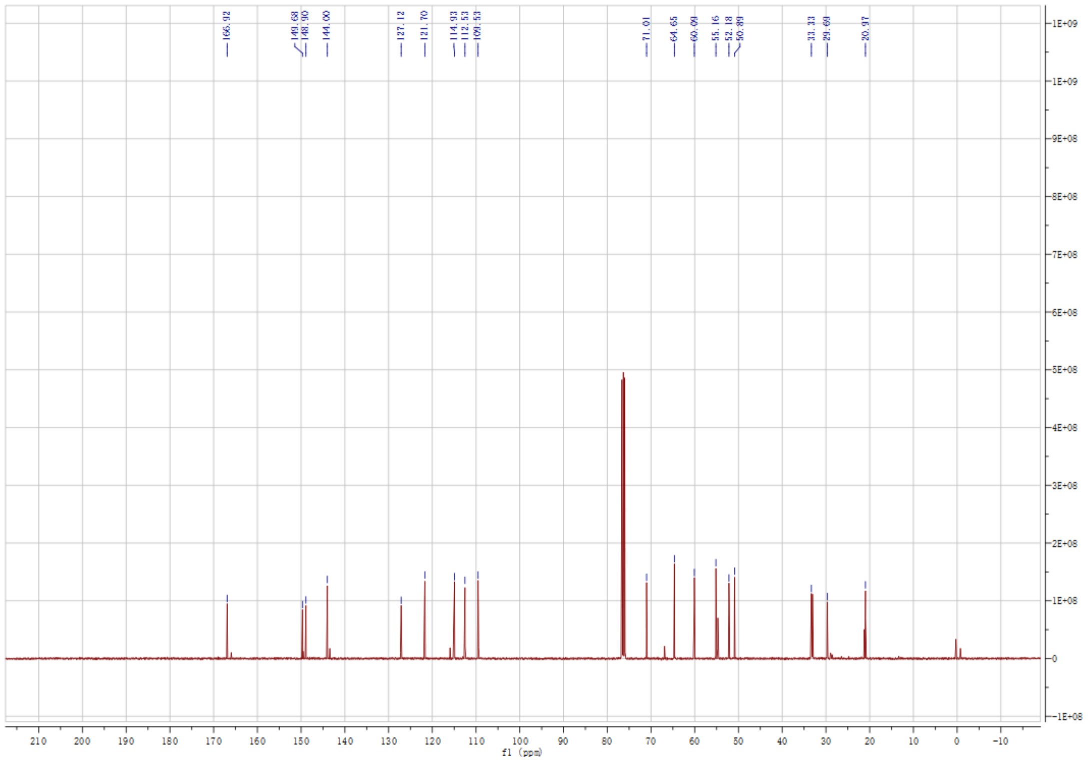

^{13}C NMR spectrum ($CDCl_3$, 101 MHz) of ***S*-A16**.

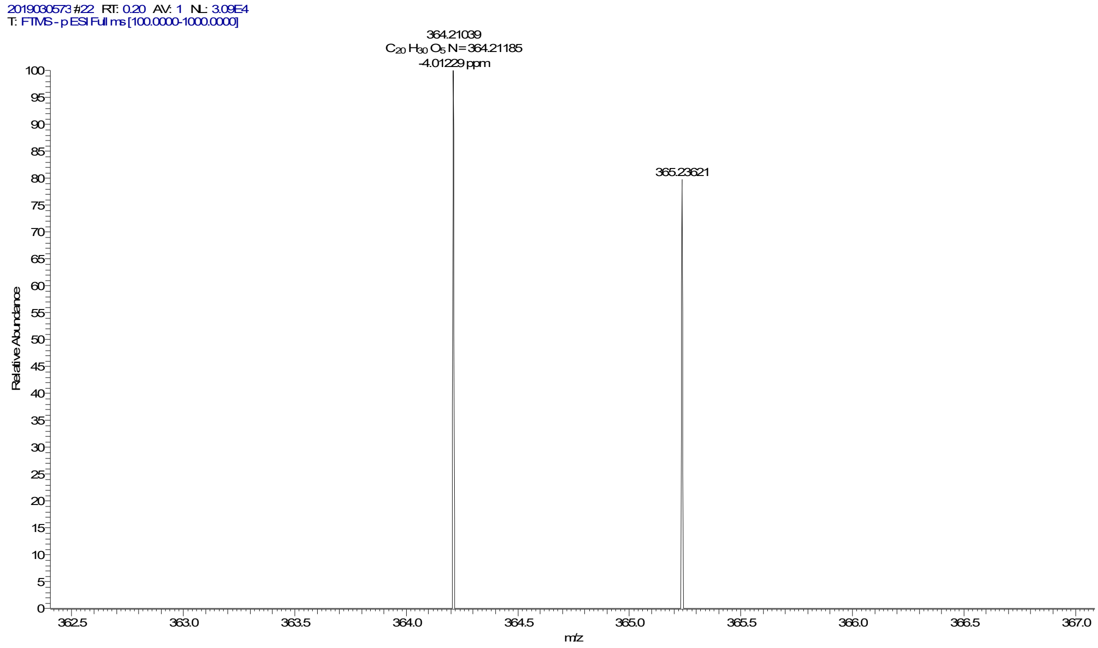

HRMS spectrum of target compound ***S*-A16**.

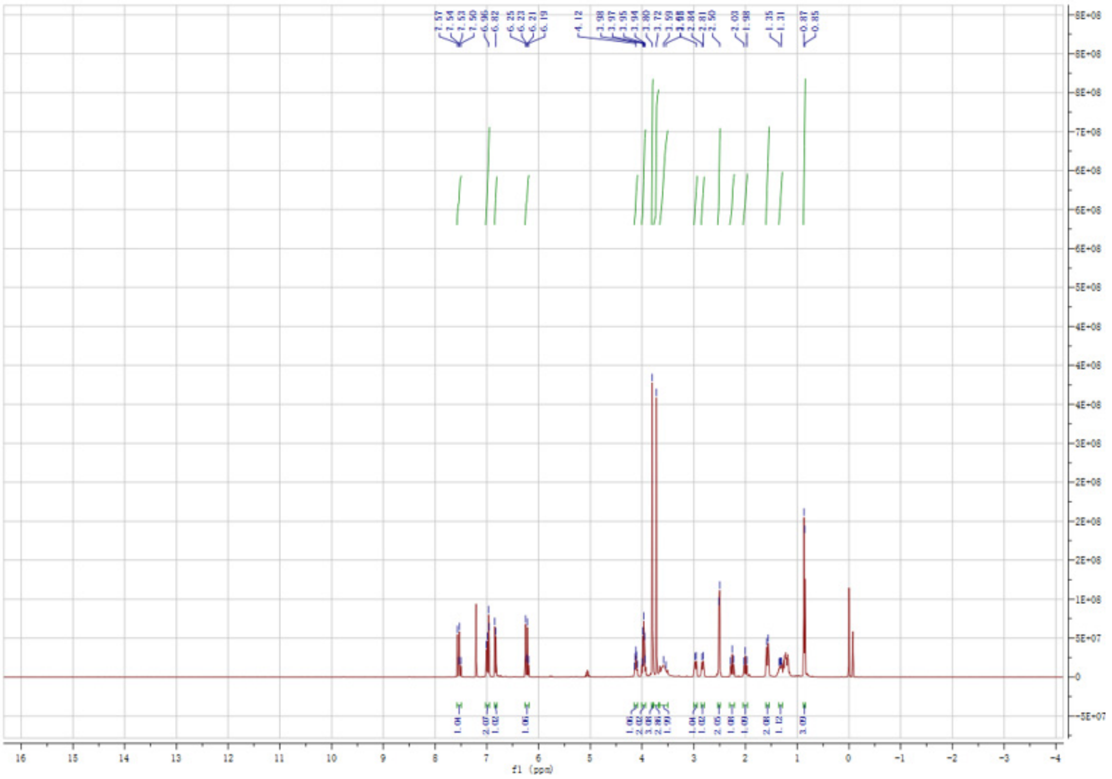

^{1}H NMR spectrum ($CDCl_3$, 400 MHz) of ***R*-A16**.

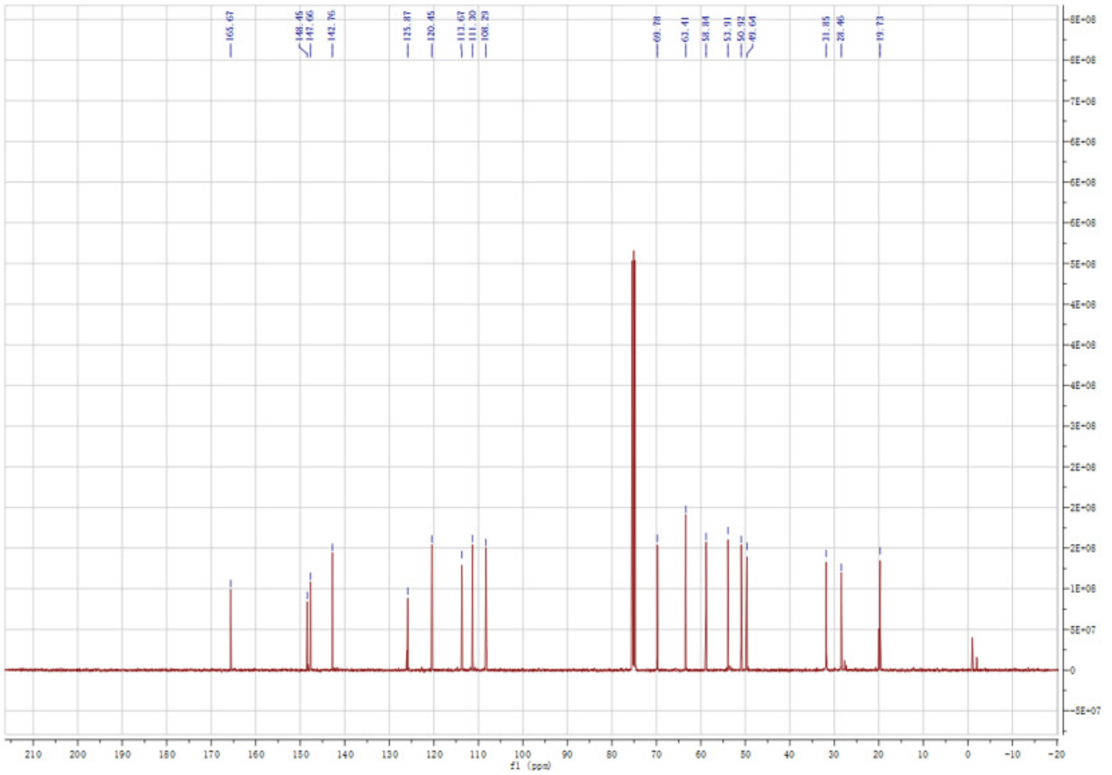

^{13}C NMR spectrum ($CDCl_3$, 101 MHz) of ***R*-A16**

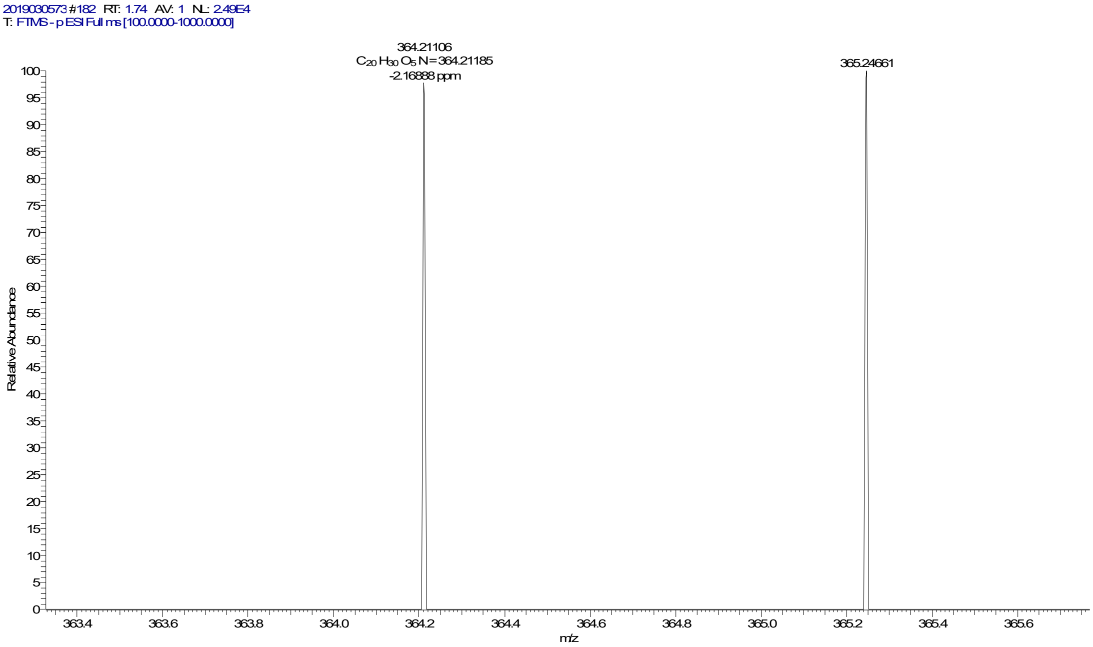

HRMS spectrum of target compound ***R*-A16**.

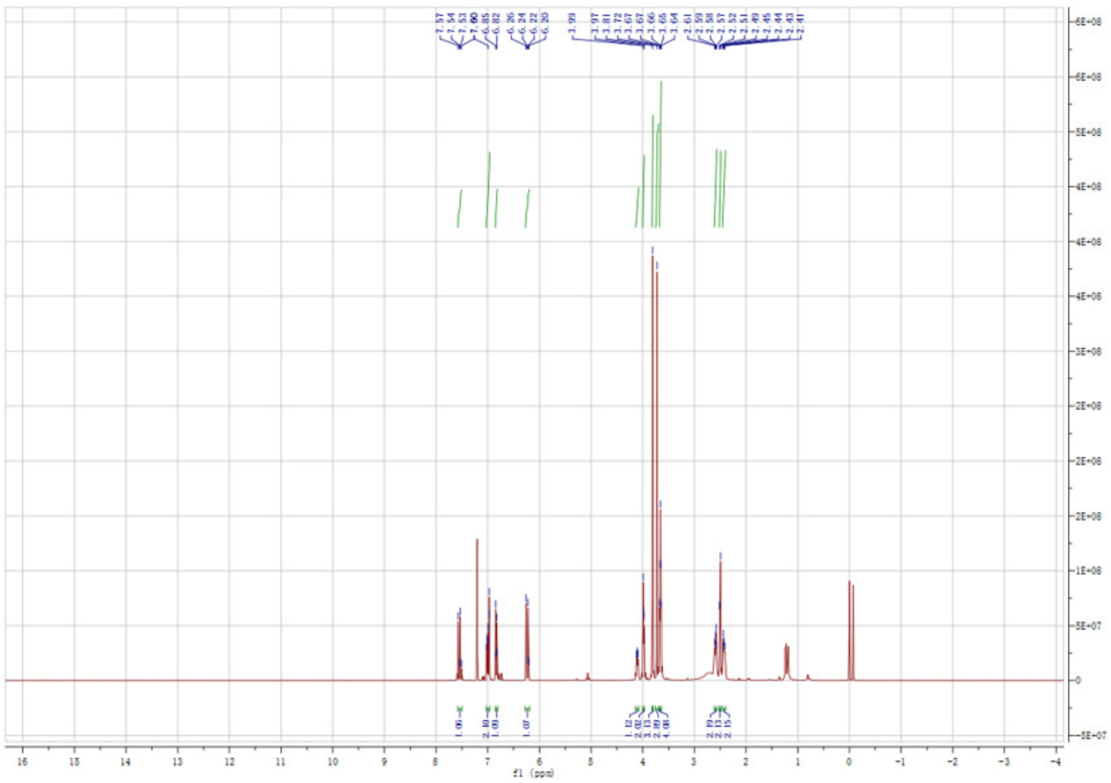

^{1}H NMR spectrum ($CDCl_3$, 400 MHz) of ***S*-A19**.

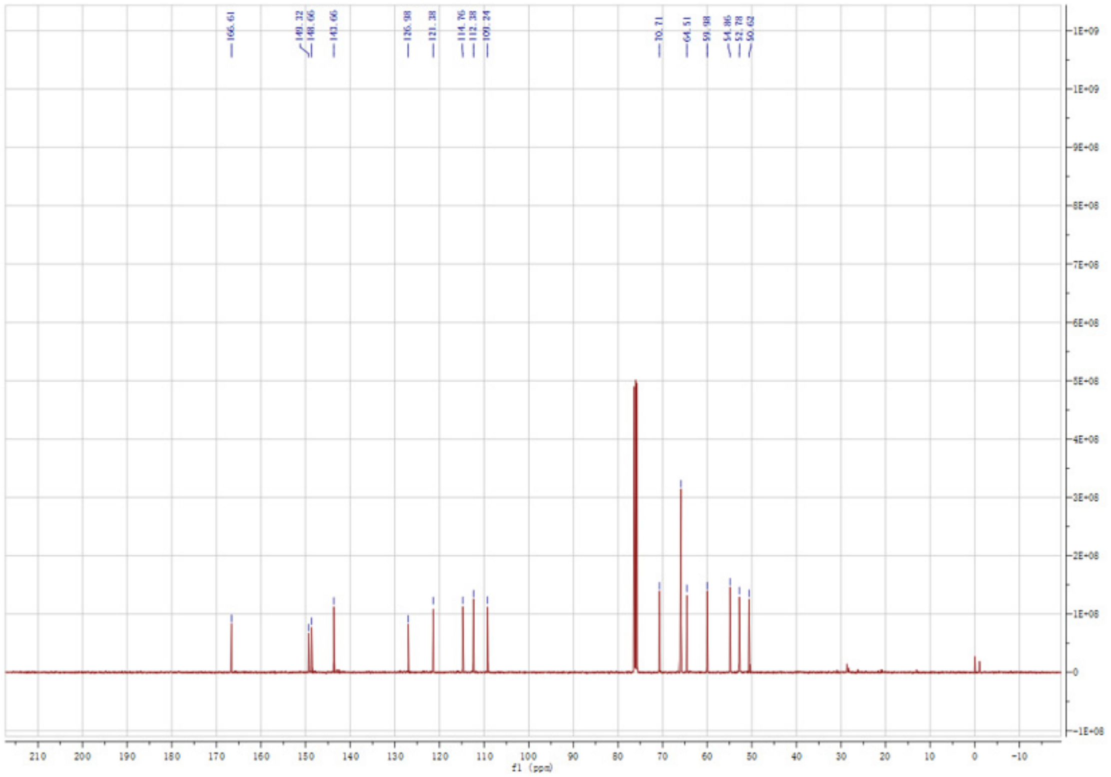

^{13}C NMR spectrum ($CDCl_3$, 101 MHz) of ***S*-A19**.

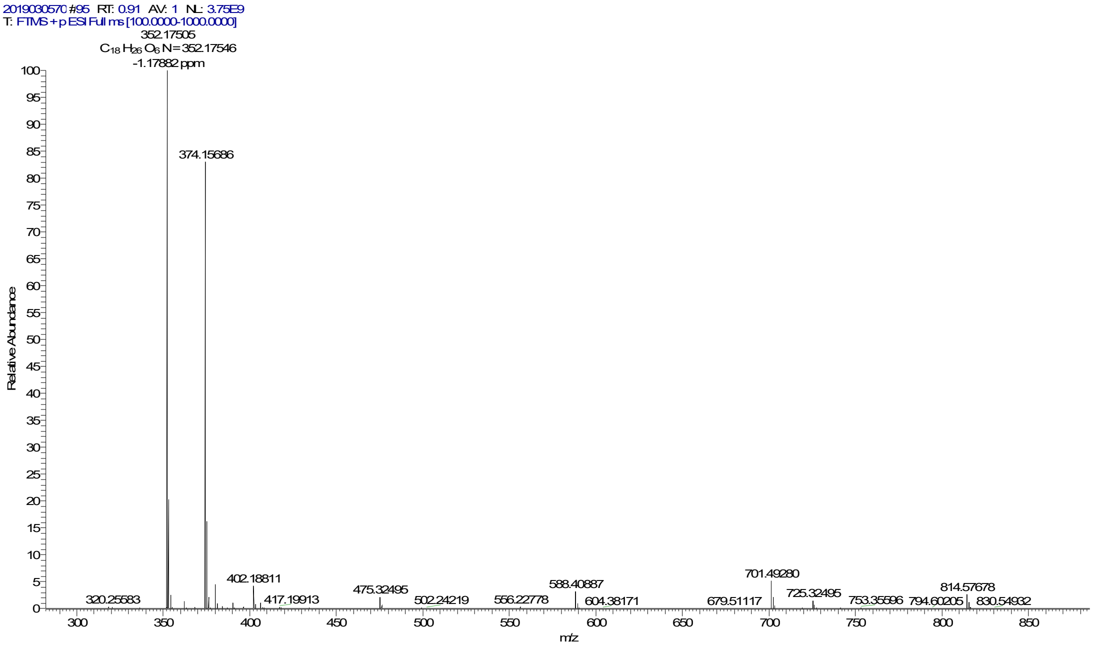

HRMS spectrum of target compound ***S*-A19**.

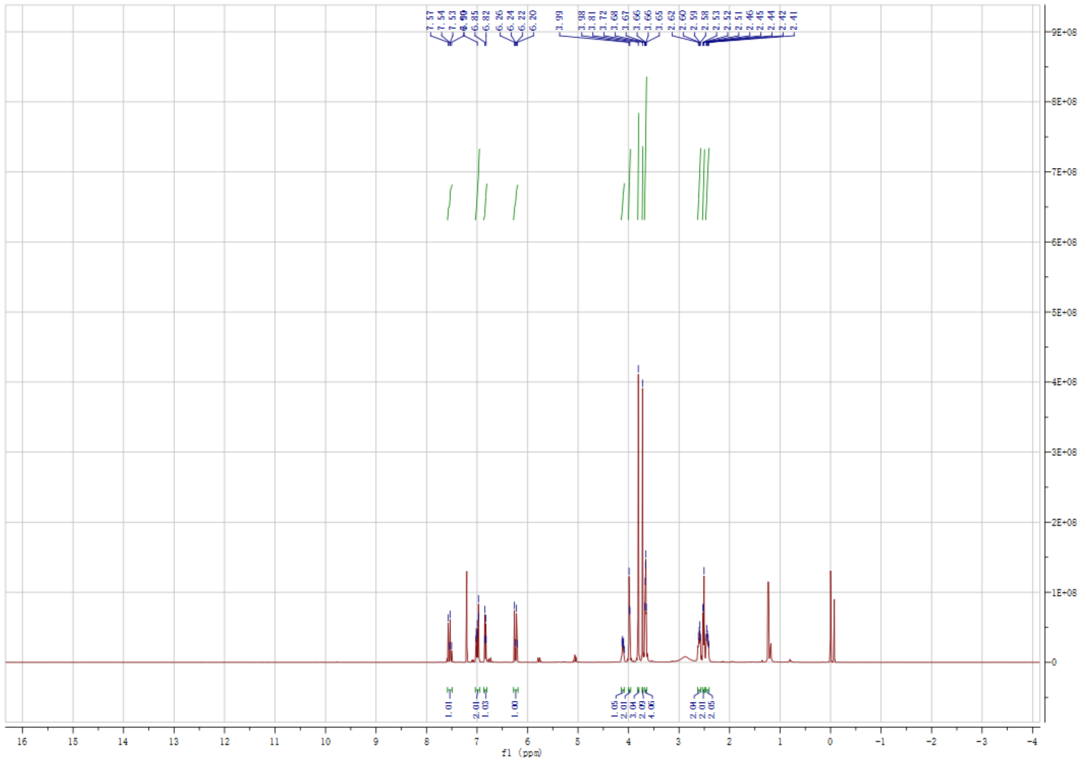

^{1}H NMR spectrum ($CDCl_3$, 400 MHz) of ***R*-A19**.

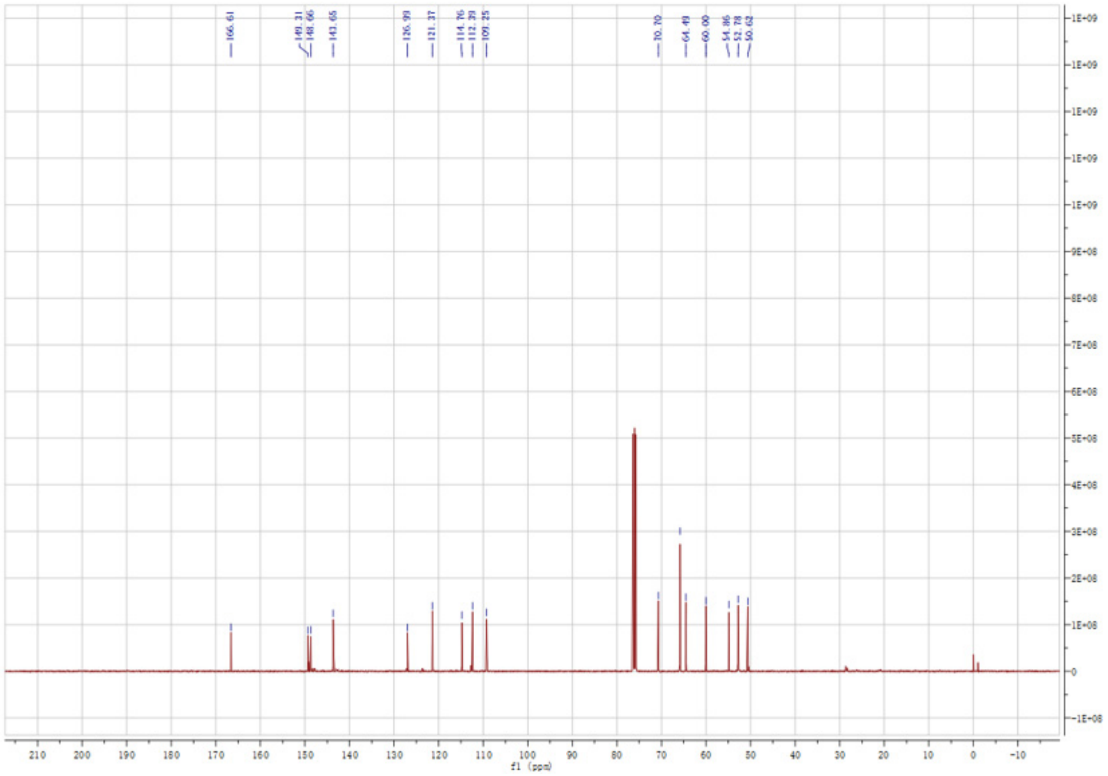

^{13}C NMR spectrum ($CDCl_3$, 101 MHz) of ***R*-A19**.

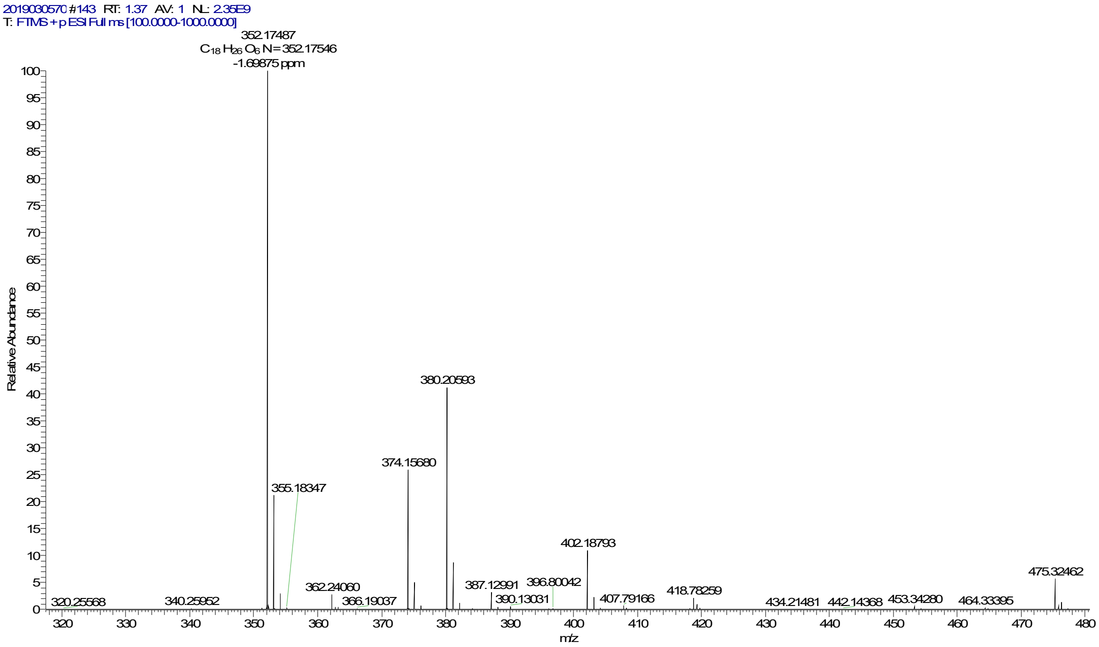

HRMS spectrum of target compound ***R*-A19**.

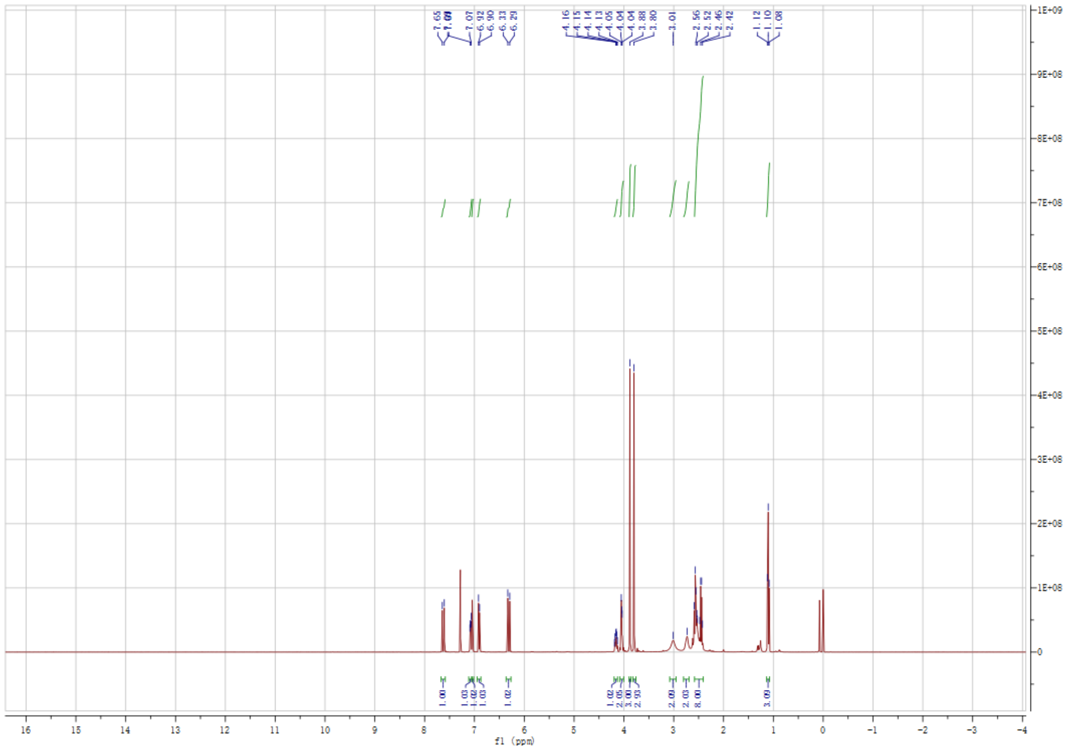

^{1}H NMR spectrum ($CDCl_3$, 400 MHz) of ***S*-A20**.

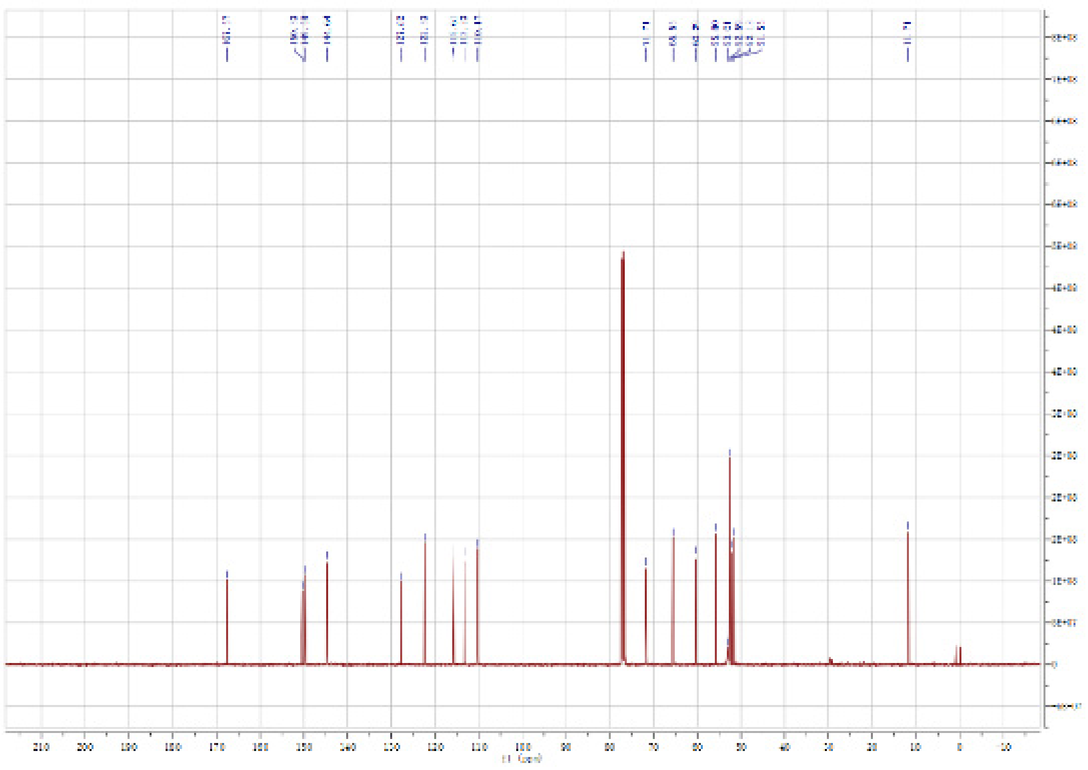

^{13}C NMR spectrum ($CDCl_3$, 101 MHz) of ***S*-A₂₀**.

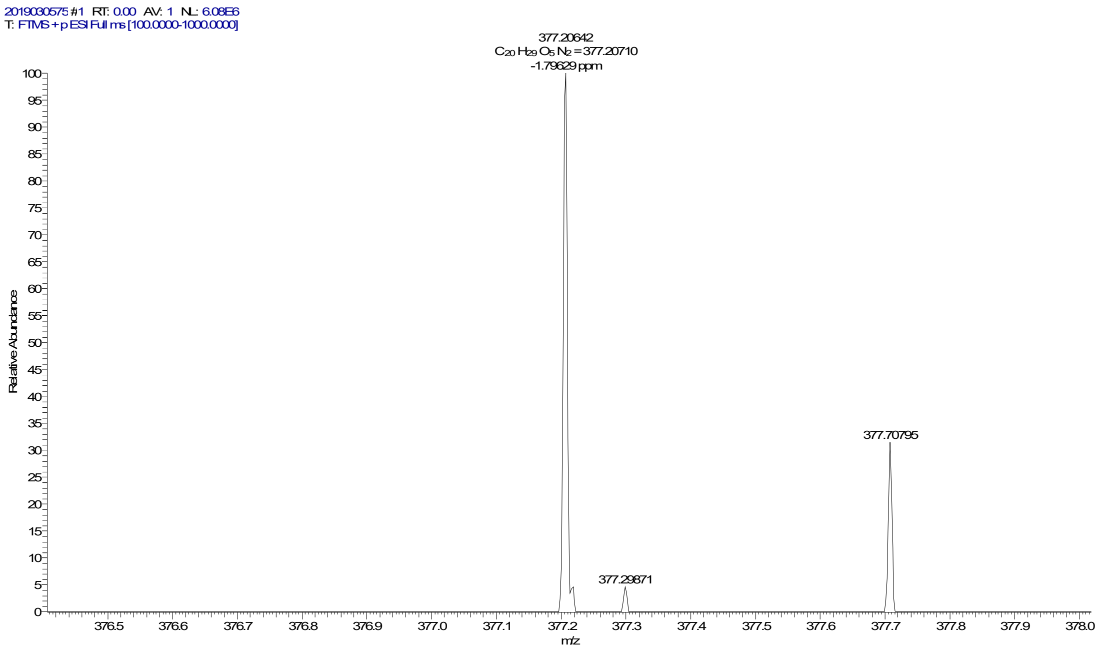

HRMS spectrum of target compound ***S*-A₂₀**.

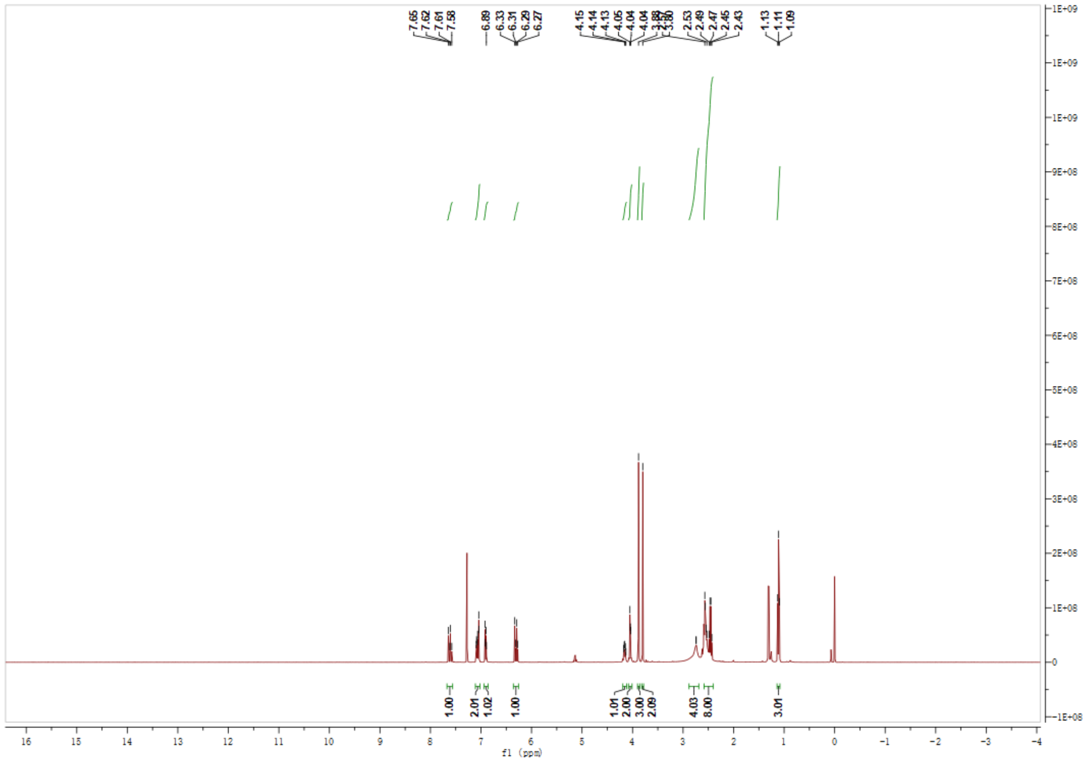

^{1}H NMR spectrum (CDCl$_3$, 400 MHz) of ***R*-A$_{20}$**.

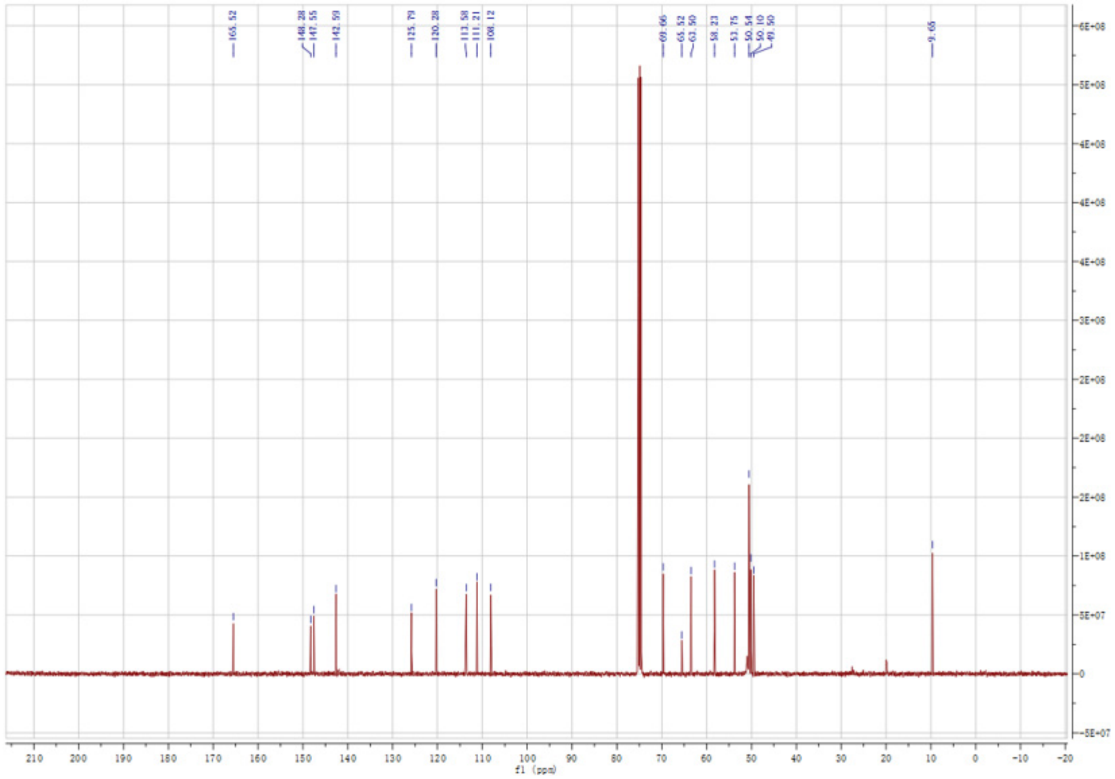

^{13}C NMR spectrum (CDCl$_3$, 101 MHz) of ***R*-A$_{20}$**.

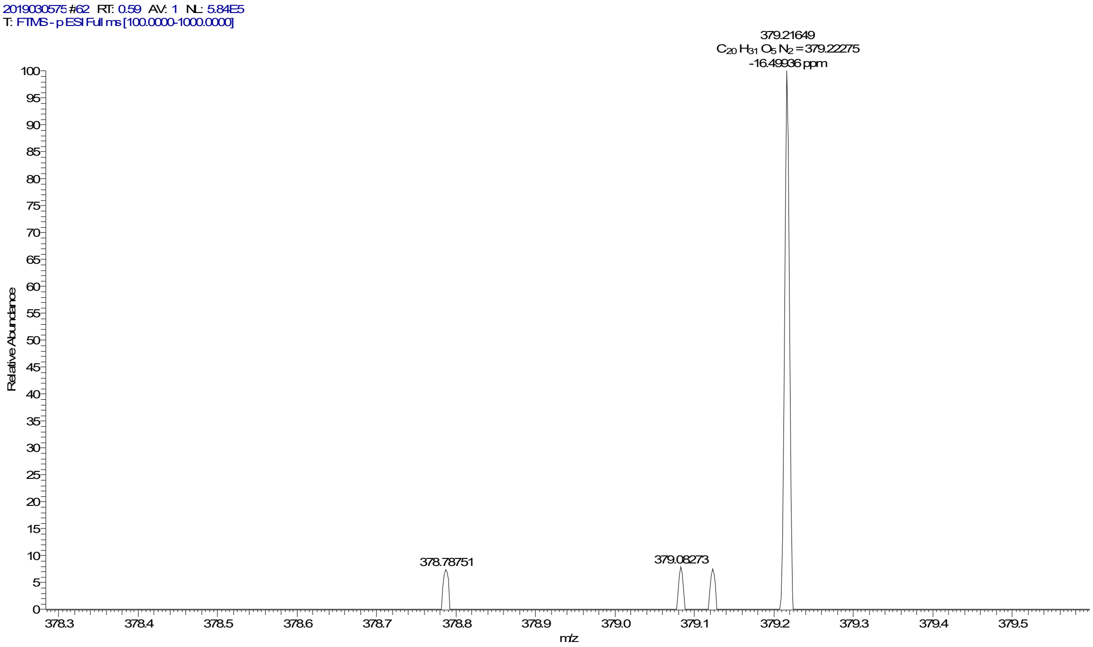

HRMS spectrum of target compound ***R*-A₂₀**.

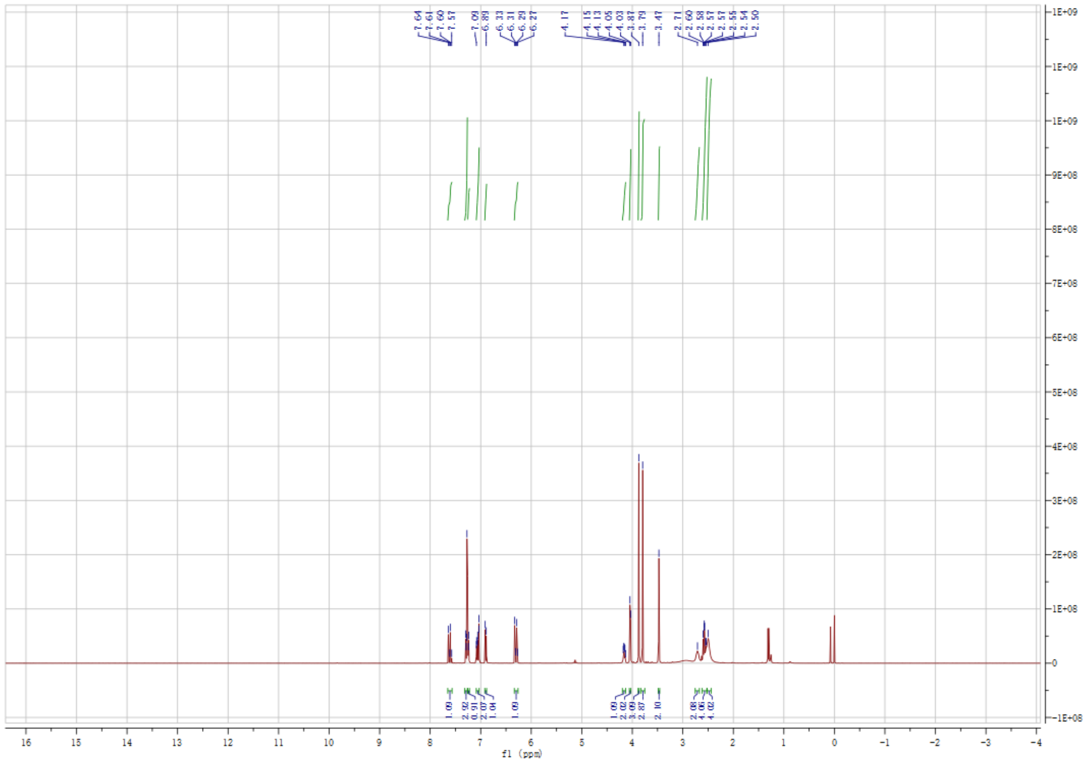

^{1}H NMR spectrum ($CDCl_3$, 400 MHz) of ***S*-A₂₉**.

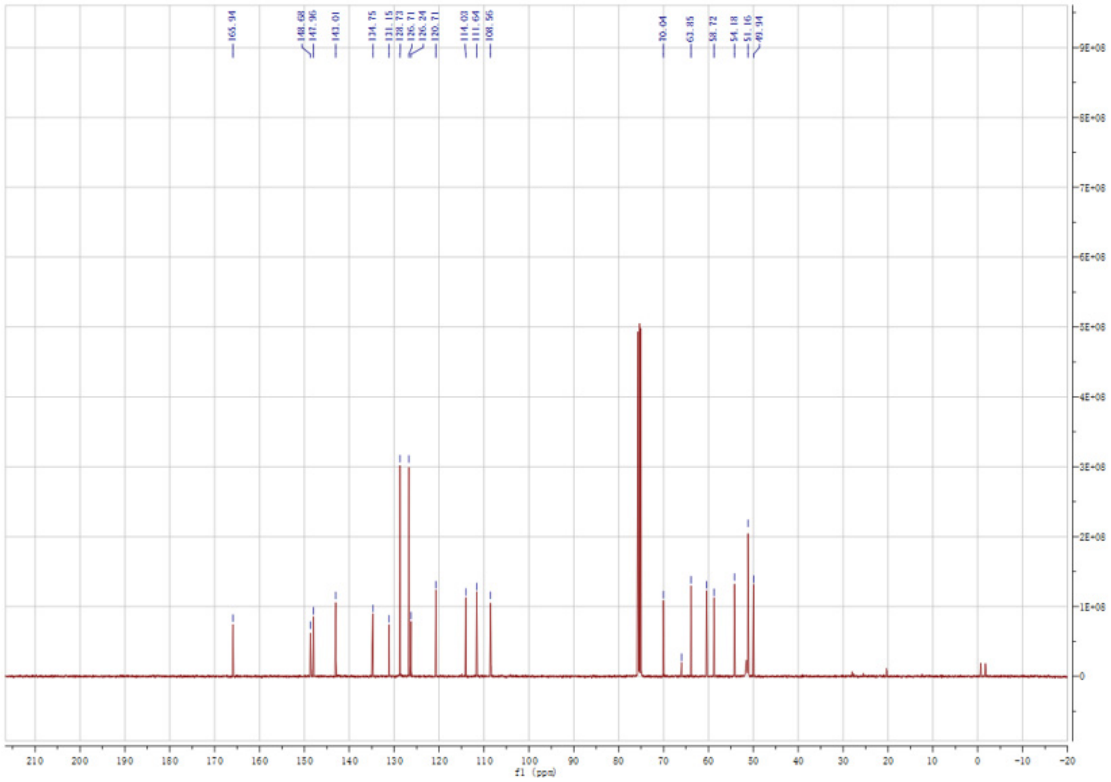

^{13}C NMR spectrum ($CDCl_3$, 101 MHz) of ***S*-A₂₉**.

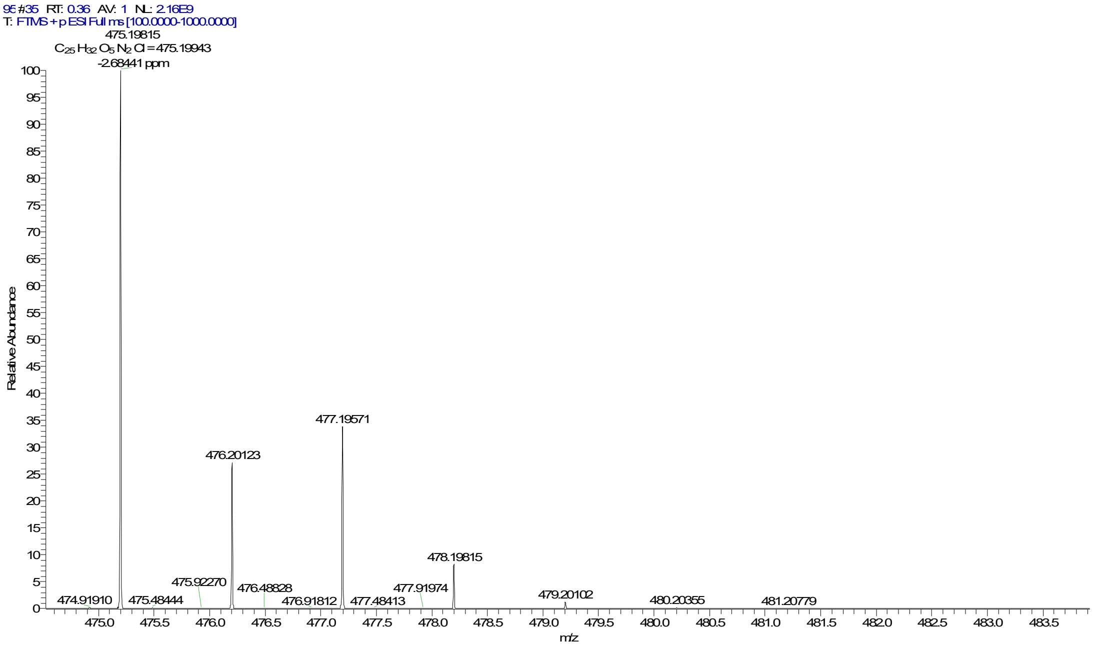

HRMS spectrum of target compound ***S*-A₂₉**.

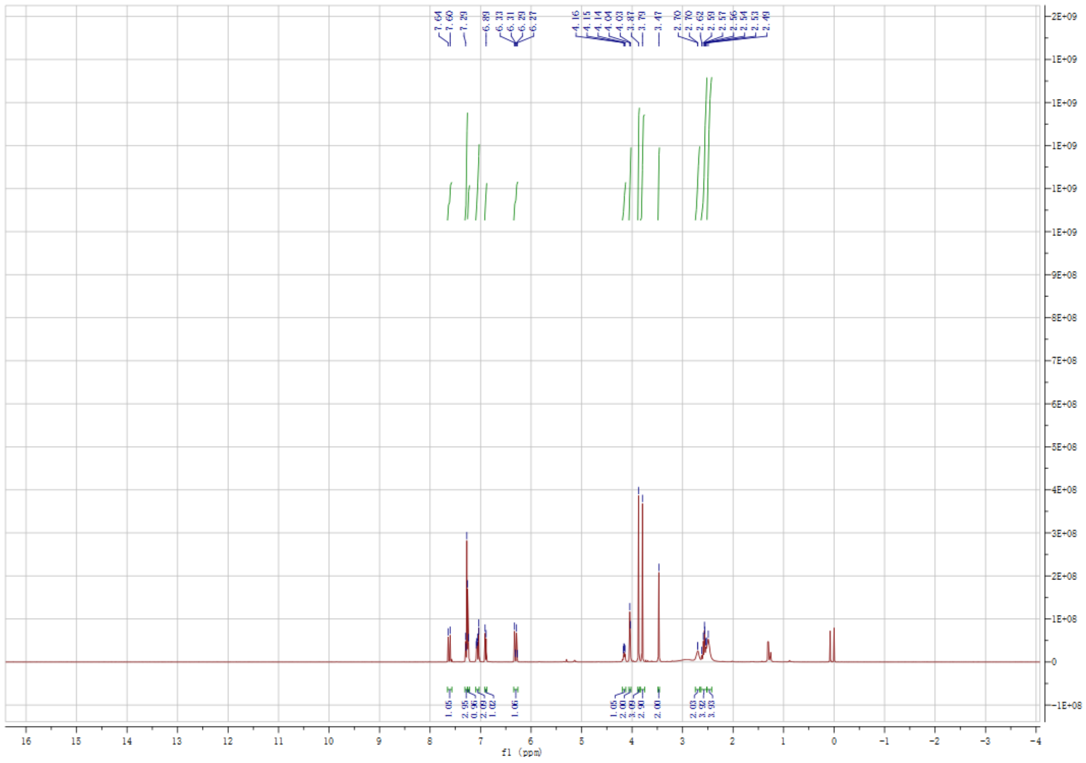

^{1}H NMR spectrum ($CDCl_3$, 400 MHz) of ***R*-A₂₉**.

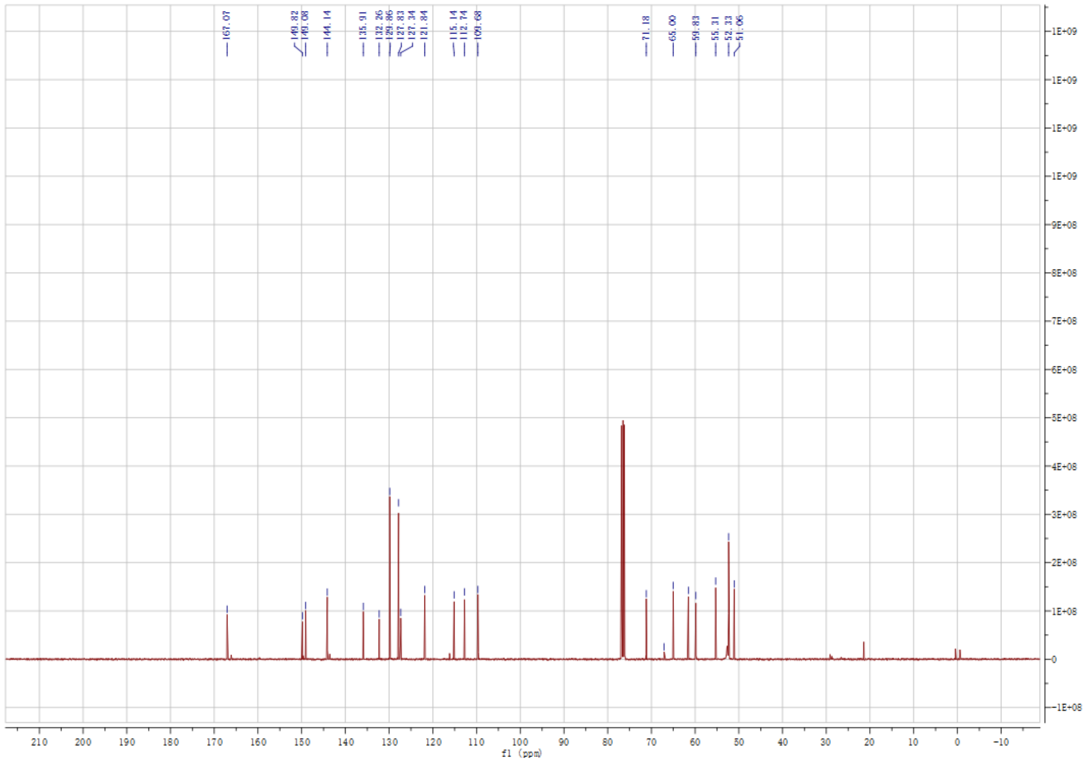

^{13}C NMR spectrum ($CDCl_3$, 101 MHz) of ***R*-A₂₉**.

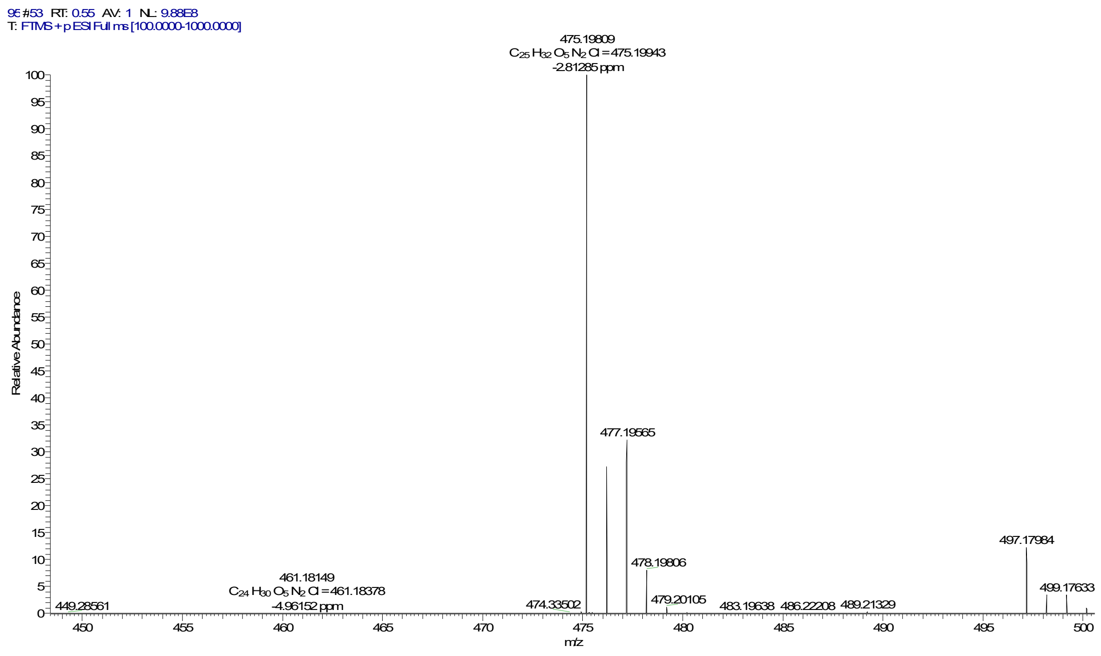

HRMS spectrum of target compound ***R*-A**$_{\mathbf{29}}$.
